# Comparative DFT study of metal-free Lewis acid-catalyzed C–H and N–H silylation of (hetero)arenes: mechanistic studies and expansion of catalyst and substrate scope†

**DOI:** 10.1039/c9ra07985h

**Published:** 2019-11-19

**Authors:** Pan Du, Jiyang Zhao

**Affiliations:** School of Life Science and Chemistry, Jiangsu Second Normal University Nanjing 210013 China; School of Environmental Science, Nanjing Xiaozhuang University Nanjing 211171 China jyzhao1981@163.com

## Abstract

Direct selective dehydrogenative silylation of thiophenes, pyridines, indoles and anilines to synthesize silyl-substituted aromatic compounds catalyzed by metal-free Lewis acids was achieved recently. However, there is still insufficient mechanistic data for these transformations. Using density functional theory calculations, we conducted a detailed investigation of the mechanism of the B(C_6_F_5_)_3_-catalyzed dehydrogenative silylation of *N*-methylindole, *N*,*N*-dimethylaniline and *N*-methylaniline. We successfully located the most favourable reaction pathways that can explain the experimental observations notably well. The most favourable pathway for B(C_6_F_5_)_3_-catalyzed C–H silylation of *N*-methylindole includes nucleophilic attack, proton abstraction and hydride migration. The C–H silylation of *N*,*N*-dimethylaniline follows a similar pathway to *N*-methylindole rather than that proposed by Hou's group. Our mechanism successfully explains that the transformations of *N*-methylindoline to *N*-methylindole produce different products at different temperatures. For *N*-methylaniline bearing both N–H and *para*-phenyl C–H bonds, the N–H silylation reaction is more facile than the C–H silylation reaction. Our proposed mechanism of N–H silylation of *N*-methylaniline is different from that proposed by the groups of Paradies and Stephan. Lewis acids Al(C_6_F_5_)_3_, Ga(C_6_F_5_)_3_ and B(2,6-Cl_2_C_6_H_3_)(*p*-HC_6_F_4_)_2_ can also catalyze the C–H silylation of *N*-methylindole like B(C_6_F_5_)_3_, but the most favourable pathways are those promoted by *N*-methylindoline. Furthermore, we also found several other types of substrates that would undergo C–H or N–H silylation reactions under moderate conditions. These findings may facilitate the design of new catalysts for the dehydrogenative silylation of inactivated (hetero)arenes.

## Abstract 2

We investigated the mechanism of the dehydrosilylation of (hetero)arenes and extended the scope of the silylation catalysts and substrates.

## Graphical abstract

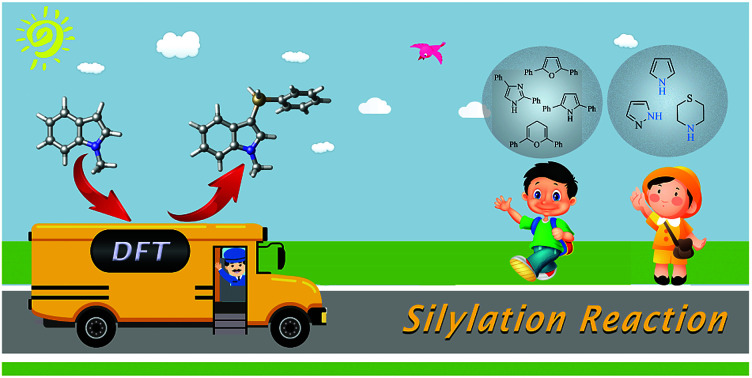

## 1 Introduction

(Hetero)arylsilanes are highly important species in molecular and materials synthesis,^1^ medicinal chemistry,^2^ and synthetic chemistry.^3^ The direct selective C–H silylation of (hetero)arenes with hydrosilanes is atom-economical, efficient and convenient and is one of the most attractive methods for synthesizing silyl-substituted aromatic compounds.^4^ To date, various metal-catalyzed C–H silylation reactions between (hetero)arenes and hydrosilanes have been reported.^5^ Generally, the use of metal catalysts has several shortcomings such as the high cost of the catalysts, difficulty of catalyst recycling, and addition of additives. Therefore, the development of a cheap and environmentally friendly synthetic method for the preparation of silylated (hetero)arenes is still a challenging task.

**Scheme 1:**
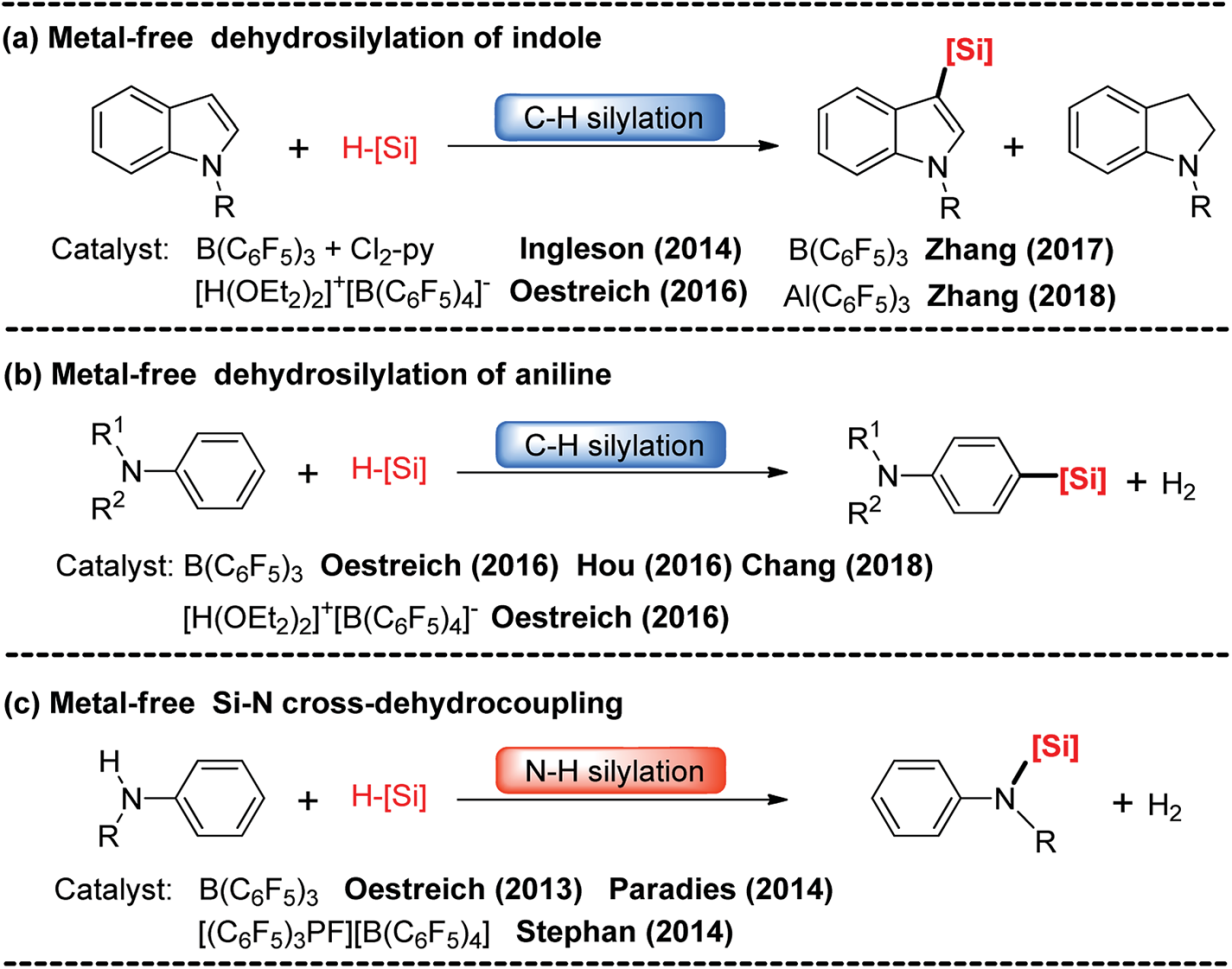
Metal-free catalyzed C–H silylation of indoles (a) and anilines (b), and N-H silylation of anilines (c).

Recently, boron-catalyzed hydrosilylation of (hetero)arenes has attracted considerable attention. The pioneering work in this area was performed by Kawashima^6^ and Ingleson groups.^7^ They accomplished intramolecular dehydrogenative silylation of 2-(SiR_2_H)-biphenyls using Lewis acid B(C_6_F_5_)_3_ or Ph_3_CB(C_6_F_5_)_4_ as the catalysts. Since then, C–H silylations of thiophenes, pyridines, indoles and anilines with hydrosilanes using Lewis acid B(C_6_F_5_)_3_, Al(C_6_F_5_)_3_ or Brønsted acid [H(OEt_2_)_2_]^+^[B(C_6_F_5_)_4_]^−^ as the catalysts have been reported (Scheme 1(a and b)).^8–13^ Furthermore, N–H silylations of aniline with hydrosilanes catalyzed by B(C_6_F_5_)_3_ and [(C_6_F_5_)_3_PF][B(C_6_F_5_)_4_] were observed by the Paradies and Stephan groups (Scheme 1(c)).^14^

**Scheme 2:**
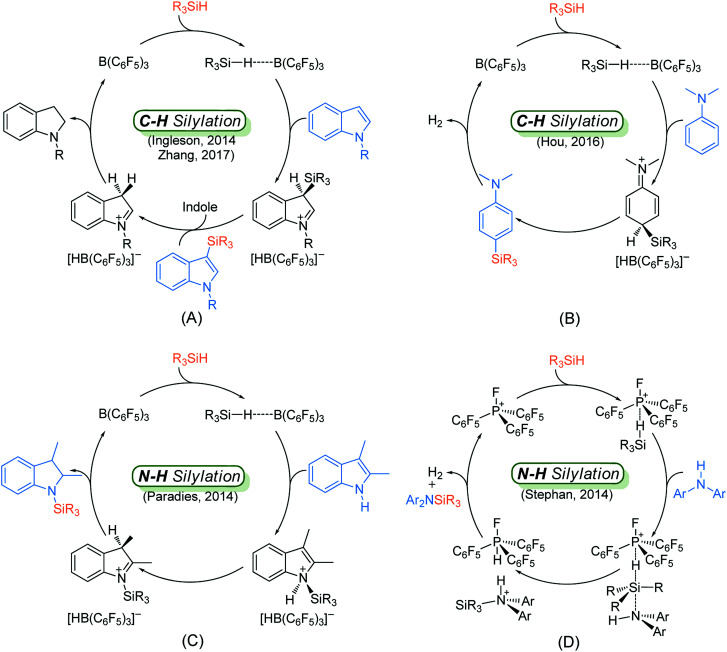
Proposed mechanisms for the Lewis acid catalyzed C–H silylation of indole (A) and aniline (B), N–H silylation of indole (C) and aniline (D).

Although many catalytic cycles for the silylation of aromatic C–H bonds using hydrosilanes have been proposed in previous experimental studies, few mechanistic investigations have been performed.^15^ Ingleson^8^ and Zhang^11^ groups proposed a possible reaction pathway for B(C_6_F_5_)_3_-catalyzed C–H silylation of indole, which includes nucleophilic attack, proton abstraction and hydride migration (exhibited in Scheme 2A). However, Hou's group^9*b*^ proposed a different mechanism for the C–H silylation of aniline catalyzed by B(C_6_F_5_)_3_, which involves nucleophilic attack and H_2_ release (Scheme 2B). For the N–H silylation of indole, Paradies and co-workers^14*c*^ proposed a catalytic cycle involving *N*-silylation, rearrangement and reduction (Scheme 2C). Stephan group investigated the mechanism of N–H silylation of anilines catalyzed by [(C_6_F_5_)_3_PF][B(C_6_F_5_)_4_] based on density functional theory (DFT) calculations. They found a dehydrocoupling mechanism that contains Si–N bond formation and H_2_ liberation (Scheme 2D). Moreover, the mechanism of B(C_6_F_5_)_3_-catalyzed silylation of carbonyl group have been established both experimentally and theoretically.^16^ Nevertheless, the mechanism for N–H silylation of aniline catalyzed by B(C_6_F_5_)_3_ has not been elucidated yet. And it is not clear which C–H silylation mechanism (Scheme 2A and B) is more reasonable. More importantly, if a substrate bears both C–H and N–H bonds, which type of silylation reaction is more likely to occur? Hence, in this work, we performed DFT calculations to investigate the molecular mechanisms of B(C_6_F_5_)_3_-catalyzed dehydrogenative silylation of indole and aniline with hydrosilanes and extend the scope of the silylation catalysts and substrates.

## 2 Computational details

All calculations were performed with the Gaussian09 program.^17^ Geometry optimizations and energy calculations were performed using the M06-2X functional^18^ that was proven to be accurate for describing weak interactions. The 6-311G(d,p) basis set was used for all atoms in the substrates and catalyst.^19^ Geometry optimizations are conducted in chlorobenzene and benzene solutions (using the self-consistent reaction field (SCRF) method with the IEFPCM solvation model^20^ with default parameters). We have performed harmonic-frequency-analysis calculations at the same level of theory to obtain the relevant thermodynamic energy corrections and to determine whether the optimized stationary points are minima or saddle points. At the M06-2x/6-311G(d,p) geometries, the energies were further refined by M06-2x/6-311++G(d,p) single-point energy calculations with the effect of the solvent taken into account by the IEFPCM solvation model. To determine the reasonability of our computational method, the wb97xd functional was also used to investigate the rate-limiting step in our mechanism. The calculated Gibbs free energies are for *T* = 298.15 K and 1 atm. Furthermore, important transition states were determined by intrinsic reaction coordinate (IRC) analysis.^21^ The 3D-optimized structures in this paper were displayed using the CYLview visualization program.^22^

Since our studied reactions involve multicomponent changes, the entropy contributions to the free energies for the reactions in the solvent will be overestimated. In addition, there are no standard quantum mechanics-based methods to accurately calculate the entropy in solution. In this study, based on “the theory of free volume”,^23^ corrections were added to calculate free energies; generally, for 2-to-1 (or 1-to-2) reactions, a correction of −2.6 (or 2.6) kcal mol^−1^ was added.

## 3 Results and discussion

In this section, we will discuss the B(C_6_F_5_)_3_-catalyzed dehydrosilylation of *N*-methylindole, *N*,*N*-dimethylaniline and *N*-methylaniline with PhSiH_3_ successively. Then, we explore other possible catalysts and select other substrates to undergo C–H or N–H silylation as in the case of indoles or anilines.

### 3.1 C–H silylation of *N*-methylindole

Based on our calculations, the B(C_6_F_5_)_3_-catalyzed C–H silylation of *N*-methylindole with PhSiH_3_ including three elementary steps: (1) nucleophilic attack of the indole to the Si centre of PhSiH_3_, (2) proton abstraction by a second *N*-methylindole, and (3) hydride migration to form indoline and regenerate B(C_6_F_5_)_3_. Moreover, we also examined the conversion of indoline to indole and the second silylation step of *N*-methylindole.

#### 3.1.1 Nucleophilic attacks of indole to B–Si complex

**Fig. 1:**
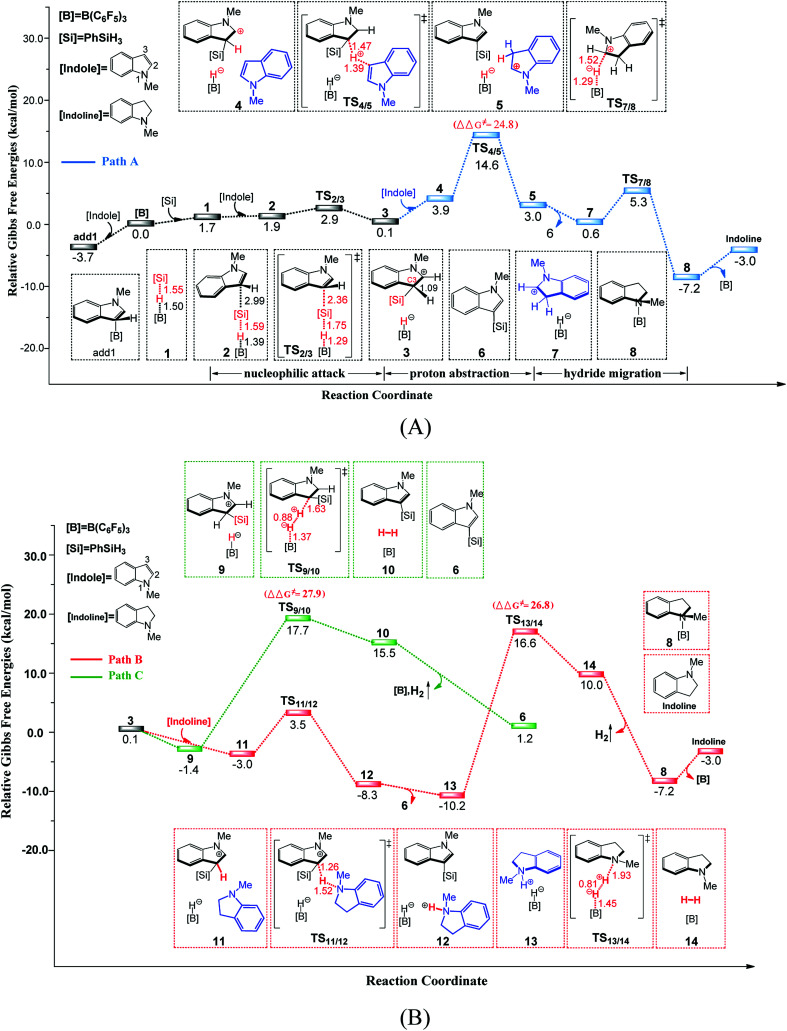
Free-energy profile for the B(C_6_F_5_)_3_-catalyzed C–H silylation of *N*-methylindole with PhSiH_3_, along with the optimized structures of the stationary points (path A in (A) and paths B and C in (B)). Key bond lengths are given in Å.

Fig. 1 illustrates the mechanism of the B(C_6_F_5_)_3_-catalyzed C–H silylation of *N*-methylindole with PhSiH_3_, together with the energy and geometry results. The optimized structures of all of the species along the reaction pathway are shown in Fig. S1 and S2 in the ESI.†

First, a stable Lewis adduct add1 is formed from indole and B(C_6_F_5_)_3_. Because the add1 is 3.7 kcal mol^−1^ lower than that of free Lewis acid and base, it can be expected that there is an equilibrium between Lewis adduct add1 and the free B(C_6_F_5_)_3_ and indole. When PhSiH_3_ participates in the reaction, a B–Si complex 1 is generated. Natural population analysis shows that charge of the Si center of complex 1 increases to 1.14 from 0.87 in PhSiH_3_, suggesting that the Si center becomes more electrophilic. Then, the C3 atom of *N*-methylindole attacks complex 1 at the Si center *via* a barrierless transition state TS_2/3_ (6.6 kcal mol^−1^, relative to add1) through the concerted linear backside S_N_2 attack, giving intermediate 3. The C–Si bond formation and Si–H bond braking take place simultaneously in TS_2/3_. It is worth noting that the mechanism of the silylation reaction of acetone with Me_3_SiH proposed by the group of Sakata^16*c*^ is analogous to the one in our proposed mechanism. The distance of C3 and Si is 2.04 Å in intermediate 3, indicating that the Si–C bond is nearly formed. Additionally, the C3–H bond distance in intermediate 3 is 1.09 Å, which is longer than that in free *N*-methylindole (1.08 Å), suggesting that the C3–H bond is weakened.

#### 3.1.2 Generation of silylated product and indoline

Following the formation of ion pair 3, there are three possible pathways to yield the silylated product. The most favourable pathway is shown in Fig. 1(A). A second *N*-methylindole molecule acting as a proton-shuttle participates in the reaction, abstracting an H^+^ from the indolium moiety in 4 through the transition state TS_4/5_ to afford intermediate 5. Then, intermediate 5 dissociates into C3-silylated indole 6 and a new ion pair 7. In ion pair 7, indoline is formed by intramolecular hydride migration through the transition state TS_7/8_, giving a stable Lewis adduct 8. Finally, 8 dissociates into free *N*-methylindoline and Lewis acid B(C_6_F_5_)_3_. Similarly, the product *N*-methylindoline can also serves as proton-shuttle to promote the reaction through transition state TS_11/12_ to generate a stable ion pair 13 (path B in Fig. 1(B)). The third pathway is direct H_2_ generation (path C in Fig. 1(B)). The free energy barriers of the rate-determining steps in pathways B and C are 27.9 (TS_9/10_) and 26.8 (TS_13/14_) kcal mol^−1^, respectively, with both values larger than that of the indole-assisted pathway (24.8 kcal mol^−1^, TS_4/5_, relative to 13). Therefore, these two pathways are less favourable than that promoted by *N*-methylindole. Overall, the rate-limiting step for B(C_6_F_5_)_3_-catalyzed C–H silylation of *N*-methylindole with PhSiH_3_ is the proton migration (TS_4/5_) with a free energy barrier of 24.8 kcal mol^−1^ (relative to 13). It's worth mentioning that *N*-methylindole acts as both substrate and proton shuttle in the C–H silylation of *N*-methylindole.

#### 3.1.3 Continuous oxidation of the resulting *N*-methylindoline to *N*-methylindole

**Scheme 3:**
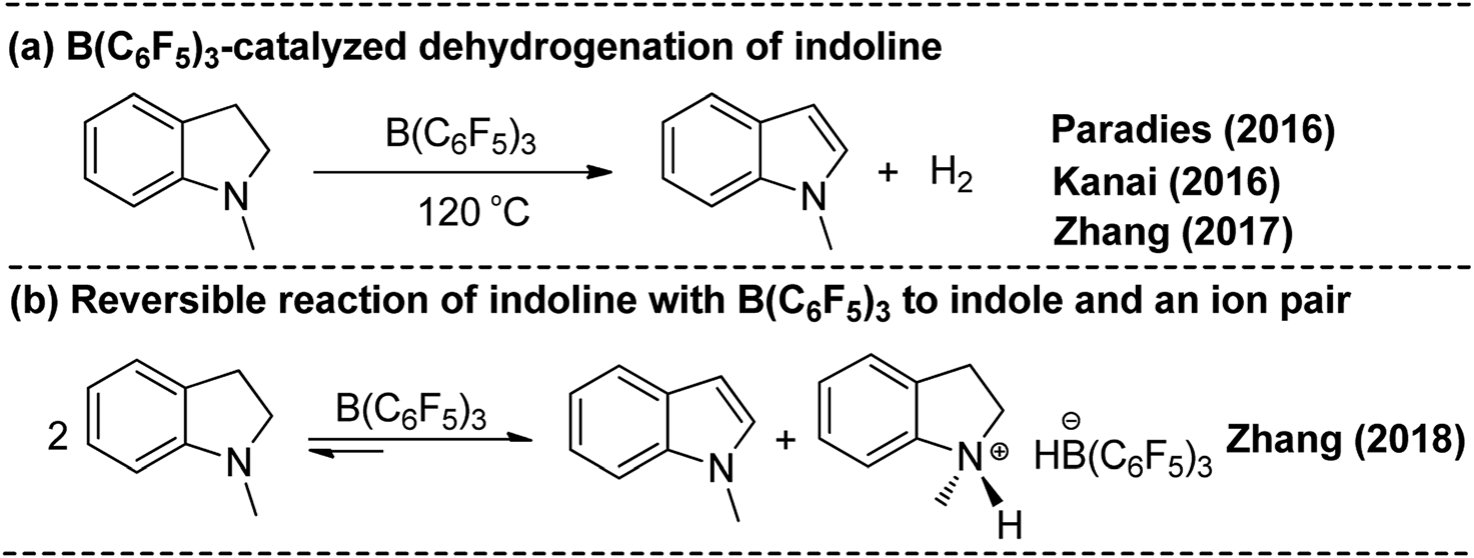
Metal-free B(C_6_F_5_)_3_-catalyzed transformation of *N*-methylindoline to *N*-methylindole ((a) producing indole and H_2_, (b) producing indole and ion pair).

**Fig. 2:**
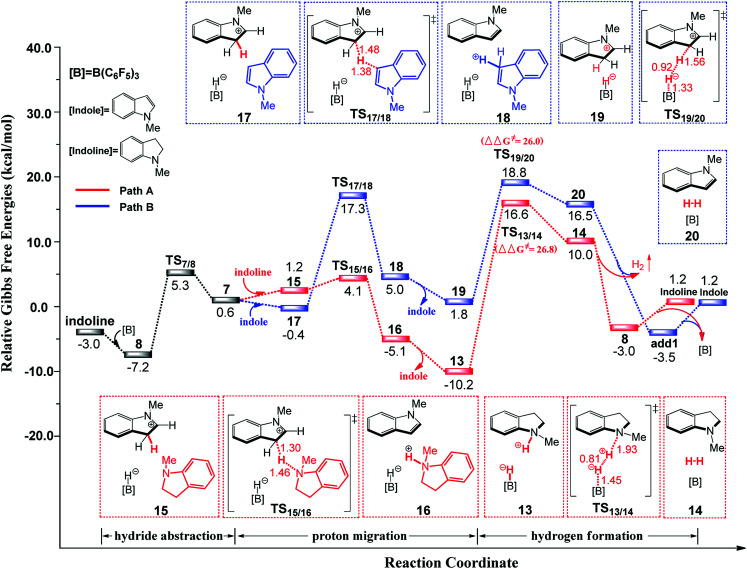
Free-energy profile for B(C_6_F_5_)_3_-catalyzed transformation of indoline to indole, along with the optimized structures of the stationary points. Key bond lengths are given in Å.

Experimentally, *N*-methylindoline was converted back to *N*-methylindole when the reaction temperature is increased to 120 °C.^11^ Similarly, the groups of Paradies and Kanai achieved the same reaction (Scheme 3(a)).^24*a*,*b*^ Furthermore, a recent study by the group of Zhang revealed a reversible reaction of indoline with B(C_6_F_5_)_3_ to yield an ion pair and an indole (Scheme 3(b)).^25^ Using DFT calculations, the group of Paradies proposed a pathway for the B(C_6_F_5_)_3_-catalyzed dehydrogenation of indoline involving hydride abstraction, proton transfer and H_2_ release.^24*a*^ We investigated the oxidation of *N*-methylindoline to *N*-methylindole and found that there are two possible reaction pathways. The Gibbs free energy profiles of these reactions along the reaction pathways are presented in Fig. 2 and the related optimized structures are given in Fig. S3.†

First, B(C_6_F_5_)_3_ selectively abstracts a hydride from the C2 position of *N*-methylindoline through transition state TS_7/8_ to give an ion pair 7 over a low barrier of 12.5 kcal mol^−1^ (as compared to 8). Starting with ion pair 7, two pathways are possible. In path A, a second *N*-methylindoline abstracts a proton from ion pair 7 through transition state TS_15/16_, leading to the formation of intermediate 16. Subsequently, intermediate 16 is dissociated to form *N*-methylindole and a stable ion pair 13 (Δ*G* = −10.2 kcal mol^−1^). This proton abstraction is very facile because a barrier of only 11.3 kcal mol^−1^ must be overcome. Finally, H_2_ is released from the ion pair 13 to regenerate *N*-methylindoline and B(C_6_F_5_)_3_. This step requires the overcoming of a free energy barrier of 26.8 kcal mol^−1^ (relative to ion pair 13). The three elementary steps in path A are analogous with that proposed by the group of Paradies.^24*a*^ Nevertheless, we found a new pathway (path B in Fig. 2) in which *N*-methylindole can also facilitate the reaction through proton abstraction (TS_17/18_, 24.5 kcal mol^−1^) and H_2_ generation (TS_19/20_, 26.0 kcal mol^−1^). Cleanly, the reaction barrier of path B is close to that of path A, indicating that both paths A and B are possible.

It is worth mentioning that the proton abstraction in path A is more facile than that in path B (11.3 kcal mol^−1^*vs.* 24.5 kcal mol^−1^). Therefore, when the reaction occurs at room temperature, it will follow pathway A to give indole and stable ion pair 13, as was found in Zhang's experiments (Scheme 2(B)).^25^ However, when the reaction temperature is increased to 120 °C (Scheme 2(A)), pathways A and B are both possible because their reaction barriers are very close (path A, 26.8 kcal mol; path B, 26.0 kcal mol^−1^). Therefore, our mechanism is in good consistent with the experimental observations. To further confirm the reaction barriers of paths A and B, we used the *w*B97XD functional to study the same reaction. The barriers obtained using the *w*B97XD functional are 22.6 (TS_13/14_) and 21.2 (TS_19/20_) kcal mol^−1^, respectively, in agreement with the values obtained using the M062X functional.

#### 3.1.4 Formation of bis(indol-3-yl)-substituted product

**Scheme 4:**
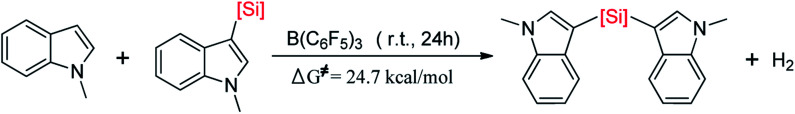
Silylation of *N*-methylindole with the silylated-indole as hydrosilane to yield bis(indol-3-yl)-substituted product.

In Zhang's experiments, the bis(indol-3-yl)-substituted product was obtained when the reaction time was extended to 24 h (Scheme 4).^11^ Therefore, we studied the process of the silylation of *N*-methylindole using silylated-indole as hydrosilane. We found that also in this case, there are four different possible reaction pathways involving indole or indoline acting as a promoter to assist the reaction and direct H_2_ generation, respectively. The optimized structures of all species and the related free energy profiles along the reaction pathway are shown in Fig. S4 and S5 in the ESI.† The *N*-methylindole and *N*-methylindoline-promoted pathways are both favourable pathways in the formation of bis(indol-3-yl)-substituted product, where Δ*G*^≠^ = 24.7 and 25.4 kcal mol^−1^, respectively. Additionally, the free energy barriers for other two pathways are 27.5 or 29.0 kcal mol^−1^, respectively, suggesting that these pathways are less favourable than these promoted by *N*-methylindole or *N*-methylindoline.

### 3.2 C–H silylation of *N*,*N*-dimethylaniline

**Fig. 3:**
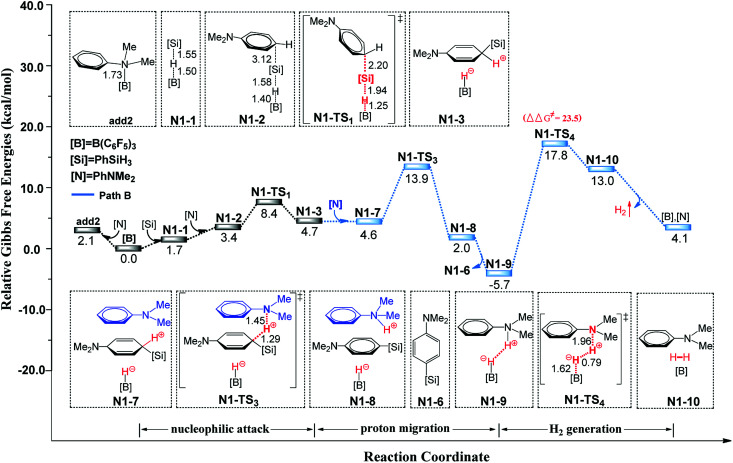
Free-energy profiles for B(C_6_F_5_)_3_-catalyzed dehydrogenative silylation of *N*,*N*-dimethylaniline with PhSiH_3_ (pathway B), along with the optimized structures of the stationary points. Key bond lengths are given in Å.

The second reaction we studied was the B(C_6_F_5_)_3_-catalyzed C–H silylation of *N*,*N*-dimethylaniline. The reaction is started with nucleophilic attack on the B–Si complex by *N*,*N*-dimethylaniline, followed by three different reaction pathways (paths A, B, and C) to generate the silylated product. The free energy profiles and related optimized geometric structures of these pathways are shown in Fig. 3 (path B) and S6–S7 (paths A and C) in the ESI.† In path A, species N1-3 undergoes an proton-hydride recombination to afford the silylated product, identical to the reaction pathway suggested by the group of Hou.^9*b*^ The free energy barrier of path A is 29.1 kcal mol^−1^. However, we found a new pathway (path B) that is more reasonable than path A. In path B, the initial step is proton-abstraction by a second *N*,*N*-dimethylaniline. The subsequent H_2_ generation is the rate-determining step with a free energy barrier of 23.5 kcal mol^−1^. This H_2_ generation transition state is in consistent with the observation by Rieger and co-workers.^26^ The process of path C is similar to that of path B, but the corresponding proton shuttle is the silylated product. The free energy barrier of path C is 27.2 kcal mol^−1^. Among these three pathways, pathway B is more favourable than paths A and C. We recalculated the three pathways using the wb97xd functional and the related reaction barriers were 27.1, 21.9 and 24.3 kcal mol^−1^, respectively. It is clear that the results obtained using the wb97xd and M062X functionals are in good agreement, and thus our calculations are reasonable.

### 3.3 Comparison of C–H and N–H silylation of PhNHMe

Generally, if the aniline bears *para*-phenyl C–H and N–H bonds, both C–H and N–H silylation reactions will occur. Therefore, we chose PhNHMe as the substrate to investigate its cross-dehydrocoupling reaction catalyzed by B(C_6_F_5_)_3_ with PhSiH_3_.

**Fig. 4:**
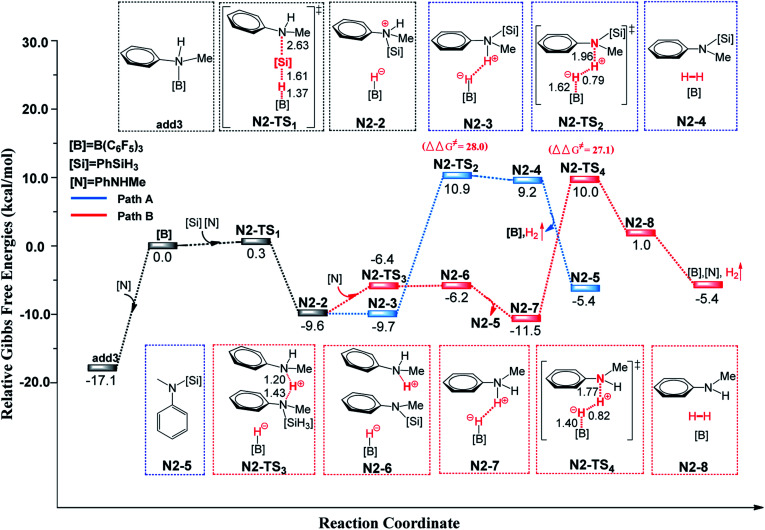
Free-energy profiles for N–H silylation of PhNHMe with PhSiH_3_ catalyzed by B(C_6_F_5_)_3_, along with the optimized structures of the stationary points. Key bond lengths are given in Å.

First, we studied the N–H silylation of PhNHMe. One possible pathway is comparable to that proposed by Stephan and co-workers^14*b*^ (see Fig. 4(A) and S8, ESI†), including a nucleophilic attack and H_2_ liberation. The second step is the rate-limiting step with the free energy barrier of 28.0 kcal mol^−1^ (25.9 kcal mol^−1^ according to the wb97xd functional calculations). However, we also obtained an alternative pathway in which a second PhNHMe served as a proton shuttle to assist the proton migration (the related free energy profile and geometries of the structures are shown in Fig. 4(B) and S8, ESI†). Our proposed pathway includes a nucleophilic attack (Δ*G*^≠^ = 17.4 kcal mol^−1^), proton abstraction (Δ*G*^≠^ = 10.7 kcal mol^−1^) and H_2_ generation (rate-limiting step, Δ*G*^≠^ = 27.1 kcal mol^−1^, 25.0 kcal mol^−1^ for the wb97xd functional calculations). Therefore, both of the pathways are possible. The two pathways of N–H silylation of *N*-methylaniline are both different from the mechanism of N–H silylation of indole proposed by the group of Paradies.^14*c*^

Next, we studied the C–H silylation of PhNHMe and found that this reaction is comparable to that of *N*,*N*-dimethylaniline. The most favourable pathway is identical to path B in C–H silylation of *N*,*N*-dimethylaniline and requires the overcoming of a free energy barrier of 36.6 kcal mol^−1^ (the related free energy profile and geometries of the structures are shown in Fig. S9 and S10, ESI†). It is clear that N–H silylation of PhNHMe is more facile than the C–H silylation. The transformation from C–H to C–Si bonds and N–H to N–Si bonds are endothermic by 43.8 and 20.2 kcal mol^−1^, respectively. So the N–H silylated product is more stable than the C–H silylated product, thereby lowering the corresponding reaction barrier. Since PhNHMe was not used in the N–H silylation experiments, we choose PhNH_2_ and Ph_2_NH that were used as substrates in experiments to study the related N–H silylation reactions with Ph_2_MeSiH in dichloromethane. The related free energy barriers of the rate-determining steps are 22.6 and 16.9 kcal mol^−1^, respectively, which are reasonable barriers for experimental conditions.^14*c*^

### 3.4 Other possible Lewis acids and substrates

The group of Zhang reported that Lewis acid Al(C_6_F_5_)_3_ can also catalyzed the C–H silylation of *N*-methylindole with Ph_2_SiH_2_.^12^ We studied the mechanisms of the reaction. There are three possible reaction pathways and the related free energy barriers are 38.0, 26.5 and 31.8, kcal mol^−1^ (Table 1), respectively. The *N*-methylindoline promoted pathway (path B) is most favourable for C–H silylation of *N*-methylindole catalyzed by Al(C_6_F_5_)_3_, which is different from that catalyzed by B(C_6_F_5_)_3_. Furthermore, we test other two Lewis acids (Ga(C_6_F_5_)_3_ and B(2,6-Cl_2_C_6_H_3_)(*p*-HC_6_F_4_)_2_)^27^ to catalyze the same reaction like Al(C_6_F_5_)_3_. The most favorable pathways catalyzed by the two Lewis acids are same with that catalyzed by Al(C_6_F_5_)_3_. The related free energy barriers of the rate-determining steps are 21.7 and 21.8 kcal mol^−1^, respectively. The geometric structures of the related transition states are shown Fig. S11 in the ESI.† It seems that all of these Lewis acids could promote the silylation of *N*-methylindole.

**Table 1:** Free energy barriers of the rate-determining steps of C–H silylation of *N*-methylindole with Ph_2_SiH_2_ catalyzed by different Lewis acids (kcal mol^−1^)

	B(C_6_F_5_)_3_	Al(C_6_F_5_)_3_	Ga(C_6_F_5_)_3_	B(2,6-Cl_2_C_6_H_3_)(*p*-HC_6_F_4_)_2_
Barriers (path A)	24.8	38.0	35.4	23.8
Barriers (path B)	26.8	26.5	21.7	21.8
Barriers (path C)	27.9	31.8	35.4	25.3

The substrates in the silylation reactions are thiophenes, pyridines, indoles and anilines in experiments. We want to extend the reaction to other types of reaction substrates. So we selected six other heterocyclic compounds to study their C–H silylation reaction with PhSiH_3_ catalyzed by B(C_6_F_5_)_3_. These substrates are shown in Table 2. The most favourable pathways for the C–H silylation of these substrates are identical to path C in C–H silylation of *N*-methylindole. The related free energy barriers of the rate-determining steps are 21.3, 17.9, 16.0, 19.8, 51.2 and 63.7 kcal mol^−1^ (Table 2), respectively. The geometric structures of the related transition states are shown Fig. S12 in the ESI.† These results suggest that entries 1–4 would undergo C–H silylation reactions under moderate conditions while entries 6 and 7 can't.

**Table 2:** Free energy barriers of the C–H silylation of a series of substrates (kcal mol^−1^)

Substrate		Δ*G* (TS)	Substrate		Δ*G* (TS)	Substrate		Δ*G* (TS)
1	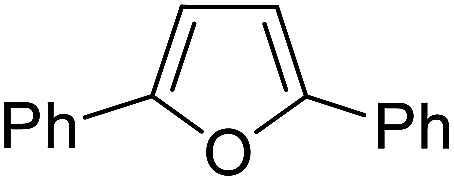	21.3	2	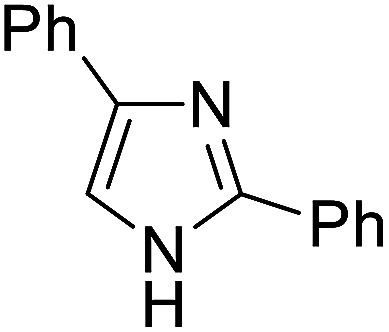	17.9	3	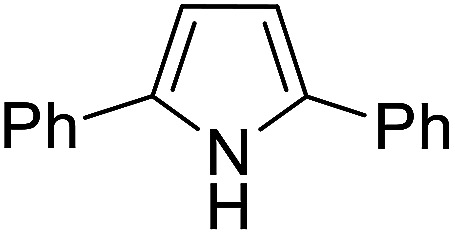	16.0
4	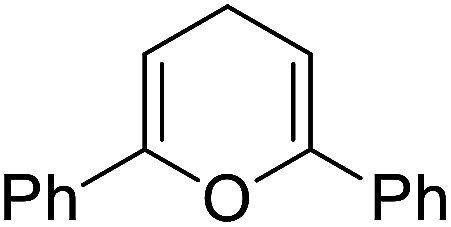	19.8	5	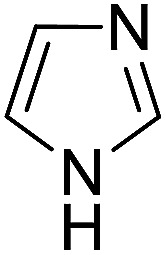	51.2	6	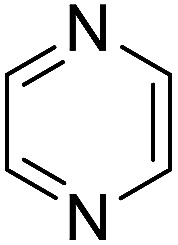	63.7

Similarly, we chose six other different types of substrates (entries 1–6 in Table 3) to study their N–H silylation reaction with PhSiH_3_ catalyzed by B(C_6_F_5_)_3_. The related free energy barriers of the rate-determining steps are 17.7, 27.7, 28.7, 33.3, 32.6 and 36.4 kcal mol^−1^ (Table 3), respectively. The geometric structures of the related transition states are shown Fig. S13 in the ESI.† The results suggest that entries 1–3 would undergo N–H silylation reaction while entries 4–6 would not undergo the same reaction. Entries 4–6 can form stable Lewis adducts with Lewis acid B(C_6_F_5_)_3_. The free energies of their Lewis adducts are 22.3, 26.3 and 24.9 kcal mol^−1^ lower than the related free Lewis acids and bases. These stable Lewis adducts inhibit the corresponding N–H silylation reaction.

**Table 3:** Free energy barriers of the N–H silylation of a series of substrates (kcal mol^−1^)

Substrate		Δ*G* (TS)	Substrate		Δ*G* (TS)	Substrate		Δ*G* (TS)
1	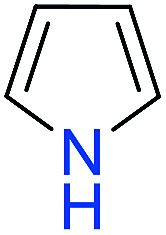	17.7	2	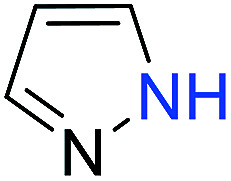	27.7	3	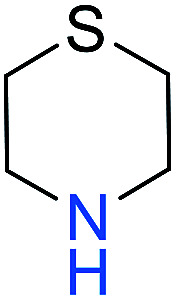	28.7
4	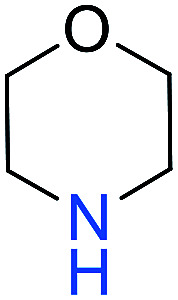	33.3	5	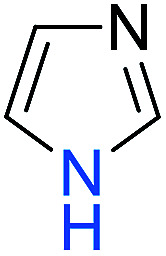	32.6	6	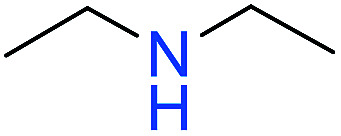	36.4

## 4 Conclusions

**Scheme 5:**
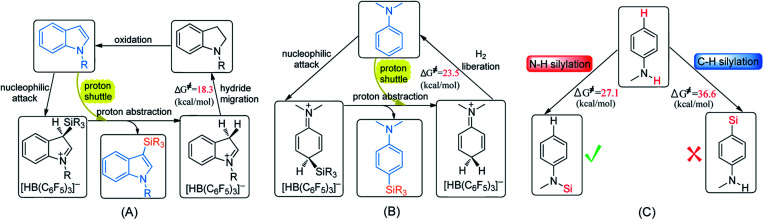
Proposed mechanisms for (A) C–H silylation of *N*-methylindole, (B) C–H silylation of *N*,*N*-dimethylaniline and (C) selectivity of silylation of *N*-methylaniline.

Transition-metal-catalyzed C–H silylation of arenes is an attractive synthetic approach since it was known as efficient, atom-economical and superior selectivity. The protocol of B(C_6_F_5_)_3_ catalytic C–H and N–H silylation of aromatic compounds features environmental conditions, high regioselectivity, and no requirement for removing residual metal catalysts. Efforts have been made to deepen the understanding of the reaction mechanisms of these reactions to enhance the activity of the catalysts. So we performed detailed density functional theory calculations on the mechanism of the dehydrogenative silylation of *N*-methylindole, *N*,*N*-dimethylaniline and *N*-methylaniline. The main conclusions are summarized as follows:

(1) The most favourable pathways for the silylation of *N*-methylindole and *N*,*N*-dimethylaniline are similar except for the last step (Scheme 5A and B). The first two steps are the same: nucleophilic attack and proton abstraction, and the last step is hydride migration for *N*-methylindole and H_2_ liberation for *N*,*N*-dimethylaniline. In the two reactions, aniline and indole not only serve as Lewis bases to attack the B–Si complex but also as a Brønsted base to assist proton transfer, leading to the lowering of the reaction barrier.

(2) For the conversion of the resulting indoline to indole, we found two compete pathways where indoline and indole both can act as proton-shuttle to facilitate the reaction. Our proposed mechanism can explain the experimental observations that an ion pair was formed at room temperature while *N*-methylindole and H_2_ was generated at 120 °C.

(3) With respect to the dehydrogenative silylation of PhNHMe that bears both N–H and *para*-phenyl C–H bonds, the N–H silylated product is more stable than the C–H silylated product, so N–H silylation of PhNHMe is more favourable than the C–H silylation (Scheme 5C). N–H silylation of PhNHMe includes nucleophilic attack, proton abstraction and H_2_ liberation.

(4) The C–H silylation of *N*-methylindole catalyzed by Al(C_6_F_5_)_3_ follows the pathway that promoted by *N*-methylindoline, which is different from that catalyzed by B(C_6_F_5_)_3_. Lewis acids (Ga(C_6_F_5_)_3_, and B(2,6-Cl_2_C_6_H_3_)(*p*-HC_6_F_4_)_2_) can also catalyze the same reaction like Al(C_6_F_5_)_3_. Moreover, we identified four other substrates that would undergo C–H silylation and three other substrates that would undergo N–H silylation with hydrosilanes using B(C_6_F_5_)_3_ as the catalyst.

Of course, our designed reactions need to be examined by experiments. We anticipate this investigation can be helpful for obtaining other metal-free catalysts to promote silylation of other substrates.

## Conflicts of interest

The authors declare no competing financial interests.

## Supplementary Material

RA-009-C9RA07985H-s001
