# Supplementary material for: Comparative DFT study of metal-free Lewis acid-catalyzed C–H and N–H silylation of (hetero)arenes: mechanistic studies and expansion of catalyst and substrate scope
Source: RSC Adv. 2019 Nov 19;9(64):37675–85. doi: 10.1039/c9ra07985h (PMC9075773; doi:10.1039/c9ra07985h)
Supplement: RA-009-C9RA07985H-s001 [file RA-009-C9RA07985H-s001.pdf]

Electronic Supplementary Material (ESI) for RSC Advances.
This journal is © The Royal Society of Chemistry 2019

Supporting Information

Comparative DFT Study of Metal-free Lewis acid-catalyzed C-H and N-H Silylation of (hetero)arenes: Mechanistic Studies and Expansion of Catalyst and Substrate Scope

*Pan Du[a], Jiyang Zhao[b]**

[a]School of Life Science and Chemistry, Jiangsu Second Normal University, Nanjing 210013, People's Republic of China.

[b] Nanjing Key Laboratory of Advanced Functional Materials,

School of Environmental Science, Nanjing Xiaozhuang University, Nanjing 211171, People's Republic of China.

jyzhao1981@163.com

Table of Contents

Figure S1. Optimized intermediates and transition states for $B(C_6F_5)_3$-catalyzed C-H silylation of N-methylindole with $PhSiH_3$……………………………………...…..…s4

Figure S2. Optimized intermediates and transition states for $B(C_6F_5)_3$-catalyzed C-H silylation of N-methylindole with $PhSiH_3$ (the other two pathways)……………...…s5

Figure S3. Optimized intermediates and transition states for $B(C_6F_5)_3$-catalyzed transformation of indoline to indole……………………………………..………...s6

Figure S4. Free-energy profiles for the generation of bis(indol-3-yl)-substituted product catalyzed by $B(C_6F_5)_3$ (pathways A, B and C)…………...……..……...…s8

Figure S5. Optimized intermediates and transition states for the generation of bis(indol-3-yl)-substituted product catalyzed by $B(C_6F_5)_3$……………...…..………...……..s9

Figure S6. Free-energy profiles for $B(C_6F_5)_3$-catalyzed dehydrosilylation of N,N-dimethylaniline with $PhSiH_3$ (pathways A and C)……..………...........……....s10

Figure S7. Optimized intermediates and transition states for $B(C_6F_5)_3$-catalyzed dehydrosilylation of N,N-dimethylaniline with $PhSiH_3$………………...………..s11

Figure S8. Optimized intermediates and transition states for N-H silylation of PhNHMe with $PhSiH_3$ catalyzed by $B(C_6F_5)_3$…………………………………...…s12

Figure S9. Free-energy profiles for C-H silylation of PhNHMe with $PhSiH_3$ catalyzed by $B(C_6F_5)_3$………………………………………………………………….s13

Figure S10. Optimized intermediates and transition states for C-H silylation of PhNHMe with $PhSiH_3$ catalyzed by $B(C_6F_5)_3$…………………………...…….s14

Figure S11. Optimized transition states for C-H silylation of indole with $PhSiH_3$

catalyzed by another Lewis acids (Al(C_6F_5)$_3$, Ga(C_6F_5)$_3$ and B(2,6-$Cl_2C_6H_3$)(p-HC_6F_4)$_2$)…………………………………………………………………....s15

Figure S12. Optimized transition states for C-H silylation of our selected six substrates with $PhSiH_3$ catalyzed by B(C_6F_5)$_3$. ……………………………………………..…….s16

Figure S13. Optimized transition states for N-H silylation of our selected six substrates with $PhSiH_3$ catalyzed by B(C_6F_5)$_3$. ………………………..…....................…....s17

Table S1. Corrected free energies of all species……………………………....……..s18

Table S2. Imaginary frequencies of all transition states. ………………..…..……….s20

Table S3. Cartesian coordinates of all species………………………..…………..…s21

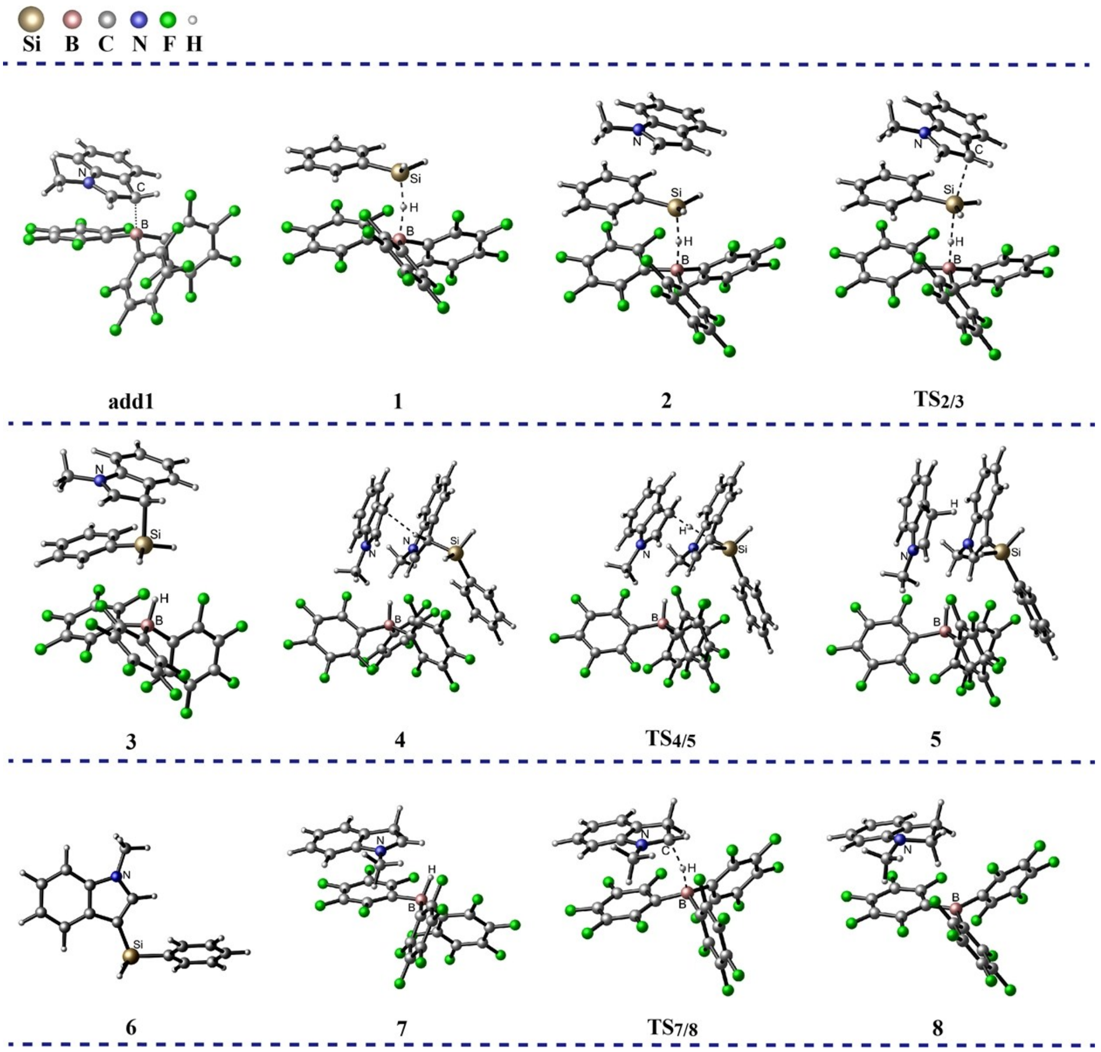

Figure S1. Optimized intermediates and transition states for $B(C_6F_5)_3$-catalyzed C-H silylation of N-methylindole with $PhSiH_3$ (Path A).

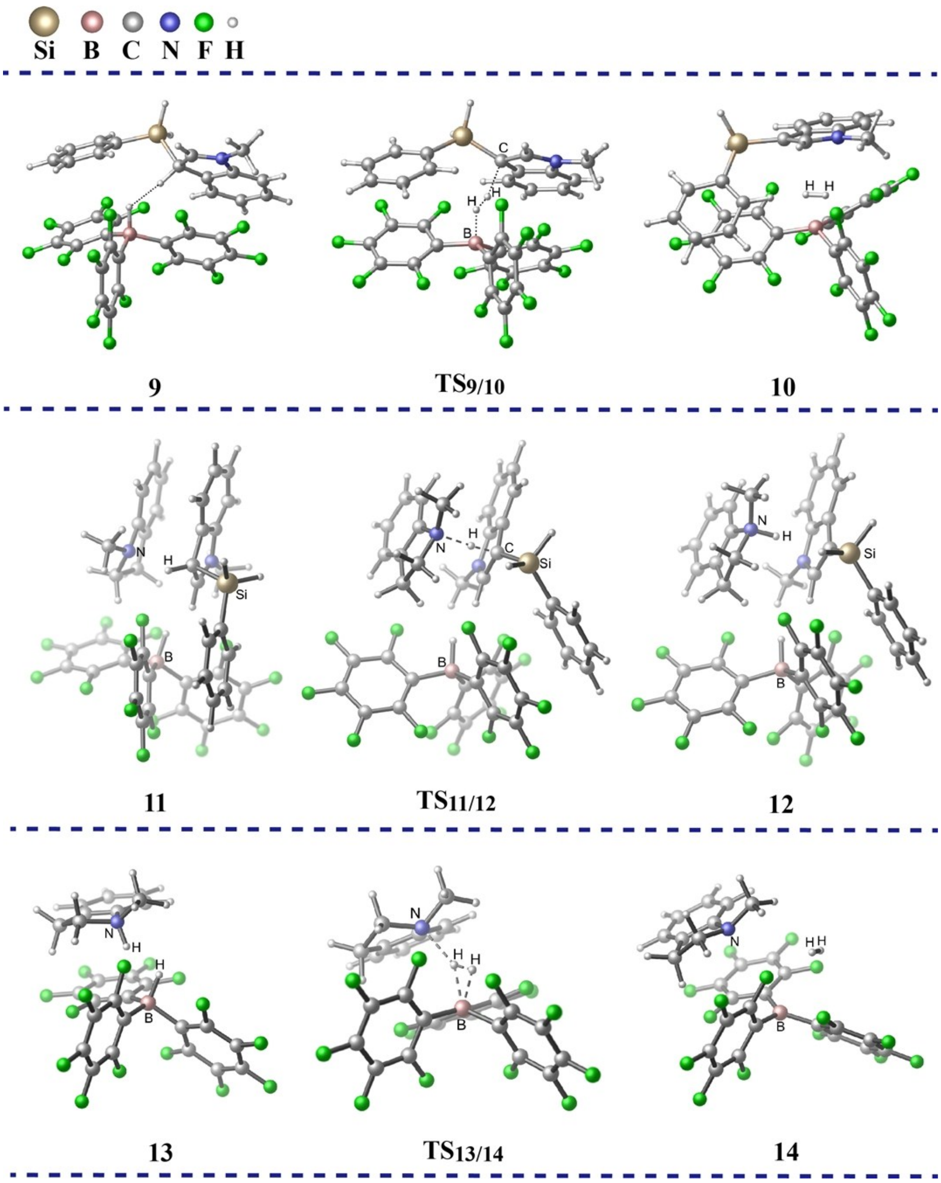

Figure S2. Optimized intermediates and transition states for $B(C_6F_5)_3$-catalyzed C-H silylation of N-methylindole with $PhSiH_3$ (Paths B and C).

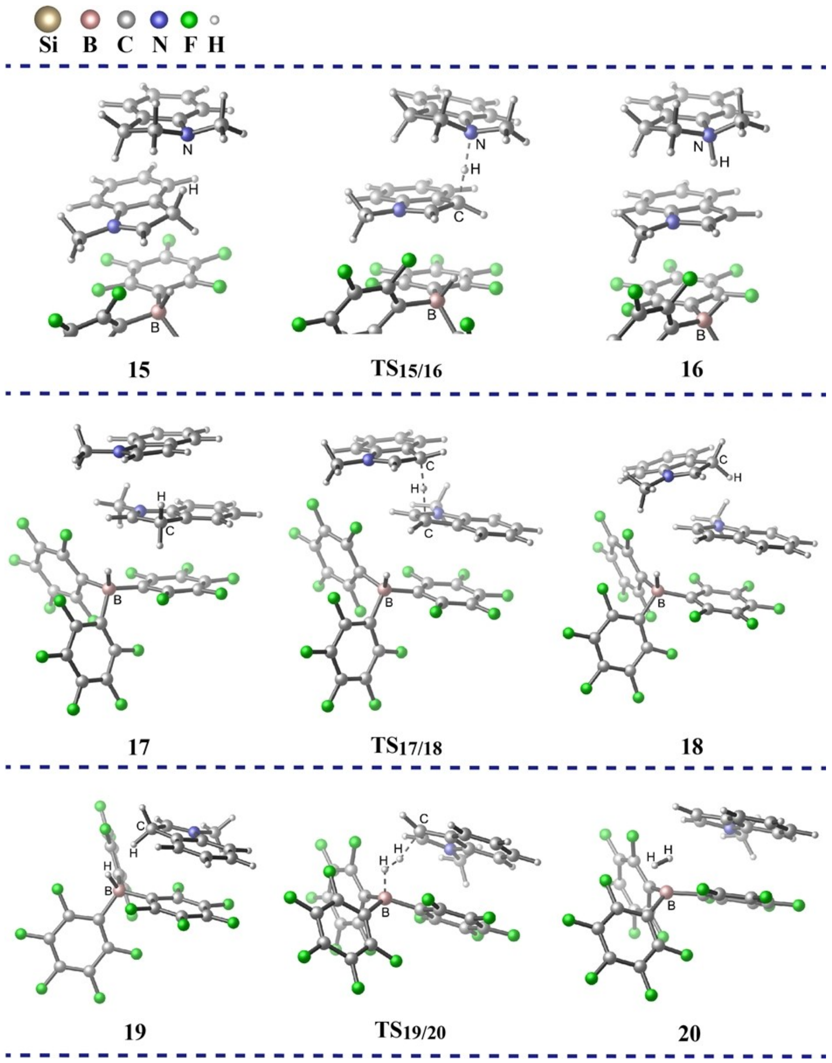

Figure S3. Optimized intermediates and transition states for $B(C_6F_5)_3$-catalyzed transformation of indoline to indole.

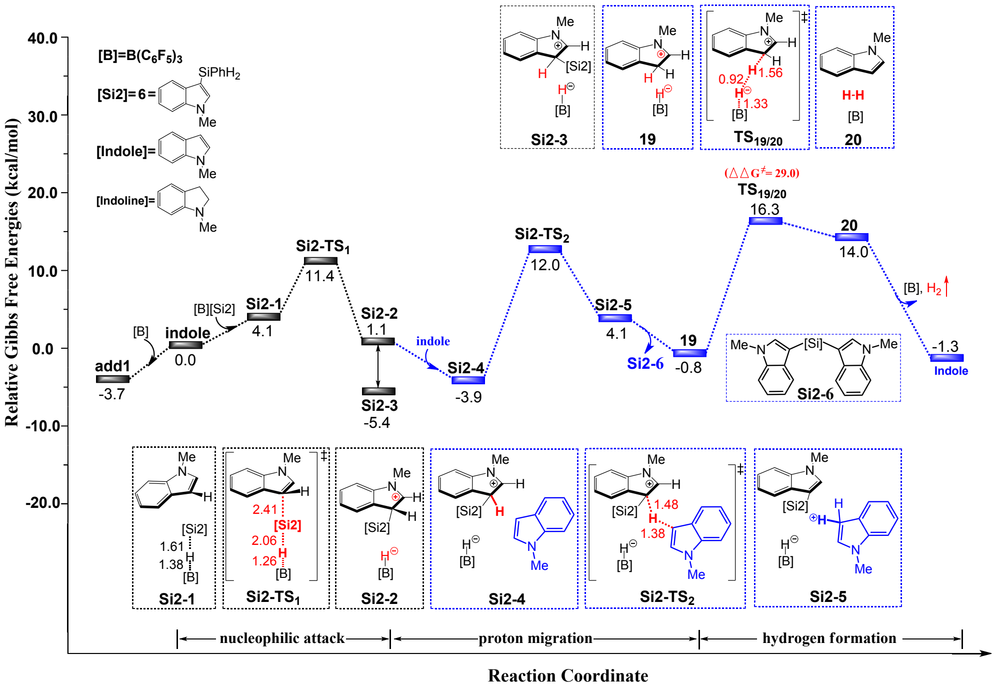
[B]=B(C6F5)3
[Si2]=6=
[Indole]=
[Indoline]=
Relative Gibbs Free Energies (kcal/mol)
add1
-3.7
indole
0.0
Si2-1
4.1
Si2-TS1
11.4
Si2-2
1.1
Si2-3
-5.4
Si2-4
-3.9
Si2-TS2
12.0
Si2-5
4.1
Si2-6
19
-0.8
(△△G≠= 29.0)
TS19/20
16.3
20
14.0
[B], H2
-1.3
Indole
nucleophilic attack
proton migration
hydrogen formation
Reaction Coordinate

(A)

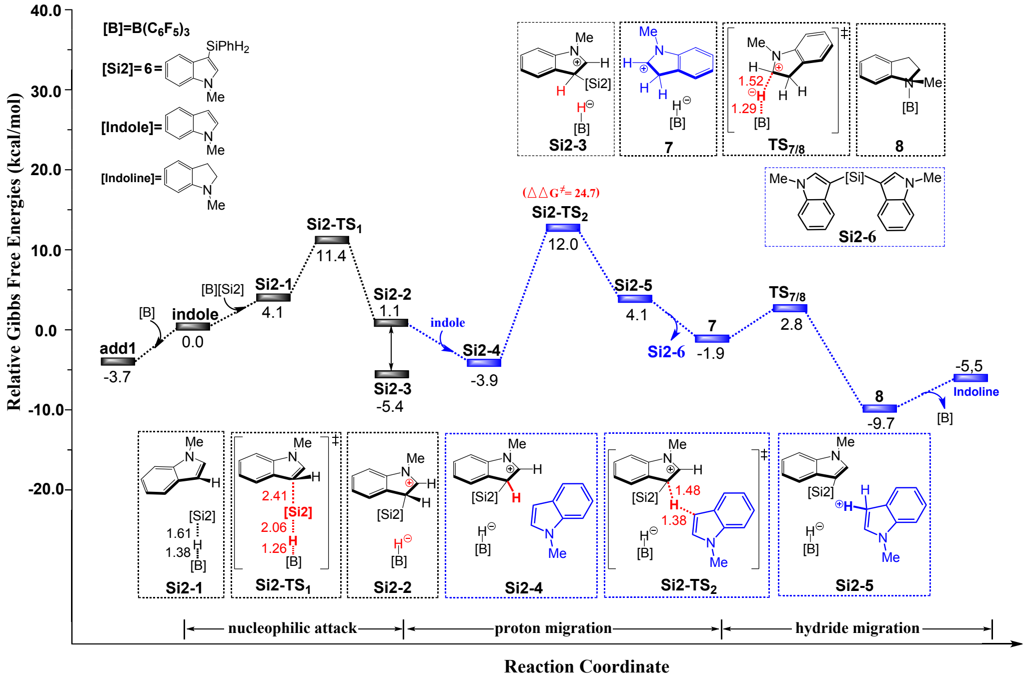
[B]=B(C6F5)3
[Si2]=6=
[Indole]=
[Indoline]=
Relative Gibbs Free Energies (kcal/mol)
add1
-3.7
indole
0.0
Si2-1
4.1
Si2-TS1
11.4
Si2-2
1.1
Si2-3
-5.4
Si2-4
-3.9
(△△G≠= 24.7)
Si2-TS2
12.0
Si2-5
4.1
Si2-6
7
-1.9
TS7/8
2.8
8
-9.7
-5.5
Indoline
nucleophilic attack
proton migration
hydride migration
Reaction Coordinate

(B)

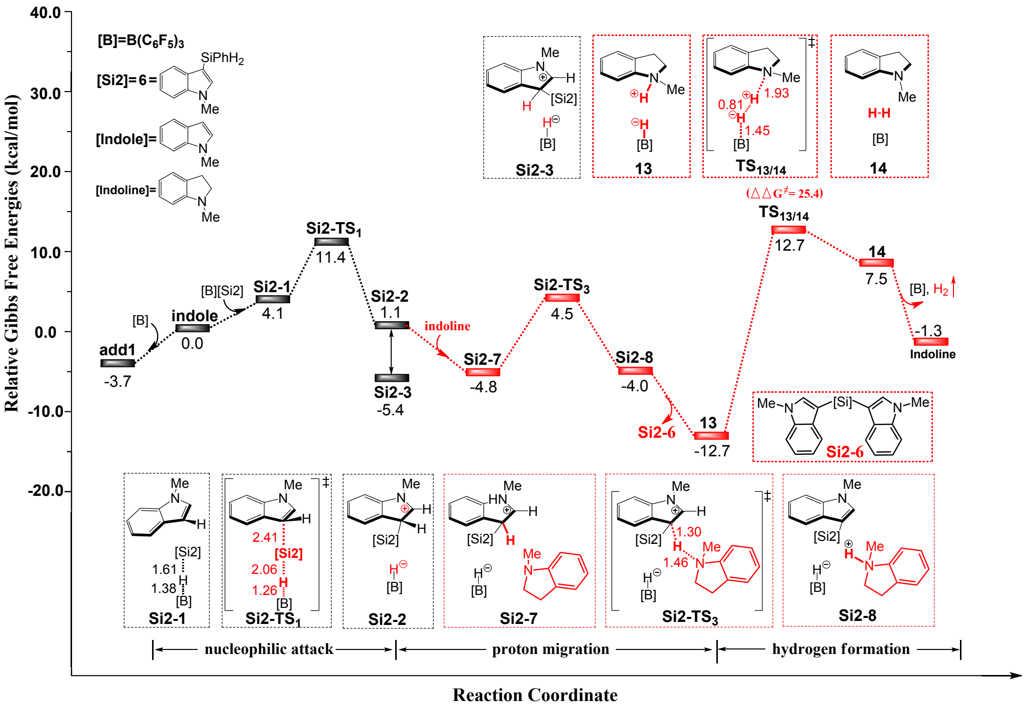

(C)

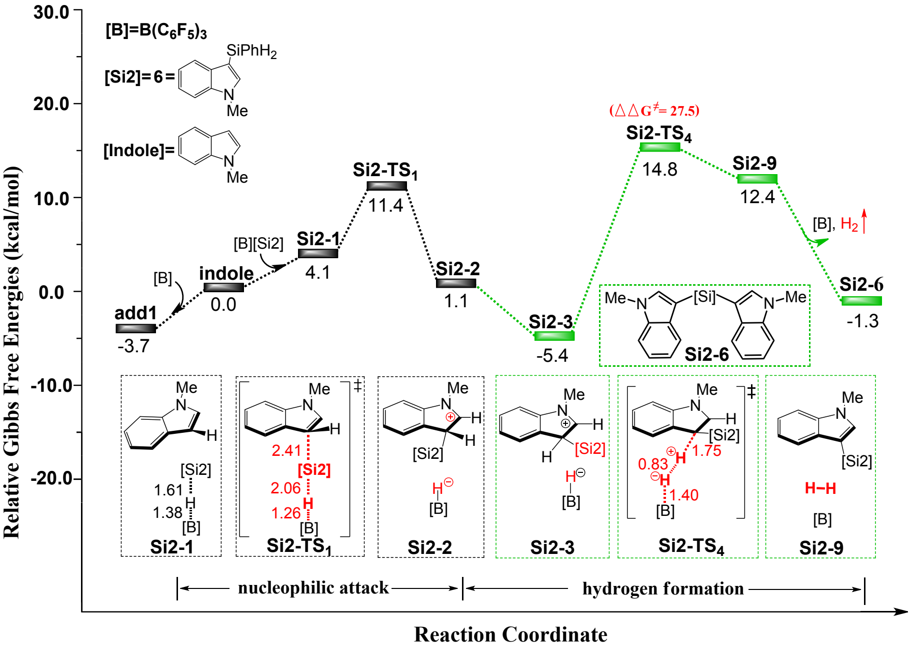

(D)

Figure S4. Free-energy profiles for the generation of bis(indol-3-yl)-substituted product catalyzed by $B(C_6F_5)_3$ (pathways A, B, C and D), along with the optimized structures of the stationary points. Key bond lengths are given in Å

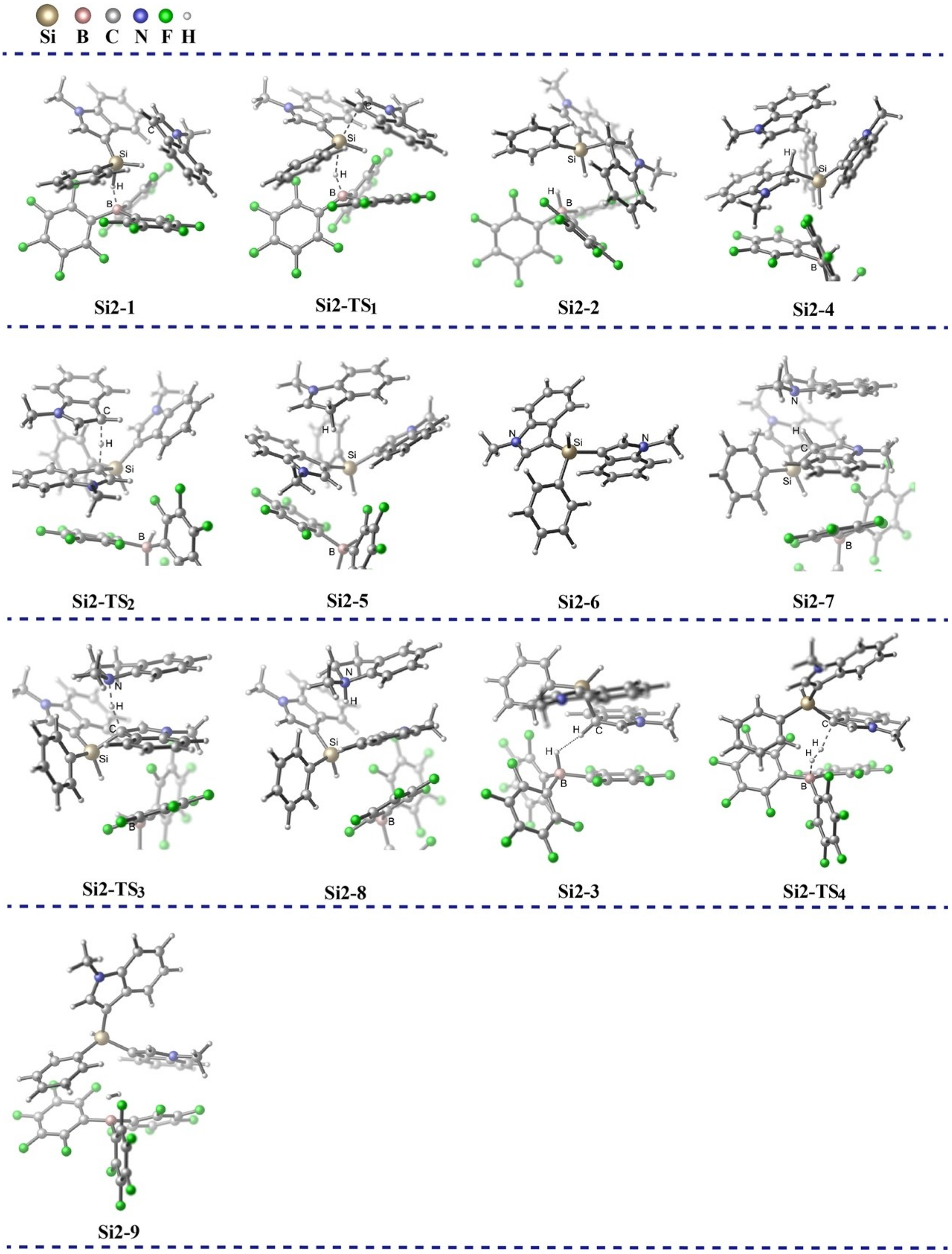

Figure S5. Optimized intermediates and transition states for the generation of bis(indol-3-yl)-substituted product catalyzed by $B(C_6F_5)_3$.

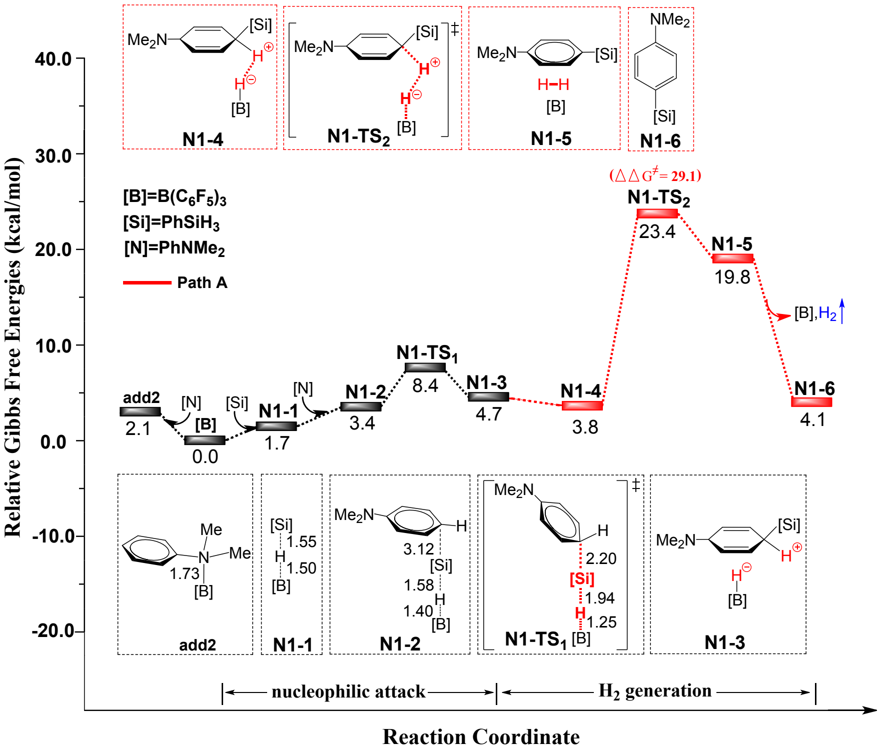

(A)

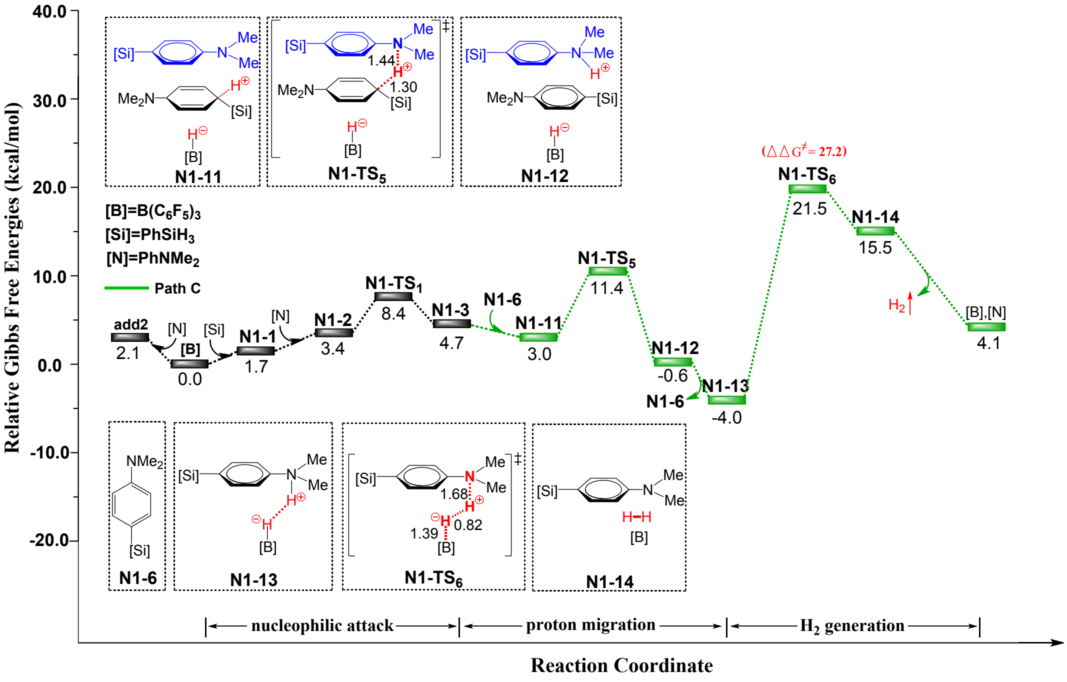

(C)

Figure S6. Free-energy profiles for $B(C_6F_5)_3$-catalyzed dehydrosilylation of N,N-dimethylaniline with $PhSiH_3$ (pathways A and C), along with the optimized structures of the stationary points. Key bond lengths are given in Å.

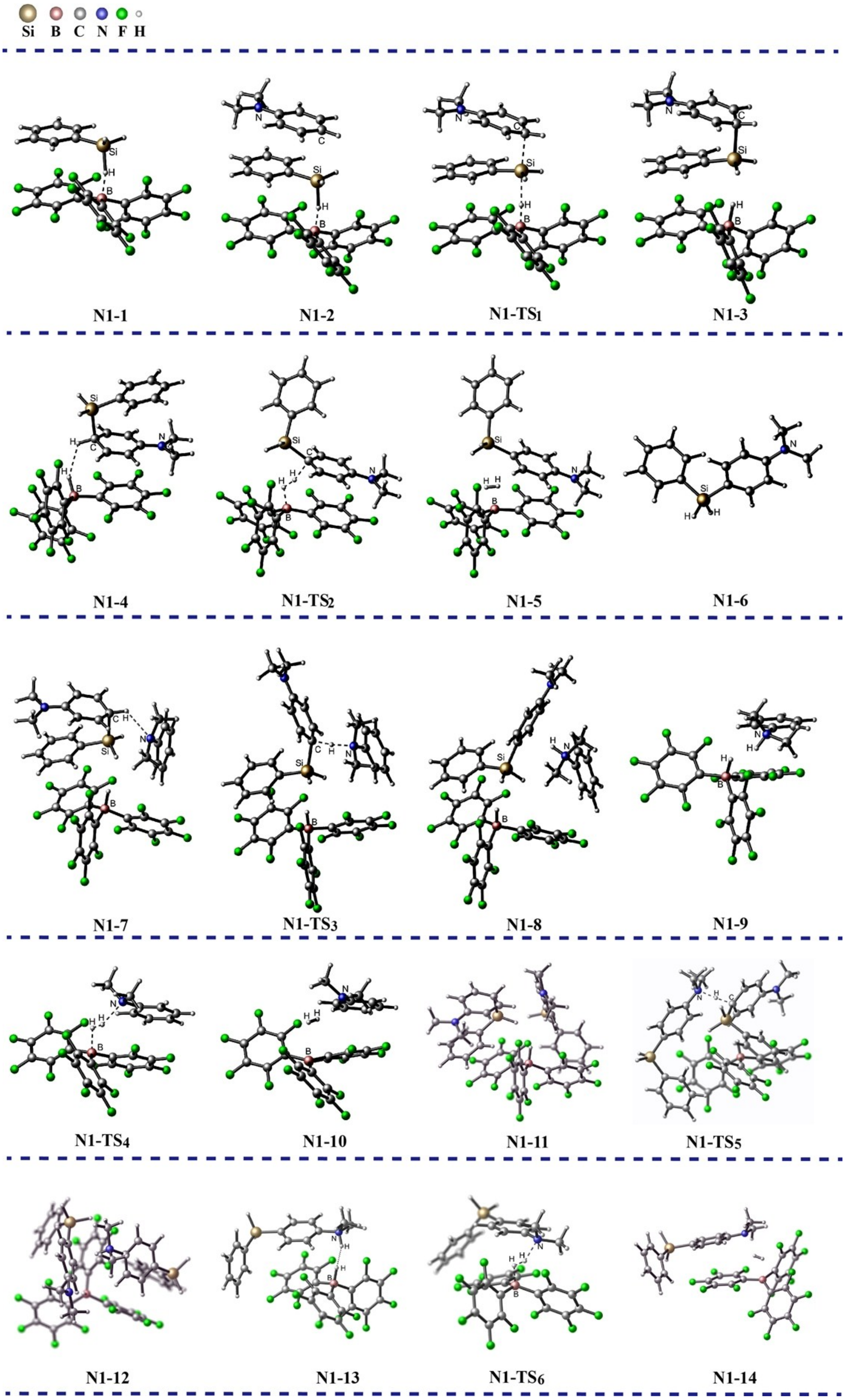

Figure S7. Optimized intermediates and transition states for $B(C_6F_5)_3$-catalyzed dehydrosilylation of N,N-dimethylaniline with $PhSiH_3$.

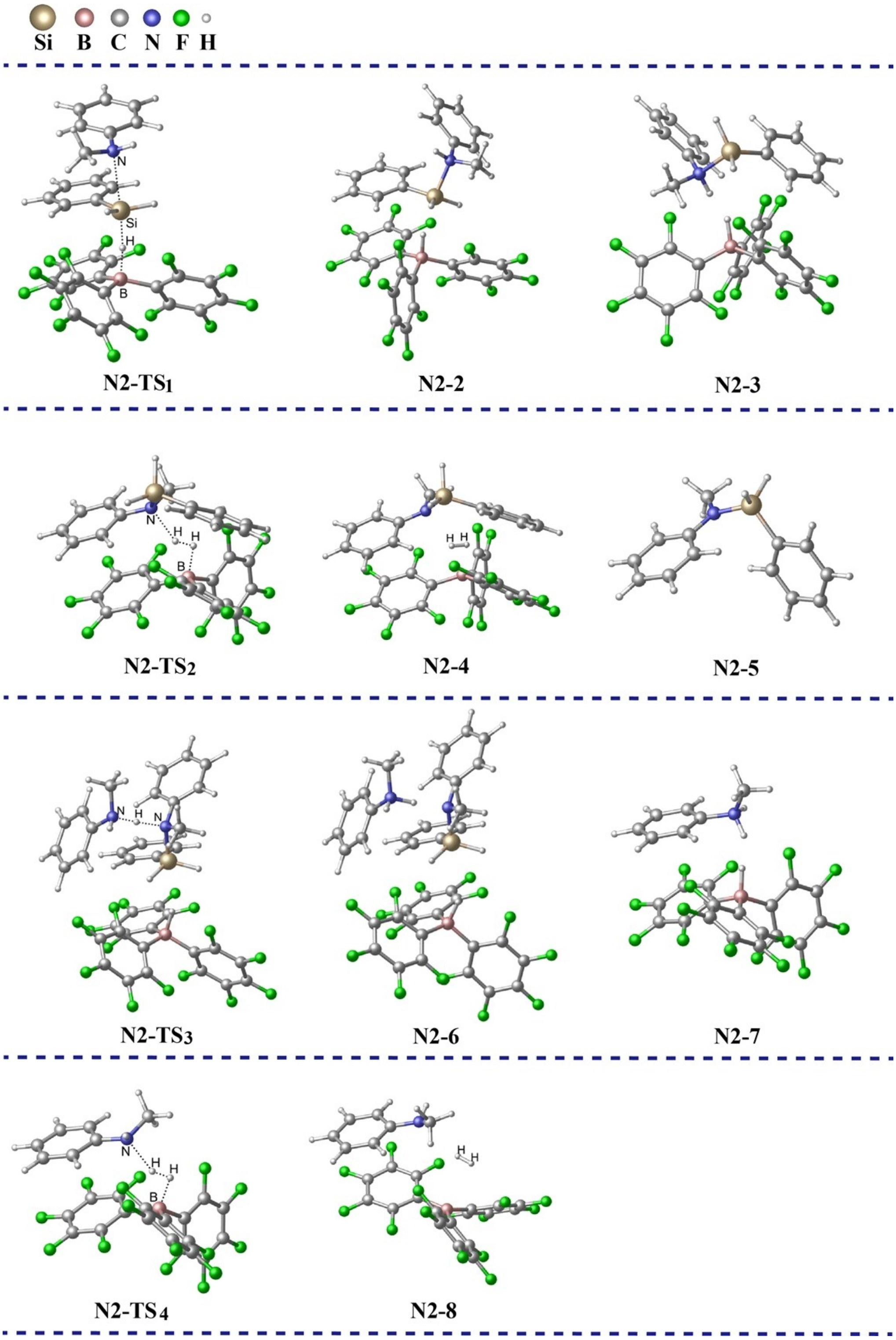

Figure S8. Optimized intermediates and transition states for N-H silylation of PhNHMe with $PhSiH_3$ catalyzed by $B(C_6F_5)_3$.

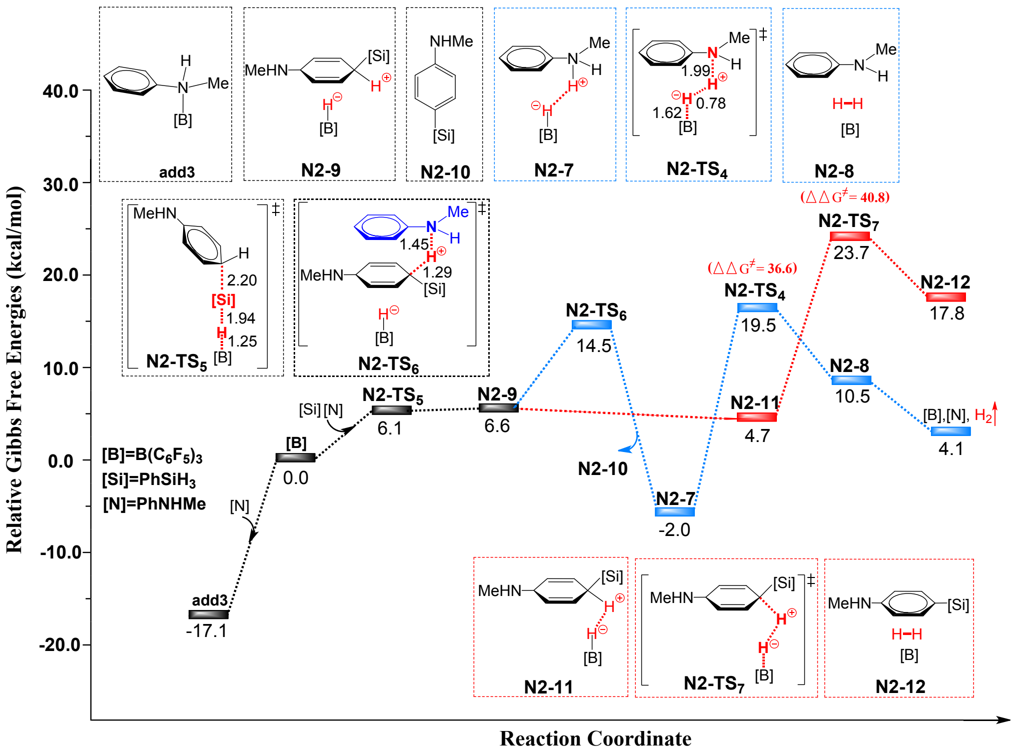

Figure S9. Free-energy profiles for C-H silylation of PhNHMe with $PhSiH_3$ catalyzed by $B(C_6F_5)_3$, along with the optimized structures of the stationary points. Key bond lengths are given in Å.

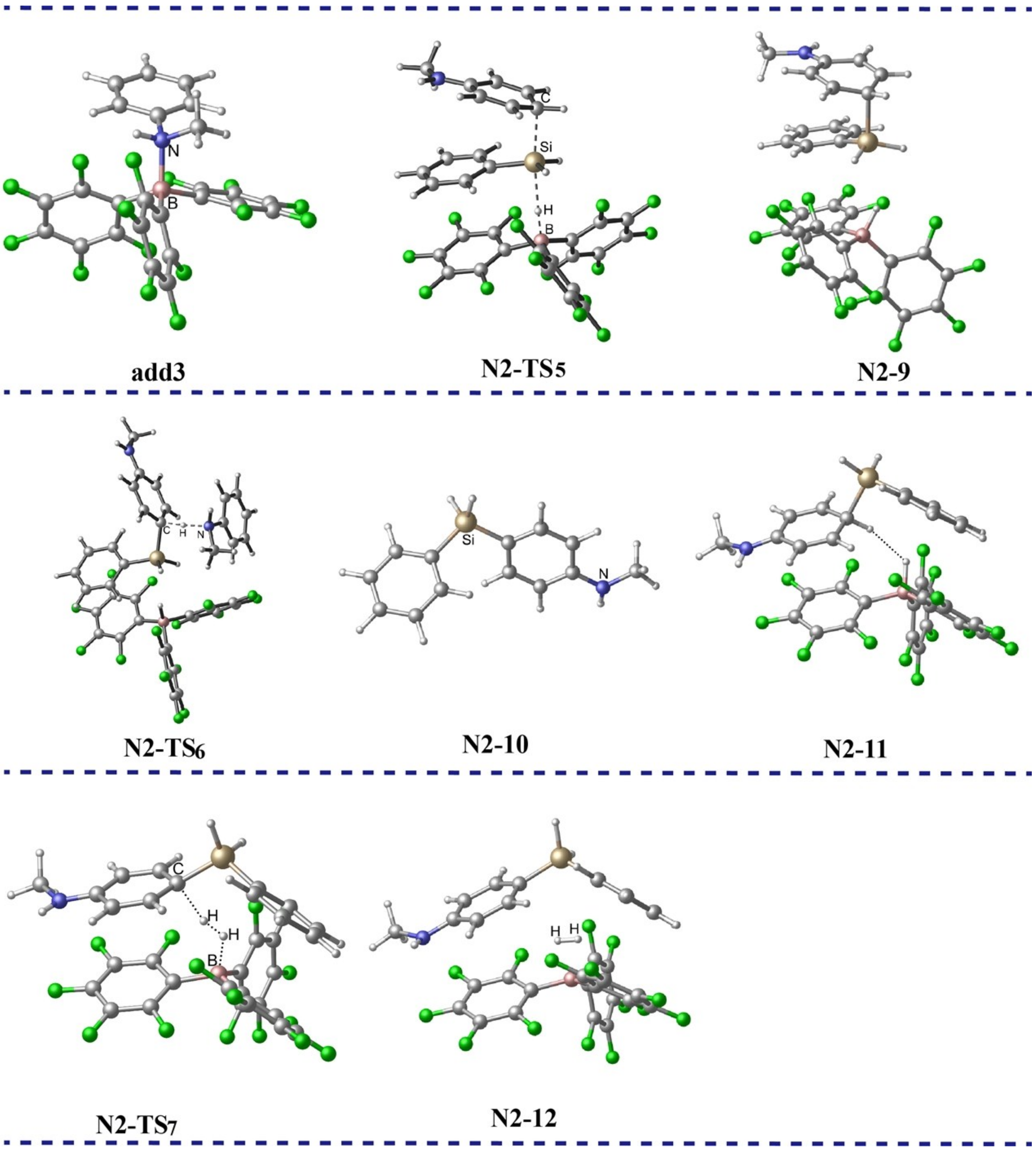

Figure S10. Optimized intermediates and transition states for C-H silylation of PhNHMe with $PhSiH_3$ catalyzed by $B(C_6F_5)_3$.

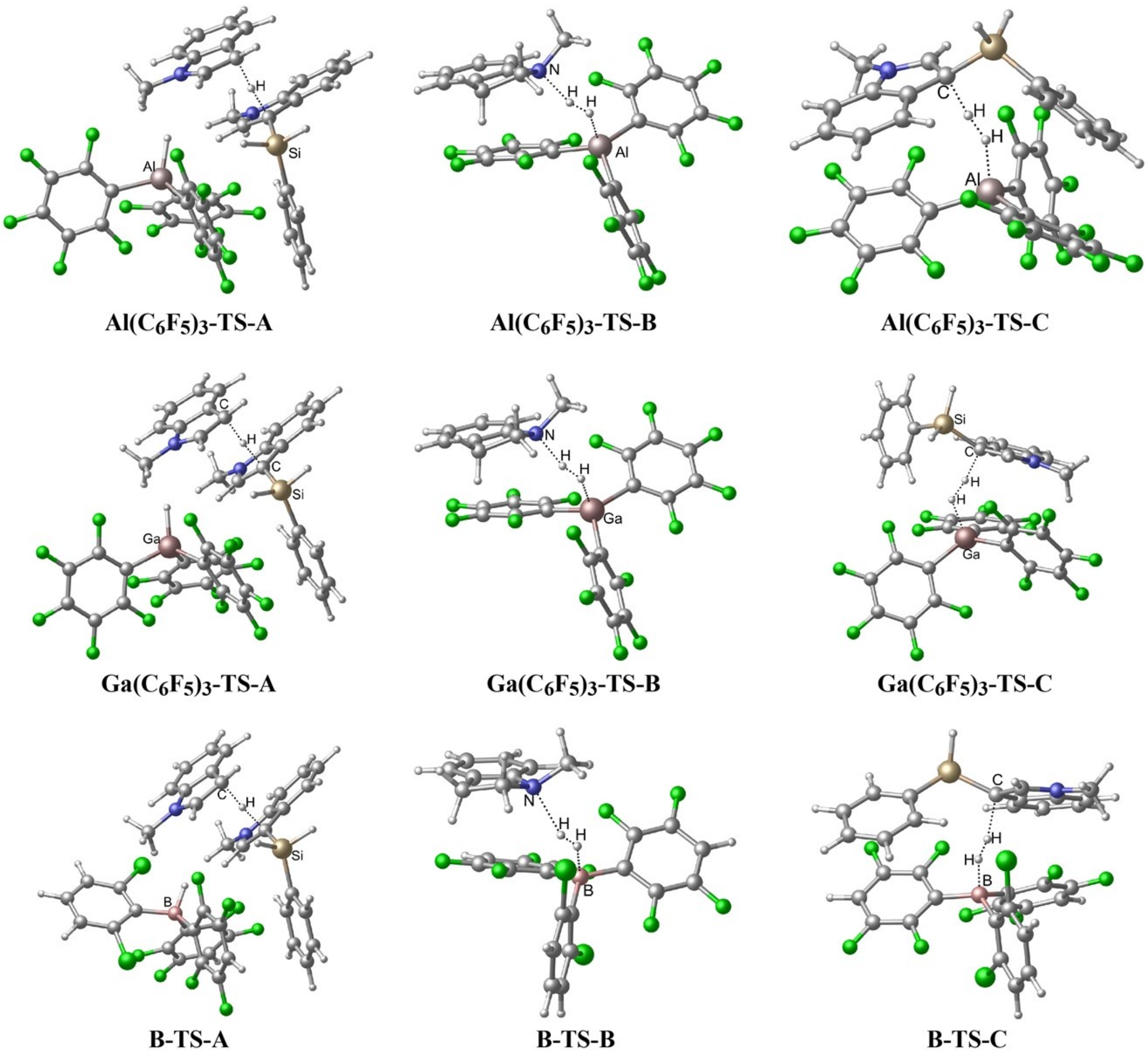

Fig S11. Optimized transition states for C-H silylation of indole with $PhSiH_3$ catalyzed by another Lewis acids ($Al(C_6F_5)_3$, $Ga(C_6F_5)_3$ and $B(2,6\text{-}Cl_2C_6H_3)(p\text{-}HC_6F_4)_2$).

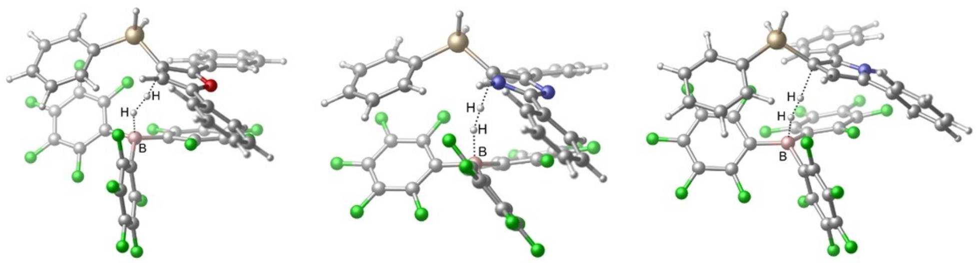

CH-a-TS **(substrate 4)** **CH-b-TS** **(substrate 9)** **CH-c-TS** **(substrate 15)**

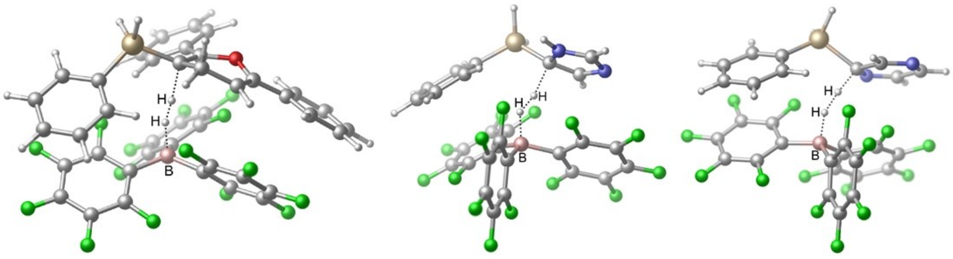

CH-d-TS **(substrate 23)** **CH-e-TS** **(substrate 8)** **CH-f-TS** **(substrate 26)**

Fig S12. Optimized transition states for C-H silylation of our selected six substrates with $PhSiH_3$ catalyzed by $B(C_6F_5)_3$.

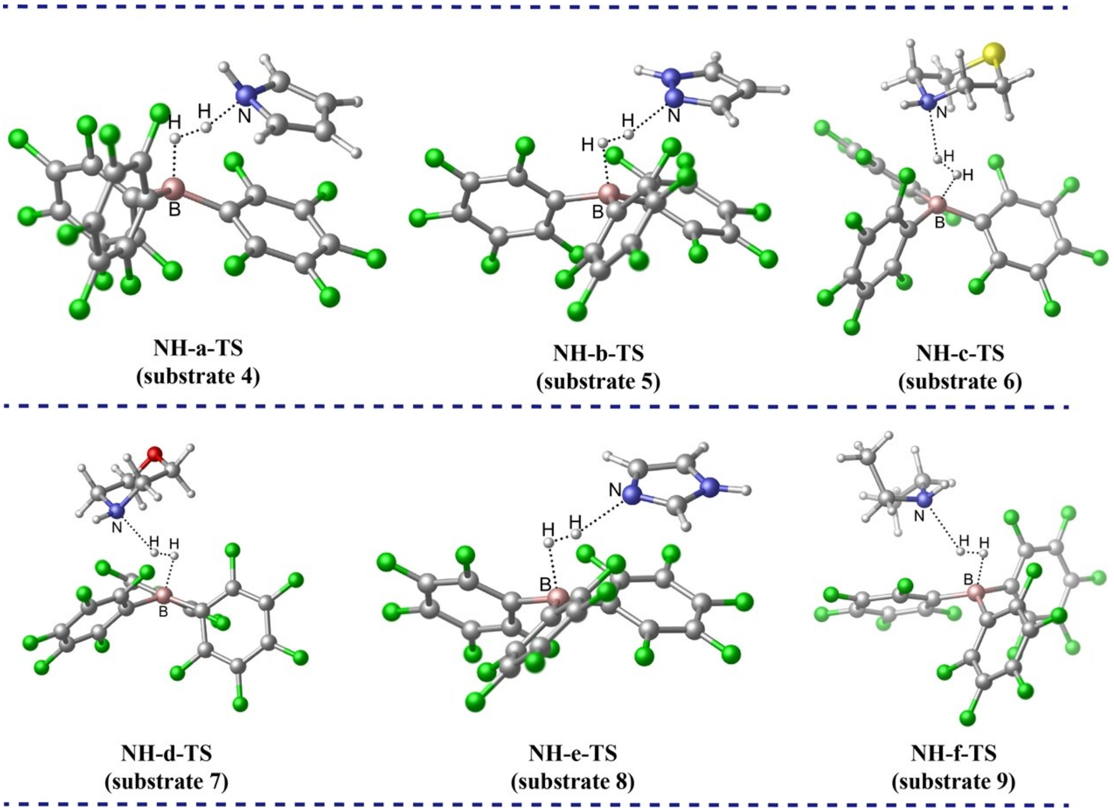

Fig S13. Optimized transition states for N-H silylation of our selected six substrates with $PhSiH_3$ catalyzed by $B(C_6F_5)_3$.

Table S1. Corrected free energies of all species.

Species	Corrected free energies	Species	Corrected free energies
**$PhSiH_3$**	-522.7889858	**N1-10**	-2575.275173
**H_2**	-1.1698301	**N1-11**	-3984.516824
**$B(C_6F_5)_3$**	-2208.12311	**N1-TS5**	-3984.503276
**indole**	-402.9351316	**N1-12**	-3984.522359
**indoline**	-404.1075367	**N1-13**	-3096.914692
**add1**	-2611.060044	**N1-TS6**	-3096.874225
**1**	-2730.905276	**N1-14**	-3096.883728
**2**	-3133.835916	**PhNHMe**	-326.7375942
**$TS_{2/3}$**	-3133.834329	**add3**	-2534.883766
**3**	-3133.838789	**N2-TS1**	-3057.640856
**4**	-3536.763785	**N2-2**	-3057.656747
**$TS_{4/5}$**	-3536.74667	**N2-3**	-3057.656913
**5**	-3536.765185	**N2-TS2**	-3057.624056
**6**	-924.5524073	**N2-4**	-3057.626797
**7**	-2612.220763	**N2-5**	-848.3653487
**$TS_{7/8}$**	-2612.213287	**N2-TS3**	-3384.38507
**8**	-2612.233119	**N2-6**	-3384.384761
**9**	-3133.841237	**N2-7**	-2536.031988
**$TS_{9/10}$**	-3133.810765	**N2-TS4**	-2535.997767
**10**	-3133.814322	**N2-8**	-2536.012093
**11**	-3537.947183	**N2-TS5**	-3057.6317521
**$TS_{11/12}$**	-3537.936712	**N2-9**	-3057.630872
**12**	-3537.95552	**N2-TS6**	-3384.3517333
**13**	-2613.410373	**N2-10**	-848.3502394
**$TS_{13/14}$**	-2613.367576	**N2-11**	-3057.63384
**14**	-2613.378167	**N2-TS7**	-3057.603609
**indoline**	-404.1075367	**N2-12**	-3057.612967
**19**	-2612.218882	**$PhNH_2$**	-287.4712056
**$TS_{19/20}$**	-2612.19177	**$PhNH_2$-add**	-2495.6210262
**20**	-2612.19544	**$PhNH_2$-product**	-1079.3503348
**15**	-3016.323253	**$PhNH_2$-TS**	-2496.7356253
**$TS_{15/16}$**	-3016.31862	**Ph_2NH**	-518.4045306
**16**	-3016.333269	**Ph_2NH-add**	-2726.5339755
**17**	-3015.153343	**Ph_2NH-product**	-1310.2787120
**$TS_{17/18}$**	-3015.125113	**Ph_2NH-TS**	-2727.6626575
**18**	-3015.144716	**$Al(C_6F_5)_3$**	-2425.7216584

**Si2-1**	-3535.595848	**$Al(C_6F_5)_3$-TS-A**	-3754.3406094
**Si2-TS1**	-3535.584215	**$Al(C_6F_5)_3$-TS-B**	-2830.9829864
**Si2-2**	-3535.600679	**$Al(C_6F_5)_3$-TS-C**	-3351.4194753
**Si2-4**	-3938.539554	**$Ga(C_6F_5)_3$**	-4108.1124811
**Si2-TS2**	-3938.514313	**$Ga(C_6F_5)_3$-TS-A**	-5436.7240244
**Si2-5**	-3938.526838	**$Ga(C_6F_5)_3$-TS-B**	-4513.3700046
**Si2-6**	-1326.31982	**$Ga(C_6F_5)_3$-TS-C**	-5033.7930826
**Si2-7**	-3939.713338	**$B(2,6\text{-}Cl_2C_6H_3)(p\text{-}HC_6F_4)_2$**	-2432.6581927
**Si2-TS3**	-3939.698527	**$B(2,6\text{-}Cl_2C_6H_3)(p\text{-}HC_6F_4)_2$-TS-A**	-3761.2696328
**Si2-8**	-3939.712084	**$B(2,6\text{-}Cl_2C_6H_3)(p\text{-}HC_6F_4)_2$-TS-B**	-2837.8970053
**Si2-TS4**	-3535.578712	**$B(2,6\text{-}Cl_2C_6H_3)(p\text{-}HC_6F_4)_2$-TS-C**	-3358.3363006
**Si2-9**	-3535.582665	**$PhSiH_3$(Bezene)**	-522.7880131
**$PhNMe_2$**	-366.0045911	**H_2(Bezene)**	-1.1696712
**add2**	-2574.120225	**$B(C_6F_5)_3$(Bezene)**	-2208.1194587
**N1-1**	-2730.905276	**CH-a-TS**	-3422.642152
**N1-2**	-3096.903056	**CH-b-TS**	-3418.844673
**N1-TS1**	-3096.89496	**CH-c-TS**	-3402.780036
**N1-3**	-3096.900926	**CH-d-TS**	-3461.897041
**N1-4**	-3096.902313	**CH-e-TS**	-2956.943111
**N1-TS2**	-3096.871137	**CH-f-TS**	-2994.989222
**N1-5**	-3096.881383	**NH-a-TS**	-2419.264671
**N1-6**	-887.6171461	**NH-b-TS**	-2435.326257
**N1-7**	-3462.901495	**NH-c-TS**	-2819.825516
**N1-TS3**	-3462.886634	**NH-d-TS**	-2496.834854
**N1-8**	-3462.905674	**NH-e-TS**	-2435.34723
**N1-9**	-2575.304944	**NH-f-TS**	-2422.81897
**N1-TS4**	-2575.267452		

Table S2. Imaginary frequencies of all transition states.

Species	Imaginary frequency	Species	Imaginary frequency
**$TS_{2/3}$**	-38.55 cm^{-1}	**N2-TS7**	-856.74 cm^{-1}
**$TS_{4/5}$**	-1245.55 cm^{-1}	**$PhNH_2$-TS**	-73.12 cm^{-1}
**$TS_{7/8}$**	-272.36 cm^{-1}	**Ph_2NH-TS**	-101.89 cm^{-1}
**$TS_{9/10}$**	-602.03 cm^{-1}	**$Al(C_6F_5)_3$-TS-A**	-1207.72 cm^{-1}
**$TS_{11/12}$**	-735.16 cm^{-1}	**$Al(C_6F_5)_3$-TS-B**	-309.82 cm^{-1}
**$TS_{13/14}$**	-503.59 cm^{-1}	**$Al(C_6F_5)_3$-TS-C**	-877.83 cm^{-1}
**$TS_{19/20}$**	-1310.24 cm^{-1}	**$Ga(C_6F_5)_3$-TS-A**	-1219.48 cm^{-1}
**$TS_{15/16}$**	-1073.32 cm^{-1}	**$Ga(C_6F_5)_3$-TS-B**	-427.31 cm^{-1}
**$TS_{17/18}$**	-1289.52 cm^{-1}	**$Ga(C_6F_5)_3$-TS-C**	-1161.17 cm^{-1}
**Si2-TS1**	-125.43 cm^{-1}	**$B(2,6-Cl_2C_6H_3)(p-HC_6F_4)_2$-TS-A**	-1204.45 cm^{-1}
**Si2-TS2**	-1187.81 cm^{-1}	**$B(2,6-Cl_2C_6H_3)(p-HC_6F_4)_2$-TS-B**	-164.63 cm^{-1}
**Si2-TS3**	-1215.09 cm^{-1}	**$B(2,6-Cl_2C_6H_3)(p-HC_6F_4)_2$-TS-C**	-628.91 cm^{-1}
**Si2-TS4**	-513.60 cm^{-1}	**CH-a-TS**	-1128.16 cm^{-1}
**N1-TS1**	-19.23 cm^{-1}	**CH-b-TS**	-975.71 cm^{-1}
**N1-TS2**	-971.99 cm^{-1}	**CH-c-TS**	-981.82 cm^{-1}
**N1-TS3**	-1011.61 cm^{-1}	**CH-d-TS**	-1046.56 cm^{-1}
**N1-TS4**	-163.20 cm^{-1}	**CH-e-TS**	-1100.10 cm^{-1}
**N1-TS5**	-1063.84 cm^{-1}	**CH-f-TS**	-626.78 cm^{-1}
**N1-TS6**	-432.10 cm^{-1}	**NH-a-TS**	-1204.25 cm^{-1}
**N2-TS1**	-86.51 cm^{-1}	**NH-b-TS**	-138.68 cm^{-1}
**N2-TS2**	-448.37 cm^{-1}	**NH-c-TS**	-139.12 cm^{-1}
**N2-TS3**	-568.59 cm^{-1}	**NH-d-TS**	-101.74 cm^{-1}
**N2-TS4**	-183.65 cm^{-1}	**NH-e-TS**	-91.14 cm^{-1}
**N2-TS5**	-21.99 cm^{-1}	**NH-f-TS**	-77.56 cm^{-1}
**N2-TS6**	-1126.04 cm^{-1}		

Table S3. Cartesian coordinates of all species.

$BPhF_3$

Symbol	X	Y	Z
F	3.355688	-3.368899	1.355477
F	1.287280	4.607038	1.269517
F	0.869893	-2.412727	1.363706
C	3.074123	-2.257854	0.684706
C	-0.642728	4.338159	-0.041347
C	0.443155	3.803624	0.632580
C	1.791642	-1.742289	0.671743
C	4.073793	-1.603516	-0.016553
F	-2.565989	4.017984	-1.350003
C	-1.528009	3.501908	-0.701927
F	1.693323	1.975361	1.294158
C	0.633226	2.434569	0.628265
C	-1.317624	2.135972	-0.671556
C	-0.234601	1.551331	-0.014858
C	1.452672	-0.571167	-0.006679
C	3.783802	-0.442281	-0.713840
F	-2.199899	1.379162	-1.324726
C	2.494322	0.055473	-0.691173
B	-0.004095	0.002471	-0.001927
F	4.743227	0.180228	-1.388909
F	2.272921	1.175140	-1.380469
C	-1.228414	-0.973451	0.017008
F	-2.539858	0.487036	1.342644
F	-0.116774	-2.593093	-1.305546
C	-2.413792	-0.666348	0.685617
C	-1.199716	-2.210025	-0.628366
C	-3.496407	-1.525011	0.726142
C	-2.269911	-3.084597	-0.623047
F	-4.600687	-1.198470	1.387653
F	-2.203459	-4.246957	-1.262034
C	-3.422657	-2.739451	0.063553
F	5.304251	-2.087137	-0.020557
F	-4.451703	-3.569097	0.086195
F	-0.834792	5.646185	-0.053478

H_2

Symbol	X	Y	Z
H	0. 0.000000	0.000000	0.370227
H	0. 0.000000	0.000000	-0.370227

$PhSiH_3$

Symbol	X	Y	Z
Si	2.341748	0.000269	0.004357
H	2.851076	-1.054713	-0.901535
H	2.863637	-0.266067	1.365320
H	2.826139	1.326849	-0.441134
C	0.464682	0.000483	-0.009381
C	-0.254528	1.201358	-0.007520
C	-0.254677	-1.201694	-0.007530
C	-1.646570	1.203537	0.002404
H	0.275024	2.148844	-0.017443
C	-1.645274	-1.204663	0.002634
H	0.274222	-2.150032	-0.017706
C	-2.343100	-0.000150	0.008124
H	-2.186185	2.143385	0.002427
H	-2.184852	-2.144530	0.003005
H	-3.426724	-0.000722	0.013689

add1

Symbol	X	Y	Z
F	0. 2.699864	-3.783019	-2.048900
F	0. -3.512512	-2.895922	-1.292838
F	0.720625	-2.048447	-1.915976
C	2.602689	-2.989077	-0.983567
C	-3.620785	-0.602001	-1.808897
C	-2.945765	-1.687141	-1.281247
C	1.564441	-2.075049	-0.878968
C	3.548632	-3.075802	0.020887
F	-3.663750	1.702759	-2.251661
C	-3.023811	0.644129	-1.759006
F	-1.139112	-2.600188	-0.168257
C	-1.698861	-1.499062	-0.709687
C	-1.772214	0.785457	-1.179847
C	-1.058836	-0.268238	-0.614935
C	1.401431	-1.225428	0.212064
C	3.449222	-2.233571	1.113028
F	-1.292102	2.036245	-1.158356
C	2.399329	-1.333132	1.176027
B	0.258943	-0.053966	0.331256
F	4.362736	-2.286858	2.081113
F	2.393515	-0.514322	2.244401

C	1.068476	1.340261	0.031974
F	1.208903	0.627027	-2.211760
F	1.109488	2.293458	2.220366
C	1.522192	1.530146	-1.271671
C	1.459506	2.330336	0.920453
C	2.276827	2.611823	-1.681932
C	2.216678	3.435867	0.553494
F	2.666954	2.734795	-2.949440
F	2.545908	4.356895	1.458613
C	2.629810	3.579269	-0.755262
F	4.546968	-3.948676	-0.067353
F	3.356375	4.630010	-1.123090
F	-4.824234	-0.754117	-2.351236
C	-1.666622	0.811354	1.963534
C	-2.744069	-0.079548	2.010667
C	-4.068023	0.290678	1.834535
C	-4.305329	1.645079	1.628798
C	-3.252136	2.564777	1.610072
C	-1.929511	2.163415	1.772079
C	-0.420817	-0.000526	1.978802
C	-0.912993	-1.331776	2.239817
H	-4.875340	-0.431288	1.848340
H	-5.320616	1.991364	1.483029
H	-1.126801	2.887050	1.729907
H	0.409344	0.305901	2.602709
N	-2.219008	-1.381110	2.202973
C	-3.042235	-2.584043	2.260883
H	-3.796513	-2.462834	3.036772
H	-3.524608	-2.735019	1.294068
H	-2.404750	-3.434081	2.489061
H	-3.470920	3.613597	1.453009
H	-0.331434	-2.233051	2.382807

1

Symbol	X	Y	Z
Si	0.630622	0.009676	2.656498
H	-0.124288	0.003150	1.307928
H	0.195310	-1.250562	3.282262
F	-4.559612	2.785529	-1.095610
F	3.312361	1.939829	-2.704963
F	-3.262735	0.467044	-1.170878
C	-3.328210	2.709617	-0.599994
C	3.589559	-0.172280	-1.717698
C	2.805215	0.931075	-2.001087
C	-2.631785	1.510037	-0.623811
C	-2.733604	3.835254	-0.059420
F	3.805898	-2.287525	-0.715335
C	3.058333	-1.222834	-0.988287
F	0.802009	2.074773	-1.859367
C	1.498516	0.980304	-1.539499
C	1.756447	-1.131298	-0.538641
C	0.927969	-0.039382	-0.785838
C	-1.339348	1.384460	-0.129765
C	-1.445315	3.755441	0.446722
F	1.303611	-2.142242	0.214997
C	-0.784195	2.545281	0.393042
B	-0.523667	0.011487	-0.134363
F	-0.865818	4.833256	0.965758
F	0.467031	2.507975	0.880560
C	-1.399370	-1.322118	-0.095726
F	-0.490431	-2.195259	-2.097899
F	-2.446300	-0.622769	1.899391
C	-1.334202	-2.310134	-1.067662
C	-2.319702	-1.534697	0.922916
C	-2.127693	-3.445118	-1.034942
C	-3.121752	-2.658610	0.997657
F	-2.036424	-4.366165	-1.989898
F	-3.980835	-2.822717	1.999314
C	-3.025061	-3.619200	0.004121
F	-3.388050	4.987729	-0.030007
F	-3.789632	-4.702365	0.050459
F	4.849798	-0.219737	-2.126472
H	0.101851	1.218880	3.307451
C	2.441057	-0.001339	2.258023
C	3.161933	-1.192792	2.421044
C	3.096079	1.115049	1.717382
C	4.497011	-1.272289	2.040834
H	2.675872	-2.070371	2.834083
C	4.427091	1.029980	1.325957
H	2.568818	2.053009	1.592128
C	5.125631	-0.165066	1.480460
H	5.038927	-2.201761	2.164496
H	4.918177	1.894440	0.894533
H	6.159479	-0.231933	1.162773

2

Symbol	X	Y	Z
Si	0.958918	0.303645	-1.359091

H	-0.404948	0.143236	-0.565320
H	1.114243	1.762202	-1.276667
F	-5.938809	-0.699015	-1.904335
F	-0.530374	-4.253140	2.843048
F	-4.364879	0.556092	-0.167348
C	-4.668991	-1.076674	-1.779976
C	0.751653	-2.345131	3.322788
C	-0.272778	-2.963798	2.631471
C	-3.824765	-0.433691	-0.886286
C	-4.186323	-2.120619	-2.547432
F	1.999094	-0.403164	3.764053
C	1.014872	-1.003690	3.100451
F	-1.992269	-2.907743	1.088343
C	-1.016261	-2.238106	1.711597
C	0.260435	-0.320679	2.167772
C	-0.773442	-0.898530	1.435658
C	-2.494490	-0.794264	-0.719315
C	-2.865923	-2.518471	-2.409722
F	0.589734	0.959826	1.944263
C	-2.062941	-1.855518	-1.503227
B	-1.486929	-0.047562	0.282982
F	-2.394259	-3.526586	-3.138523
F	-0.794062	-2.283211	-1.381499
C	-1.850684	1.485430	0.580447
F	-2.131134	1.196876	2.911176
F	-1.651200	1.979419	-1.718110
C	-2.147440	1.992992	1.836112
C	-1.911946	2.394902	-0.466211
C	-2.474627	3.322553	2.048345
C	-2.225608	3.731098	-0.297420
F	-2.752019	3.767411	3.271582
F	-2.260509	4.564731	-1.334118
C	-2.512722	4.195864	0.975060
F	-4.983470	-2.742730	-3.406968
F	-2.822546	5.473236	1.163800
F	1.491521	-3.032517	4.184461
H	0.499456	-0.256386	-2.636192
C	2.137433	-0.662560	-0.307256
C	3.046932	0.025458	0.506061
C	2.069292	-2.061582	-0.215620
C	3.860406	-0.667008	1.398592
H	3.121990	1.107857	0.450248
C	2.870744	-2.748980	0.687588
H	1.376824	-2.617087	-0.836843
C	3.761848	-2.050377	1.501176
H	4.556910	-0.120483	2.022919
H	2.795770	-3.827645	0.763722
H	4.375989	-2.586435	2.215407
C	4.928942	0.796478	-1.811013
C	5.872353	1.132236	-0.835049
C	3.991180	1.728840	-2.311640
C	5.852899	2.428536	-0.355529
H	6.574422	0.399835	-0.453595
C	3.998955	3.039398	-1.808228
C	4.922540	3.373730	-0.835911
H	6.563178	2.725787	0.406621
H	3.286458	3.771251	-2.172149
H	4.938289	4.379561	-0.433956
C	3.165377	1.017614	-3.246319
C	3.626510	-0.277267	-3.265181
H	2.384906	1.422789	-3.871792
N	4.685409	-0.415923	-2.412592
C	5.335645	-1.660158	-2.047554
H	6.417052	-1.564405	-2.157245
H	5.093613	-1.920673	-1.013994
H	4.981531	-2.448651	-2.708872
H	3.278419	-1.126040	-3.835576

3

Symbol	X	Y	Z
Si	-1.905213	0.526016	-1.603336
H	0.459774	-0.054242	-0.993395
H	-1.492395	-0.659935	-2.368092
F	5.108793	2.972948	-2.071657
F	0.793131	1.236704	4.630950
F	4.161294	0.577884	-1.523704
C	3.912855	2.867870	-1.487690
C	-0.303705	-0.666702	3.796987
C	0.537952	0.412337	3.609994
C	3.386317	1.619421	-1.180203
C	3.192482	4.008750	-1.194949
F	-1.364040	-2.569966	2.913124
C	-0.558973	-1.518491	2.736476
F	1.942551	1.678572	2.277250
C	1.111175	0.627120	2.363377
C	0.009945	-1.248486	1.505747
C	0.854198	-0.170537	1.259936
C	2.151599	1.434305	-0.573014

C	1.950465	3.880563	-0.596754
F	-0.305055	-2.087431	0.503227
C	1.471292	2.616894	-0.308117
B	1.425881	0.003628	-0.262481
F	1.234285	4.971388	-0.309736
F	0.258305	2.566492	0.276902
C	2.351246	-1.299821	-0.569314
F	3.894477	-0.646756	1.083340
F	0.930790	-2.145146	-2.270384
C	3.500906	-1.545729	0.166320
C	2.025823	-2.268379	-1.502213
C	4.294906	-2.664500	-0.003357
C	2.789441	-3.409823	-1.705381
F	5.400249	-2.848996	0.723174
F	2.435759	-4.318733	-2.619243
C	3.930548	-3.607948	-0.951116
F	3.681127	5.213955	-1.485952
F	4.677082	-4.697554	-1.134120
F	-0.883745	-0.878821	4.979600
H	-1.063681	1.720507	-1.760874
C	-2.487654	0.214713	0.137280
C	-3.155688	-0.971986	0.464821
C	-2.358935	1.207518	1.119277
C	-3.688549	-1.159253	1.736559
H	-3.266188	-1.758582	-0.276582
C	-2.878778	1.014442	2.395095
H	-1.838744	2.131046	0.893474
C	-3.547443	-0.168127	2.703229
H	-4.197287	-2.085047	1.976311
H	-2.754036	1.781571	3.150797
H	-3.944466	-0.322560	3.699936
C	-5.400688	0.161873	-1.463104
C	-6.331162	-0.751492	-0.983260
C	-4.493042	-0.126435	-2.485528
C	-6.335971	-1.998675	-1.588157
H	-7.007987	-0.510036	-0.173376
C	-4.508473	-1.388109	-3.077394
C	-5.441435	-2.309651	-2.623136
H	-7.041132	-2.748567	-1.252819
H	-3.810189	-1.641703	-3.865484
H	-5.478179	-3.294491	-3.071778
C	-3.596926	1.041475	-2.610564
C	-4.177259	2.009003	-1.718139
H	-3.214665	1.374016	-3.572805
N	-5.156259	1.478449	-1.024857
C	-5.814163	2.068785	0.136959
H	-6.892161	2.056095	-0.015711
H	-5.547920	1.480383	1.017376
H	-5.465000	3.091350	0.256437
H	-3.847242	3.022270	-1.529266

4

Symbol	X	Y	Z
Si	3.157224	2.340074	-1.486532
H	-0.511034	-0.603451	0.176836
H	4.503660	2.946876	-1.427692
F	-4.929187	-2.647598	-3.013306
F	-4.785551	-0.997377	4.041166
F	-4.042117	-0.447207	-1.794539
C	-3.906853	-2.731634	-2.160324
C	-2.831483	0.262524	4.362833
C	-3.668075	-0.470231	3.538919
C	-3.418015	-1.602552	-1.521251
C	-3.327394	-3.963288	-1.908489
F	-0.856653	1.527431	4.608495
C	-1.679777	0.816980	3.833107
F	-4.212688	-1.348233	1.467584
C	-3.337870	-0.647224	2.203145
C	-1.395576	0.613705	2.494353
C	-2.185714	-0.128163	1.630051
C	-2.355442	-1.632005	-0.630908
C	-2.276430	-4.046973	-1.012168
F	-0.263356	1.188179	2.032978
C	-1.829788	-2.893320	-0.390378
B	-1.685190	-0.337864	0.092604
F	-1.699677	-5.225991	-0.767407
F	-0.806482	-3.042062	0.476255
C	-1.800928	1.034562	-0.780397
F	-3.820072	1.731265	0.237133
F	0.196550	0.551951	-1.981010
C	-2.820367	1.965534	-0.625801
C	-0.854558	1.371066	-1.735113
C	-2.883111	3.154773	-1.332701
C	-0.890613	2.532113	-2.489501
F	-3.865134	4.032631	-1.117261
F	0.047713	2.786634	-3.404227
C	-1.909602	3.439654	-2.274657
F	-3.778523	-5.056495	-2.520634

F	-1.948621	4.583300	-2.955449
F	-3.134510	0.434952	5.647959
H	2.836910	1.998845	-2.887363
C	1.830654	3.452881	-0.790790
C	1.137649	3.190433	0.392282
C	1.475261	4.589220	-1.533822
C	0.095296	4.013432	0.811839
H	1.398758	2.337034	1.006521
C	0.449856	5.424524	-1.110138
H	1.988253	4.816124	-2.463636
C	-0.249950	5.129350	0.058635
H	-0.441910	3.775488	1.722069
H	0.181281	6.293038	-1.699430
H	-1.067651	5.766468	0.375743
C	3.951919	0.026042	1.616605
C	4.703783	-0.224852	2.752911
C	4.479329	0.553802	0.439850
C	6.059880	0.063510	2.668244
H	4.265360	-0.632008	3.655216
C	5.836958	0.837715	0.371680
C	6.617463	0.581374	1.494249
H	6.695803	-0.118454	3.525365
H	6.278653	1.238552	-0.532714
H	7.679765	0.789326	1.462028
C	3.372943	0.647964	-0.558800
C	2.238739	0.094915	0.184080
H	3.559660	0.004815	-1.437017
N	2.574720	-0.232493	1.398415
C	1.707954	-0.847539	2.403391
H	2.213993	-1.730932	2.789170
H	1.527919	-0.130013	3.203879
H	0.769755	-1.129904	1.928460
H	1.227157	-0.061937	-0.169846
C	4.467513	-2.426718	-1.235234
C	3.113004	-2.712745	-0.934720
C	2.713515	-3.265757	0.287780
C	3.704432	-3.505702	1.223106
C	5.057133	-3.206108	0.956136
C	5.446242	-2.674952	-0.257858
C	4.487082	-1.892809	-2.566045
C	3.186427	-1.876578	-3.002088
H	1.671548	-3.492619	0.489092
H	3.437966	-3.941696	2.179395
H	6.488191	-2.450154	-0.455593
H	5.348690	-1.572750	-3.130396
N	2.351321	-2.356843	-2.023629
C	0.913909	-2.542471	-2.123980
H	0.657647	-3.585762	-1.923045
H	0.392772	-1.900034	-1.412087
H	0.595036	-2.281151	-3.131287
H	5.800610	-3.398991	1.720273
H	2.776131	-1.552184	-3.946858

5

Symbol	X	Y	Z
Si	2.987091	2.803880	-0.746273
H	-0.442863	-0.598927	0.247372
H	4.183158	3.662431	-0.551914
F	-4.500275	-2.969617	-3.230474
F	-5.280405	-1.181652	3.484206
F	-3.859662	-0.735859	-1.935810
C	-3.508945	-2.986217	-2.338190
C	-3.278806	-0.194881	4.218935
C	-4.052894	-0.736759	3.207546
C	-3.146242	-1.834821	-1.656406
C	-2.836335	-4.169810	-2.087256
F	-1.257212	0.802561	4.891852
C	-2.011722	0.274941	3.924983
F	-4.357742	-1.321005	0.988798
C	-3.543727	-0.804522	1.920642
C	-1.545535	0.186783	2.622904
C	-2.275073	-0.358122	1.576452
C	-2.123088	-1.789409	-0.718824
C	-1.816131	-4.181255	-1.153641
F	-0.307068	0.672667	2.405229
C	-1.495036	-3.006847	-0.491856
B	-1.624550	-0.454700	0.083339
F	-1.149795	-5.312096	-0.907074
F	-0.486659	-3.094887	0.401741
C	-1.824808	0.926179	-0.768868
F	-4.024055	1.316336	0.013896
F	0.360770	0.792094	-1.708343
C	-2.975153	1.705452	-0.724064
C	-0.830054	1.429219	-1.592723
C	-3.126857	2.891520	-1.422351
C	-0.947424	2.588871	-2.341000
F	-4.243554	3.614987	-1.314052
F	0.046054	2.993643	-3.136520

C	-2.104306	3.336371	-2.242182
F	-3.168399	-5.284915	-2.734987
F	-2.229688	4.478402	-2.915264
F	-3.752674	-0.121744	5.461856
H	2.927873	2.513550	-2.207076
C	1.421181	3.740044	-0.291648
C	0.604753	3.373961	0.783179
C	1.035933	4.841478	-1.069522
C	-0.573474	4.065722	1.057573
H	0.879916	2.539700	1.417792
C	-0.129718	5.544994	-0.791275
H	1.644802	5.146443	-1.915910
C	-0.943307	5.149631	0.269114
H	-1.198570	3.755599	1.887582
H	-0.413933	6.389544	-1.407988
H	-1.864088	5.683781	0.475132
C	4.061625	-0.290691	1.743553
C	4.982375	-0.959926	2.552760
C	4.420122	0.814821	0.934109
C	6.295283	-0.524736	2.515067
H	4.683156	-1.792888	3.178608
C	5.761525	1.225774	0.912032
C	6.682978	0.551981	1.695422
H	7.039175	-1.020708	3.127135
H	6.068640	2.065859	0.298057
H	7.720404	0.865573	1.691546
C	3.219212	1.270423	0.266893
C	2.226627	0.416639	0.701787
H	4.709989	0.658489	-2.059726
N	2.714557	-0.515102	1.572226
C	1.925018	-1.504173	2.283879
H	2.449980	-2.461012	2.281731
H	1.749170	-1.187546	3.314433
H	0.967035	-1.620582	1.777078
H	1.172797	0.417013	0.467928
C	4.697906	-1.442797	-1.470405
C	3.487039	-2.088425	-1.253367
C	3.320507	-3.165240	-0.400948
C	4.467353	-3.600016	0.257171
C	5.698833	-2.972834	0.052727
C	5.830808	-1.886764	-0.809051
C	4.458257	-0.331810	-2.449986
C	2.996138	-0.450737	-2.701409
H	2.356664	-3.635635	-0.243431
H	4.400983	-4.437007	0.941015
H	6.787172	-1.398414	-0.945808
H	5.000842	-0.456807	-3.394479
N	2.492915	-1.444743	-2.053237
C	1.102582	-1.892957	-2.052175
H	1.092237	-2.965236	-2.246951
H	0.684912	-1.675368	-1.068943
H	0.550489	-1.357395	-2.818193
H	6.568192	-3.328919	0.591448
H	2.381776	0.169996	-3.341210

6

Symbol	X	Y	Z
Si	-3.176230	1.524231	1.787530
H	-3.504391	1.263771	3.211945
H	-1.895183	2.274566	1.754346
C	-4.492085	2.598848	0.986071
C	-5.809223	2.137976	0.849395
C	-4.185020	3.873205	0.495757
C	-6.784569	2.922707	0.243949
H	-6.077186	1.151873	1.218347
C	-5.157889	4.663026	-0.111768
H	-3.173383	4.255768	0.586756
C	-6.458389	4.187850	-0.238762
H	-7.797673	2.549860	0.147541
H	-4.900361	5.647003	-0.486372
H	-7.217033	4.800284	-0.712005
C	-2.557241	-2.210093	0.159065
C	-1.994152	-3.488717	0.163404
C	-2.340051	-1.278265	1.201161
C	-1.196282	-3.825622	1.243373
H	-2.172494	-4.187665	-0.645620
C	-1.526731	-1.651363	2.280794
C	-0.964682	-2.916032	2.292351
H	-0.740597	-4.807849	1.282477
H	-1.342120	-0.959342	3.095894
H	-0.334435	-3.215522	3.121143
C	-3.068643	-0.076604	0.864433
C	-3.673228	-0.349043	-0.341528
N	-3.377132	-1.614724	-0.771778
C	-3.845035	-2.246279	-1.989653
H	-2.999839	-2.533987	-2.618004
H	-4.436450	-3.134045	-1.756490
H	-4.466703	-1.538773	-2.534655

H -4.313897 0.284475 -0.939698

7

Symbol	X	Y	Z
H	0.429365	0.035005	1.742730
F	2.038899	-2.782695	-3.246179
F	-3.436916	0.137589	-2.460938
F	1.218315	-0.442320	-2.214816
C	1.789501	-2.677368	-1.940556
C	-2.987579	2.069929	-1.203401
C	-2.658897	0.788014	-1.593624
C	1.366903	-1.484615	-1.384329
C	1.952799	-3.783905	-1.120471
F	-2.521580	3.938005	0.148185
C	-2.190691	2.715582	-0.272730
F	-1.368345	-1.116656	-1.412436
C	-1.544152	0.169553	-1.043177
C	-1.078112	2.065017	0.226170
C	-0.691881	0.778083	-0.135450
C	1.072348	-1.322748	-0.035830
C	1.694221	-3.669153	0.231905
F	-0.349087	2.737294	1.134542
C	1.274124	-2.447993	0.741813
B	0.643813	0.136416	0.555800
F	1.831630	-4.730362	1.030018
F	1.025193	-2.425841	2.069385
C	1.971508	1.081143	0.402155
F	1.208236	2.463943	-1.371283
F	2.979763	-0.189506	2.120264
C	2.170792	2.111530	-0.504567
C	3.058164	0.809070	1.224647
C	3.350225	2.838556	-0.583147
C	4.252556	1.507033	1.180525
F	3.485997	3.824575	-1.473009
F	5.259970	1.200953	2.001694
C	4.398486	2.535212	0.265404
F	2.350658	-4.945774	-1.634019
F	5.539087	3.221120	0.200462
F	-4.086193	2.657341	-1.672663
H	-1.717472	1.014103	2.610865
C	-3.528605	0.106780	1.758003
C	-3.428563	-1.104364	1.079095
C	-4.301534	-1.515034	0.090722
C	-5.335280	-0.632026	-0.215308
C	-5.461587	0.588348	0.447960
C	-4.561629	0.972775	1.443537
C	-2.404449	0.172750	2.752051
C	-1.697324	-1.111393	2.504345
H	-4.185359	-2.455794	-0.432037
H	-6.042911	-0.894677	-0.990844
H	-4.664083	1.927489	1.945444
H	-2.746627	0.207627	3.792679
N	-2.284427	-1.800198	1.583347
C	-1.884148	-3.116575	1.085839
H	-2.743983	-3.781999	1.144873
H	-1.559595	-3.004153	0.051038
H	-1.074260	-3.491970	1.704970
H	-6.271470	1.254610	0.179053
H	-0.815488	-1.485121	3.006467

8

Symbol	X	Y	Z
H	-0.501761	-0.452452	1.738608
F	5.156207	-1.792907	-1.699682
F	-2.939846	-3.002131	-1.846680
F	3.151132	-0.017922	-1.917645
C	4.060323	-1.937013	-0.963685
C	-3.325641	-0.686328	-1.914019
C	-2.490394	-1.765613	-1.668396
C	3.016865	-1.033144	-1.057213
C	3.948913	-3.003311	-0.086929
F	-3.650639	1.638665	-1.971705
C	-2.868188	0.602953	-1.703957
F	-0.441140	-2.620193	-1.069653
C	-1.197399	-1.539514	-1.243271
C	-1.595728	0.793846	-1.199703
C	-0.692913	-0.257775	-0.973588
C	1.841368	-1.158612	-0.324372
C	2.799785	-3.157559	0.671645
F	-1.267416	2.053640	-0.930755
C	1.769131	-2.244875	0.536430
B	0.756966	-0.022210	-0.481168
F	2.698006	-4.175891	1.518443
F	0.687452	-2.429669	1.295429
C	1.306276	1.416760	-0.139620
F	0.997169	2.208170	-2.330856
F	1.715077	0.759531	2.087100
C	1.413174	2.429320	-1.082603

C	1.785807	1.694831	1.131894
C	1.957344	3.663681	-0.784493
C	2.318742	2.926554	1.473442
F	2.055934	4.610323	-1.710968
F	2.746254	3.167312	2.707789
C	2.406557	3.911621	0.504673
F	4.938484	-3.876200	0.024939
F	2.924246	5.092963	0.807178
F	-4.553283	-0.891549	-2.347702
H	-1.139769	1.838459	1.923107
C	-3.092555	0.874413	1.724653
C	-3.338825	-0.508908	1.768281
C	-4.462649	-1.053674	1.154003
C	-5.368974	-0.177606	0.545590
C	-5.149712	1.193501	0.530794
C	-3.994743	1.725922	1.121837
C	-1.796820	1.146923	2.455816
C	-1.189784	-0.263654	2.572835
H	-4.642055	-2.122285	1.151061
H	-6.252198	-0.584454	0.067008
H	-3.804868	2.793423	1.088507
H	-1.998881	1.568587	3.444784
N	-2.344874	-1.162012	2.482271
C	-2.115428	-2.573790	2.263818
H	-3.011722	-3.140243	2.522117
H	-1.844020	-2.800034	1.222004
H	-1.303609	-2.907772	2.909795
H	-5.858485	1.850442	0.042716
H	-0.639257	-0.429515	3.500009

9

Symbol	X	Y	Z
F	-3.791179	-4.085149	-1.716314
F	-4.829964	2.440748	-1.126469
F	-2.195034	-1.933357	-1.965724
C	-3.291830	-3.490483	-0.630298
C	-2.674076	3.300201	-1.478299
C	-3.505850	2.272188	-1.083233
C	-2.464625	-2.386968	-0.730603
C	-3.604243	-3.982900	0.626909
F	-0.482838	4.108134	-1.763548
C	-1.304617	3.117720	-1.408193
F	-3.853846	0.151904	-0.240888
C	-2.960156	1.080648	-0.621563
C	-0.812060	1.920218	-0.929624
C	-1.595422	0.846842	-0.523545
C	-1.926647	-1.727388	0.364705
C	-3.088562	-3.361813	1.749028
F	0.536613	1.832602	-0.853921
C	-2.264932	-2.253924	1.599666
B	-0.915573	-0.467141	0.170164
F	-3.388699	-3.834819	2.962005
F	-1.807296	-1.700299	2.734406
C	0.407494	-1.056209	-0.600371
F	0.004673	-0.112674	-2.737298
F	1.056133	-2.095871	1.429662
C	0.766535	-0.858642	-1.926214
C	1.287156	-1.853066	0.122779
C	1.923521	-1.379403	-2.489498
C	2.446965	-2.397693	-0.400106
F	2.246666	-1.114033	-3.756360
F	3.283269	-3.104175	0.372505
C	2.772201	-2.155908	-1.722363
F	-4.397194	-5.047013	0.752049
F	3.896495	-2.645639	-2.242127
F	-3.177034	4.466851	-1.882013
C	4.249827	-0.178043	0.796097
C	5.343715	-0.675281	0.106305
C	3.345566	0.740739	0.263902
C	5.513182	-0.206529	-1.191399
H	6.019706	-1.399501	0.542419
C	3.533536	1.203646	-1.032502
C	4.620913	0.714054	-1.750545
H	6.343140	-0.571247	-1.782554
H	2.840557	1.905726	-1.478825
H	4.774570	1.045373	-2.769928
C	2.305951	1.010134	1.300389
C	2.693157	0.101972	2.374478
H	1.273585	0.817135	0.984533
N	3.791413	-0.536076	2.092356
C	4.465709	-1.524413	2.931480
H	4.505181	-2.465225	2.384949
H	5.472492	-1.172434	3.152031
H	3.900393	-1.647868	3.850938
H	2.173145	-0.073071	3.307651
Si	2.214947	2.889668	1.841064
H	-0.552618	-0.109078	1.267254
H	2.960286	3.648805	0.818816

C	0.423848	3.405935	1.853109
C	0.045649	4.612313	1.250304
C	-0.572756	2.587922	2.399396
C	-1.292307	4.990026	1.194612
H	0.795395	5.255201	0.800992
C	-1.912291	2.954516	2.328872
H	-0.316467	1.641624	2.865989
C	-2.271804	4.157120	1.726920
H	-1.571948	5.919335	0.712199
H	-2.673200	2.298039	2.734333
H	-3.316675	4.438726	1.660900
H	2.877783	2.991255	3.158840

10

Symbol	X	Y	Z
F	-1.049714	5.173519	-1.905241
F	4.352379	1.092314	-2.025673
F	-0.868217	2.517122	-2.339679
C	-0.788185	4.348953	-0.896746
C	3.441762	-1.069246	-2.071312
C	3.318771	0.282987	-1.819664
C	-0.701774	2.983686	-1.095969
C	-0.592544	4.857136	0.378115
F	2.482067	-3.213676	-2.071539
C	2.361236	-1.908055	-1.851692
F	2.111351	2.090631	-1.056607
C	2.125332	0.781962	-1.320511
C	1.184146	-1.373949	-1.366436
C	1.014686	-0.020411	-1.077527
C	-0.422907	2.088600	-0.074345
C	-0.311696	3.997954	1.426031
F	0.182503	-2.228526	-1.139155
C	-0.229511	2.636915	1.181474
B	-0.356297	0.532334	-0.456066
F	-0.126082	4.480970	2.650701
F	0.044043	1.843589	2.233526
C	-1.756265	-0.059067	-0.963383
F	-0.980472	-0.765964	-3.084174
F	-2.738514	0.642001	1.059546
C	-1.971394	-0.660639	-2.195879
C	-2.868815	0.047297	-0.139768
C	-3.205526	-1.166717	-2.568791
C	-4.113097	-0.454485	-0.469279
F	-3.371599	-1.742357	-3.755889
F	-5.131269	-0.373860	0.381613
C	-4.277562	-1.072363	-1.696312
F	-0.673284	6.163935	0.592317
F	-5.457700	-1.574640	-2.034605
F	4.591782	-1.566518	-2.499875
C	-1.747949	-1.813200	2.544675
C	-3.126208	-1.980932	2.367690
C	-0.786475	-2.587299	1.857339
C	-3.531472	-2.940708	1.460054
H	-3.843725	-1.375214	2.908389
C	-1.235055	-3.555705	0.942781
C	-2.593412	-3.717300	0.749695
H	-4.590270	-3.093838	1.285302
H	-0.523338	-4.155853	0.389037
H	-2.949212	-4.458286	0.044041
C	0.522417	-2.142618	2.296074
C	0.268851	-1.149721	3.222337
H	-0.626905	0.086787	1.011876
N	-1.072641	-0.927330	3.356340
C	-1.704828	0.038369	4.237550
H	-2.405565	0.650329	3.667896
H	-2.237626	-0.470178	5.042924
H	-0.936515	0.681896	4.660600
H	0.960483	-0.559575	3.807366
Si	2.177173	-2.795363	1.763762
H	0.032114	-0.281671	0.823469
H	1.980901	-3.606071	0.537083
C	3.404980	-1.416309	1.379579
C	4.652410	-1.768506	0.841418
C	3.152784	-0.059222	1.608245
C	5.608231	-0.804119	0.542617
H	4.879061	-2.812060	0.638824
C	4.111769	0.911916	1.323144
H	2.193982	0.261817	2.004113
C	5.340527	0.540403	0.790819
H	6.558761	-1.099171	0.114254
H	3.891837	1.957239	1.506874
H	6.082791	1.294192	0.557519
H	2.777969	-3.676134	2.794176

11

Symbol	X	Y	Z
Si	-2.547091	-3.567803	0.535120
H	0.360386	0.574100	0.300568

H	-2.839091	-3.508190	1.982949
F	2.934399	3.566373	-3.800729
F	5.440967	1.445501	3.102123
F	2.818910	1.157205	-2.694663
C	2.413002	3.431481	-2.579659
C	3.620543	0.242209	3.969486
C	4.234956	0.905184	2.920825
C	2.338128	2.183707	-1.974724
C	1.940824	4.545963	-1.912106
F	1.760807	-0.952596	4.779683
C	2.370122	-0.316074	3.776791
F	4.247849	1.649391	0.729579
C	3.590920	0.996117	1.696662
C	1.767287	-0.195761	2.535634
C	2.337417	0.450896	1.451310
C	1.803317	1.982091	-0.711527
C	1.411026	4.397141	-0.641397
F	0.536468	-0.750727	2.426158
C	1.369022	3.137039	-0.072379
B	1.539155	0.555227	0.031104
F	0.933203	5.461058	0.008997
F	0.846880	3.056395	1.165279
C	1.817748	-0.749567	-0.911932
F	4.135706	-0.816803	-0.445022
F	-0.456674	-0.884364	-1.588701
C	3.067129	-1.336990	-1.066231
C	0.807641	-1.362518	-1.635524
C	3.299958	-2.448039	-1.858662
C	0.988421	-2.472118	-2.444580
F	4.516044	-2.992457	-1.941058
F	-0.041385	-3.018120	-3.096801
C	2.250651	-3.020081	-2.556490
F	1.996353	5.748625	-2.482179
F	2.448776	-4.114215	-3.289226
F	4.226183	0.144764	5.151502
H	-3.521603	-4.399657	-0.197386
C	-0.802752	-4.114700	0.186274
C	0.270236	-3.621202	0.942159
C	-0.543036	-5.073777	-0.801203
C	1.565928	-4.071082	0.715617
H	0.100630	-2.886002	1.722210
C	0.751339	-5.533535	-1.022135
H	-1.355475	-5.470431	-1.401130
C	1.804538	-5.033187	-0.262434
H	2.386599	-3.673715	1.301859
H	0.939341	-6.274010	-1.790029
H	2.815126	-5.383340	-0.442178
C	-4.192108	-0.515656	1.369912
C	-5.289256	-0.036082	2.064824
C	-4.280978	-1.417105	0.305577
C	-6.532000	-0.489293	1.638627
H	-5.189771	0.666767	2.882295
C	-5.531145	-1.866349	-0.101370
C	-6.650714	-1.382712	0.569692
H	-7.424787	-0.136722	2.139458
H	-5.632840	-2.566467	-0.921890
H	-7.636340	-1.708088	0.260688
C	-2.891689	-1.752184	-0.111898
C	-2.089100	-0.841602	0.693027
H	-2.636817	-1.764992	-1.171406
N	-2.825666	-0.203135	1.560467
C	-2.346301	0.709986	2.595716
H	-2.856734	1.665878	2.475866
H	-2.571474	0.283646	3.572927
H	-1.271512	0.830127	2.480448
H	-1.015077	-0.701899	0.666905
H	-1.772974	2.697344	-2.392819
H	-1.468882	3.787869	-0.257961
C	-3.390772	2.766119	-0.058056
C	-3.910473	1.775049	-0.905165
C	-5.282314	1.572582	-1.012742
C	-6.124403	2.348193	-0.213460
C	-5.616882	3.303056	0.660919
C	-4.235142	3.519431	0.735539
C	-1.888252	2.781730	-0.219694
C	-1.712903	2.008984	-1.535718
H	-5.693795	0.829262	-1.684721
H	-7.196043	2.197717	-0.279036
H	-3.833679	4.285830	1.390430
H	-1.390093	2.248582	0.596780
N	-2.871383	1.097319	-1.550250
C	-3.148278	0.421716	-2.800466
H	-3.939062	-0.318111	-2.654804
H	-3.463596	1.120820	-3.588364
H	-2.247824	-0.099801	-3.127666
H	-6.290729	3.894946	1.268022
H	-0.781363	1.447726	-1.589442

12

Symbol	X	Y	Z
Si	2.658773	3.241873	-0.840299
H	-0.346585	-0.555646	0.210935
H	3.763143	4.220353	-0.686523
F	-4.353102	-3.126968	-3.178642
F	-5.011654	-1.584543	3.596244
F	-3.873636	-0.914140	-1.775942
C	-3.309984	-3.084266	-2.347728
C	-3.052209	-0.494652	4.298073
C	-3.826742	-1.049890	3.293951
C	-3.031055	-1.942597	-1.612413
C	-2.497580	-4.197437	-2.215800
F	-1.076691	0.611522	4.937181
C	-1.830825	0.069412	3.978044
F	-4.175378	-1.568741	1.064793
C	-3.362722	-1.036420	1.988406
C	-1.409115	0.059247	2.657863
C	-2.139288	-0.495425	1.617127
C	-1.958792	-1.838994	-0.736647
C	-1.427470	-4.150395	-1.341174
F	-0.213214	0.635645	2.418174
C	-1.192633	-2.989637	-0.622187
B	-1.541928	-0.498977	0.099195
F	-0.630369	-5.216038	-1.203880
F	-0.136179	-3.022291	0.215559
C	-1.882453	0.874920	-0.715258
F	-4.034205	1.165103	0.230049
F	0.227002	0.824632	-1.807955
C	-3.062420	1.597213	-0.586606
C	-0.973599	1.417381	-1.609583
C	-3.318696	2.769975	-1.276037
C	-1.192728	2.573804	-2.340208
F	-4.455212	3.445148	-1.087411
F	-0.270290	3.034469	-3.188700
C	-2.377457	3.261120	-2.164182
F	-2.747120	-5.301589	-2.917237
F	-2.607784	4.392835	-2.827209
F	-3.482006	-0.499067	5.559063
H	2.624197	2.855391	-2.275904
C	1.017314	4.032323	-0.385288
C	0.216537	3.582479	0.670587
C	0.562271	5.120831	-1.143443
C	-1.007137	4.187798	0.949007

H	0.537395	2.750897	1.287840
C	-0.649915	5.738161	-0.859014
H	1.156405	5.488148	-1.975278
C	-1.440979	5.266398	0.186394
H	-1.620179	3.812952	1.760996
H	-0.986185	6.574730	-1.459753
H	-2.396886	5.732536	0.397026
C	4.118264	0.233188	1.581307
C	5.147514	-0.397063	2.289829
C	4.322745	1.415399	0.829095
C	6.404357	0.173014	2.217893
H	4.966237	-1.295660	2.867690
C	5.614928	1.967169	0.775195
C	6.637135	1.341749	1.463857
H	7.227331	-0.283907	2.753887
H	5.802558	2.870889	0.206533
H	7.636096	1.759586	1.435344
C	3.052135	1.771368	0.228591
C	2.171370	0.792277	0.650130
H	3.593386	0.029298	-0.924962
N	2.798299	-0.131315	1.439635
C	2.178982	-1.299779	2.045784
H	2.822859	-2.168737	1.892722
H	2.023415	-1.138427	3.113887
H	1.219251	-1.477447	1.561326
H	1.119371	0.674895	0.436125
H	3.083907	-1.271334	-3.475729
H	1.194356	-2.453153	-2.543888
C	2.771505	-2.686150	-1.053941
C	3.966790	-1.996356	-0.928834
C	5.066718	-2.470687	-0.241545
C	4.939007	-3.729723	0.343827
C	3.752965	-4.453131	0.226736
C	2.658984	-3.940050	-0.468356
C	1.777150	-1.865204	-1.834736
C	2.683904	-0.859803	-2.548770
H	5.983008	-1.901120	-0.150590
H	5.775291	-4.148389	0.889071
H	1.738096	-4.503503	-0.548423
H	1.081010	-1.357085	-1.160844
N	3.864645	-0.693231	-1.614114
C	5.103686	-0.207444	-2.276640
H	5.851289	-0.002427	-1.512129
H	5.450926	-0.973415	-2.967237

H	4.861557	0.712241	-2.807196
H	3.681734	-5.432867	0.682685
H	2.238972	0.117155	-2.724669

13

Symbol	X	Y	Z
H	-0.248588	0.018127	-1.508872
F	-3.295457	-2.658558	2.829236
F	3.070484	-0.790714	3.010256
F	-2.005492	-0.404176	2.136301
C	-2.656569	-2.585322	1.661888
C	3.111100	1.243816	1.836784
C	2.492607	0.051143	2.149591
C	-1.995538	-1.433432	1.277668
C	-2.644040	-3.686171	0.818044
F	3.147686	3.208290	0.547706
C	2.538445	2.071648	0.885558
F	0.824819	-1.512660	1.832360
C	1.314350	-0.302190	1.504106
C	1.367493	1.677905	0.267347
C	0.702740	0.485714	0.540778
C	-1.311696	-1.307365	0.076000
C	-1.969559	-3.613367	-0.385986
F	0.885208	2.498386	-0.677954
C	-1.326258	-2.432247	-0.725891
B	-0.613145	0.101958	-0.347811
F	-1.937160	-4.671127	-1.198090
F	-0.664588	-2.435333	-1.907386
C	-1.773019	1.248086	-0.352776
F	-1.100666	2.381775	1.618959
F	-2.662421	0.263869	-2.307939
C	-1.943410	2.256990	0.583264
C	-2.725556	1.216644	-1.363198
C	-2.971071	3.186521	0.519005
C	-3.767797	2.122378	-1.465076
F	-3.086697	4.141944	1.443666
F	-4.651724	2.046067	-2.462319
C	-3.889366	3.119248	-0.512767
F	-3.270085	-4.806458	1.170727
F	-4.883388	4.002753	-0.587879
F	4.275256	1.568163	2.393342
H	1.152319	-0.929521	-1.535792
H	2.878332	-3.180965	-2.195375
H	4.210439	-3.020655	-0.246667
C	3.932257	-0.919355	-0.718473
C	3.118875	-0.368331	-1.691883
C	3.322852	0.877260	-2.257026
C	4.424280	1.596725	-1.800732
C	5.265234	1.067859	-0.821241
C	5.028485	-0.190385	-0.273245
C	3.420696	-2.271693	-0.292623
C	2.409472	-2.610467	-1.394405
H	2.658820	1.286405	-3.007876
H	4.621630	2.579957	-2.207401
H	5.680295	-0.591224	0.493975
H	2.946885	-2.224668	0.690112
N	2.005420	-1.282739	-2.006964
C	1.675691	-1.380987	-3.457754
H	1.252886	-0.433565	-3.784818
H	2.593211	-1.602848	-3.998820
H	0.943133	-2.173371	-3.585449
H	6.111729	1.648850	-0.477185
H	1.510525	-3.120860	-1.058650

14

Symbol	X	Y	Z
H	0.973924	-0.926818	-2.666436
F	0.812696	3.773365	3.195620
F	-3.313695	-1.305515	2.449218
F	1.095679	1.136546	2.673736
C	0.713708	3.324413	1.949014
C	-2.385717	-2.865873	0.962773
C	-2.370336	-1.625897	1.573126
C	0.859967	1.980223	1.661092
C	0.457251	4.207542	0.911788
F	-1.428296	-4.387704	-0.547836
C	-1.387148	-3.208324	0.063167
F	-1.433753	0.456898	1.863583
C	-1.367885	-0.726550	1.254872
C	-0.368438	-2.313094	-0.185036
C	-0.306209	-1.043570	0.401370
C	0.751439	1.467816	0.377672
C	0.366894	3.735460	-0.386371
F	0.584913	-2.709157	-1.030904
C	0.521432	2.380996	-0.636035
B	0.910277	-0.097182	0.185160
F	0.124723	4.581435	-1.385062
F	0.451256	1.982230	-1.908457

C	2.354681	-0.657425	-0.050178
F	1.985726	-2.557710	1.318507
F	2.964534	1.157537	-1.438642
C	2.802949	-1.833041	0.554953
C	3.301887	0.036163	-0.804014
C	4.099280	-2.295113	0.423211
C	4.600355	-0.408680	-0.972273
F	4.488943	-3.411698	1.026863
F	5.462223	0.270683	-1.719709
C	5.000237	-1.578744	-0.348092
F	0.309602	5.501574	1.161148
F	6.240747	-2.013280	-0.489911
F	-3.357526	-3.722235	1.223364
H	0.941407	-0.192859	-2.774867
H	-3.398686	2.588762	-2.468511
H	-3.995974	2.876475	-0.135629
C	-3.822753	0.714067	-0.444352
C	-3.154697	0.046507	-1.486979
C	-3.483657	-1.260919	-1.825759
C	-4.488502	-1.897353	-1.089047
C	-5.145894	-1.248072	-0.051182
C	-4.811214	0.074146	0.274158
C	-3.252285	2.111102	-0.353808
C	-2.632785	2.257658	-1.749827
H	-2.990997	-1.775579	-2.641971
H	-4.758194	-2.917683	-1.338362
H	-5.326634	0.587820	1.078092
H	-2.471869	2.151371	0.415244
N	-2.208886	0.887629	-2.076566
C	-1.851934	0.650716	-3.461369
H	-1.431414	-0.348335	-3.575035
H	-2.721216	0.749006	-4.128184
H	-1.090586	1.371866	-3.758501
H	-5.919603	-1.762628	0.504954
H	-1.793036	2.949460	-1.801537

15

Symbol	X	Y	Z
H	-1.007525	0.106582	1.398992
F	-4.424947	2.538128	-2.821332
F	0.883839	-0.768715	-3.936659
F	-3.304140	0.258549	-1.947969
C	-3.656379	2.544447	-1.731794
C	0.961119	-2.473109	-2.321752
C	0.508569	-1.241206	-2.746395
C	-3.068587	1.385260	-1.261041
C	-3.438556	3.735018	-1.054233
F	1.065019	-4.096012	-0.623290
C	0.591992	-2.929471	-1.067237
F	-0.613741	0.741358	-2.381987
C	-0.300200	-0.482850	-1.909605
C	-0.229300	-2.145273	-0.280377
C	-0.721919	-0.899064	-0.657164
C	-2.247629	1.334745	-0.140826
C	-2.642997	3.736024	0.075227
F	-0.535040	-2.632579	0.935602
C	-2.078800	2.543736	0.509115
B	-1.666628	-0.090341	0.404017
F	-2.412595	4.877487	0.728208
F	-1.303777	2.636489	1.611977
C	-2.975657	-0.953598	0.875849
F	-3.013486	-2.514199	-0.914154
F	-3.190556	0.509022	2.719314
C	-3.543092	-2.038131	0.223592
C	-3.640443	-0.551731	2.028166
C	-4.672720	-2.698635	0.686293
C	-4.769778	-1.179372	2.525178
F	-5.173033	-3.743263	0.022279
F	-5.362411	-0.750019	3.642012
C	-5.290034	-2.267435	1.845677
F	-3.989587	4.865442	-1.490963
F	-6.376418	-2.889314	2.302613
F	1.784096	-3.190778	-3.083614
H	1.380901	-0.936802	1.553895
C	2.689165	-0.299036	-0.107703
C	2.370297	0.816184	-0.878559
C	2.816142	1.018076	-2.171933
C	3.624858	0.014975	-2.701036
C	3.957275	-1.115192	-1.952270
C	3.497859	-1.284865	-0.646999
C	2.046996	-0.128655	1.237505
C	1.308787	1.143781	1.070120
H	2.537577	1.888392	-2.752392
H	3.991984	0.111771	-3.714610
H	3.768190	-2.162253	-0.072255
H	2.808273	0.006537	2.030882
N	1.519991	1.659386	-0.098089
C	0.991112	2.928553	-0.596801

H	1.824418	3.532088	-0.954648
H	0.298020	2.716206	-1.411054
H	0.482042	3.435740	0.218034
H	4.587805	-1.876353	-2.394216
H	0.683417	1.646715	1.794429
H	5.629829	2.337948	3.412586
H	5.452571	3.197929	1.179974
C	5.660055	1.043627	0.858101
C	5.546836	0.035279	1.826044
C	6.137368	-1.208550	1.638075
C	6.829681	-1.431292	0.444315
C	6.932477	-0.441893	-0.526522
C	6.341968	0.810065	-0.319002
C	4.920585	2.263444	1.355546
C	4.768457	1.945577	2.853095
H	6.073143	-1.985052	2.390563
H	7.300846	-2.393574	0.280529
H	6.424911	1.587918	-1.070443
H	3.942338	2.341862	0.867790
N	4.769449	0.474223	2.907822
C	4.999972	-0.114987	4.213978
H	4.803005	-1.187101	4.182393
H	6.030495	0.047908	4.559217
H	4.311878	0.332955	4.931405
H	7.476948	-0.637593	-1.442218
H	3.854110	2.343560	3.296844

16

Symbol	X	Y	Z
H	-1.299581	0.187735	1.500306
F	-4.432614	2.829034	-2.795335
F	0.705383	-1.093871	-3.780535
F	-3.620442	0.458616	-1.839337
C	-3.613892	2.776867	-1.742165
C	0.865621	-2.564023	-1.956336
C	0.348191	-1.422067	-2.534501
C	-3.185145	1.567827	-1.224639
C	-3.177074	3.954989	-1.156972
F	1.058124	-3.939248	-0.058466
C	0.530613	-2.862044	-0.648101
F	-0.943854	0.490583	-2.456084
C	-0.505814	-0.607665	-1.804505
C	-0.320552	-2.018303	0.042855
C	-0.882575	-0.865443	-0.495160
C	-2.324335	1.454320	-0.140384
C	-2.326830	3.896372	-0.069312
F	-0.603507	-2.369634	1.309264
C	-1.932206	2.658330	0.418747
B	-1.881272	0.003764	0.462408
F	-1.889125	5.026355	0.495220
F	-1.098263	2.691635	1.478290
C	-3.247912	-0.825287	0.823341
F	-3.200609	-2.317369	-1.024647
F	-3.561133	0.573184	2.705203
C	-3.788527	-1.878417	0.099394
C	-3.978208	-0.453599	1.945326
C	-4.953148	-2.537652	0.466815
C	-5.145522	-1.081077	2.347667
F	-5.426987	-3.550237	-0.265049
F	-5.802570	-0.682381	3.441024
C	-5.636101	-2.137394	1.600157
F	-3.570448	5.132329	-1.642403
F	-6.758035	-2.758460	1.966614
F	1.696575	-3.351348	-2.640756
H	2.005204	-0.981921	2.627603
C	2.780333	-0.855416	0.505808
C	2.540967	0.143121	-0.466171
C	3.046649	0.059451	-1.770415
C	3.821277	-1.041296	-2.076903
C	4.087887	-2.041533	-1.116962
C	3.575722	-1.962959	0.161169
C	2.093460	-0.440228	1.698744
C	1.480883	0.755412	1.402894
H	2.816396	0.811910	-2.516294
H	4.217265	-1.150322	-3.079229
H	3.773202	-2.743284	0.886957
H	3.756562	0.836236	1.892622
N	1.767226	1.124701	0.113461
C	1.229726	2.279162	-0.587044
H	2.019897	2.763184	-1.164370
H	0.423044	1.978404	-1.259425
H	0.843301	2.984294	0.145835
H	4.694031	-2.893381	-1.399956
H	0.831161	1.371843	2.005269
H	5.411400	3.243084	1.841318
H	5.891878	3.051260	-0.476499
C	6.021060	0.977349	0.161839
C	5.701777	0.452573	1.400904

C	6.235462	-0.715488	1.908962
C	7.150234	-1.385297	1.100800
C	7.489291	-0.885952	-0.155825
C	6.929823	0.295400	-0.636084
C	5.258570	2.252319	-0.092604
C	4.717508	2.600766	1.299058
H	5.959564	-1.102914	2.881610
H	7.599504	-2.304079	1.455380
H	7.198458	0.677686	-1.613366
H	4.453277	2.077122	-0.808177
N	4.678747	1.288821	2.053272
C	4.820785	1.449065	3.525287
H	4.661449	0.484357	4.001529
H	5.820742	1.822138	3.736357
H	4.064529	2.154777	3.862979
H	8.202331	-1.425181	-0.767024
H	3.720499	3.037807	1.316267

17

Symbol	X	Y	Z
H	-0.786979	0.405736	1.196183
F	-3.950992	2.158293	-3.516120
F	0.698790	-2.410765	-3.569403
F	-3.312102	0.059464	-1.969228
C	-3.074696	2.302243	-2.521558
C	0.702636	-3.423900	-1.453618
C	0.327269	-2.390167	-2.288112
C	-2.733957	1.238675	-1.706399
C	-2.487695	3.539045	-2.300549
F	0.751481	-4.329692	0.715257
C	0.357671	-3.363126	-0.114688
F	-0.667961	-0.341424	-2.667009
C	-0.384577	-1.313624	-1.776015
C	-0.351784	-2.270426	0.349016
C	-0.750071	-1.198192	-0.443251
C	-1.818118	1.327934	-0.664804
C	-1.576700	3.683073	-1.271949
F	-0.633754	-2.260165	1.665899
C	-1.275705	2.586352	-0.476530
B	-1.510718	0.046021	0.296154
F	-0.993606	4.864484	-1.056800
F	-0.372229	2.814073	0.505454
C	-2.924577	-0.401538	0.985407
F	-3.292591	-2.362738	-0.301121
F	-2.814990	1.523468	2.352544
C	-3.686802	-1.514085	0.660314
C	-3.461634	0.406756	1.979528
C	-4.885962	-1.823584	1.286476
C	-4.654111	0.137169	2.629416
F	-5.578635	-2.910588	0.938683
F	-5.119598	0.948116	3.582663
C	-5.372358	-0.992776	2.278688
F	-2.797033	4.575116	-3.077045
F	-6.523214	-1.274279	2.889095
F	1.414217	-4.446619	-1.922757
H	1.341211	-1.039368	2.079355
C	2.860689	-1.378503	0.539541
C	2.758715	-0.679047	-0.657544
C	3.252273	-1.138222	-1.866264
C	3.881571	-2.378071	-1.839184
C	4.002063	-3.100480	-0.648067
C	3.495489	-2.611245	0.553735
C	2.208057	-0.557455	1.611569
C	1.773103	0.642662	0.858004
H	3.146048	-0.575601	-2.785676
H	4.277719	-2.791945	-2.757546
H	3.586205	-3.182177	1.469409
H	2.893653	-0.268842	2.420359
N	2.078533	0.548543	-0.394332
C	1.775633	1.524562	-1.439775
H	2.680196	1.700345	-2.018712
H	0.986939	1.118825	-2.075223
H	1.446475	2.447595	-0.970090
H	4.493880	-4.065001	-0.663898
H	1.250009	1.505676	1.244166
C	5.324428	0.692696	1.744877
C	4.649999	1.865135	1.325613
C	4.691823	2.320993	0.002633
C	5.396377	1.555101	-0.908885
C	6.053224	0.369157	-0.519301
C	6.026895	-0.064636	0.791596
C	5.086805	0.564916	3.154046
C	4.299633	1.624737	3.517542
H	4.195183	3.237998	-0.294677
H	5.451042	1.875837	-1.942992
H	6.536907	-0.975521	1.083467
H	5.448692	-0.210967	3.809949
N	4.011281	2.403179	2.420539

C	3.317243	3.678848	2.436953
H	4.019365	4.502472	2.294632
H	2.562569	3.715396	1.648594
H	2.819323	3.793218	3.397656
H	6.586220	-0.207315	-1.265688
H	3.903088	1.894920	4.484735

18

Symbol	X	Y	Z
H	0.855929	0.115552	-1.418791
F	2.138282	2.586256	3.836382
F	-1.323992	-2.790121	2.909500
F	2.399308	0.377593	2.331256
C	1.594553	2.536756	2.618778
C	-0.383895	-3.889675	1.052834
C	-0.557323	-2.751344	1.812807
C	1.716203	1.412549	1.822751
C	0.893524	3.632691	2.138629
F	0.528240	-4.892643	-0.868659
C	0.378987	-3.808591	-0.100357
F	-0.244232	-0.496180	2.197105
C	0.032648	-1.558927	1.413230
C	0.951342	-2.600627	-0.453986
C	0.807536	-1.424130	0.273763
C	1.158133	1.296906	0.555663
C	0.334335	3.575807	0.876849
F	1.663672	-2.588093	-1.595297
C	0.482471	2.422409	0.118298
B	1.406695	-0.039252	-0.354962
F	-0.333653	4.632943	0.397329
F	-0.102461	2.455604	-1.100195
C	3.018653	-0.100079	-0.638225
F	3.532728	-1.918755	0.800256
F	2.782789	1.754808	-2.085536
C	3.929767	-0.979668	-0.072092
C	3.572692	0.835953	-1.502594
C	5.288243	-0.956651	-0.355906
C	4.920469	0.897014	-1.813499
F	6.120539	-1.832882	0.213293
F	5.393506	1.817988	-2.658335
C	5.787734	-0.012339	-1.233264
F	0.764309	4.728858	2.884905
F	7.090686	0.025223	-1.515118
F	-0.950261	-5.043120	1.408571
H	-1.307960	-0.917247	-2.336226
C	-2.600100	-2.212856	-1.016716
C	-3.151859	-1.916991	0.253959
C	-3.828635	-2.873703	1.019056
C	-3.933965	-4.145877	0.489701
C	-3.387991	-4.464872	-0.770980
C	-2.730727	-3.514865	-1.528115
C	-1.919348	-1.030212	-1.453349
C	-2.061292	-0.105683	-0.452165
H	-4.224650	-2.635291	1.998996
H	-4.430605	-4.919942	1.061873
H	-2.294525	-3.774449	-2.486209
H	-4.822151	-0.321997	-1.194088
N	-2.820410	-0.618858	0.568268
C	-3.133394	0.038926	1.822733
H	-4.214776	0.118885	1.952075
H	-2.704220	-0.521052	2.655170
H	-2.700783	1.038782	1.806322
H	-3.476047	-5.479157	-1.140832
H	-1.632722	0.881594	-0.366797
C	-4.614532	1.831905	-0.915658
C	-3.552733	2.486461	-1.535127
C	-2.920446	3.599707	-1.013016
C	-3.424816	4.079053	0.194327
C	-4.511627	3.464395	0.817871
C	-5.119130	2.333396	0.272118
C	-4.957142	0.623515	-1.737038
C	-3.962873	0.712362	-2.836158
H	-2.068113	4.062568	-1.492749
H	-2.957463	4.939903	0.654289
H	-5.951489	1.853956	0.772554
H	-5.975316	0.624461	-2.140120
N	-3.222861	1.765003	-2.722807
C	-2.083110	2.112195	-3.568834
H	-2.162158	3.159752	-3.853728
H	-1.173761	1.948208	-2.989237
H	-2.094094	1.478209	-4.451095
H	-4.882553	3.868021	1.751645
H	-3.842366	0.025708	-3.663305

19

Symbol	X	Y	Z
H	0.518459	-0.093996	-1.768990
F	3.009038	3.027839	2.596305

F	-2.936251	-0.004682	2.923036
F	1.965773	0.640839	1.967419
C	2.422585	2.846415	1.412517
C	-2.934697	-1.750196	1.349211
C	-2.358258	-0.610123	1.877151
C	1.881227	1.623298	1.060351
C	2.350008	3.902341	0.517009
F	-2.934652	-3.408994	-0.315327
C	-2.367826	-2.332969	0.231376
F	-0.790657	1.086489	1.818270
C	-1.231942	-0.066566	1.278000
C	-1.231395	-1.766574	-0.322096
C	-0.610146	-0.623376	0.169156
C	1.258414	1.378852	-0.157644
C	1.731805	3.714573	-0.704578
F	-0.741250	-2.372449	-1.413369
C	1.207439	2.466751	-1.013368
B	0.747028	-0.110519	-0.585572
F	1.639342	4.728917	-1.567558
F	0.601405	2.369079	-2.216148
C	1.978840	-1.165201	-0.380616
F	1.211099	-2.138446	1.644049
F	2.984072	-0.319282	-2.345950
C	2.121209	-2.071815	0.659737
C	3.008060	-1.177400	-1.313275
C	3.189944	-2.950470	0.762554
C	4.093501	-2.035257	-1.249186
F	3.274518	-3.808019	1.782847
F	5.050258	-2.007352	-2.180102
C	4.182294	-2.933770	-0.200117
F	2.863243	5.088471	0.836008
F	5.216066	-3.770657	-0.116249
F	-4.050549	-2.244652	1.879767
H	-1.220810	0.618464	-2.246672
C	-3.348021	0.312772	-1.828176
C	-3.890806	0.835877	-0.659006
C	-4.985352	0.298489	-0.004087
C	-5.537354	-0.843925	-0.576037
C	-5.004240	-1.397580	-1.743022
C	-3.908150	-0.825919	-2.385591
C	-2.174490	1.161135	-2.214915
C	-2.141339	2.158915	-1.120100
H	-5.379592	0.722019	0.911608
H	-6.385693	-1.316952	-0.098769
H	-3.495723	-1.267832	-3.283959
H	-2.283151	1.665098	-3.182202
N	-3.100033	1.964818	-0.276615
C	-3.353537	2.730031	0.943311
H	-4.360529	3.141428	0.892480
H	-3.264957	2.057828	1.796759
H	-2.618082	3.526300	1.017422
H	-5.449623	-2.295592	-2.151956
H	-1.434914	2.968355	-0.989924

20

Symbol	X	Y	Z
H	0.646908	-0.244972	-1.793755
F	2.849796	3.034655	2.774555
F	-3.147160	0.525830	2.884226
F	1.689166	0.671808	2.170570
C	2.396286	2.806038	1.546575
C	-3.125208	-1.402009	1.545891
C	-2.543152	-0.217253	1.955333
C	1.798613	1.605180	1.217175
C	2.519155	3.784233	0.570992
F	-3.102598	-3.273276	0.125087
C	-2.532023	-2.148524	0.542164
F	-0.906851	1.406898	1.758450
C	-1.375152	0.221855	1.352242
C	-1.356285	-1.690990	-0.018434
C	-0.727179	-0.511691	0.367605
C	1.298216	1.328990	-0.047387
C	2.049876	3.543582	-0.707650
F	-0.813079	-2.425662	-0.997480
C	1.457130	2.323674	-0.997212
B	0.685034	-0.132252	-0.279880
F	2.168637	4.478252	-1.646112
F	1.034636	2.138890	-2.256852
C	1.823564	-1.270112	-0.264592
F	0.996184	-2.375837	1.660814
F	2.819420	-0.289390	-2.154041
C	1.914729	-2.265579	0.699006
C	2.852532	-1.230228	-1.196252
C	2.961238	-3.175043	0.726171
C	3.905688	-2.124315	-1.209771
F	3.017268	-4.111976	1.666988
F	4.856286	-2.053703	-2.134937
C	3.956875	-3.105635	-0.233161

F	3.088001	4.946345	0.864585
F	4.956482	-3.975934	-0.217408
F	-4.271537	-1.797024	2.080779
H	-0.047584	0.072696	-1.626872
C	-2.994580	0.291404	-2.111792
C	-3.653630	0.872721	-1.002007
C	-4.790811	0.304362	-0.419490
C	-5.249859	-0.885377	-0.955238
C	-4.596315	-1.495621	-2.044764
C	-3.481514	-0.921353	-2.627378
C	-1.897636	1.159524	-2.435070
C	-1.930395	2.186627	-1.524969
H	-5.284955	0.769988	0.426537
H	-6.121328	-1.362168	-0.523353
H	-2.989141	-1.398164	-3.467216
H	-1.214837	1.075851	-3.266497
N	-2.978380	2.020119	-0.658787
C	-3.357517	2.920327	0.413890
H	-4.325740	3.379253	0.204688
H	-3.413362	2.380275	1.360453
H	-2.601615	3.698371	0.500556
H	-4.978145	-2.433692	-2.429075
H	-1.294445	3.055385	-1.438852

$TS_{2/3}$

Symbol	X	Y	Z
Si	1.116841	0.466843	-1.442392
H	-0.324114	0.104175	-0.525631
H	0.989964	1.876130	-1.045349
F	-5.937378	-0.499480	-1.743976
F	-0.592655	-4.421286	2.629810
F	-4.262897	0.610905	-0.011820
C	-4.669504	-0.907821	-1.707652
C	0.750284	-2.587139	3.219902
C	-0.286711	-3.130535	2.487740
C	-3.768745	-0.339282	-0.817202
C	-4.247992	-1.909508	-2.561403
F	2.054276	-0.712200	3.775937
C	1.056196	-1.244674	3.071570
F	-1.987406	-2.939467	0.934953
C	-0.996604	-2.330496	1.601918
C	0.332526	-0.488394	2.171408
C	-0.709173	-0.988966	1.396225
C	-2.438047	-0.725028	-0.737513
C	-2.931489	-2.336773	-2.509932
F	0.698831	0.796361	2.033116
C	-2.071296	-1.742734	-1.606984
B	-1.342994	-0.051089	0.245154
F	-2.515703	-3.311241	-3.318840
F	-0.812272	-2.215117	-1.571869
C	-1.716458	1.467157	0.648339
F	-1.974645	1.075045	2.969139
F	-1.577964	2.072354	-1.629925
C	-1.998371	1.918118	1.928131
C	-1.801205	2.427031	-0.350259
C	-2.323216	3.236218	2.208601
C	-2.117084	3.753433	-0.116434
F	-2.581606	3.623409	3.457416
F	-2.175471	4.633704	-1.115777
C	-2.381835	4.159945	1.180044
F	-5.098883	-2.465870	-3.418277
F	-2.691771	5.428312	1.434549
F	1.457697	-3.344701	4.054202
H	0.373412	-0.074537	-2.588871
C	2.177641	-0.631421	-0.377800
C	3.088923	-0.063219	0.521669
C	2.048210	-2.027404	-0.413566
C	3.854968	-0.868911	1.359664
H	3.199623	1.015741	0.582567
C	2.799267	-2.830258	0.438155
H	1.345783	-2.491107	-1.095479
C	3.704169	-2.251278	1.325709
H	4.552870	-0.413146	2.051835
H	2.674203	-3.906598	0.413503
H	4.282827	-2.877062	1.995350
C	4.755217	0.890746	-1.866592
C	5.834717	1.122130	-1.014607
C	3.758174	1.850135	-2.113829
C	5.897116	2.364492	-0.409899
H	6.580035	0.359716	-0.822945
C	3.843654	3.101051	-1.490380
C	4.913033	3.343096	-0.646132
H	6.716768	2.589167	0.261239
H	3.085305	3.856406	-1.659261
H	4.997940	4.303864	-0.153326
C	2.766192	1.208734	-2.954602
C	3.271554	-0.067673	-3.221148
H	2.030718	1.710660	-3.567937

N	4.419554	-0.266844	-2.558052
C	5.101480	-1.540191	-2.382155
H	6.164642	-1.415688	-2.585827
H	4.955756	-1.891633	-1.357277
H	4.681617	-2.261826	-3.079467
H	2.842071	-0.856750	-3.822690

$TS_{4/5}$

Symbol	X	Y	Z
Si	-3.307944	-2.419349	-1.197257
H	0.448735	0.659384	0.105989
H	-4.627205	-3.084217	-1.088095
F	5.279784	2.408001	-2.689138
F	4.757030	0.590368	4.047629
F	4.162302	0.287699	-1.535545
C	4.170885	2.560839	-1.963469
C	2.591927	-0.238273	4.422600
C	3.554437	0.269951	3.566933
C	3.562040	1.472148	-1.357838
C	3.624060	3.823561	-1.811146
F	0.411096	-1.079071	4.721726
C	1.353150	-0.584665	3.914829
F	4.252386	0.903646	1.453791
C	3.259721	0.433326	2.222001
C	1.108559	-0.405974	2.563623
C	2.026638	0.119051	1.667514
C	2.408192	1.569373	-0.592381
C	2.481030	3.975769	-1.047985
F	-0.112580	-0.783622	2.131094
C	1.916010	2.860044	-0.452585
B	1.606627	0.326805	0.103475
F	1.928036	5.182813	-0.903963
F	0.795006	3.084272	0.264442
C	1.699788	-1.072986	-0.734235
F	3.714280	-1.759713	0.299259
F	-0.330390	-0.643716	-1.909643
C	2.715683	-2.006155	-0.560175
C	0.736032	-1.443053	-1.659897
C	2.771980	-3.212471	-1.237361
C	0.764819	-2.623484	-2.384463
F	3.752559	-4.087637	-1.004494
F	-0.187636	-2.902705	-3.277430
C	1.789745	-3.521754	-2.162350
F	4.191185	4.878578	-2.393046
F	1.823451	-4.682205	-2.813964
F	2.859431	-0.398153	5.717528
H	-3.075109	-2.105256	-2.629877
C	-1.920474	-3.518329	-0.583685
C	-1.185727	-3.228327	0.569038
C	-1.576350	-4.660375	-1.321891
C	-0.115905	-4.030402	0.959345
H	-1.439986	-2.368169	1.176374
C	-0.523591	-5.475964	-0.925528
H	-2.122979	-4.909131	-2.226775
C	0.216907	-5.153715	0.210769
H	0.453158	-3.773859	1.845982
H	-0.265529	-6.350325	-1.511112
H	1.055030	-5.775319	0.505451
C	-4.092193	0.468356	1.592638
C	-4.914400	1.090443	2.527498
C	-4.572221	-0.443677	0.636390
C	-6.263085	0.775432	2.482326
H	-4.521582	1.793938	3.251620
C	-5.937606	-0.740498	0.607461
C	-6.768435	-0.128722	1.533475
H	-6.940807	1.233381	3.192333
H	-6.338301	-1.434030	-0.122890
H	-7.828544	-0.351263	1.529490
C	-3.446058	-0.812881	-0.227732
C	-2.352149	-0.111421	0.331011
H	-3.698878	0.040778	-1.396897
N	-2.730094	0.643668	1.367890
C	-1.865607	1.511018	2.160089
H	-2.365118	2.467473	2.308359
H	-1.656782	1.042776	3.123244
H	-0.934930	1.670001	1.616605
H	-1.317366	-0.116661	0.017778
C	-4.079213	2.116939	-1.472134
C	-2.773651	2.630995	-1.425526
C	-2.392091	3.663156	-0.572764
C	-3.388140	4.199135	0.229967
C	-4.704900	3.712249	0.188849
C	-5.061518	2.664582	-0.645508
C	-4.035966	0.965349	-2.371865
C	-2.742139	0.993511	-2.942090
H	-1.370012	4.019335	-0.527019
H	-3.142973	5.009555	0.905615
H	-6.070428	2.268545	-0.644560

H	-4.889379	0.513213	-2.859515
N	-1.994315	1.913532	-2.340897
C	-0.575193	2.162515	-2.564051
H	-0.422722	3.230690	-2.722559
H	-0.012890	1.822704	-1.696289
H	-0.255787	1.608622	-3.443348
H	-5.452094	4.155700	0.835624
H	-2.311998	0.333926	-3.682631

$TS_{7/8}$

Symbol	X	Y	Z
H	0.060343	-0.069769	1.319645
F	3.974338	-2.394819	-2.498230
F	-2.879485	-3.130448	-1.807467
F	2.021752	-0.567829	-2.232000
C	3.345113	-2.256910	-1.332642
C	-3.372875	-0.834616	-1.902999
C	-2.509543	-1.867940	-1.594933
C	2.336876	-1.322957	-1.170930
C	3.701207	-3.057753	-0.259868
F	-3.830141	1.468754	-1.904469
C	-2.991534	0.469655	-1.637204
F	-0.520281	-2.644228	-0.716356
C	-1.277167	-1.578735	-1.029649
C	-1.760073	0.712291	-1.057559
C	-0.846842	-0.289716	-0.735745
C	1.644971	-1.144293	0.018745
C	3.045549	-2.909321	0.948961
F	-1.504925	1.989971	-0.739581
C	2.041202	-1.960816	1.063076
B	0.495333	-0.008459	0.109204
F	3.382948	-3.675779	1.985686
F	1.438644	-1.873882	2.265447
C	1.178656	1.451077	0.006063
F	0.668868	1.801607	-2.279135
F	1.800797	1.313229	2.282393
C	1.261149	2.211676	-1.153194
C	1.826782	1.981023	1.113187
C	1.937438	3.419446	-1.212253
C	2.507002	3.186838	1.099907
F	1.990118	4.116367	-2.346281
F	3.102570	3.653358	2.196695
C	2.563747	3.909680	-0.078795
F	4.666983	-3.962269	-0.391748
F	3.209789	5.070764	-0.121232
F	-4.574174	-1.088905	-2.409078
H	-0.878834	2.006000	1.805750
C	-2.830522	1.013490	1.858829
C	-2.981344	-0.373302	1.831745
C	-4.121720	-0.998633	1.361667
C	-5.148119	-0.169025	0.901581
C	-5.014934	1.215867	0.915326
C	-3.849028	1.821908	1.394512
C	-1.467984	1.325865	2.422282
C	-0.853207	-0.050503	2.528744
H	-4.216382	-2.077578	1.336176
H	-6.055536	-0.616006	0.514597
H	-3.743685	2.899959	1.389116
H	-1.532727	1.753199	3.428988
N	-1.800559	-0.968611	2.340016
C	-1.616137	-2.406174	2.459844
H	-2.491880	-2.838579	2.942855
H	-1.483589	-2.851439	1.470743
H	-0.731573	-2.599334	3.061079
H	-5.820205	1.833075	0.537916
H	-0.000858	-0.288715	3.153018

$TS_{9/10}$

Symbol	X	Y	Z
F	0.290909	5.314251	-1.677210
F	5.114840	0.789432	-1.806804
F	0.430437	2.676407	-2.202893
C	0.240660	4.431478	-0.683028
C	4.127705	-1.321171	-1.526175
C	4.042625	0.056954	-1.510645
C	0.310145	3.073047	-0.929986
C	0.116126	4.872332	0.625168
F	3.105814	-3.406967	-1.162624
C	3.016941	-2.079805	-1.194842
F	2.858575	2.005668	-1.135536
C	2.848890	0.668187	-1.152069
C	1.849921	-1.433855	-0.847969
C	1.710735	-0.051376	-0.817206
C	0.272382	2.116157	0.074139
C	0.059878	3.952084	1.656209
F	0.800576	-2.198965	-0.499329
C	0.136150	2.599191	1.363423
B	0.306363	0.564716	-0.353856

F	-0.074862	4.370198	2.913266
F	0.043471	1.751983	2.403782
C	-1.014570	0.136103	-1.155971
F	0.093362	-0.890752	-2.986798
F	-2.332658	1.088544	0.555330
C	-1.038302	-0.515563	-2.382098
C	-2.257779	0.493502	-0.646977
C	-2.216484	-0.812454	-3.050216
C	-3.451439	0.243227	-1.294888
F	-2.192429	-1.463758	-4.211252
F	-4.612620	0.618369	-0.763318
C	-3.427808	-0.433903	-2.501725
F	0.045838	6.172884	0.886331
F	-4.565643	-0.736702	-3.114321
F	5.278408	-1.920385	-1.810125
C	-3.370706	-1.523782	1.566871
C	-4.591420	-1.729006	0.923981
C	-2.179458	-2.168202	1.181195
C	-4.592849	-2.596523	-0.154428
H	-5.492225	-1.216530	1.237970
C	-2.208407	-3.037184	0.082314
C	-3.411854	-3.236631	-0.573522
H	-5.515508	-2.777461	-0.692119
H	-1.305148	-3.533340	-0.252140
H	-3.448542	-3.900028	-1.429567
C	-1.121473	-1.716325	2.084505
C	-1.776547	-0.834593	2.955152
H	-0.295779	-0.539293	1.147709
N	-3.077920	-0.705650	2.647514
C	-4.020214	0.232221	3.233965
H	-4.317685	0.964500	2.481407
H	-4.899435	-0.302678	3.593378
H	-3.539921	0.739870	4.067164
H	-1.367771	-0.283586	3.791325
Si	0.311486	-2.811545	2.616101
H	0.318427	-0.003018	0.915434
H	0.207398	-4.077934	1.858361
C	2.021756	-2.098068	2.330497
C	3.056167	-2.940598	1.904320
C	2.311418	-0.743044	2.533864
C	4.335208	-2.443719	1.670622
H	2.860295	-3.994222	1.731847
C	3.582667	-0.236825	2.279670
H	1.538309	-0.063899	2.882297
C	4.595332	-1.087703	1.844448
H	5.120720	-3.108506	1.329300
H	3.782837	0.819341	2.419925
H	5.584229	-0.693687	1.638977
H	0.134104	-3.088735	4.061933

$TS_{11/12}$

Symbol	X	Y	Z
Si	2.937162	2.734236	-1.051452
H	-0.443759	-0.540425	0.227432
H	4.186141	3.507273	-0.864420
F	-4.473593	-3.093682	-3.127275
F	-5.100652	-1.312911	3.648431
F	-3.975731	-0.857268	-1.755357
C	-3.429249	-3.043129	-2.298121
C	-3.157561	-0.148291	4.268184
C	-3.923848	-0.785399	3.306996
C	-3.143933	-1.892854	-1.579543
C	-2.619314	-4.157007	-2.152639
F	-1.195145	1.029995	4.821254
C	-1.944533	0.409527	3.906771
F	-4.265243	-1.469637	1.120637
C	-3.460604	-0.861332	2.002675
C	-1.525882	0.309700	2.590306
C	-2.244965	-0.331773	1.593406
C	-2.070321	-1.784789	-0.707254
C	-1.545802	-4.103839	-1.282185
F	-0.339481	0.889102	2.302983
C	-1.307976	-2.936079	-0.575978
B	-1.630652	-0.432016	0.087118
F	-0.748345	-5.166513	-1.137810
F	-0.252616	-2.960597	0.262563
C	-1.887995	0.925979	-0.779557
F	-4.047682	1.328834	0.100839
F	0.247416	0.758279	-1.814009
C	-3.036197	1.702863	-0.695337
C	-0.931293	1.408329	-1.658982
C	-3.216540	2.878809	-1.405237
C	-1.078102	2.558465	-2.415403
F	-4.325656	3.607230	-1.258628
F	-0.119887	2.954451	-3.256471
C	-2.229239	3.309354	-2.274038
F	-2.873846	-5.268318	-2.841169
F	-2.378011	4.446434	-2.951203

F	-3.587074	-0.068341	5.526340
H	2.800821	2.420605	-2.492771
C	1.423381	3.678088	-0.484633
C	0.668071	3.310760	0.631812
C	0.994315	4.776420	-1.244783
C	-0.501617	3.990455	0.963378
H	0.976693	2.483139	1.259286
C	-0.159820	5.471996	-0.906329
H	1.552827	5.080968	-2.124724
C	-0.917268	5.069965	0.192628
H	-1.082911	3.671437	1.820828
H	-0.481969	6.311851	-1.510126
H	-1.832315	5.595471	0.441250
C	3.923291	0.071296	1.854932
C	4.766412	-0.396389	2.854766
C	4.350494	0.904889	0.812906
C	6.091467	0.008714	2.789421
H	4.411073	-1.048532	3.642916
C	5.686259	1.303869	0.769267
C	6.543743	0.849309	1.761890
H	6.788257	-0.327103	3.547068
H	6.047832	1.951430	-0.020966
H	7.584016	1.150183	1.746313
C	3.202927	1.110401	-0.100183
C	2.146546	0.395775	0.553873
H	3.402604	0.324992	-1.066345
N	2.566906	-0.217128	1.643343
C	1.780653	-1.139135	2.459898
H	2.346777	-2.062601	2.578242
H	1.579693	-0.687275	3.431353
H	0.846616	-1.348113	1.941164
H	1.122402	0.277153	0.226553
H	2.905571	-1.727590	-3.708287
H	1.206454	-2.839420	-2.442296
C	2.780439	-2.601601	-0.953396
C	3.957928	-1.866634	-1.096530
C	5.091981	-2.132487	-0.343572
C	5.023496	-3.176720	0.579150
C	3.856193	-3.919350	0.734490
C	2.723781	-3.636530	-0.033947
C	1.733425	-2.056127	-1.895650
C	2.572026	-1.165580	-2.829077
H	5.998282	-1.548442	-0.451152
H	5.893501	-3.407433	1.182000
H	1.815862	-4.216159	0.080752
H	0.984194	-1.471280	-1.354690
N	3.766783	-0.796539	-2.022885
C	4.927452	-0.351585	-2.791650
H	5.684919	0.045355	-2.113897
H	5.354830	-1.174918	-3.373015
H	4.614145	0.445532	-3.466134
H	3.827451	-4.729861	1.452682
H	2.053759	-0.263319	-3.153952

$TS_{13/14}$

Symbol	X	Y	Z
H	-0.350955	-0.192168	-1.525569
F	-3.725172	-2.937847	2.362300
F	3.323538	-2.507093	0.913290
F	-2.238595	-0.708771	2.038768
C	-3.095358	-2.714681	1.212278
C	2.865126	-0.643207	2.280093
C	2.537804	-1.482228	1.229914
C	-2.324063	-1.583479	1.027874
C	-3.211178	-3.625488	0.173091
F	2.337745	1.206920	3.624553
C	2.024979	0.408743	2.605958
F	1.113773	-2.023864	-0.530370
C	1.387571	-1.220553	0.510539
C	0.876381	0.629064	1.862813
C	0.535262	-0.158291	0.772115
C	-1.658897	-1.303683	-0.157395
C	-2.560512	-3.388197	-1.023953
F	0.100008	1.648708	2.241874
C	-1.799743	-2.237627	-1.167547
B	-0.743761	0.025414	-0.182859
F	-2.667597	-4.261103	-2.022575
F	-1.185940	-2.057708	-2.347630
C	-1.507053	1.439285	-0.209746
F	-3.710134	0.541734	-0.212016
F	0.556737	2.578242	-0.217343
C	-2.888999	1.595323	-0.222065
C	-0.784706	2.626884	-0.232299
C	-3.506332	2.838259	-0.250497
C	-1.357190	3.882538	-0.270802
F	-4.832940	2.930773	-0.259143
F	-0.603637	4.977598	-0.300741
C	-2.738436	3.987130	-0.277711

F	-3.946282	-4.720484	0.327350
F	-3.317681	5.179079	-0.312782
F	3.980830	-0.842050	2.969750
H	0.360020	0.235636	-1.508953
H	2.451704	-0.202212	-4.185162
H	3.803001	-1.957947	-3.401464
C	3.816223	-0.670473	-1.643102
C	3.070783	0.493518	-1.462056
C	3.461164	1.470271	-0.554119
C	4.620822	1.247497	0.187723
C	5.370762	0.085346	0.019690
C	4.970682	-0.879151	-0.904804
C	3.127126	-1.566583	-2.640835
C	2.072905	-0.623616	-3.248147
H	2.882481	2.373161	-0.409675
H	4.937707	1.992080	0.908640
H	5.547465	-1.787356	-1.037262
H	2.668662	-2.414182	-2.131446
N	1.896028	0.496533	-2.276749
C	1.612638	1.765859	-2.963109
H	1.489375	2.567565	-2.240004
H	2.424090	2.022743	-3.652923
H	0.685282	1.659681	-3.529336
H	6.266291	-0.068147	0.608884
H	1.113001	-1.103624	-3.445182

TS$_{15/16}$

Symbol	X	Y	Z
H	-1.088860	0.112110	1.479665
F	-4.229669	2.620009	-2.906204
F	0.914198	-0.992298	-3.814596
F	-3.275721	0.304268	-1.940160
C	-3.484437	2.612304	-1.799842
C	0.961704	-2.604777	-2.106116
C	0.528868	-1.392430	-2.600266
C	-2.983009	1.433685	-1.279678
C	-3.201881	3.808091	-1.156492
F	1.034142	-4.134460	-0.321325
C	0.576970	-2.987003	-0.831718
F	-0.577478	0.616644	-2.356575
C	-0.277605	-0.580586	-1.812194
C	-0.243655	-2.154431	-0.095557
C	-0.720690	-0.926792	-0.546046
C	-2.189032	1.366240	-0.140905
C	-2.430721	3.793979	-0.010563
F	-0.577592	-2.581142	1.135307
C	-1.955768	2.582569	0.474623
B	-1.693850	-0.072541	0.451758
F	-2.135817	4.940044	0.609438
F	-1.200164	2.663371	1.591027
C	-3.055132	-0.896103	0.847100
F	-3.027572	-2.463996	-0.938543
F	-3.347303	0.579926	2.670387
C	-3.608772	-1.970708	0.166427
C	-3.778508	-0.473299	1.955884
C	-4.779262	-2.602475	0.563283
C	-4.950347	-1.071865	2.386532
F	-5.264449	-3.638325	-0.126185
F	-5.599024	-0.623019	3.464488
C	-5.454473	-2.151338	1.681870
F	-3.667684	4.957580	-1.641746
F	-6.580879	-2.745669	2.075629
F	1.777855	-3.377372	-2.822150
H	1.712067	-0.926731	1.908818
C	2.732205	-0.419947	-0.040261
C	2.446750	0.680179	-0.857929
C	2.928225	0.815154	-2.155202
C	3.717034	-0.221793	-2.632119
C	4.009562	-1.339441	-1.836506
C	3.529683	-1.448308	-0.539440
C	2.126323	-0.146723	1.276060
C	1.388210	1.059832	1.072139
H	2.682899	1.671000	-2.771743
H	4.101973	-0.173026	-3.642792
H	3.764310	-2.313106	0.069782
H	3.136629	0.274966	1.969896
N	1.624559	1.559108	-0.128102
C	1.123532	2.825226	-0.654023
H	1.960195	3.416803	-1.026145
H	0.419739	2.621004	-1.461630
H	0.625971	3.361495	0.150224
H	4.619651	-2.134652	-2.246489
H	0.744817	1.581112	1.765833
H	5.123505	2.581121	3.146614
H	5.597416	3.203057	0.885347
C	5.743969	1.028244	0.788029
C	5.403524	0.128536	1.796432
C	5.949719	-1.142143	1.873564

C	6.853298	-1.513165	0.877075
C	7.185621	-0.636198	-0.151509
C	6.634526	0.646267	-0.199637
C	4.976332	2.313480	0.984499
C	4.434483	2.148694	2.414333
H	5.687447	-1.829299	2.668487
H	7.300739	-2.499130	0.907721
H	6.901514	1.331072	-0.996242
H	4.164426	2.389393	0.254827
N	4.376648	0.681120	2.621666
C	4.306224	0.245048	4.015906
H	4.102827	-0.825106	4.053547
H	5.242221	0.459700	4.539053
H	3.485727	0.771195	4.503829
H	7.887368	-0.947880	-0.915297
H	3.441821	2.577946	2.567329

TS$_{17/18}$

Symbol	X	Y	Z
H	-1.023405	-0.200671	-1.537984
F	-1.649089	-2.952207	3.679563
F	0.755968	3.174971	2.752216
F	-2.361235	-0.795914	2.255551
C	-1.111788	-2.737436	2.477848
C	-0.228243	4.017263	0.791890
C	0.044967	2.960625	1.637592
C	-1.466037	-1.635922	1.719121
C	-0.180418	-3.632476	1.975489
F	-1.139483	4.770729	-1.239204
C	-0.906341	3.768499	-0.388041
F	0.001863	0.712468	2.166950
C	-0.359001	1.677833	1.296115
C	-1.294749	2.474639	-0.686233
C	-1.040279	1.375897	0.127276
C	-0.935261	-1.355276	0.466219
C	0.377559	-3.397036	0.733727
F	-1.934179	2.299670	-1.855487
C	-0.003009	-2.273107	0.013802
B	-1.477246	-0.096817	-0.425173
F	1.275410	-4.259624	0.236721
F	0.620322	-2.104161	-1.172892
C	-3.099531	-0.261496	-0.568168
F	-3.723478	1.474390	0.926563
F	-2.743746	-2.071841	-2.047319
C	-4.067447	0.495794	0.075138
C	-3.595045	-1.261996	-1.394829
C	-5.429872	0.298829	-0.100650
C	-4.944672	-1.495720	-1.598120
F	-6.319778	1.060628	0.541165
F	-5.361587	-2.471108	-2.410414
C	-5.872074	-0.702974	-0.944773
F	0.174229	-4.704198	2.681693
F	-7.177375	-0.907281	-1.123431
F	0.177933	5.250190	1.094130
H	1.145281	0.210675	-1.735860
C	2.148762	2.117380	-1.043946
C	2.730454	2.372319	0.211208
C	3.140452	3.641986	0.614026
C	2.929978	4.677424	-0.282065
C	2.337528	4.449892	-1.536050
C	1.953756	3.178246	-1.932157
C	1.879020	0.688468	-1.100241
C	2.204519	0.205688	0.177453
H	3.577647	3.816297	1.589476
H	3.212244	5.685182	-0.004384
H	1.484291	3.013282	-2.895061
H	3.076107	0.176542	-1.809771
N	2.739567	1.178077	0.927372
C	3.266316	1.026569	2.272640
H	4.342303	1.211706	2.275111
H	2.764427	1.730356	2.936454
H	3.073278	0.009713	2.607793
H	2.170204	5.289243	-2.199640
H	2.116422	-0.795550	0.576854
C	4.685988	-1.017407	-1.174907
C	4.108527	-2.296662	-1.166168
C	4.211059	-3.170752	-0.087264
C	4.926730	-2.719195	1.010890
C	5.514985	-1.443640	1.024628
C	5.393725	-0.580648	-0.054549
C	4.212643	-0.356908	-2.387120
C	3.537831	-1.370164	-3.110758
H	3.743388	-4.146504	-0.096474
H	5.036212	-3.363555	1.874093
H	5.831758	0.410690	-0.029931
H	4.666800	0.503477	-2.859741
N	3.428874	-2.472189	-2.378492
C	2.729906	-3.697919	-2.747899

H	3.451296	-4.510747	-2.831629
H	1.989545	-3.930860	-1.985452
H	2.235098	-3.542809	-3.703175
H	6.071405	-1.129673	1.899559
H	3.075255	-1.308050	-4.085515

TS$_{19/20}$

Symbol	X	Y	Z
H	0.367994	-0.188273	-1.587892
F	2.945070	2.913214	2.810347
F	-3.199658	1.004805	2.845970
F	1.580191	0.663089	2.226436
C	2.513582	2.694461	1.570918
C	-3.267341	-1.044545	1.701397
C	-2.624885	0.139882	2.006306
C	1.809238	1.550282	1.249612
C	2.770713	3.626611	0.576923
F	-3.329971	-3.037389	0.458774
C	-2.699362	-1.912892	0.785724
F	-0.905712	1.651200	1.688207
C	-1.421577	0.449476	1.389530
C	-1.497277	-1.570156	0.198698
C	-0.803883	-0.397080	0.481018
C	1.326261	1.284391	-0.024672
C	2.325681	3.394994	-0.711233
F	-0.998101	-2.421943	-0.706238
C	1.623544	2.230973	-0.989478
B	0.591642	-0.126588	-0.276555
F	2.572433	4.285756	-1.669655
F	1.225592	2.059793	-2.262266
C	1.673183	-1.328746	-0.222744
F	0.816586	-2.362081	1.732132
F	2.707782	-0.447258	-2.146415
C	1.726064	-2.314423	0.752754
C	2.685210	-1.370724	-1.173296
C	2.712402	-3.288726	0.779186
C	3.683800	-2.327323	-1.184727
F	2.724710	-4.215703	1.734678
F	4.625535	-2.326736	-2.125369
C	3.694638	-3.295937	-0.195088
F	3.441967	4.736885	0.862756
F	4.643010	-4.225656	-0.181273
F	-4.451093	-1.319955	2.234421
H	-0.391965	0.309505	-1.714354
C	-2.717762	0.186018	-2.234140
C	-3.466630	0.877681	-1.263442
C	-4.657346	0.384875	-0.730113
C	-5.087905	-0.846442	-1.194549
C	-4.352666	-1.559006	-2.159141
C	-3.173921	-1.058406	-2.685071
C	-1.541896	0.993876	-2.506684
C	-1.692183	2.144212	-1.725918
H	-5.212329	0.931024	0.024276
H	-6.002140	-1.273090	-0.800774
H	-2.613565	-1.616884	-3.424943
H	-0.932661	0.955624	-3.399956
N	-2.804994	2.066865	-0.978141
C	-3.251203	3.037209	0.009870
H	-4.248134	3.392468	-0.252237
H	-3.275647	2.581620	1.000880
H	-2.556082	3.873505	0.017768
H	-4.718359	-2.522245	-2.492684
H	-1.056321	3.016243	-1.672697

Si2-1

Symbol	X	Y	Z
F	1.808070	-3.357489	-3.350903
F	-5.964587	-1.481127	-0.854785
F	0.672253	-0.987059	-3.191684
C	0.910582	-3.132572	-2.392630
C	-5.296332	0.234915	0.603008
C	-5.001075	-0.693290	-0.382197
C	0.311084	-1.888952	-2.271634
C	0.591986	-4.137626	-1.500157
F	-4.559184	1.942814	2.045575
C	-4.294467	1.056014	1.087315
F	-3.476151	-1.707295	-1.807279
C	-3.705920	-0.791169	-0.859297
C	-3.016252	0.936119	0.568608
C	-2.668685	0.006358	-0.397454
C	-0.613375	-1.581179	-1.276982
C	-0.352364	-3.895876	-0.518320
F	-2.081049	1.757951	1.075754
C	-0.950649	-2.650180	-0.444745
B	-1.170824	-0.102650	-0.966106
F	-0.676829	-4.857262	0.343333
F	-1.895726	-2.508283	0.490492
C	-0.734090	1.119903	-1.920394

F	-2.938595	1.699912	-2.582597
F	1.534395	0.715231	-1.439802
C	-1.622655	1.933612	-2.610579
C	0.603469	1.462048	-2.061724
C	-1.210198	3.017997	-3.374654
C	1.058284	2.531164	-2.804519
F	-2.101673	3.767809	-4.017929
F	2.360849	2.804098	-2.884891
C	0.134972	3.323159	-3.468012
F	1.181145	-5.323917	-1.585289
F	0.540459	4.357725	-4.192584
F	-6.532409	0.332996	1.079674
C	4.007320	-1.549769	0.067273
C	3.983618	-2.338140	-1.088308
C	3.763644	-2.084339	1.356597
C	3.717890	-3.686750	-0.930758
H	4.154027	-1.908639	-2.069278
C	3.496379	-3.457801	1.479957
C	3.473295	-4.242131	0.341098
H	3.681022	-4.327152	-1.803943
H	3.311545	-3.894417	2.455776
H	3.261100	-5.301677	0.420591
C	3.841875	-0.987795	2.277770
C	4.138781	0.125270	1.535350
H	3.705883	-1.018360	3.347410
N	4.235875	-0.200495	0.203749
C	4.684130	0.672490	-0.866571
H	5.603162	0.281561	-1.307994
H	3.919996	0.759354	-1.640914
H	4.882845	1.659238	-0.451153
H	4.284793	1.149361	1.847759
Si	0.634718	0.016405	1.313476
H	-0.464251	0.266025	0.164565
H	1.632869	-0.700625	0.518466
C	-0.288006	-1.110022	2.458885
C	-1.479671	-0.735796	3.095739
C	0.178431	-2.421222	2.615565
C	-2.182453	-1.650576	3.869966
H	-1.871158	0.269820	2.978391
C	-0.524479	-3.335950	3.392247
H	1.087011	-2.736290	2.110745
C	-1.705129	-2.950764	4.017395
H	-3.105776	-1.353397	4.353033
H	-0.156524	-4.349501	3.497606
H	-2.259076	-3.664319	4.615941
C	1.618552	3.819085	2.503049
C	2.219720	5.072647	2.371397
C	1.715010	2.813860	1.515418
C	2.938621	5.312025	1.213616
H	2.125361	5.826369	3.144175
C	2.460380	3.085977	0.359913
C	3.059990	4.325445	0.218272
H	3.417043	6.273519	1.072172
H	2.579031	2.334645	-0.407765
H	3.630870	4.540804	-0.677471
C	0.977721	1.667211	2.004229
C	0.493664	2.050063	3.244062
N	0.866802	3.319539	3.546329
C	0.548542	4.050257	4.759634
H	1.464395	4.324329	5.285679
H	-0.013073	4.953417	4.516143
H	-0.057787	3.415895	5.402462
H	-0.095551	1.483068	3.950588

Si2-2

Symbol	X	Y	Z
F	-1.914684	-2.225650	-4.232750
F	-6.434547	1.050476	-0.046498
F	-1.872156	0.119770	-2.979839
C	-1.759888	-2.188846	-2.905317
C	-5.451712	0.548616	2.031277
C	-5.336976	0.808398	0.674887
C	-1.720479	-0.980067	-2.224911
C	-1.607789	-3.370858	-2.206327
F	-4.419311	0.044545	4.081405
C	-4.313375	0.297213	2.773372
F	-4.016161	1.060564	-1.221867
C	-4.081658	0.806191	0.095522
C	-3.073187	0.310555	2.149083
C	-2.908967	0.567722	0.797765
C	-1.510288	-0.881558	-0.856566
C	-1.400496	-3.327878	-0.840379
F	-2.016433	0.060994	2.940728
C	-1.373061	-2.100184	-0.202936
B	-1.466181	0.486538	0.044822
F	-1.210560	-4.464099	-0.163777
F	-1.192419	-2.125717	1.126834
C	-1.045138	1.820067	-0.798054

F	-2.570258	3.288517	0.278004
F	0.638872	0.621291	-1.977103
C	-1.564931	3.090959	-0.590073
C	0.003361	1.786765	-1.706267
C	-1.090745	4.234105	-1.220252
C	0.507176	2.894515	-2.359019
F	-1.631490	5.426308	-0.960844
F	1.563377	2.776945	-3.178086
C	-0.038140	4.139667	-2.110466
F	-1.611049	-4.542154	-2.847952
F	0.460523	5.225793	-2.700778
F	-6.651144	0.541574	2.613775
C	2.670260	-2.084512	-2.272229
C	1.913099	-2.584820	-3.322307
C	2.843385	-2.751809	-1.054551
C	1.334609	-3.830115	-3.123801
H	1.777785	-2.037451	-4.246769
C	2.230600	-3.988632	-0.864756
C	1.493384	-4.520737	-1.915047
H	0.732476	-4.267493	-3.910326
H	2.321022	-4.519936	0.074571
H	1.013673	-5.484125	-1.792298
C	3.626250	-1.851193	-0.175450
C	3.990315	-0.763342	-1.052378
H	4.397108	-2.227609	0.494311
N	3.393324	-0.873779	-2.213221
C	3.438710	0.092747	-3.308592
H	3.875865	-0.387177	-4.183414
H	2.424941	0.426592	-3.524442
H	4.047593	0.940403	-3.001968
H	4.612138	0.092253	-0.820525
Si	2.271510	-0.959998	1.005754
H	-0.612241	0.351295	0.887793
H	1.186601	-0.740967	0.035262
C	1.870265	-2.146969	2.383999
C	0.756793	-1.823327	3.173690
C	2.621398	-3.279328	2.714294
C	0.401110	-2.618321	4.256208
H	0.147462	-0.958230	2.929411
C	2.266005	-4.076063	3.799457
H	3.494310	-3.555007	2.130610
C	1.155332	-3.745917	4.569077
H	-0.468871	-2.360230	4.848472
H	2.854766	-4.952656	4.042381
H	0.876643	-4.367818	5.411674
C	3.694541	2.759519	1.974487
C	4.014476	4.084411	1.670200
C	3.171599	1.864783	1.013951
C	3.796285	4.507127	0.369743
H	4.411281	4.757146	2.421324
C	2.958675	2.325316	-0.292868
C	3.271302	3.638186	-0.603977
H	4.027865	5.529749	0.097816
H	2.532792	1.665352	-1.041795
H	3.099616	4.008255	-1.608177
C	2.956470	0.599587	1.679922
C	3.348706	0.806622	2.987038
N	3.789649	2.081892	3.171770
C	4.263003	2.661279	4.415674
H	5.302837	2.977169	4.315328
H	3.648971	3.521626	4.686509
H	4.190285	1.911378	5.200475
H	3.329344	0.116291	3.819265

Si2-3

Symbol	X	Y	Z
F	-6.213127	-2.395605	1.288582
F	-4.838025	3.771714	-0.982271
F	-4.334565	-1.476532	-0.404205
C	-5.138367	-1.759297	1.760285
C	-2.900975	3.406411	-2.257751
C	-3.754788	3.033780	-1.239365
C	-4.153873	-1.284273	0.912823
C	-5.000510	-1.564751	3.125387
F	-0.919475	3.001594	-3.459807
C	-1.775105	2.640259	-2.500515
F	-4.352791	1.643248	0.502391
C	-3.470084	1.908115	-0.475945
C	-1.531864	1.535871	-1.708861
C	-2.351794	1.109134	-0.671410
C	-3.023124	-0.611152	1.351015
C	-3.889724	-0.899259	3.609946
F	-0.396088	0.855908	-1.987831
C	-2.928607	-0.437763	2.721100
B	-1.881915	-0.114429	0.304660
F	-3.755368	-0.709175	4.925733
F	-1.882927	0.208685	3.262926
C	-1.322835	-1.451069	-0.464191

F	-2.314592	-1.067124	-2.581582
F	-0.204375	-2.059503	1.535844
C	-1.548937	-1.821972	-1.781728
C	-0.516517	-2.328365	0.251438
C	-0.994643	-2.954681	-2.362498
C	0.051848	-3.470641	-0.284931
F	-1.205135	-3.236069	-3.649808
F	0.875672	-4.235648	0.444016
C	-0.188245	-3.788084	-1.609583
F	-5.933871	-2.014374	3.964356
F	0.366268	-4.868972	-2.156386
F	-3.132596	4.507793	-2.972635
C	2.798060	-2.157085	-0.821507
C	3.254331	-3.264475	-1.516888
C	2.091982	-1.105106	-1.407617
C	2.966264	-3.294343	-2.877255
H	3.787449	-4.070996	-1.029887
C	1.818298	-1.148588	-2.769073
C	2.257605	-2.256028	-3.489930
H	3.285395	-4.143106	-3.468166
H	1.259203	-0.354935	-3.248857
H	2.038308	-2.320001	-4.548410
C	1.778619	-0.117821	-0.337996
C	2.295522	-0.771663	0.855518
H	0.722182	0.147713	-0.229875
N	2.889096	-1.894740	0.570047
C	3.585646	-2.773310	1.504137
H	3.145605	-3.767013	1.438285
H	4.641254	-2.800031	1.231124
H	3.469906	-2.376503	2.509153
H	2.245557	-0.414695	1.876258
Si	2.583892	1.662345	-0.610160
H	-0.956379	0.321924	0.948675
H	2.957020	1.690756	-2.040225
C	1.251477	2.916846	-0.255257
C	0.973976	3.938603	-1.171970
C	0.455131	2.811876	0.892554
C	-0.073540	4.827162	-0.949403
H	1.564017	4.031011	-2.077939
C	-0.604309	3.686768	1.108237
H	0.637959	2.025733	1.620051
C	-0.868632	4.694755	0.185412
H	-0.286806	5.604188	-1.674617
H	-1.228383	3.572251	1.986859
H	-1.701086	5.370660	0.345019
C	5.858351	1.235396	1.805035
C	7.006886	0.552154	2.213959
C	5.127427	0.866781	0.653095
C	7.415638	-0.520192	1.440020
H	7.556076	0.848029	3.099893
C	5.574995	-0.215514	-0.119919
C	6.709553	-0.899581	0.281832
H	8.301810	-1.073872	1.725281
H	5.050493	-0.503779	-1.026936
H	7.070185	-1.733801	-0.308262
C	4.004184	1.770821	0.552343
C	4.123034	2.621931	1.629647
N	5.224623	2.316506	2.377213
C	5.674031	3.001866	3.575378
H	5.701582	2.309851	4.418695
H	6.669288	3.421429	3.420135
H	4.978883	3.808003	3.799184
H	3.480174	3.439588	1.925379

Si2-4

Symbol	X	Y	Z
Si	-2.735385	-0.311647	-0.838961
H	1.869174	0.523343	0.136417
F	5.377840	-0.588428	-4.382106
F	6.619388	-0.452861	3.009000
F	3.940897	-1.769094	-2.478506
C	4.903660	0.139476	-3.369401
C	4.532219	-1.129573	3.840672
C	5.322455	-0.683793	2.799132
C	4.143784	-0.446985	-2.366979
C	5.173763	1.494683	-3.317173
F	2.401253	-1.797517	4.598689
C	3.187843	-1.369560	3.608136
F	5.612453	-0.041363	0.599998
C	4.762641	-0.475184	1.543739
C	2.680894	-1.153361	2.342005
C	3.422372	-0.693972	1.260737
C	3.619674	0.260381	-1.295661
C	4.690452	2.240169	-2.255176
F	1.368556	-1.421353	2.174914
C	3.946371	1.608768	-1.274657
B	2.652949	-0.364512	-0.141223
F	4.939470	3.550617	-2.192682

F	3.528192	2.383016	-0.254434
C	1.754093	-1.594864	-0.732480
F	2.661246	-3.338792	0.600549
F	0.683242	-0.053240	-2.174036
C	1.804274	-2.926007	-0.347797
C	0.823786	-1.321300	-1.727917
C	0.986900	-3.910121	-0.883524
C	0.007322	-2.274797	-2.311976
F	1.049128	-5.164215	-0.432617
F	-0.868350	-1.952101	-3.268227
C	0.079555	-3.584703	-1.872452
F	5.894997	2.075737	-4.274219
F	-0.719364	-4.514056	-2.393211
F	5.053326	-1.326738	5.049997
H	-1.914760	-0.075060	-2.045160
C	-2.343577	-1.930275	0.008870
C	-1.329436	-2.079296	0.961497
C	-3.043971	-3.074817	-0.400277
C	-1.022579	-3.327080	1.494722
H	-0.744604	-1.228058	1.287599
C	-2.731726	-4.325640	0.121632
H	-3.839201	-2.988451	-1.134599
C	-1.722159	-4.452822	1.071375
H	-0.221917	-3.416375	2.219838
H	-3.273694	-5.200996	-0.215496
H	-1.474833	-5.427781	1.473938
C	-2.136607	1.439038	2.567612
C	-2.504760	1.591605	3.895029
C	-3.042801	1.442248	1.506211
C	-3.860827	1.771586	4.139421
H	-1.779119	1.577063	4.698327
C	-4.394560	1.634786	1.767865
C	-4.788242	1.799158	3.091929
H	-4.203975	1.896742	5.158535
H	-5.119580	1.652263	0.963615
H	-5.836630	1.948720	3.318296
C	-2.267915	1.240325	0.253256
C	-0.891002	1.223459	0.726250
H	-2.415528	2.011788	-0.516095
N	-0.834180	1.303336	2.028633
C	0.368343	1.258527	2.857600
H	0.377274	2.140798	3.494974
H	0.345384	0.351864	3.462576
H	1.241770	1.257567	2.207345
H	0.012560	1.118950	0.134478
C	-1.520295	4.458510	0.082037
C	-0.195760	4.019967	-0.169899
C	0.804639	4.035655	0.809564
C	0.441830	4.475970	2.069768
C	-0.880278	4.872629	2.358547
C	-1.859147	4.870028	1.381595
C	-2.238494	4.321834	-1.151586
C	-1.354400	3.805327	-2.063590
H	1.815264	3.713064	0.587100
H	1.189567	4.513356	2.853668
H	-2.871276	5.183213	1.611607
H	-3.271143	4.570733	-1.338529
N	-0.126409	3.609886	-1.479599
C	1.069300	3.089069	-2.120190
H	1.883372	3.814159	-2.048608
H	1.378293	2.154033	-1.648397
H	0.848834	2.896694	-3.168367
H	-1.127303	5.194092	3.363342
H	-1.497984	3.555976	-3.104343
C	-6.814047	-0.324987	-1.279506
C	-8.113463	-0.604076	-0.846855
C	-5.671195	-0.634399	-0.508514
C	-8.250677	-1.205591	0.391495
H	-8.977026	-0.360586	-1.454455
C	-5.843768	-1.244970	0.743676
C	-7.127104	-1.521933	1.179301
H	-9.241312	-1.438430	0.762831
H	-4.984722	-1.491030	1.358894
H	-7.274797	-1.993086	2.143483
C	-4.517038	-0.197280	-1.263721
C	-5.025939	0.334945	-2.430498
N	-6.387410	0.266085	-2.449784
C	-7.262208	0.713257	-3.517717
H	-7.846491	-0.124261	-3.902226
H	-7.939372	1.487532	-3.153174
H	-6.653956	1.121250	-4.322010
H	-4.499233	0.760784	-3.273301

Si2-5

Symbol	X	Y	Z
F	-1.432253	-2.038638	-3.399431
F	-7.444157	-1.718255	-0.456940
F	-2.473327	0.116462	-2.271597

C	-1.575942	-2.000310	-2.068056
C	-7.116534	-1.106035	1.791456
C	-6.666598	-1.172109	0.482902
C	-2.127622	-0.889917	-1.447343
C	-1.162771	-3.076358	-1.308151
F	-6.740290	-0.466357	4.021046
C	-6.310768	-0.534087	2.757086
F	-5.042174	-0.739945	-1.113172
C	-5.417201	-0.665754	0.174550
C	-5.063265	-0.038272	2.400816
C	-4.568702	-0.082780	1.106875
C	-2.324594	-0.792278	-0.078125
C	-1.332152	-3.030363	0.064516
F	-4.341366	0.500182	3.395306
C	-1.907956	-1.907893	0.639305
B	-3.069201	0.435333	0.718953
F	-0.948004	-4.072186	0.811199
F	-2.061521	-1.940679	1.975317
C	-3.035366	1.826191	-0.142012
F	-5.328947	1.915949	-0.769604
F	-0.723251	2.018068	0.348362
C	-4.091584	2.435021	-0.806009
C	-1.824213	2.494432	-0.269804
C	-3.959230	3.591503	-1.562505
C	-1.638929	3.635453	-1.030522
F	-5.014746	4.122503	-2.185000
F	-0.428620	4.194233	-1.149452
C	-2.722110	4.194634	-1.684936
F	-0.605813	-4.138300	-1.887584
F	-2.573346	5.296821	-2.420001
F	-8.317059	-1.589130	2.114186
C	1.445738	-1.419364	-2.797846
C	1.713656	-2.330250	-3.824177
C	1.737429	-1.676265	-1.430703
C	2.285325	-3.538874	-3.467882
H	1.464852	-2.103357	-4.854254
C	2.290463	-2.932330	-1.106192
C	2.559317	-3.836606	-2.118892
H	2.495803	-4.277196	-4.232056
H	2.490587	-3.202752	-0.076784
H	2.971101	-4.806946	-1.866641
C	1.314641	-0.513653	-0.671622
C	0.775119	0.335893	-1.613968
H	3.354171	0.656703	-1.810675

N	0.862155	-0.180021	-2.877774
C	0.349536	0.433178	-4.090210
H	1.159883	0.619085	-4.797801
H	-0.395866	-0.216689	-4.549172
H	-0.120520	1.378932	-3.826744
H	0.295734	1.293269	-1.480542
Si	1.401152	-0.207613	1.175797
H	-2.458919	0.630043	1.737173
H	0.033146	-0.066412	1.722278
C	2.150511	-1.742766	1.967686
C	1.314610	-2.757026	2.444172
C	3.534530	-1.961920	1.989740
C	1.837384	-3.968055	2.893480
H	0.240229	-2.606013	2.455763
C	4.064879	-3.162062	2.452616
H	4.203834	-1.180506	1.641022
C	3.213076	-4.173555	2.894573
H	1.170513	-4.746351	3.245831
H	5.139658	-3.310688	2.474922
H	3.621945	-5.112612	3.249421
C	3.640801	3.206224	1.958789
C	4.138580	4.484188	1.694331
C	2.747627	2.529542	1.090436
C	3.720988	5.099197	0.525836
H	4.825298	4.973934	2.375623
C	2.336461	3.190894	-0.075682
C	2.823197	4.458565	-0.347820
H	4.081404	6.092326	0.285989
H	1.634750	2.727602	-0.754268
H	2.497528	4.973545	-1.243861
C	2.500366	1.220571	1.655203
C	3.247598	1.193859	2.814031
N	3.921230	2.368138	3.009628
C	4.808262	2.678344	4.112092
H	5.836544	2.796934	3.761427
H	4.489822	3.598310	4.605244
H	4.771667	1.861933	4.830563
H	3.342790	0.402111	3.544495
C	5.294564	0.720930	-0.838445
C	5.846146	-0.482750	-0.411982
C	6.694761	-0.605059	0.673362
C	6.990305	0.575590	1.351620
C	6.456913	1.797189	0.936912
C	5.602247	1.888154	-0.160558

C 4.411914 0.434098 -2.017959
C 4.580429 -1.033315 -2.180912
H 7.101342 -1.558070 0.988647
H 7.646877 0.542737 2.212087
H 5.181806 2.841709 -0.460386
H 4.687120 0.965987 -2.933899
N 5.373923 -1.514752 -1.281279
C 5.728822 -2.920538 -1.107392
H 6.811608 -2.997337 -1.019802
H 5.245946 -3.286914 -0.199426
H 5.372613 -3.479986 -1.967814
H 6.709124 2.700253 1.480321
H 4.136240 -1.672409 -2.936411

Si2-6

Symbol	X	Y	Z
C	3.585887	-0.025620	-3.829454
C	3.568684	0.635643	-5.060345
C	3.924422	0.623625	-2.619157
C	3.907878	1.977716	-5.062094
H	3.306035	0.119155	-5.976354
C	4.269889	1.982735	-2.656641
C	4.257503	2.644023	-3.872342
H	3.909552	2.525005	-5.997221
H	4.549502	2.500376	-1.745234
H	4.525060	3.693112	-3.914559
C	3.847930	-0.361925	-1.563894
C	3.482801	-1.531317	-2.190888
N	3.317200	-1.343316	-3.537875
C	2.944456	-2.349036	-4.512090
H	2.008686	-2.076515	-5.004397
H	3.726855	-2.457535	-5.266110
H	2.812319	-3.300352	-4.000390
H	3.334259	-2.515199	-1.766594
Si	4.238203	-0.159284	0.237697
H	3.135505	0.506495	0.981825
C	4.477365	-1.886073	0.950507
C	3.379437	-2.721833	1.194635
C	5.759590	-2.391736	1.195420
C	3.555351	-4.021763	1.659567
H	2.371238	-2.353730	1.023564
C	5.941279	-3.691033	1.661430
H	6.627006	-1.761436	1.020720
C	4.839101	-4.507860	1.892576
H	2.694133	-4.653436	1.844678
H	6.941916	-4.065219	1.845179
H	4.978804	-5.519100	2.256789
C	7.851978	1.805442	0.156313
C	9.127446	2.025001	-0.371522
C	6.972158	0.825984	-0.360146
C	9.512870	1.236850	-1.442383
H	9.787746	2.780044	0.039030
C	7.391688	0.046150	-1.448921
C	8.653556	0.258062	-1.976222
H	10.494188	1.377270	-1.880095
H	6.731383	-0.705894	-1.868780
H	8.989285	-0.336183	-2.817655
C	5.758251	0.877903	0.424318
C	5.971931	1.873619	1.349247
N	7.213520	2.432857	1.201640
C	7.780365	3.505093	1.994975
H	8.681385	3.164511	2.509183
H	8.031308	4.356840	1.359662
H	7.047292	3.819328	2.735239
H	5.313249	2.240162	2.124356

Si2-7

Symbol	X	Y	Z
F	-1.668815	-2.336071	-3.706986
F	-7.527073	-0.673280	-0.884199
F	-2.312865	-0.020557	-2.581225
C	-1.763150	-2.258929	-2.375754
C	-7.024958	-0.720728	1.414924
C	-6.636977	-0.518955	0.099828
C	-2.114082	-1.066092	-1.758703
C	-1.516412	-3.381185	-1.606438
F	-6.474828	-0.746540	3.698408
C	-6.101870	-0.553947	2.429877
F	-4.995204	0.038485	-1.450116
C	-5.328279	-0.159676	-0.163270
C	-4.798510	-0.187953	2.117889
C	-4.365785	0.022281	0.819350
C	-2.263485	-0.929333	-0.386561
C	-1.610541	-3.287071	-0.228554
F	-3.963300	-0.039238	3.156044
C	-1.998674	-2.084957	0.339282
B	-2.824925	0.379489	0.426568
F	-1.354710	-4.358037	0.528297

F	-2.130935	-2.074485	1.678179
C	-2.650303	1.794402	-0.370364
F	-4.821489	2.672790	0.035656
F	-0.422773	1.193785	-0.889294
C	-3.578573	2.822990	-0.446956
C	-1.413641	2.115880	-0.907371
C	-3.300531	4.070185	-0.995016
C	-1.081574	3.342565	-1.447279
F	-4.237880	5.018730	-1.030493
F	0.159311	3.582609	-1.894680
C	-2.040051	4.339762	-1.492513
F	-1.212326	-4.544611	-2.181748
F	-1.745431	5.537539	-1.995554
F	-8.279922	-1.071806	1.697274
C	1.632530	-1.315678	-2.339249
C	1.277674	-2.088311	-3.434313
C	2.035372	-1.844308	-1.111858
C	1.335724	-3.464668	-3.260693
H	0.960179	-1.647915	-4.370846
C	2.077875	-3.227846	-0.952442
C	1.728273	-4.022789	-2.038836
H	1.058187	-4.115372	-4.080069
H	2.381680	-3.675017	-0.013325
H	1.749862	-5.100477	-1.935341
C	2.396343	-0.700380	-0.230749
C	2.197781	0.449688	-1.098189
H	3.426276	-0.740943	0.162275
N	1.731577	0.092667	-2.262368
C	1.293185	0.979140	-3.333623
H	1.795120	0.691819	-4.256429
H	0.211421	0.879765	-3.440517
H	1.552349	2.001048	-3.071397
H	2.355733	1.494789	-0.859155
Si	1.500645	-0.548559	1.498306
H	-2.175564	0.490281	1.437248
H	0.046155	-0.397709	1.313670
C	1.940593	-2.195869	2.255426
C	0.944559	-3.124730	2.573616
C	3.283792	-2.586828	2.366167
C	1.280143	-4.411384	2.989004
H	-0.101222	-2.851927	2.483426
C	3.621089	-3.867562	2.787880
H	4.076899	-1.893849	2.093172
C	2.615920	-4.782635	3.096407

H	0.496961	-5.123183	3.221048
H	4.662563	-4.157285	2.866595
H	2.876236	-5.784863	3.415616
C	3.212488	2.963905	2.763146
C	3.395709	4.347680	2.692369
C	2.223313	2.288754	2.012554
C	2.560917	5.055201	1.844818
H	4.159567	4.847650	3.276037
C	1.385580	3.038136	1.173262
C	1.563263	4.407440	1.091771
H	2.673054	6.129384	1.763108
H	0.602539	2.547323	0.608796
H	0.923330	4.992764	0.441844
C	2.341781	0.878218	2.310097
C	3.376716	0.789312	3.218189
N	3.901144	2.020630	3.492878
C	5.007813	2.308967	4.386179
H	5.832301	2.760956	3.830851
H	4.689355	2.989873	5.176918
H	5.347864	1.377810	4.834870
H	3.782516	-0.083803	3.710517
H	7.257013	0.378393	0.703093
H	6.154916	2.514650	0.390335
C	5.267056	1.239512	-1.157010
C	5.323418	-0.160201	-1.240281
C	5.061040	-0.822547	-2.434659
C	4.743068	-0.049078	-3.554760
C	4.687491	1.339410	-3.482839
C	4.948958	1.992705	-2.272145
C	5.522528	1.634160	0.279510
C	6.166473	0.357111	0.842077
H	5.097510	-1.903457	-2.503423
H	4.541281	-0.546129	-4.497073
H	4.904209	3.074607	-2.209800
H	4.569054	1.831458	0.784792
N	5.576377	-0.712807	0.020107
C	6.180979	-2.029275	0.101862
H	5.517496	-2.771962	-0.346167
H	7.155528	-2.066826	-0.403273
H	6.319265	-2.292045	1.151478
H	4.447635	1.917389	-4.367156
H	5.946513	0.190999	1.898452

Si2-8

Symbol	X	Y	Z
F	0.951904	-0.823041	3.641733
F	7.356481	-0.659963	1.793025
F	2.104792	0.955238	2.063264
C	1.280915	-1.175009	2.392311
C	7.355627	-1.162261	-0.506212
C	6.719199	-0.662348	0.618676
C	1.905269	-0.270465	1.545715
C	1.002590	-2.452795	1.949720
F	7.306417	-1.636738	-2.806529
C	6.694785	-1.156361	-1.719629
F	4.859761	0.313295	1.611385
C	5.431786	-0.173219	0.497746
C	5.402451	-0.651791	-1.793409
C	4.725305	-0.143381	-0.696841
C	2.317478	-0.585270	0.258427
C	1.372420	-2.812368	0.664767
F	4.830828	-0.679233	-3.006488
C	2.025179	-1.886747	-0.134083
B	3.180504	0.375133	-0.759677
F	1.121945	-4.050879	0.229487
F	2.403148	-2.315248	-1.352511
C	2.973198	1.955352	-0.402856
F	5.226645	2.583555	0.025226
F	0.673427	1.616264	-0.798282
C	3.924352	2.900486	-0.052436
C	1.677774	2.448470	-0.457236
C	3.614077	4.222542	0.246297
C	1.316306	3.746088	-0.156238
F	4.575424	5.086100	0.583410
F	0.031280	4.126863	-0.198162
C	2.301208	4.651709	0.199586
F	0.398506	-3.327378	2.749809
F	1.987576	5.914187	0.491590
F	8.596697	-1.642350	-0.417203
C	-1.884996	-0.955021	2.697067
C	-2.088608	-1.532960	3.952859
C	-2.075849	-1.656965	1.481847
C	-2.530390	-2.841430	3.983847
H	-1.914828	-0.970148	4.862559
C	-2.500603	-2.997241	1.553234
C	-2.738546	-3.564182	2.792662
H	-2.704425	-3.325354	4.937266
H	-2.623933	-3.585606	0.652469
H	-3.063627	-4.595953	2.851851
C	-1.770483	-0.738256	0.397449
C	-1.437950	0.446649	1.032081
H	-3.950488	-0.088595	0.007793
N	-1.502566	0.328172	2.388527
C	-1.225095	1.378559	3.352170
H	-2.117202	1.589920	3.944742
H	-0.407889	1.077059	4.007202
H	-0.933079	2.276276	2.809977
H	-1.112957	1.384759	0.606761
Si	-1.552565	-1.026010	-1.442849
H	2.755845	0.206971	-1.874020
H	-0.190891	-0.595060	-1.831446
C	-1.724752	-2.843498	-1.883663
C	-0.581707	-3.562727	-2.249382
C	-2.950785	-3.525635	-1.869611
C	-0.655664	-4.914634	-2.577579
H	0.382952	-3.067573	-2.273062
C	-3.033093	-4.872404	-2.202431
H	-3.859236	-3.006888	-1.577810
C	-1.880183	-5.570340	-2.556676
H	0.245488	-5.452326	-2.847555
H	-3.990742	-5.379505	-2.183074
H	-1.939792	-6.621422	-2.813443
C	-3.983204	1.667407	-3.436344
C	-4.378145	2.967855	-3.766005
C	-2.917033	1.393694	-2.547065
C	-3.662634	4.008785	-3.200360
H	-5.202731	3.153488	-4.444301
C	-2.197840	2.474502	-2.011661
C	-2.575505	3.764449	-2.339473
H	-3.933539	5.030588	-3.437014
H	-1.339758	2.307314	-1.374577
H	-2.018003	4.599771	-1.933417
C	-2.814506	-0.045887	-2.415007
C	-3.801891	-0.545716	-3.238176
N	-4.509505	0.461965	-3.841472
C	-5.601795	0.306764	-4.784263
H	-6.503526	0.792358	-4.405969
H	-5.335690	0.745577	-5.747239
H	-5.799077	-0.754507	-4.919890
H	-4.045249	-1.573250	-3.469534
H	-6.686361	0.901566	-0.375829
H	-5.908553	3.153059	0.057326

C	-5.058285	1.904538	1.630204
C	-4.941248	0.523186	1.684272
C	-4.851918	-0.205856	2.850688
C	-4.880953	0.523868	4.039606
C	-4.989442	1.912426	4.021639
C	-5.075195	2.616729	2.819579
C	-5.177100	2.358232	0.195165
C	-5.614313	1.064877	-0.494270
H	-4.754791	-1.285948	2.857175
H	-4.815082	-0.002222	4.983730
H	-5.170705	3.695607	2.816132
H	-4.213194	2.700768	-0.194609
N	-4.939872	-0.020880	0.311635
C	-5.560897	-1.360021	0.145985
H	-4.939862	-2.099139	0.650507
H	-6.559172	-1.332514	0.579094
H	-5.612388	-1.571667	-0.921507
H	-5.014656	2.454839	4.958919
H	-5.325210	0.961102	-1.535710

Si2-9

Symbol	X	Y	Z
F	-6.048037	-0.516612	2.538895
F	-4.307900	3.807436	-1.982806
F	-4.526748	-0.574464	0.310487
C	-4.759330	-0.196679	2.600873
C	-2.534823	2.852662	-3.188801
C	-3.337529	2.900794	-2.064497
C	-3.962500	-0.224074	1.472511
C	-4.196357	0.174429	3.812639
F	-0.677948	1.921947	-4.296766
C	-1.502474	1.932177	-3.252469
F	-3.909372	2.154970	0.043881
C	-3.108035	2.021916	-1.016030
C	-1.308052	1.077548	-2.187707
C	-2.102003	1.064442	-1.047684
C	-2.616521	0.112524	1.488916
C	-2.855264	0.507353	3.874831
F	-0.266322	0.236589	-2.258793
C	-2.092168	0.468010	2.718378
B	-1.815406	-0.014950	0.103009
F	-2.310078	0.857099	5.036694
F	-0.791793	0.782010	2.833307
C	-1.604962	-1.558866	-0.321385
F	-2.529978	-1.370994	-2.497030
F	-0.630677	-1.971728	1.789536
C	-1.954154	-2.111265	-1.546423
C	-1.040157	-2.436743	0.598372
C	-1.727116	-3.442976	-1.854356
C	-0.823654	-3.774260	0.334571
F	-2.037739	-3.922998	-3.054472
F	-0.244937	-4.566137	1.235346
C	-1.159583	-4.277760	-0.909153
F	-4.942117	0.207342	4.909948
F	-0.920549	-5.548899	-1.197477
F	-2.723278	3.712970	-4.180714
C	2.137009	-2.888590	-0.207304
C	2.130021	-4.214592	-0.652537
C	1.943460	-1.785443	-1.074046
C	1.904169	-4.426862	-2.000930
H	2.274569	-5.041053	0.033408
C	1.714040	-2.038916	-2.436387
C	1.692567	-3.348930	-2.882469
H	1.880500	-5.439743	-2.384374
H	1.549034	-1.219453	-3.125585
H	1.510141	-3.553516	-3.930601
C	2.053777	-0.579112	-0.274893
C	2.292500	-1.031472	1.008383
H	-0.320933	-0.034838	0.297932
N	2.329542	-2.393814	1.064107
C	2.546632	-3.197006	2.253458
H	1.660125	-3.789541	2.480655
H	3.400259	-3.860395	2.103724
H	2.758490	-2.530727	3.087202
H	2.492164	-0.462264	1.906421
Si	2.468541	1.146869	-0.850934
H	-0.570295	0.639116	0.615222
H	2.588830	1.118925	-2.329790
C	1.186945	2.464195	-0.429708
C	0.686559	3.332202	-1.406216
C	0.696340	2.597165	0.877949
C	-0.290273	4.277956	-1.097482
H	1.052502	3.261896	-2.426931
C	-0.303377	3.514667	1.187181
H	1.087007	1.961928	1.669084
C	-0.800074	4.356831	0.193855
H	-0.665168	4.940788	-1.869767
H	-0.686936	3.577257	2.199139

H	-1.579475	5.072788	0.427811
C	6.134595	1.617887	0.969748
C	7.373751	1.139793	1.404900
C	5.197863	0.803838	0.290009
C	7.666457	-0.188026	1.145325
H	8.077108	1.780921	1.923464
C	5.525785	-0.539192	0.042491
C	6.751598	-1.018775	0.470811
H	8.617387	-0.595927	1.466572
H	4.828555	-1.189356	-0.476573
H	7.016620	-2.052590	0.283731
C	4.042273	1.625720	0.003108
C	4.344557	2.864936	0.520666
N	5.586753	2.874659	1.095370
C	6.229249	3.998636	1.747264
H	6.434825	3.766271	2.794014
H	7.166344	4.245416	1.244605
H	5.564243	4.858459	1.698513
H	3.744282	3.764659	0.528904

Si2-TS1

Symbol	X	Y	Z
F	3.632252	-0.688185	-3.425948
F	-2.962581	-5.280109	-1.023023
F	1.103876	0.020967	-3.215564
C	2.877077	-1.250963	-2.479509
C	-3.818243	-3.670223	0.460857
C	-2.906689	-4.049778	-0.509074
C	1.547601	-0.880256	-2.325544
C	3.425515	-2.203946	-1.644707
F	-4.629379	-2.013508	1.921850
C	-3.760145	-2.390039	0.980755
F	-1.103347	-3.574363	-1.884643
C	-1.949010	-3.144976	-0.936177
C	-2.791578	-1.516279	0.514955
C	-1.841940	-1.857059	-0.435262
C	0.716179	-1.392965	-1.336265
C	2.627373	-2.783654	-0.674295
F	-2.796520	-0.281244	1.049968
C	1.304430	-2.392090	-0.560477
B	-0.726278	-0.772661	-0.888763
F	3.139579	-3.712271	0.135879
F	0.584238	-3.034782	0.368510
C	-1.361093	0.383272	-1.851707
F	-3.302276	-0.825315	-2.509234
F	0.494363	1.782132	-1.433516
C	-2.582249	0.305795	-2.507925
C	-0.722760	1.608155	-1.987627
C	-3.141619	1.368748	-3.207688
C	-1.238284	2.694545	-2.664151
F	-4.323097	1.234207	-3.811344
F	-0.564855	3.847959	-2.720595
C	-2.473592	2.575904	-3.278633
F	4.699357	-2.568433	-1.780542
F	-3.001123	3.604887	-3.936211
F	-4.745382	-4.527737	0.884918
C	3.953916	1.709963	0.172283
C	4.675433	1.410418	-0.985431
C	3.988896	0.908341	1.329551
C	5.491415	0.295427	-0.941868
H	4.600105	2.021615	-1.876513
C	4.858571	-0.192357	1.355468
C	5.597278	-0.484272	0.224450
H	6.063480	0.018433	-1.818726
H	4.968920	-0.792324	2.250493
H	6.265396	-1.336268	0.229508
C	3.039296	1.487933	2.262523
C	2.617277	2.683029	1.664722
H	2.950734	1.272767	3.318248
N	3.115694	2.789293	0.421770
C	2.837993	3.851119	-0.536936
H	3.779967	4.268686	-0.892371
H	2.268064	3.454682	-1.378772
H	2.262593	4.628569	-0.038488
H	1.960137	3.445884	2.057473
Si	0.936490	0.599441	1.480318
H	-0.463661	-0.154422	0.175546
H	1.596990	0.678656	0.182778
C	0.893151	-1.016439	2.406401
C	-0.348635	-1.532377	2.807035
C	2.033413	-1.799036	2.608409
C	-0.439321	-2.785933	3.399689
H	-1.254451	-0.963568	2.632389
C	1.945000	-3.053094	3.200970
H	2.997845	-1.448835	2.269239
C	0.708021	-3.546948	3.599064
H	-1.408056	-3.171864	3.695362
H	2.839696	-3.648898	3.335491

H	0.635126	-4.528250	4.053135
C	-1.676653	3.555296	2.646931
C	-2.358995	4.766140	2.511149
C	-0.785464	3.062065	1.666790
C	-2.126503	5.500360	1.361419
H	-3.043424	5.115856	3.274978
C	-0.561601	3.840278	0.522431
C	-1.232630	5.042405	0.377799
H	-2.639631	6.443312	1.217701
H	0.121891	3.507700	-0.245053
H	-1.070412	5.635130	-0.514583
C	-0.278524	1.793321	2.150403
C	-0.878819	1.615626	3.385117
N	-1.707569	2.649107	3.685539
C	-2.512365	2.789621	4.885613
H	-2.255058	3.714549	5.404184
H	-3.573029	2.800967	4.629689
H	-2.312157	1.945579	5.541952
H	-0.760119	0.806081	4.090947

Si2-TS2

Symbol	X	Y	Z
Si	-2.941436	-0.582399	-0.941057
H	1.761591	0.598398	0.065341
F	6.549138	-0.226497	-3.195396
F	5.969815	-1.152706	3.696806
F	4.707726	-1.521184	-1.765910
C	5.708430	0.461599	-2.421048
C	3.701577	-0.973009	4.278428
C	4.703630	-0.957490	3.323741
C	4.734276	-0.184584	-1.676575
C	5.814372	1.840651	-2.356448
F	1.407990	-0.803577	4.787320
C	2.389333	-0.789321	3.882026
F	5.402491	-0.778313	1.125742
C	4.379722	-0.746087	1.992200
C	2.116584	-0.585925	2.539701
C	3.082262	-0.533372	1.546500
C	3.833624	0.479420	-0.855690
C	4.949417	2.547900	-1.541447
F	0.814883	-0.437165	2.218481
C	3.998371	1.856512	-0.808223
B	2.639245	-0.225839	0.004475
F	5.030926	3.879371	-1.476727
F	3.185461	2.610706	-0.039269
C	2.007915	-1.549116	-0.716709
F	3.520840	-3.053454	0.309061
F	0.366279	-0.269077	-1.870397
C	2.465498	-2.842229	-0.490423
C	0.930377	-1.467632	-1.584545
C	1.889536	-3.967230	-1.055032
C	0.339110	-2.560816	-2.198307
F	2.344498	-5.189601	-0.771105
F	-0.691753	-2.405437	-3.032542
C	0.818316	-3.825590	-1.920548
F	6.739941	2.478224	-3.070750
F	0.254184	-4.898693	-2.470656
F	3.997137	-1.170443	5.561960
H	-2.422821	-0.358515	-2.315567
C	-2.245195	-2.183502	-0.253302
C	-1.324320	-2.231729	0.796898
C	-2.617749	-3.389593	-0.864487
C	-0.765224	-3.439566	1.207729
H	-1.029079	-1.323062	1.306199
C	-2.069430	-4.598793	-0.453579
H	-3.337971	-3.383484	-1.677725
C	-1.134272	-4.624042	0.579465
H	-0.042263	-3.450581	2.015924
H	-2.360285	-5.519934	-0.944336
H	-0.692242	-5.564566	0.888226
C	-2.306775	2.273972	1.961471
C	-2.715154	3.141444	2.971562
C	-3.200938	1.659075	1.064901
C	-4.073195	3.396315	3.066835
H	-2.004222	3.602198	3.646500
C	-4.567227	1.944492	1.175801
C	-4.986559	2.806166	2.177503
H	-4.437249	4.062773	3.839121
H	-5.281161	1.498344	0.494390
H	-6.041682	3.028786	2.280328
C	-2.400164	0.898968	0.097862
C	-1.071329	1.098109	0.532966
H	-2.401713	1.834585	-1.054250
N	-1.016241	1.906460	1.599408
C	0.192028	2.344419	2.287353
H	0.143738	3.422049	2.438934
H	0.275678	1.834154	3.248036
H	1.054463	2.104324	1.666159

H	-0.156689	0.702143	0.113123
C	-1.845357	3.859197	-1.115174
C	-0.450269	3.757558	-1.231243
C	0.435790	4.502558	-0.458651
C	-0.130909	5.394607	0.439761
C	-1.523787	5.516741	0.571171
C	-2.391956	4.745613	-0.186697
C	-2.407942	2.831085	-1.991798
C	-1.300651	2.319376	-2.711164
H	1.508903	4.383245	-0.545683
H	0.515769	6.004087	1.059178
H	-3.465110	4.817606	-0.052828
H	-3.421032	2.812663	-2.372194
N	-0.166862	2.802331	-2.215703
C	1.185963	2.416753	-2.600614
H	1.761738	3.315609	-2.823238
H	1.644956	1.864597	-1.782048
H	1.131645	1.781217	-3.480729
H	-1.926518	6.218696	1.291056
H	-1.284987	1.571658	-3.491071
C	-7.015352	-0.924182	-0.569890
C	-8.168216	-1.231294	0.158244
C	-5.716095	-1.078699	-0.036237
C	-7.993156	-1.693886	1.450924
H	-9.157048	-1.112111	-0.268701
C	-5.571655	-1.547443	1.277814
C	-6.708384	-1.849095	2.006277
H	-8.862054	-1.942098	2.048489
H	-4.584270	-1.672193	1.710671
H	-6.613527	-2.213193	3.022074
C	-4.779865	-0.658094	-1.053234
C	-5.556014	-0.288153	-2.128992
N	-6.887616	-0.437362	-1.852656
C	-7.993405	-0.179082	-2.754508
H	-8.526674	-1.104332	-2.981014
H	-8.687240	0.533848	-2.305775
H	-7.601606	0.241973	-3.678054
H	-5.251629	0.076638	-3.100429

Si2-TS3

Symbol	X	Y	Z
F	-1.525133	-1.794982	-3.882369
F	-7.474179	-0.662306	-0.937163
F	-2.250703	0.361986	-2.527907
C	-1.649385	-1.871587	-2.552890
C	-6.969101	-0.940269	1.345056
C	-6.590392	-0.580332	0.061296
C	-2.033449	-0.759992	-1.816157
C	-1.401638	-3.069672	-1.907880
F	-6.415776	-1.197678	3.613472
C	-6.052341	-0.850340	2.375299
F	-4.972357	0.204018	-1.413102
C	-5.297471	-0.142120	-0.155865
C	-4.764967	-0.400278	2.110145
C	-4.343019	-0.027796	0.844817
C	-2.206671	-0.773231	-0.439875
C	-1.521331	-3.126317	-0.530037
F	-3.934034	-0.339576	3.160488
C	-1.932819	-1.996824	0.159770
B	-2.816696	0.427968	0.499105
F	-1.275753	-4.273827	0.109448
F	-2.097153	-2.136374	1.487665
C	-2.694715	1.914209	-0.167961
F	-4.926514	2.658861	0.175050
F	-0.422220	1.455953	-0.604075
C	-3.671745	2.895954	-0.238245
C	-1.452663	2.328741	-0.622168
C	-3.435724	4.179435	-0.719322
C	-1.161896	3.590570	-1.098262
F	-4.421088	5.078921	-0.760858
F	0.081437	3.902744	-1.492476
C	-2.171755	4.535979	-1.149767
F	-1.083258	-4.161634	-2.601657
F	-1.927495	5.765605	-1.601319
F	-8.209334	-1.368728	1.582919
C	1.655922	-1.279944	-2.353215
C	1.443854	-2.050086	-3.492701
C	2.054898	-1.811238	-1.116714
C	1.621314	-3.416915	-3.360444
H	1.144836	-1.604745	-4.433363
C	2.213794	-3.199020	-1.007677
C	1.992136	-3.982540	-2.130537
H	1.454601	-4.061243	-4.214416
H	2.510165	-3.655811	-0.070581
H	2.103731	-5.057381	-2.057780
C	2.292450	-0.679246	-0.193437
C	1.971299	0.450312	-0.998897
H	3.639488	-0.553248	-0.020250

N	1.597062	0.108255	-2.224421
C	1.105329	1.009489	-3.257950
H	1.678058	0.865973	-4.173377
H	0.048934	0.799233	-3.436768
H	1.218326	2.033819	-2.909241
H	1.997916	1.495774	-0.724100
Si	1.618054	-0.716055	1.594361
H	-2.170470	0.460371	1.516753
H	0.152088	-0.522732	1.542067
C	1.975121	-2.438184	2.230372
C	0.933055	-3.368827	2.310232
C	3.280937	-2.882481	2.476419
C	1.189300	-4.705258	2.606419
H	-0.088129	-3.054318	2.125858
C	3.542604	-4.214345	2.778406
H	4.108675	-2.182793	2.421514
C	2.493691	-5.129774	2.836245
H	0.370037	-5.412991	2.651616
H	4.559460	-4.540619	2.963465
H	2.694725	-6.170294	3.062368
C	3.535375	2.569457	3.137917
C	3.863546	3.928742	3.117850
C	2.533427	2.015918	2.307740
C	3.152347	4.740257	2.251555
H	4.638216	4.332515	3.759104
C	1.799267	2.876607	1.474917
C	2.120503	4.221671	1.445018
H	3.379050	5.798510	2.207218
H	0.976199	2.496632	0.880389
H	1.561424	4.889854	0.801013
C	2.497211	0.592820	2.563278
C	3.433676	0.382863	3.552071
N	4.061585	1.549363	3.897398
C	5.082021	1.714790	4.915694
H	5.993535	2.122655	4.474941
H	4.730317	2.386853	5.700196
H	5.301958	0.742991	5.352587
H	3.710183	-0.536195	4.049245
H	6.621532	0.653008	0.593883
H	5.842310	2.790758	-0.133544
C	4.951299	1.460791	-1.611435
C	4.958327	0.069187	-1.584096
C	4.807202	-0.705191	-2.722646
C	4.642338	-0.033646	-3.933595
C	4.625245	1.359075	-3.981662
C	4.780520	2.117788	-2.820524
C	5.101056	1.995126	-0.207317
C	5.531056	0.740839	0.570307
H	4.797242	-1.788335	-2.679921
H	4.521842	-0.605715	-4.845570
H	4.769824	3.200600	-2.863591
H	4.152039	2.389581	0.168484
N	4.993669	-0.401628	-0.227539
C	5.670011	-1.686468	-0.005717
H	5.055189	-2.495212	-0.403646
H	6.649173	-1.689146	-0.491352
H	5.798855	-1.835384	1.064690
H	4.493872	1.858837	-4.933846
H	5.137049	0.692129	1.585273

Si2-TS4

Symbol	X	Y	Z
F	-5.354461	-1.005012	3.413012
F	-4.403998	4.179618	-0.601210
F	-4.406352	-0.679134	0.912709
C	-4.078588	-0.681258	3.219356
C	-2.763590	3.583415	-2.170756
C	-3.470112	3.324009	-1.013005
C	-3.570869	-0.510208	1.944865
C	-3.237256	-0.510374	4.307321
F	-1.067632	2.968402	-3.677460
C	-1.778768	2.701632	-2.585385
F	-3.891907	2.019999	0.848495
C	-3.178744	2.188208	-0.270254
C	-1.519714	1.585544	-1.819363
C	-2.202589	1.277651	-0.648372
C	-2.251892	-0.159739	1.694428
C	-1.911063	-0.175134	4.102669
F	-0.536853	0.767281	-2.231515
C	-1.444135	-0.009223	2.807947
B	-1.780648	-0.036287	0.161494
F	-1.096494	-0.022631	5.144722
F	-0.140156	0.286606	2.671223
C	-1.797463	-1.445214	-0.599234
F	-3.020165	-0.714522	-2.496620
F	-0.559143	-2.392704	1.173332
C	-2.399560	-1.690614	-1.826434
C	-1.204788	-2.546872	0.006225

C	-2.394407	-2.940470	-2.425776
C	-1.200943	-3.813377	-0.545001
F	-2.958067	-3.124420	-3.617984
F	-0.621571	-4.831625	0.086730
C	-1.787692	-4.004671	-1.783533
F	-3.701991	-0.671896	5.541170
F	-1.748622	-5.199405	-2.359625
F	-2.992263	4.693486	-2.862636
C	1.955634	-2.830498	-0.528644
C	1.934163	-4.118560	-1.065304
C	1.626708	-1.678954	-1.273076
C	1.539330	-4.240916	-2.385971
H	2.194100	-4.985311	-0.470075
C	1.228182	-1.834021	-2.608521
C	1.184157	-3.110021	-3.145140
H	1.495882	-5.222542	-2.841501
H	0.952031	-0.972043	-3.203957
H	0.869594	-3.244846	-4.173399
C	1.786803	-0.524279	-0.393740
C	2.206252	-1.081516	0.818933
H	0.162104	-0.093553	0.108174
N	2.286833	-2.422562	0.753088
C	2.612562	-3.330286	1.838271
H	1.799431	-4.046626	1.967437
H	3.539286	-3.861104	1.615676
H	2.736321	-2.754580	2.752497
H	2.473603	-0.572059	1.735091
Si	2.426585	1.145094	-0.978503
H	-0.459961	0.372012	0.391246
H	2.576740	1.035589	-2.450070
C	1.332256	2.630058	-0.637383
C	1.156917	3.605486	-1.626786
C	0.694111	2.814614	0.596235
C	0.356549	4.722080	-1.400168
H	1.636800	3.486007	-2.593124
C	-0.127833	3.915359	0.819490
H	0.827699	2.087868	1.393191
C	-0.298722	4.869206	-0.181317
H	0.224347	5.462353	-2.181234
H	-0.633972	4.030216	1.771302
H	-0.942750	5.724637	-0.010806
C	6.095874	1.087305	0.878352
C	7.265663	0.437759	1.281846
C	5.094127	0.445123	0.114003
C	7.418158	-0.885284	0.903643
H	8.022631	0.946915	1.866883
C	5.280085	-0.896221	-0.256714
C	6.435750	-1.546107	0.141646
H	8.311861	-1.422513	1.197598
H	4.532520	-1.414647	-0.850890
H	6.593118	-2.580523	-0.139850
C	4.036436	1.406385	-0.103767
C	4.453831	2.553212	0.532670
N	5.679612	2.378286	1.116266
C	6.421814	3.360643	1.882024
H	6.554981	3.022058	2.911431
H	7.401148	3.530886	1.431299
H	5.866279	4.296116	1.884500
H	3.949417	3.506123	0.617890

PhNMe$_2$

Symbol	X	Y	Z
C	-0.186179	-0.000015	-0.083422
C	0.544424	-1.204849	-0.036784
C	0.544774	1.204758	-0.040052
C	1.931624	-1.194660	0.020810
H	0.030726	-2.155879	-0.045480
C	1.931955	1.194400	0.017589
H	0.031240	2.155848	-0.052725
C	2.643841	-0.000193	0.045748
H	2.459795	-2.141112	0.052321
H	2.460352	2.140812	0.046310
H	3.725356	-0.000271	0.092940
N	-1.566891	0.000427	-0.178278
C	-2.277900	1.240631	0.073796
H	-3.346786	1.060060	-0.020464
H	-2.077280	1.644406	1.075650
H	-2.006333	2.000622	-0.662300
C	-2.278565	-1.240451	0.067835
H	-3.346782	-1.061208	-0.036381
H	-1.999522	-1.999527	-0.666333
H	-2.086371	-1.644469	1.071279

add2

Symbol	X	Y	Z
F	3.325068	-2.073519	-2.510428
F	-1.844798	-4.481684	-0.806898
F	1.056351	-0.879220	-1.893413

C	3.065054	-1.791920	-1.235812
C	-3.148239	-2.596375	-1.320052
C	-2.004801	-3.159681	-0.789147
C	1.883274	-1.171304	-0.878270
C	3.985531	-2.117722	-0.255708
F	-4.416334	-0.663922	-1.729574
C	-3.305582	-1.225428	-1.255770
F	-0.008456	-3.000166	0.331959
C	-1.041029	-2.341902	-0.219981
C	-2.315736	-0.443473	-0.683704
C	-1.126804	-0.950224	-0.155830
C	1.518451	-0.846091	0.432567
C	3.691781	-1.803535	1.053904
F	-2.599380	0.860701	-0.638665
C	2.490626	-1.178674	1.363957
B	0.067243	-0.040173	0.542763
F	4.562896	-2.092602	2.018766
F	2.337730	-0.914715	2.675523
C	0.432463	1.383361	-0.211517
F	-0.797265	1.057079	-2.239374
F	2.123812	1.794438	1.407496
C	-0.003933	1.804300	-1.469452
C	1.447738	2.187314	0.314175
C	0.402516	2.992790	-2.062839
C	1.878886	3.379007	-0.241274
F	-0.078645	3.343607	-3.253448
F	2.832496	4.097326	0.347306
C	1.332227	3.800095	-1.438941
F	5.129715	-2.713275	-0.571940
F	1.728482	4.935466	-2.000338
F	-4.088958	-3.360063	-1.861867
C	-1.902764	0.842605	2.034733
C	-1.994642	2.200822	1.731871
C	-3.062601	0.101840	2.231355
C	-3.237739	2.807063	1.626351
H	-1.111856	2.795689	1.546455
C	-4.306251	0.717561	2.112305
H	-3.031846	-0.953287	2.459572
C	-4.400635	2.068357	1.812608
H	-3.291791	3.861467	1.386183
H	-5.200981	0.125410	2.258740
H	-5.370025	2.542309	1.723268
N	-0.552155	0.214514	2.137001
C	-0.650377	-1.125716	2.804967
H	0.352545	-1.499845	2.970607
H	-1.155893	-1.004755	3.762556
H	-1.201476	-1.825348	2.187894
C	0.211978	1.074470	3.114405
H	1.186223	0.644385	3.294916
H	0.323364	2.081256	2.736234
H	-0.370174	1.101703	4.035399

N1-1

Symbol	X	Y	Z
Si	0.630622	0.009676	2.656498
H	-0.124288	0.003150	1.307928
H	0.195310	-1.250562	3.282262
F	-4.559612	2.785529	-1.095610
F	3.312361	1.939829	-2.704963
F	-3.262735	0.467044	-1.170878
C	-3.328210	2.709617	-0.599994
C	3.589559	-0.172280	-1.717698
C	2.805215	0.931075	-2.001087
C	-2.631785	1.510037	-0.623811
C	-2.733604	3.835254	-0.059420
F	3.805898	-2.287525	-0.715335
C	3.058333	-1.222834	-0.988287
F	0.802009	2.074773	-1.859367
C	1.498516	0.980304	-1.539499
C	1.756447	-1.131298	-0.538641
C	0.927969	-0.039382	-0.785838
C	-1.339348	1.384460	-0.129765
C	-1.445315	3.755441	0.446722
F	1.303611	-2.142242	0.214997
C	-0.784195	2.545281	0.393042
B	-0.523667	0.011487	-0.134363
F	-0.865818	4.833256	0.965758
F	0.467031	2.507975	0.880560
C	-1.399370	-1.322118	-0.095726
F	-0.490431	-2.195259	-2.097899
F	-2.446300	-0.622769	1.899391
C	-1.334202	-2.310134	-1.067662
C	-2.319702	-1.534697	0.922916
C	-2.127693	-3.445118	-1.034942
C	-3.121752	-2.658610	0.997657
F	-2.036424	-4.366165	-1.989898
F	-3.980835	-2.822717	1.999314
C	-3.025061	-3.619200	0.004121

F	-3.388050	4.987729	-0.030007
F	-3.789632	-4.702365	0.050459
F	4.849798	-0.219737	-2.126472
H	0.101851	1.218880	3.307451
C	2.441057	-0.001339	2.258023
C	3.161933	-1.192792	2.421044
C	3.096079	1.115049	1.717382
C	4.497011	-1.272289	2.040834
H	2.675872	-2.070371	2.834083
C	4.427091	1.029980	1.325957
H	2.568818	2.053009	1.592128
C	5.125631	-0.165066	1.480460
H	5.038927	-2.201761	2.164496
H	4.918177	1.894440	0.894533
H	6.159479	-0.231933	1.162773

N1-2

Symbol	X	Y	Z
Si	-0.732056	0.244429	-1.579105
H	0.556010	0.040237	-0.684858
H	-0.514623	-0.816687	-2.574817
F	5.416989	3.067350	-0.337552
F	-0.625001	1.126304	4.667693
F	4.434740	0.620818	-0.034000
C	4.106635	2.906447	-0.173405
C	-1.240505	-0.885496	3.624770
C	-0.472109	0.262764	3.665398
C	3.566326	1.637511	-0.018003
C	3.275228	4.010816	-0.156590
F	-1.796845	-2.900661	2.550006
C	-1.070279	-1.786636	2.587143
F	1.161916	1.633112	2.778102
C	0.448356	0.508349	2.657241
C	-0.152789	-1.498400	1.596052
C	0.629571	-0.346706	1.577504
C	2.207228	1.416334	0.160296
C	1.911969	3.838745	0.025253
F	-0.057342	-2.372972	0.584298
C	1.418288	2.559448	0.185026
B	1.545911	-0.038975	0.302836
F	1.105249	4.896099	0.049412
F	0.093503	2.434623	0.378334
C	2.396554	-1.239170	-0.332248
F	2.646667	-2.418671	1.702991
F	2.284924	-0.212623	-2.453612
C	2.889962	-2.313357	0.391992
C	2.716071	-1.225719	-1.683141
C	3.654611	-3.316152	-0.182468
C	3.467745	-2.210148	-2.297467
F	4.112472	-4.327103	0.552042
F	3.742585	-2.152728	-3.598164
C	3.944070	-3.263424	-1.534781
F	3.777276	5.229619	-0.308649
F	4.674696	-4.217675	-2.099073
F	-2.142841	-1.122944	4.568979
H	-0.478190	1.614792	-2.038372
C	-2.111526	-0.109590	-0.406298
C	-2.688707	-1.385893	-0.427530
C	-2.553928	0.816959	0.547553
C	-3.676904	-1.733475	0.485946
H	-2.358156	-2.118777	-1.156260
C	-3.541429	0.468406	1.461651
H	-2.129773	1.813941	0.579799
C	-4.102694	-0.806409	1.431512
H	-4.101545	-2.730044	0.469469
H	-3.871701	1.189254	2.201091
H	-4.867796	-1.077844	2.150152
C	-5.131964	0.372631	-1.983775
C	-4.569269	1.669020	-1.981483
C	-4.589005	-0.574527	-2.881282
C	-3.489764	1.973454	-2.793024
H	-4.964780	2.433638	-1.327408
C	-3.508151	-0.250655	-3.685523
H	-5.004278	-1.571203	-2.939770
C	-2.932104	1.018501	-3.644848
H	-3.073387	2.974409	-2.758039
H	-3.109454	-1.002873	-4.357641
H	-2.102681	1.272137	-4.294370
N	-6.162205	0.044639	-1.143865
C	-6.757621	-1.273769	-1.216581
H	-7.523734	-1.361543	-0.449391
H	-7.221726	-1.462046	-2.192266
H	-6.007915	-2.050680	-1.036007
C	-6.756805	1.060301	-0.298351
H	-7.545027	0.607019	0.298480
H	-6.015499	1.486129	0.385430
H	-7.194330	1.876627	-0.885381

N1-3

Symbol	X	Y	Z
Si	-1.611254	0.145268	-2.204888
H	0.496484	0.095546	-0.856907
H	-1.006253	-1.027944	-2.857225
F	5.420259	3.107290	-0.014502
F	-0.243301	1.098639	4.622902
F	4.353678	0.687857	0.187096
C	4.095737	2.966363	-0.104299
C	-0.840991	-0.963774	3.669997
C	-0.166701	0.238728	3.602384
C	3.508023	1.711345	-0.014035
C	3.299145	4.081208	-0.279696
F	-1.422489	-3.002419	2.653884
C	-0.761925	-1.842032	2.603685
F	1.195034	1.741044	2.495632
C	0.572469	0.549396	2.467995
C	-0.030394	-1.483689	1.487170
C	0.667845	-0.286627	1.366097
C	2.140685	1.499340	-0.100522
C	1.925193	3.924506	-0.352519
F	-0.027575	-2.362244	0.468974
C	1.386877	2.654490	-0.257926
B	1.400975	0.046725	-0.051100
F	1.142745	4.996948	-0.504048
F	0.042371	2.575184	-0.291357
C	2.378923	-1.156810	-0.548887
F	3.149585	-1.708496	1.618698
F	1.802277	-0.758858	-2.812590
C	3.142251	-1.947364	0.297570
C	2.483317	-1.469598	-1.894947
C	3.938568	-2.990642	-0.143163
C	3.269500	-2.502528	-2.383168
F	4.650483	-3.724149	0.716728
F	3.328829	-2.763286	-3.692672
C	4.001350	-3.271369	-1.497404
F	3.843216	5.294967	-0.364835
F	4.764561	-4.269850	-1.943110
F	-1.586626	-1.266010	4.735057
H	-0.832949	1.384828	-2.317175
C	-2.344687	-0.231787	-0.538810
C	-2.854594	-1.511285	-0.284138
C	-2.504683	0.758722	0.440090
C	-3.527911	-1.790629	0.900426
H	-2.722186	-2.302902	-1.015040
C	-3.174871	0.481147	1.627265
H	-2.095989	1.750648	0.278739
C	-3.699918	-0.789399	1.851919
H	-3.903775	-2.789558	1.087570
H	-3.282145	1.253451	2.380935
H	-4.214981	-1.006878	2.781039
C	-5.380602	0.363127	-1.596096
C	-4.788020	1.648767	-1.868110
C	-5.077624	-0.720536	-2.497151
C	-3.766731	1.745774	-2.752346
H	-5.143991	2.529467	-1.354317
C	-4.044375	-0.604965	-3.365675
H	-5.651741	-1.634494	-2.457954
C	-3.207077	0.575922	-3.399492
H	-3.301313	2.709302	-2.929242
H	-3.791497	-1.439112	-4.010957
H	-2.682279	0.757970	-4.337626
N	-6.190655	0.188194	-0.564005
C	-6.822310	-1.110654	-0.319504
H	-7.312086	-1.081964	0.648883
H	-7.567109	-1.330449	-1.087399
H	-6.065934	-1.895942	-0.303736
C	-6.491433	1.295237	0.347136
H	-7.013769	0.900208	1.213182
H	-5.563969	1.760973	0.682788
H	-7.124898	2.040256	-0.139388

N1-4

Symbol	X	Y	Z
F	3.275803	4.570197	-1.287859
F	5.439019	-1.550769	-1.427068
F	2.123234	2.188889	-1.784857
C	2.841518	3.810277	-0.279124
C	3.526102	-2.898543	-1.622732
C	4.120888	-1.701851	-1.274960
C	2.240334	2.586435	-0.506956
C	2.994635	4.249329	1.026573
F	1.567665	-4.192986	-1.776255
C	2.158722	-3.036707	-1.462458
F	4.028273	0.446024	-0.448601
C	3.345698	-0.666161	-0.766894
C	1.433317	-1.981714	-0.943831
C	1.972063	-0.754137	-0.578534

C	1.781922	1.754341	0.503446
C	2.547114	3.458369	2.068144
F	0.115954	-2.199246	-0.781831
C	1.951736	2.235002	1.790069
B	1.022057	0.354589	0.157169
F	2.688758	3.882434	3.327388
F	1.532279	1.526923	2.851199
C	-0.345525	0.782902	-0.649030
F	0.088670	-0.268199	-2.732463
F	-1.022467	1.937426	1.305229
C	-0.706795	0.477525	-1.953717
C	-1.255437	1.594769	0.025881
C	-1.889707	0.911219	-2.541672
C	-2.425634	2.078123	-0.530665
F	-2.208554	0.554133	-3.788370
F	-3.266416	2.848005	0.169241
C	-2.750520	1.723910	-1.828001
F	3.569075	5.426574	1.274661
F	-3.897147	2.147730	-2.368968
F	4.255310	-3.906924	-2.100500
H	-1.250181	-0.483397	1.936339
Si	-2.126022	-2.352441	3.072607
H	0.665387	-0.149339	1.198382
H	-3.377890	-3.134758	3.025795
C	-0.661668	-3.312296	2.438388
C	-0.828283	-4.494395	1.706967
C	0.628600	-2.781879	2.576111
C	0.265397	-5.130303	1.128054
H	-1.819105	-4.919439	1.579450
C	1.722550	-3.417678	1.999785
H	0.786630	-1.860464	3.129095
C	1.539575	-4.592553	1.275972
H	0.124115	-6.039973	0.557056
H	2.713609	-2.990893	2.103882
H	2.389787	-5.083066	0.815601
H	-1.897858	-1.735643	4.394278
C	-4.515122	-0.149001	0.164742
C	-4.356899	0.422530	1.482400
C	-3.581438	-1.160564	-0.258251
C	-3.280410	0.112516	2.237261
H	-5.072981	1.146156	1.842358
C	-2.496562	-1.439623	0.496950
H	-3.700107	-1.643044	-1.217083
C	-2.278873	-0.859353	1.816623
H	-3.148814	0.602687	3.196019
H	-1.762314	-2.144990	0.129192
N	-5.465067	0.275632	-0.650983
C	-5.631188	-0.322896	-1.980793
H	-4.736157	-0.168950	-2.586386
H	-5.833896	-1.390756	-1.893505
H	-6.470630	0.155261	-2.474233
C	-6.337872	1.394222	-0.274414
H	-6.953962	1.653807	-1.128935
H	-6.985791	1.115352	0.558083
H	-5.734909	2.260456	-0.001351

N1-5

Symbol	X	Y	Z
F	2.209794	4.932505	-1.723947
F	5.137208	-0.926910	-1.955954
F	1.368648	2.408841	-2.189135
C	1.916742	4.127133	-0.707814
C	3.447326	-2.548892	-1.813521
C	3.876929	-1.243143	-1.671730
C	1.479338	2.834813	-0.924530
C	2.052117	4.580378	0.595596
F	1.733201	-4.140775	-1.582306
C	2.140689	-2.878479	-1.495868
F	3.502239	0.951635	-1.068185
C	2.998450	-0.277278	-1.203473
C	1.293267	-1.888513	-1.043212
C	1.674410	-0.559509	-0.885120
C	1.173941	1.956796	0.104844
C	1.749965	3.739433	1.651605
F	0.045797	-2.249929	-0.726820
C	1.316332	2.449102	1.389700
B	0.627555	0.509778	-0.314721
F	1.871061	4.174497	2.902295
F	1.015726	1.680594	2.448031
C	-0.865715	0.570190	-0.906338
F	-0.422916	-0.677330	-2.873694
F	-1.531245	1.832481	0.971776
C	-1.275829	0.003119	-2.105326
C	-1.836817	1.290216	-0.213789
C	-2.574748	0.121959	-2.577089
C	-3.128862	1.456088	-0.670609
F	-2.937969	-0.464078	-3.715938
F	-4.022359	2.139091	0.038546

C	-3.502919	0.854989	-1.859276
F	2.469103	5.818511	0.828293
F	-4.748710	0.964603	-2.300018
F	4.291833	-3.489824	-2.213029
H	-0.055844	-0.094793	0.970363
Si	-0.345446	-1.657427	3.663204
H	0.709527	-0.177793	1.080725
H	-0.679681	-2.715256	4.648214
C	1.207648	-2.199500	2.742177
C	1.312506	-3.492430	2.206477
C	2.304749	-1.338322	2.595771
C	2.466039	-3.908667	1.550301
H	0.491711	-4.194641	2.318494
C	3.459574	-1.746991	1.930023
H	2.265589	-0.338208	3.016967
C	3.542620	-3.035053	1.412582
H	2.527368	-4.913062	1.147930
H	4.295607	-1.064139	1.827902
H	4.444254	-3.360908	0.905345
H	-0.052671	-0.393810	4.381392
C	-4.076796	-1.057387	0.820235
C	-3.891405	-0.263589	1.973811
C	-3.087814	-2.020872	0.530851
C	-2.767398	-0.417397	2.768684
H	-4.620443	0.488697	2.241079
C	-1.984244	-2.172418	1.357158
H	-3.183712	-2.667097	-0.330896
C	-1.779578	-1.374972	2.491698
H	-2.661940	0.232252	3.632591
H	-1.264158	-2.942148	1.101068
N	-5.157051	-0.867896	-0.011339
C	-5.373570	-1.796433	-1.106813
H	-4.523433	-1.789475	-1.795843
H	-5.524236	-2.826027	-0.759639
H	-6.251859	-1.482771	-1.665924
C	-6.293758	-0.091784	0.459605
H	-7.045482	-0.063130	-0.325915
H	-6.742851	-0.524932	1.361660
H	-6.001759	0.936852	0.673736

N1-6

Symbol	X	Y	Z
Si	1.673286	1.112648	0.110415
H	1.793222	2.535170	0.520837
H	2.396240	0.293139	1.117087
C	2.511681	0.812832	-1.546773
C	1.852106	1.107294	-2.747324
C	3.814631	0.305844	-1.616464
C	2.475568	0.909803	-3.975226
H	0.835337	1.488472	-2.722759
C	4.443192	0.105468	-2.842300
H	4.345894	0.057283	-0.702692
C	3.773612	0.409023	-4.023455
H	1.949880	1.144341	-4.893636
H	5.451399	-0.290893	-2.876149
H	4.260090	0.251879	-4.979234
C	-2.910569	0.015395	-0.152734
C	-2.468583	1.145704	0.567789
C	-1.926334	-0.775064	-0.787377
C	-1.120851	1.464882	0.629357
H	-3.180041	1.780499	1.077707
C	-0.586328	-0.433709	-0.709728
H	-2.209126	-1.656374	-1.346571
C	-0.138958	0.691085	-0.002350
H	-0.831494	2.349265	1.189917
H	0.132615	-1.066350	-1.223782
N	-4.243901	-0.297634	-0.241729
C	-4.645194	-1.553231	-0.847566
H	-4.338530	-1.600513	-1.896207
H	-4.217515	-2.418258	-0.325678
H	-5.729409	-1.631620	-0.814037
C	-5.206876	0.448816	0.545253
H	-6.203591	0.062111	0.345661
H	-5.012099	0.369791	1.622150
H	-5.198256	1.508100	0.273287

N1-7

Symbol	X	Y	Z
Si	-2.286593	1.432432	-0.293212
H	0.668317	0.082165	0.509547
H	-2.288316	2.378142	-1.424508
F	5.185035	-3.577935	1.616661
F	-0.032732	-4.695516	-2.471961
F	4.015674	-2.084886	-0.284698
C	4.031944	-2.953527	1.869522
C	-0.768039	-2.599012	-3.229917
C	0.008705	-3.362530	-2.380760
C	3.415010	-2.168546	0.912565

C	3.432718	-3.097032	3.110909
F	-1.500467	-0.466677	-3.907139
C	-0.735110	-1.220811	-3.113552
F	1.496801	-3.573121	-0.628172
C	0.794394	-2.742209	-1.418624
C	0.054213	-0.651205	-2.133478
C	0.836825	-1.367720	-1.238389
C	2.214772	-1.504517	1.119885
C	2.233149	-2.458945	3.364798
F	-0.016436	0.692882	-2.023486
C	1.652774	-1.680266	2.373000
B	1.548844	-0.552609	-0.018274
F	1.648640	-2.598375	4.559065
F	0.482996	-1.095370	2.693654
C	2.651921	0.547339	-0.511881
F	2.980286	-0.263275	-2.717970
F	2.535431	1.530145	1.636152
C	3.245803	0.640070	-1.761868
C	3.044863	1.528494	0.388418
C	4.135013	1.647861	-2.110501
C	3.919111	2.558016	0.085128
F	4.677936	1.687065	-3.329928
F	4.222887	3.491700	0.989641
C	4.473620	2.615790	-1.182102
F	4.006464	-3.850349	4.049636
F	5.327075	3.588478	-1.501599
F	-1.564379	-3.181585	-4.127117
H	-1.084037	1.420377	0.541613
C	-2.778892	-0.304524	-0.743996
C	-3.661108	-0.578822	-1.796446
C	-2.259941	-1.377805	-0.005202
C	-4.015465	-1.888238	-2.106236
H	-4.064749	0.234143	-2.392305
C	-2.619595	-2.687662	-0.307368
H	-1.547853	-1.189703	0.794684
C	-3.500290	-2.942048	-1.356078
H	-4.682694	-2.088095	-2.936474
H	-2.196143	-3.509543	0.259288
H	-3.765153	-3.963552	-1.604682
C	-5.198610	-0.234233	1.393686
C	-4.163971	0.153047	2.320168
C	-5.689478	0.764606	0.476010
C	-3.478952	1.304617	2.116840
H	-3.917563	-0.483926	3.156995
C	-4.979083	1.900298	0.277238
H	-6.598844	0.588620	-0.079484
C	-3.719383	2.134876	0.953443
H	-2.667344	1.585385	2.780028
H	-5.322329	2.627825	-0.450278
H	-3.394305	3.172074	1.033203
N	-5.687045	-1.464317	1.387781
C	-6.743773	-1.844392	0.446814
H	-6.893402	-2.917947	0.506269
H	-7.681866	-1.342622	0.692836
H	-6.444276	-1.588068	-0.570385
C	-5.169476	-2.477116	2.310700
H	-5.567965	-3.445332	2.023682
H	-4.081154	-2.515289	2.245855
H	-5.469077	-2.259184	3.338008
C	-0.759126	4.187513	0.857336
C	0.368790	3.655183	0.206298
C	-1.567898	5.083730	0.134666
C	0.634805	3.953291	-1.122431
H	1.034888	2.993763	0.739199
C	-1.282129	5.382195	-1.193531
H	-2.421635	5.556887	0.601945
C	-0.190800	4.813231	-1.840493
H	1.502400	3.502763	-1.594226
H	-1.926436	6.073556	-1.724781
H	0.019731	5.045071	-2.876910
N	-1.093802	3.787349	2.153662
C	-1.903523	4.702259	2.947776
H	-2.042286	4.276615	3.940408
H	-1.439207	5.691856	3.049035
H	-2.895831	4.833671	2.510892
C	-0.092632	3.032874	2.897888
H	-0.494155	2.799286	3.883030
H	0.133184	2.089633	2.394619
H	0.848268	3.585802	3.023098

N1-8

Symbol	X	Y	Z
Si	1.423758	1.164754	0.810504
H	-1.054676	0.271120	0.749075
H	1.243136	0.137800	-0.247884
F	-6.279814	-2.421377	1.454332
F	-5.122134	3.728899	-0.912321
F	-4.561413	-1.452060	-0.372190

C	-5.161263	-1.804881	1.845680
C	-3.120655	3.539340	-2.128348
C	-4.000749	3.052566	-1.184812
C	-4.255110	-1.306213	0.927376
C	-4.899051	-1.654530	3.198140
F	-1.091928	3.301807	-3.296150
C	-1.959246	2.833665	-2.393546
F	-4.615254	1.496421	0.406080
C	-3.705092	1.874017	-0.510502
C	-1.706965	1.668320	-1.697786
C	-2.550386	1.139174	-0.725741
C	-3.084442	-0.651158	1.279696
C	-3.746290	-1.006299	3.599624
F	-0.549479	1.044831	-1.981212
C	-2.867241	-0.520248	2.640717
B	-2.026816	-0.123427	0.159518
F	-3.492643	-0.856467	4.903541
F	-1.775125	0.108707	3.108857
C	-1.543170	-1.419284	-0.712924
F	-2.668771	-0.882794	-2.729578
F	-0.333216	-2.189140	1.174266
C	-1.884911	-1.711145	-2.024984
C	-0.738392	-2.369946	-0.097202
C	-1.456177	-2.849657	-2.693629
C	-0.316221	-3.534685	-0.714073
F	-1.805884	-3.071573	-3.962904
F	0.436536	-4.433112	-0.061832
C	-0.669996	-3.772194	-2.030089
F	-5.755392	-2.127660	4.104635
F	-0.248781	-4.878027	-2.649767
F	-3.366660	4.690432	-2.757870
H	0.982000	0.638848	2.124194
C	0.508769	2.757770	0.427840
C	0.960975	3.630457	-0.569163
C	-0.662542	3.090485	1.119853
C	0.262349	4.794287	-0.874975
H	1.866204	3.401238	-1.123685
C	-1.364507	4.253362	0.817381
H	-1.043964	2.418761	1.883333
C	-0.903180	5.106154	-0.181146
H	0.619937	5.451149	-1.659161
H	-2.276361	4.489167	1.354480
H	-1.456904	6.005704	-0.426065
C	6.141049	1.501688	1.186921
C	5.308378	1.412980	2.328277
C	5.501150	1.596033	-0.071575
C	3.928196	1.383065	2.199227
H	5.741304	1.370368	3.318300
C	4.115955	1.551497	-0.170825
H	6.085747	1.703216	-0.975516
C	3.282030	1.422189	0.951954
H	3.333728	1.306710	3.105538
H	3.679660	1.589873	-1.165744
H	4.270907	-0.568546	0.662973
N	7.502061	1.476000	1.294747
C	8.321365	1.660137	0.110089
H	9.369425	1.599402	0.391922
H	8.125370	0.877020	-0.628733
H	8.145088	2.632913	-0.362564
C	8.121933	1.504979	2.608210
H	9.202303	1.503876	2.488670
H	7.836768	2.398919	3.173645
H	7.846452	0.623723	3.195434
C	3.948481	-2.145534	-0.572024
C	3.091962	-3.227613	-0.682724
C	4.516380	-1.544998	-1.688954
C	2.788791	-3.711761	-1.953134
H	2.643104	-3.693113	0.182942
C	4.202148	-2.035553	-2.948915
H	5.186188	-0.700440	-1.577794
C	3.337336	-3.118088	-3.082101
H	2.115930	-4.554715	-2.049079
H	4.630974	-1.567164	-3.825522
H	3.088990	-3.495458	-4.066046
N	4.311109	-1.595299	0.756093
C	5.715368	-1.955932	1.137557
H	5.923706	-1.515023	2.110787
H	5.784459	-3.041535	1.174991
H	6.394950	-1.553663	0.388944
C	3.355623	-1.942321	1.850950
H	3.617055	-1.336310	2.715223
H	2.340447	-1.720869	1.523261
H	3.461054	-2.998031	2.090637

N1-9

Symbol	X	Y	Z
H	-0.053446	0.010873	1.344072
F	-3.660869	3.001262	-2.241337

F	2.494632	0.477839	-3.829231
F	-2.514628	0.621311	-1.838872
C	-2.737156	2.861565	-1.290688
C	3.016262	-1.185192	-2.255300
C	2.195015	-0.166301	-2.699321
C	-2.137003	1.637386	-1.053078
C	-2.365451	3.960692	-0.532949
F	3.502954	-2.812020	-0.628151
C	2.703784	-1.842844	-1.078325
F	0.330743	1.182890	-2.460905
C	1.076216	0.186996	-1.957014
C	1.581468	-1.453358	-0.371414
C	0.731620	-0.426534	-0.762598
C	-1.166677	1.433577	-0.079760
C	-1.396963	3.814957	0.442007
F	1.345383	-2.110293	0.781860
C	-0.829099	2.566010	0.639245
B	-0.504205	-0.026096	0.222221
F	-1.021522	4.864852	1.173968
F	0.124491	2.509181	1.606908
C	-1.645441	-1.188118	0.284702
F	-1.381419	-1.903439	-1.958060
F	-2.104115	-0.645885	2.544449
C	-1.983961	-2.027455	-0.767556
C	-2.348242	-1.405585	1.461611
C	-2.946645	-3.020200	-0.667839
C	-3.316948	-2.385711	1.605880
F	-3.237185	-3.801330	-1.710093
F	-3.959558	-2.553782	2.764125
C	-3.617130	-3.200809	0.529104
F	-2.930240	5.146343	-0.747873
F	-4.545532	-4.149210	0.643090
F	4.116565	-1.510640	-2.932115
H	1.090995	0.863294	2.242513
N	1.902097	0.629217	2.834828
C	2.168214	1.801843	3.726759
H	2.354822	2.674598	3.106655
H	1.291421	1.960683	4.350867
H	3.041379	1.571784	4.334029
C	3.019304	0.402498	1.891429
C	3.110777	1.276304	0.814255
C	3.930338	-0.622331	2.082336
C	4.148659	1.118114	-0.093286
H	2.369861	2.057060	0.678458
C	4.966869	-0.769567	1.162634
H	3.844778	-1.310508	2.912203
C	5.078763	0.095888	0.082140
H	4.221698	1.785642	-0.942904
H	5.678779	-1.574330	1.292403
H	5.880307	-0.034637	-0.634481
C	1.483130	-0.566429	3.624940
H	1.355333	-1.402518	2.942655
H	2.238302	-0.778248	4.378177
H	0.536606	-0.323819	4.103091

N1-10

Symbol	X	Y	Z
H	-0.726448	0.113004	2.275244
F	-4.203268	3.671454	-1.091641
F	3.285608	0.540341	-2.878215
F	-3.519368	1.101522	-1.215332
C	-2.998895	3.289963	-0.681401
C	3.046929	-1.365691	-1.528371
C	2.563018	-0.165453	-2.015072
C	-2.619015	1.961312	-0.737238
C	-2.109946	4.229876	-0.185811
F	2.802250	-3.202377	-0.081786
C	2.308200	-2.079217	-0.596310
F	0.901289	1.438389	-2.119312
C	1.323387	0.286403	-1.594464
C	1.071570	-1.608101	-0.208773
C	0.518433	-0.425348	-0.706028
C	-1.361591	1.516667	-0.330206
C	-0.852050	3.834286	0.238978
F	0.403818	-2.339942	0.686998
C	-0.500490	2.501223	0.148734
B	-0.947053	0.005717	-0.370344
F	-0.001170	4.732594	0.722381
F	0.720029	2.172947	0.575181
C	-2.041853	-1.093138	-0.122624
F	-1.130855	-2.564380	-1.739657
F	-3.135544	0.207082	1.525483
C	-2.059541	-2.299936	-0.819316
C	-3.067698	-0.915264	0.804329
C	-3.025604	-3.269083	-0.617180
C	-4.037668	-1.869560	1.046717
F	-3.009790	-4.401886	-1.310577
F	-4.985609	-1.663179	1.953835

C	-4.017018	-3.052013	0.325088
F	-2.462220	5.502450	-0.114577
F	-4.942299	-3.973293	0.537567
F	4.207536	-1.837123	-1.949598
H	0.000177	0.011191	2.138037
N	2.469964	-0.095974	2.459308
C	2.168534	1.022765	3.350336
H	1.878902	1.905517	2.783402
H	1.324069	0.748582	3.982421
H	3.022761	1.273483	3.993476
C	3.546769	0.100256	1.577577
C	3.667616	1.307181	0.866651
C	4.514098	-0.892629	1.350599
C	4.685034	1.490316	-0.057870
H	2.945640	2.097282	1.014016
C	5.532739	-0.694424	0.423082
H	4.481407	-1.827699	1.892582
C	5.623489	0.489197	-0.297516
H	4.735921	2.424170	-0.606091
H	6.256566	-1.485134	0.262332
H	6.408397	0.631839	-1.029527
C	2.398458	-1.386836	3.122998
H	2.415160	-2.189403	2.383600
H	3.217071	-1.549172	3.839890
H	1.450820	-1.448325	3.658995

N1-11

Symbol	X	Y	Z
Si	1.695196	-2.248061	-0.840896
H	-0.459647	0.081965	0.649212
H	1.347508	-3.315766	-1.802398
F	0.504795	5.877453	1.357143
F	1.685309	2.352395	-4.149515
F	-0.977405	4.079188	0.030027
C	0.738162	4.572491	1.513033
C	0.456912	0.353519	-4.066483
C	0.873655	1.530654	-3.478992
C	-0.019412	3.623429	0.848947
C	1.766738	4.157735	2.344586
F	-0.781495	-1.643143	-3.907535
C	-0.375299	-0.494807	-3.354918
F	0.971257	2.978404	-1.684686
C	0.472672	1.833963	-2.183950
C	-0.748281	-0.147628	-2.069181
C	-0.328730	1.006152	-1.415890
C	0.187252	2.255920	0.964121
C	2.008927	2.805654	2.497846
F	-1.546996	-1.016453	-1.423395
C	1.212140	1.892472	1.822817
B	-0.710582	1.154404	0.166243
F	3.009619	2.391599	3.285171
F	1.515121	0.593225	2.032975
C	-2.305080	1.394906	0.418512
F	-2.886463	2.037057	-1.794563
F	-1.992896	0.816663	2.691913
C	-3.247944	1.761666	-0.531060
C	-2.822327	1.155305	1.685431
C	-4.603145	1.868097	-0.257218
C	-4.169342	1.224818	1.998604
F	-5.467705	2.217621	-1.214216
F	-4.609121	0.944756	3.228740
C	-5.069709	1.585035	1.011564
F	2.517111	5.053844	2.984871
F	-6.376684	1.636127	1.274124
F	0.872692	0.023118	-5.289410
H	0.571795	-1.797766	-0.006287
C	2.650824	-0.884960	-1.677339
C	3.213451	-1.085949	-2.945330
C	2.878174	0.339765	-1.035936
C	3.982009	-0.097447	-3.550994
H	3.038964	-2.018723	-3.473560
C	3.663259	1.324704	-1.629640
H	2.419535	0.532433	-0.072085
C	4.216910	1.104655	-2.886910
H	4.390999	-0.260343	-4.540906
H	3.812978	2.272739	-1.125374
H	4.810604	1.877477	-3.360691
C	5.150092	-1.293032	0.514095
C	4.108629	-1.214158	1.505082
C	5.216505	-2.476095	-0.308409
C	3.084119	-2.099620	1.481757
H	4.112603	-0.423156	2.240939
C	4.186914	-3.354453	-0.313712
H	6.078304	-2.650440	-0.935689
C	2.977003	-3.116337	0.450552
H	2.278674	-2.012929	2.204879
H	4.231491	-4.227308	-0.956068
H	2.395403	-3.998512	0.721367

N	6.019996	-0.307032	0.363625
C	7.023644	-0.354792	-0.702810
H	7.534526	0.602007	-0.743576
H	7.759157	-1.138149	-0.510785
H	6.535016	-0.533485	-1.662610
C	5.928302	0.896258	1.193818
H	6.663869	1.615859	0.849320
H	4.933178	1.338240	1.106958
H	6.128498	0.661298	2.240517
Si	-2.820743	-3.753401	-1.035062
H	-2.067320	-3.596666	-2.302827
H	-3.361763	-5.138860	-1.015040
C	-4.228081	-2.510632	-1.017525
C	-5.078071	-2.409816	0.091943
C	-4.460564	-1.662266	-2.106608
C	-6.131781	-1.501470	0.109890
H	-4.912299	-3.043590	0.958797
C	-5.514276	-0.752995	-2.095518
H	-3.800982	-1.701141	-2.967967
C	-6.354799	-0.676155	-0.988805
H	-6.773268	-1.432167	0.981163
H	-5.670697	-0.093223	-2.940934
H	-7.164992	0.043687	-0.971624
C	-0.024585	-3.193681	2.731681
C	-0.956474	-2.204743	2.360715
C	0.021769	-4.370010	1.952447
C	-1.777377	-2.380007	1.257985
H	-1.036653	-1.281866	2.915047
C	-0.815815	-4.523323	0.855021
H	0.694040	-5.176855	2.214001
C	-1.732737	-3.536882	0.470944
H	-2.457063	-1.574151	0.994608
H	-0.751423	-5.451276	0.292381
N	0.829316	-3.004552	3.799782
C	1.619089	-4.122571	4.279575
H	0.997193	-4.977081	4.573978
H	2.322929	-4.462325	3.512664
H	2.198799	-3.802785	5.142487
C	0.614210	-1.867823	4.680028
H	1.365807	-1.883631	5.466141
H	0.719493	-0.928695	4.130577
H	-0.379334	-1.885252	5.144310

N1-12

Symbol	X	Y	Z
Si	-3.758641	1.071611	2.213955
H	0.000124	0.767530	-1.417211
H	-5.116511	1.299208	2.772152
F	4.377377	4.051501	-3.163619
F	1.639578	2.511376	3.915084
F	3.697645	1.622382	-2.381774
C	3.217854	3.874887	-2.523884
C	0.298911	0.625321	3.521674
C	1.093026	1.654224	3.050674
C	2.832741	2.609644	-2.095281
C	2.399626	4.960748	-2.283801
F	-0.981581	-1.298116	3.053740
C	-0.246275	-0.268262	2.617026
F	2.105204	2.809306	1.325999
C	1.323224	1.781167	1.686571
C	0.008997	-0.099508	1.267243
C	0.796744	0.916117	0.737720
C	1.646714	2.359665	-1.422576
C	1.193942	4.761389	-1.631996
F	-0.538014	-1.015363	0.445386
C	0.849773	3.483149	-1.237502
B	1.066506	0.931295	-0.881543
F	0.389165	5.799843	-1.386561
F	-0.337413	3.350464	-0.619989
C	2.001701	-0.362208	-1.207281
F	3.761617	0.472159	0.115728
F	0.366080	-1.414716	-2.575118
C	3.268966	-0.503891	-0.662988
C	1.572351	-1.432183	-1.974789
C	4.084265	-1.600974	-0.878268
C	2.334915	-2.573893	-2.176747
F	5.317203	-1.654621	-0.370389
F	1.857887	-3.600058	-2.897582
C	3.598360	-2.661753	-1.622532
F	2.760363	6.182255	-2.678424
F	4.337844	-3.754855	-1.806716
F	0.058268	0.497978	4.825674
H	-3.274829	-0.222347	2.764738
C	-2.566800	2.420235	2.735165
C	-2.228544	2.578099	4.087917
C	-1.973741	3.270521	1.798962
C	-1.330541	3.559206	4.487981
H	-2.660724	1.921093	4.837413

C	-1.062930	4.249406	2.194590
H	-2.216036	3.170745	0.747776
C	-0.747018	4.397834	3.538899
H	-1.071326	3.662052	5.535229
H	-0.600557	4.884052	1.447624
H	-0.032060	5.150644	3.848877
C	-4.338346	0.422750	-2.443905
C	-3.043837	0.533019	-1.882211
C	-5.435319	0.621277	-1.573547
C	-2.870880	0.778970	-0.525989
H	-2.164862	0.438132	-2.505387
C	-5.234709	0.865879	-0.221793
H	-6.447561	0.590830	-1.953445
C	-3.956669	0.921362	0.356574
H	-1.849517	0.868041	-0.172175
H	-6.110860	1.008757	0.403910
H	-4.009336	-1.295493	-0.572849
N	-4.520496	0.109187	-3.762815
C	-5.847310	0.230893	-4.344882
H	-5.788350	0.008940	-5.407200
H	-6.543329	-0.480531	-3.890934
H	-6.255724	1.240220	-4.221422
C	-3.367503	0.045435	-4.646891
H	-3.701269	-0.248477	-5.638738
H	-2.847384	1.007248	-4.718419
H	-2.654110	-0.706512	-4.299502
Si	0.991286	-5.341195	0.504784
H	0.733051	-6.417888	1.490310
H	1.462214	-5.938571	-0.766149
C	2.264018	-4.130317	1.166940
C	3.623427	-4.472158	1.219290
C	1.871856	-2.884010	1.668475
C	4.555571	-3.602499	1.770835
H	3.961032	-5.424740	0.822278
C	2.807727	-2.004295	2.211483
H	0.826230	-2.597892	1.626082
C	4.147701	-2.366693	2.269191
H	5.603465	-3.877140	1.796690
H	2.493083	-1.032218	2.575992
H	4.878691	-1.680566	2.680741
C	-3.008858	-3.012793	-0.140701
C	-2.020232	-2.955725	-1.114343
C	-2.836403	-3.753367	1.017991
C	-0.850261	-3.677063	-0.928609
H	-2.140837	-2.342992	-2.000038
C	-1.646397	-4.456471	1.191790
H	-3.591391	-3.797300	1.789870
C	-0.639166	-4.439156	0.225590
H	-0.097242	-3.641430	-1.705751
H	-1.517613	-5.027869	2.104375
N	-4.275314	-2.279211	-0.408489
C	-5.239364	-2.279836	0.730055
H	-5.565353	-3.303169	0.905800
H	-4.749630	-1.869164	1.610256
H	-6.085243	-1.661163	0.444171
C	-4.947351	-2.792578	-1.647775
H	-5.879988	-2.247954	-1.775185
H	-4.299786	-2.625080	-2.504347
H	-5.130076	-3.856314	-1.510043

N1-13

Symbol	X	Y	Z
H	-1.161232	-0.174933	1.413310
F	-3.758856	4.785070	0.558366
F	2.341588	0.912374	-3.022139
F	-3.824140	2.204931	0.008996
C	-2.648282	4.061074	0.710857
C	1.866003	-1.258664	-2.264966
C	1.601671	0.098475	-2.266272
C	-2.647717	2.701229	0.423523
C	-1.494063	4.667331	1.164373
F	1.298209	-3.417459	-1.511424
C	1.065041	-2.104199	-1.516148
F	0.377955	1.926782	-1.561988
C	0.561936	0.601808	-1.494987
C	0.033996	-1.553381	-0.779024
C	-0.248739	-0.195909	-0.700768
C	-1.528443	1.893090	0.557160
C	-0.350609	3.902364	1.326341
F	-0.715214	-2.423561	-0.059338
C	-0.399231	2.554820	1.026816
B	-1.442616	0.280691	0.319591
F	0.772486	4.471658	1.767782
F	0.744407	1.864042	1.203371
C	-2.822507	-0.420283	-0.179945
F	-2.814138	0.828871	-2.173076
F	-2.991529	-1.824821	1.728962
C	-3.382070	-0.117752	-1.412785

C	-3.469544	-1.414567	0.528713
C	-4.517094	-0.733551	-1.907820
C	-4.605677	-2.067858	0.076116
F	-5.023972	-0.398727	-3.094661
F	-5.183999	-3.025743	0.803722
C	-5.134450	-1.719298	-1.152795
F	-1.479230	5.968564	1.447262
F	-6.225685	-2.328857	-1.611316
F	2.877880	-1.749301	-2.981754
H	-0.887017	-1.812789	2.101541
C	1.070206	-2.318199	2.120395
C	1.784595	-3.435841	1.720034
C	1.575426	-1.036165	1.949571
C	3.030183	-3.251766	1.126543
H	1.392660	-4.436702	1.832627
C	2.814381	-0.872819	1.343963
H	0.996908	-0.165846	2.232737
C	3.564240	-1.976883	0.921408
H	3.575740	-4.131516	0.801690
H	3.183726	0.135060	1.183877
N	-0.299586	-2.433828	2.681680
C	-0.375488	-1.924256	4.088138
H	-1.417205	-1.951117	4.400737
H	0.235184	-2.570627	4.715007
H	-0.000418	-0.905401	4.117253
C	-0.908321	-3.793059	2.587700
H	-1.943034	-3.708787	2.908202
H	-0.865349	-4.125762	1.554807
H	-0.363313	-4.469182	3.242714
Si	5.242581	-1.757473	0.080554
H	6.307324	-2.224006	0.999270
H	5.253006	-2.572420	-1.155729
C	5.473698	0.055257	-0.306491
C	6.062821	0.923514	0.621719
C	4.961123	0.595157	-1.492304
C	6.133297	2.290697	0.373797
H	6.470255	0.531098	1.548495
C	5.023165	1.962771	-1.740631
H	4.512536	-0.056231	-2.235499
C	5.610045	2.810810	-0.806633
H	6.593738	2.950005	1.100129
H	4.611192	2.362837	-2.659118
H	5.659921	3.876200	-0.998002

N1-14

Symbol	X	Y	Z
H	-1.216005	0.628681	2.413569
F	-2.642771	5.119501	-1.398322
F	2.773324	2.162596	-0.596590
F	-1.356848	2.806011	-1.716361
C	-2.893624	4.107164	-0.575949
C	2.260999	0.152372	-1.691874
C	1.885943	1.217909	-0.888148
C	-2.235565	2.899019	-0.718051
C	-3.825183	4.258521	0.438525
F	1.736889	-1.903599	-2.693627
C	1.367394	-0.869333	-1.949202
F	0.299287	2.307954	0.384588
C	0.602714	1.264811	-0.387467
C	0.095686	-0.807989	-1.405716
C	-0.352473	0.271480	-0.640011
C	-2.480122	1.806319	0.113148
C	-4.095527	3.202498	1.293316
F	-0.699827	-1.842737	-1.675824
C	-3.434686	2.002124	1.108283
B	-1.821942	0.402160	-0.139537
F	-4.979770	3.351215	2.272500
F	-3.712153	1.016785	1.962992
C	-2.788087	-0.855450	-0.141282
F	-3.066386	-0.414919	-2.426912
F	-2.619457	-1.446523	2.141132
C	-3.373387	-1.167122	-1.360654
C	-3.120323	-1.669705	0.924939
C	-4.228151	-2.237099	-1.537163
C	-3.964820	-2.760715	0.785216
F	-4.757592	-2.504317	-2.726136
F	-4.227119	-3.541257	1.830590
C	-4.520953	-3.045327	-0.448552
F	-4.453608	5.412382	0.591988
F	-5.330329	-4.085789	-0.591636
F	3.482539	0.109532	-2.200058
H	-0.617022	0.254810	2.199146
C	1.478234	-2.168526	1.273908
C	2.028473	-3.019852	0.300353
C	2.248030	-1.059122	1.672071
C	3.298815	-2.782771	-0.214698
H	1.475064	-3.876229	-0.059639
C	3.514923	-0.842125	1.154699

H	1.848922	-0.352269	2.387294
C	4.081538	-1.702351	0.203185
H	3.679926	-3.471997	-0.962843
H	4.069828	0.028526	1.493069
N	0.202301	-2.379050	1.819199
C	0.099207	-2.243889	3.273998
H	-0.953384	-2.229292	3.552711
H	0.594099	-3.081638	3.784528
H	0.548554	-1.316975	3.619103
C	-0.510708	-3.556926	1.347334
H	-1.481667	-3.591902	1.840271
H	-0.671130	-3.498018	0.269104
H	0.020347	-4.491692	1.579405
Si	5.814340	-1.438412	-0.465486
H	6.729933	-2.485341	0.053240
H	5.793647	-1.526878	-1.945270
C	6.474299	0.224494	0.100930
C	7.444111	0.293845	1.109064
C	5.986849	1.426904	-0.430522
C	7.910454	1.520422	1.575404
H	7.841995	-0.620364	1.538255
C	6.448592	2.653890	0.033591
H	5.236444	1.412534	-1.214133
C	7.411647	2.702086	1.038142
H	8.661512	1.552737	2.356077
H	6.058029	3.572499	-0.388670
H	7.772333	3.658282	1.399055

N1-TS1

Symbol	X	Y	Z
Si	-1.043021	0.122253	-1.883088
H	0.430651	0.036110	-0.621427
H	-0.436819	-1.082703	-2.470552
F	5.392104	2.974711	-0.214413
F	-0.614281	1.302864	4.669739
F	4.319604	0.574559	0.131423
C	4.065430	2.855820	-0.146604
C	-1.211143	-0.777809	3.757448
C	-0.476850	0.389776	3.704927
C	3.474408	1.610453	0.022162
C	3.271545	3.982358	-0.245233
F	-1.760169	-2.852678	2.796850
C	-1.057958	-1.718726	2.753562
F	1.062399	1.772148	2.678529
C	0.389503	0.610920	2.641785
C	-0.197957	-1.450207	1.707340
C	0.552006	-0.283839	1.594425
C	2.101377	1.424509	0.091776
C	1.895355	3.850061	-0.164033
F	-0.122097	-2.380712	0.740976
C	1.353186	2.590337	0.004870
B	1.357041	-0.014129	0.213765
F	1.117113	4.930918	-0.243499
F	0.013797	2.523032	0.117031
C	2.280775	-1.228967	-0.331999
F	2.599472	-2.309242	1.752333
F	2.145360	-0.304236	-2.502694
C	2.816387	-2.254846	0.429836
C	2.595321	-1.273135	-1.682746
C	3.600126	-3.265877	-0.104320
C	3.371047	-2.263052	-2.260296
F	4.089341	-4.234021	0.672623
F	3.636772	-2.255670	-3.568033
C	3.878911	-3.270788	-1.459296
F	3.822098	5.183282	-0.408783
F	4.629227	-4.235819	-1.988031
F	-2.071048	-0.994844	4.751820
H	-0.393600	1.417847	-2.117776
C	-2.185277	-0.169694	-0.446031
C	-2.744440	-1.442115	-0.270911
C	-2.548883	0.849670	0.441952
C	-3.647751	-1.689303	0.758019
H	-2.470416	-2.253473	-0.937529
C	-3.445687	0.602035	1.476793
H	-2.129878	1.842636	0.330777
C	-4.002471	-0.665215	1.630692
H	-4.060007	-2.683024	0.887989
H	-3.707231	1.397934	2.164869
H	-4.696372	-0.858346	2.440984
C	-4.955007	0.325022	-2.105065
C	-4.300433	1.581511	-2.314072
C	-4.466838	-0.811630	-2.826002
C	-3.126254	1.633687	-3.005629
H	-4.721048	2.489174	-1.906756
C	-3.285083	-0.737768	-3.503107
H	-5.011939	-1.743902	-2.803939
C	-2.494123	0.450719	-3.498157
H	-2.627688	2.588225	-3.134216

H	-2.908240	-1.617628	-4.013291
H	-1.726710	0.558921	-4.261178
N	-5.988727	0.217024	-1.265079
C	-6.677207	-1.059768	-1.099562
H	-7.439567	-0.949447	-0.333865
H	-7.159581	-1.370706	-2.029670
H	-5.972821	-1.831680	-0.781781
C	-6.459212	1.379210	-0.516878
H	-7.190869	1.050517	0.215747
H	-5.626160	1.846467	0.011716
H	-6.927587	2.114306	-1.176870

N1-TS2

Symbol	X	Y	Z
F	1.474924	5.162592	-1.725271
F	5.143526	-0.311862	-2.070541
F	0.900427	2.578550	-2.222422
C	1.340489	4.302388	-0.718257
C	3.739416	-2.162071	-1.732386
C	3.958704	-0.799294	-1.705668
C	1.039505	2.973079	-0.949820
C	1.507418	4.737369	0.587019
F	2.296978	-3.975844	-1.336178
C	2.508793	-2.661807	-1.340644
F	3.254137	1.356207	-1.285485
C	2.948241	0.053780	-1.281609
C	1.535886	-1.780540	-0.918582
C	1.698079	-0.399931	-0.879287
C	0.902207	2.038234	0.065125
C	1.371677	3.840049	1.630879
F	0.369485	-2.307307	-0.522005
C	1.071571	2.515747	1.352260
B	0.506246	0.527041	-0.324444
F	1.521564	4.255668	2.887164
F	0.921247	1.696984	2.408434
C	-0.955989	0.425647	-0.992430
F	-0.394410	-1.054603	-2.762556
F	-1.776675	1.917867	0.654448
C	-1.309226	-0.350939	-2.089483
C	-1.998871	1.151606	-0.421696
C	-2.613140	-0.449040	-2.555182
C	-3.301075	1.105589	-0.879299
F	-2.910657	-1.242717	-3.582649
F	-4.260941	1.801375	-0.271465
C	-3.616068	0.280199	-1.942707
F	1.796342	6.010910	0.833749
F	-4.874527	0.179875	-2.357524
F	4.710089	-2.992298	-2.097773
H	-0.331813	-0.389562	1.257825
Si	0.004439	-1.537095	3.438210
H	0.411040	-0.031626	0.929879
H	-0.443314	-2.621685	4.342847
C	1.590854	-2.081611	2.598276
C	1.788431	-3.425011	2.248792
C	2.628563	-1.172890	2.346965
C	2.981867	-3.848815	1.673361
H	1.008552	-4.155909	2.441002
C	3.824516	-1.592584	1.771351
H	2.506270	-0.127218	2.612017
C	4.003534	-2.932115	1.439663
H	3.116509	-4.890914	1.409671
H	4.617075	-0.876097	1.587361
H	4.935387	-3.261352	0.992958
H	0.233174	-0.289698	4.199758
C	-3.998279	-1.232290	1.028977
C	-3.638523	-0.249808	1.992335
C	-3.028094	-2.217116	0.700041
C	-2.375579	-0.229850	2.534327
H	-4.348318	0.512081	2.282594
C	-1.786111	-2.199475	1.287352
H	-3.267623	-3.011230	0.007535
C	-1.379109	-1.185936	2.195761
H	-2.132274	0.557092	3.242004
H	-1.091777	-2.993517	1.036791
N	-5.212777	-1.219775	0.429747
C	-5.548219	-2.239589	-0.554361
H	-4.800150	-2.265736	-1.350946
H	-5.615268	-3.235048	-0.103550
H	-6.505432	-1.991534	-1.004567
C	-6.271459	-0.347795	0.926675
H	-7.166797	-0.515729	0.334416
H	-6.500263	-0.561233	1.975218
H	-5.992566	0.701814	0.830421

N1-TS3

Symbol	X	Y	Z
Si	-1.633698	-0.303161	0.739713
H	0.900377	-0.220211	0.644953

H	-1.245959	0.680013	-0.294496
F	6.681340	0.778201	1.411215
F	3.744510	-4.723650	-1.039424
F	4.761309	0.417144	-0.436927
C	5.428882	0.509902	1.788700
C	1.894849	-3.929806	-2.251565
C	2.881650	-3.737877	-1.306968
C	4.420926	0.329309	0.859188
C	5.127162	0.402080	3.137077
F	0.032927	-3.084320	-3.413849
C	1.002663	-2.903547	-2.512601
F	3.933977	-2.450089	0.295294
C	2.955119	-2.529834	-0.624086
C	1.114522	-1.720902	-1.809099
C	2.076694	-1.478898	-0.834159
C	3.107561	0.039001	1.197251
C	3.832120	0.112516	3.524103
F	0.202445	-0.771929	-2.086735
C	2.853796	-0.061013	2.554172
B	1.952714	-0.126411	0.063394
F	3.537885	0.003074	4.823263
F	1.619597	-0.348206	3.005563
C	1.885829	1.264886	-0.790482
F	2.817421	0.441924	-2.809296
F	0.944310	2.335586	1.103296
C	2.311154	1.458266	-2.096608
C	1.395977	2.404158	-0.165023
C	2.246285	2.681197	-2.749709
C	1.346313	3.649811	-0.765302
F	2.653530	2.804435	-4.015044
F	0.888157	4.721826	-0.104699
C	1.760392	3.787032	-2.077841
F	6.081188	0.572203	4.053183
F	1.683014	4.973214	-2.683231
F	1.781839	-5.097007	-2.888338
H	-1.076595	0.020321	2.069166
C	-1.289321	-2.093141	0.317880
C	-1.981336	-2.755400	-0.703282
C	-0.304713	-2.797653	1.022506
C	-1.696452	-4.080904	-1.016101
H	-2.744245	-2.234464	-1.274186
C	-0.015938	-4.122287	0.710300
H	0.259508	-2.296122	1.803694
C	-0.712015	-4.765264	-0.309171
H	-2.230796	-4.574766	-1.819165
H	0.759748	-4.648859	1.254794
H	-0.478698	-5.793862	-0.560917
C	-6.092539	-1.371700	0.840114
C	-5.342550	-1.297629	2.056590
C	-5.530591	-0.771627	-0.328803
C	-4.126653	-0.679624	2.082377
H	-5.733984	-1.735642	2.963114
C	-4.308482	-0.160813	-0.275996
H	-6.064211	-0.806276	-1.267537
C	-3.534306	-0.067481	0.928101
H	-3.583709	-0.645460	3.023458
H	-3.907876	0.276046	-1.186947
H	-3.358901	1.187068	1.184209
N	-7.284335	-1.980898	0.801468
C	-8.040747	-2.050627	-0.445811
H	-8.970582	-2.580517	-0.263009
H	-8.278306	-1.049623	-0.813844
H	-7.479466	-2.588277	-1.213468
C	-7.842018	-2.595728	2.003284
H	-8.804804	-3.033628	1.758377
H	-7.186695	-3.385462	2.377640
H	-7.989685	-1.851760	2.789579
C	-3.211447	3.231943	0.169882
C	-2.028239	3.874884	-0.198148
C	-4.254156	3.138041	-0.758096
C	-1.893746	4.409779	-1.475680
H	-1.210810	3.981657	0.499553
C	-4.105265	3.671138	-2.032534
H	-5.183491	2.647974	-0.500856
C	-2.925366	4.308253	-2.399999
H	-0.973192	4.917024	-1.739818
H	-4.922191	3.587323	-2.739039
H	-2.813448	4.725586	-3.392789
N	-3.354755	2.607065	1.458104
C	-4.684908	2.767273	2.075272
H	-4.654010	2.327992	3.071554
H	-4.958728	3.822695	2.145649
H	-5.434315	2.228685	1.495911
C	-2.272921	2.868670	2.419997
H	-2.465221	2.275916	3.313540
H	-1.314889	2.561695	2.003050
H	-2.233149	3.928116	2.691043

N1-TS4

Symbol	X	Y	Z
H	-0.546304	0.029200	1.517805
F	-4.547716	3.005709	-1.165267
F	3.033144	1.429542	-3.077795
F	-3.431877	0.593771	-1.121647
C	-3.319057	2.862786	-0.678146
C	3.256904	-0.602688	-1.926491
C	2.526390	0.529085	-2.238892
C	-2.716140	1.614029	-0.640739
C	-2.633902	3.967109	-0.205478
F	3.436821	-2.617412	-0.725957
C	2.742831	-1.526540	-1.032669
F	0.635042	1.839160	-2.015392
C	1.285145	0.728080	-1.656320
C	1.507824	-1.285777	-0.464526
C	0.729967	-0.168316	-0.749882
C	-1.433312	1.417812	-0.146354
C	-1.349980	3.817148	0.295823
F	1.060490	-2.187912	0.419271
C	-0.783214	2.558736	0.306517
B	-0.710665	-0.003938	-0.090340
F	-0.684484	4.874404	0.749658
F	0.464818	2.448695	0.784282
C	-1.660614	-1.287107	-0.019743
F	-0.697795	-2.358103	-1.902468
F	-2.788690	-0.387359	1.843302
C	-1.600684	-2.344603	-0.918007
C	-2.656852	-1.374705	0.945923
C	-2.466993	-3.425080	-0.860360
C	-3.531919	-2.439268	1.044209
F	-2.376634	-4.413125	-1.745422
F	-4.460715	-2.478054	1.994321
C	-3.434888	-3.473112	0.126889
F	-3.200278	5.165070	-0.233044
F	-4.267062	-4.502765	0.195188
F	4.453175	-0.789973	-2.464262
H	0.240912	0.080598	1.536034
N	1.815004	0.103818	2.568364
C	1.569159	1.314316	3.364089
H	1.802827	2.209917	2.797231
H	0.511806	1.348124	3.635197
H	2.169475	1.304845	4.282453
C	3.070371	0.083515	1.896932
C	3.424804	1.162696	1.071620
C	3.946457	-1.002238	1.983990
C	4.619767	1.154390	0.370319
H	2.745513	1.997005	0.953142
C	5.139884	-1.006150	1.262620
H	3.710916	-1.856180	2.603619
C	5.488686	0.066713	0.456365
H	4.862893	1.994926	-0.270280
H	5.795591	-1.865454	1.337877
H	6.414072	0.056762	-0.105468
C	1.495897	-1.082372	3.359132
H	1.537841	-1.975050	2.737573
H	2.171520	-1.201894	4.216147
H	0.477895	-0.975405	3.737989

N1-TS5

Symbol	X	Y	Z
Si	0.620961	3.714372	1.116018
H	0.786864	-0.551505	-1.139744
H	0.611348	5.169753	1.396741
F	3.015325	-5.680816	-2.000566
F	1.300013	-1.529690	4.583892
F	0.765071	-4.345054	-1.623145
C	3.063064	-4.470705	-1.439049
C	-0.202518	0.029120	3.679943
C	0.685190	-1.016749	3.515844
C	1.897201	-3.745117	-1.222466
C	4.283199	-3.932710	-1.080589
F	-1.661627	1.595106	2.711407
C	-0.802994	0.580635	2.562136
F	1.825089	-2.521625	2.182601
C	0.952609	-1.502553	2.243744
C	-0.494308	0.074238	1.311440
C	0.383207	-0.981188	1.091777
C	1.876669	-2.484331	-0.646284
C	4.316369	-2.675042	-0.501318
F	-1.107579	0.655515	0.259262
C	3.131021	-1.993797	-0.301345
B	0.581811	-1.514133	-0.446084
F	5.488525	-2.127132	-0.162907
F	3.238409	-0.774280	0.264983
C	-0.856751	-2.137525	-0.886960
F	-0.715578	-3.852809	0.719969
F	-1.229090	-0.476849	-2.542839

C	-1.420215	-3.225163	-0.235854
C	-1.664654	-1.539401	-1.838035
C	-2.684914	-3.711112	-0.510745
C	-2.953379	-1.970384	-2.123226
F	-3.184530	-4.759076	0.149868
F	-3.708115	-1.328871	-3.023410
C	-3.463109	-3.070629	-1.461590
F	5.412030	-4.609184	-1.290605
F	-4.688926	-3.513704	-1.736830
F	-0.470452	0.506675	4.894096
H	-0.766512	3.222023	0.982951
C	1.563195	2.798059	2.450913
C	1.325895	3.105813	3.797406
C	2.459304	1.764468	2.150548
C	1.961363	2.398639	4.812975
H	0.632938	3.899256	4.061548
C	3.094293	1.055071	3.165576
H	2.657466	1.487807	1.120933
C	2.846020	1.371164	4.497918
H	1.758525	2.641935	5.849089
H	3.767760	0.244252	2.910733
H	3.331536	0.811402	5.288457
C	3.757176	2.348870	-1.864839
C	2.567547	1.579863	-1.676430
C	3.746738	3.716064	-1.435505
C	1.482267	2.125670	-1.057077
H	2.524696	0.544933	-1.984899
C	2.651546	4.237939	-0.812816
H	4.622193	4.334001	-1.572856
C	1.447303	3.484420	-0.585568
H	0.610301	1.495990	-0.905783
H	2.694460	5.268193	-0.470019
H	0.458547	4.047507	-1.214159
N	4.848530	1.803957	-2.412115
C	6.063477	2.593955	-2.595026
H	6.802630	1.985435	-3.106981
H	5.860922	3.475331	-3.205953
H	6.475084	2.911031	-1.633444
C	4.882688	0.374540	-2.719432
H	5.858507	0.126591	-3.125698
H	4.720831	-0.213404	-1.812881
H	4.122301	0.114623	-3.458299
Si	-5.642553	1.457978	-0.660082
H	-6.525315	2.303036	0.181960
H	-6.385962	1.085704	-1.888659
C	-5.155606	-0.120703	0.232894
C	-5.976255	-1.253368	0.169185
C	-4.005427	-0.178276	1.032608
C	-5.675966	-2.398870	0.901425
H	-6.861230	-1.246912	-0.459941
C	-3.695999	-1.325945	1.756593
H	-3.346933	0.681857	1.089221
C	-4.538287	-2.433301	1.700586
H	-6.320730	-3.267422	0.837278
H	-2.798004	-1.361676	2.364456
H	-4.294943	-3.328310	2.261111
C	-1.803653	3.945750	-1.742625
C	-1.881577	2.579020	-2.028044
C	-2.887616	4.574755	-1.130114
C	-3.023094	1.865892	-1.697961
H	-1.056123	2.051874	-2.489483
C	-4.024279	3.839798	-0.800848
H	-2.864968	5.631039	-0.900850
C	-4.119532	2.474437	-1.077197
H	-3.049681	0.807261	-1.926262
H	-4.848382	4.355610	-0.319035
N	-0.574948	4.646512	-2.021326
C	-0.540532	6.062256	-1.636274
H	-1.233653	6.658535	-2.236460
H	-0.787369	6.166518	-0.580370
H	0.473718	6.429585	-1.795238
C	-0.053871	4.458793	-3.388472
H	0.937085	4.912001	-3.439710
H	0.039948	3.399792	-3.615572
H	-0.713620	4.932839	-4.119703

N1-TS6

Symbol	X	Y	Z
H	-1.229151	0.098153	1.399480
F	-4.650757	3.660850	-1.535764
F	2.693277	2.538210	-1.612807
F	-3.357877	1.340969	-1.825028
C	-3.780481	3.272089	-0.608203
C	2.673938	0.186966	-1.672030
C	2.066722	1.390573	-1.363640
C	-3.106466	2.066858	-0.729645
C	-3.538303	4.078432	0.489537
F	2.593429	-2.161986	-1.664794

C	2.010153	-0.997019	-1.395729
F	0.275750	2.604786	-0.555615
C	0.790087	1.397732	-0.817445
C	0.733705	-0.946858	-0.873382
C	0.067938	0.237585	-0.565817
C	-2.183313	1.624631	0.207138
C	-2.621158	3.677266	1.447498
F	0.127301	-2.127168	-0.677721
C	-1.964754	2.471961	1.283713
B	-1.426012	0.210381	0.024482
F	-2.381022	4.451054	2.502217
F	-1.072200	2.126566	2.227085
C	-2.421424	-0.957908	-0.465130
F	-1.548113	-1.021945	-2.657248
F	-3.463741	-1.026690	1.651885
C	-2.420938	-1.474493	-1.753131
C	-3.389593	-1.469935	0.384362
C	-3.304633	-2.455374	-2.168423
C	-4.283390	-2.460867	0.014921
F	-3.267597	-2.921318	-3.413507
F	-5.161094	-2.945970	0.889671
C	-4.238987	-2.955807	-1.275770
F	-4.176756	5.234216	0.622604
F	-5.085648	-3.903165	-1.658424
F	3.864483	0.153076	-2.246431
H	-1.146941	-0.717111	1.444156
C	0.791091	-1.775866	2.272700
C	1.651134	-2.785929	1.842040
C	1.314753	-0.496110	2.504359
C	3.004624	-2.517543	1.652155
H	1.280265	-3.781612	1.641460
C	2.665685	-0.247622	2.320008
H	0.671126	0.314050	2.826770
C	3.545820	-1.252653	1.893548
H	3.647195	-3.326995	1.321245
H	3.036179	0.751622	2.528057
N	-0.619256	-1.982308	2.419134
C	-1.137027	-1.614617	3.749190
H	-2.224297	-1.570737	3.696787
H	-0.836985	-2.357477	4.496028
H	-0.764797	-0.638846	4.052869
C	-1.125408	-3.300046	2.027513
H	-2.211308	-3.284055	2.128184
H	-0.868994	-3.508908	0.990116
H	-0.732036	-4.093843	2.673438
Si	5.388678	-0.923155	1.738610
H	5.812059	-0.215566	2.971944
H	6.086495	-2.225054	1.621579
C	5.852529	0.135501	0.261913
C	5.440798	1.472846	0.177020
C	6.650519	-0.377800	-0.766610
C	5.811092	2.269370	-0.901141
H	4.828765	1.906602	0.963593
C	7.028995	0.417871	-1.843972
H	6.986609	-1.408992	-0.726142
C	6.608860	1.741633	-1.912056
H	5.478548	3.299124	-0.952635
H	7.649597	0.003963	-2.630224
H	6.900696	2.361539	-2.751662

PhNHMe

Symbol	X	Y	Z
C	-0.162804	-0.032836	-0.076771
C	0.573408	-1.226401	-0.024361
C	0.540401	1.184906	-0.053892
C	1.962952	-1.191000	0.036151
H	0.064436	-2.182081	-0.030533
C	1.924012	1.203141	0.010045
H	-0.014793	2.116940	-0.090798
C	2.653560	0.015282	0.053744
H	2.508098	-2.127456	0.074124
H	2.437588	2.157887	0.026689
H	3.734827	0.032469	0.102530
N	-1.536324	-0.031267	-0.177624
C	-2.316715	-1.227494	0.052931
H	-3.372866	-0.968245	0.004497
H	-2.119648	-1.971514	-0.723041
H	-2.110509	-1.684898	1.028742
H	-1.982543	0.840968	0.058317

add3

Symbol	X	Y	Z
B	-0.073540	-0.129101	0.446908
C	1.176551	-1.004353	-0.132284
C	2.336741	-0.408114	-0.620000
C	3.442113	-1.122011	-1.049113
C	3.421143	-2.504662	-0.998928
C	2.296980	-3.146729	-0.513957

C	1.212948	-2.392803	-0.091850
C	-0.375250	1.259528	-0.376867
C	-1.143989	2.271778	0.184210
C	-1.530276	3.425349	-0.472872
C	-1.177947	3.586335	-1.800475
C	-0.458478	2.587482	-2.429436
C	-0.083566	1.452793	-1.724756
C	-1.533658	-0.885752	0.437673
C	-1.929744	-1.544040	-0.723908
C	-3.178099	-2.110001	-0.902814
C	-4.118005	-2.017312	0.110399
C	-3.785825	-1.356991	1.276489
C	-2.520073	-0.804668	1.410659
F	2.447408	0.924493	-0.676296
F	4.527398	-0.491852	-1.493678
F	4.473233	-3.206744	-1.402399
F	2.268282	-4.476092	-0.446788
F	0.176486	-3.087966	0.400722
F	-1.570016	2.154158	1.461971
F	-2.240515	4.361639	0.150898
F	-1.537853	4.677849	-2.464840
F	-0.135735	2.718534	-3.713775
F	0.572686	0.530818	-2.436193
F	-2.290891	-0.175550	2.583654
F	-4.676274	-1.251881	2.259784
F	-5.323690	-2.552995	-0.040543
F	-3.487020	-2.734910	-2.036028
F	-1.075983	-1.645615	-1.750586
N	0.359229	0.186145	2.022967
H	-0.406103	0.732012	2.421659
C	1.571596	1.011315	2.156473
C	1.436468	2.394126	2.198888
C	2.829612	0.422321	2.221212
C	2.571375	3.193068	2.269674
H	0.455729	2.851572	2.194489
C	3.958580	1.231110	2.285786
H	2.950410	-0.652221	2.209780
C	3.834993	2.615221	2.301507
H	2.460863	4.269572	2.302621
H	4.937412	0.769747	2.323537
H	4.718145	3.239642	2.350231
C	0.444851	-1.064521	2.840261
H	-0.508720	-1.579519	2.805901
H	1.209813	-1.717007	2.432049
H	0.693655	-0.797100	3.865477

N2-2

Symbol	X	Y	Z
Si	1.587277	0.700004	-1.504207
H	-0.144833	0.163909	-0.790214
H	1.571391	1.704342	-0.431318
F	-6.018097	-0.062592	-0.132470
F	-1.331306	-4.847379	1.326630
F	-3.687735	0.111284	1.190014
C	-4.865393	-0.140484	-0.798100
C	0.495290	-3.596787	2.110443
C	-0.700781	-3.674944	1.424930
C	-3.651493	-0.039283	-0.142631
C	-4.876681	-0.332545	-2.170313
F	2.308220	-2.291746	2.849011
C	1.142898	-2.376396	2.205655
F	-2.379309	-2.714210	0.160476
C	-1.226714	-2.537408	0.825784
C	0.580997	-1.271361	1.598321
C	-0.607273	-1.297871	0.878336
C	-2.424981	-0.119323	-0.786944
C	-3.679170	-0.427898	-2.854543
F	1.266583	-0.117399	1.691312
C	-2.486505	-0.322291	-2.153988
B	-1.045949	0.025853	0.053613
F	-3.683186	-0.621393	-4.174758
F	-1.359416	-0.444099	-2.879172
C	-1.068250	1.415982	0.895354
F	-1.344204	0.443963	3.036993
F	-0.831014	2.611869	-1.129652
C	-1.212404	1.533268	2.269620
C	-0.963452	2.618899	0.211020
C	-1.229712	2.753708	2.927738
C	-0.968770	3.859333	0.825705
F	-1.366558	2.813113	4.253015
F	-0.844322	4.981267	0.113867
C	-1.106131	3.924873	2.201096
F	-6.032642	-0.431236	-2.823219
F	-1.118188	5.103800	2.819501
F	1.032972	-4.684847	2.658734
H	0.720739	0.857471	-2.678688
C	2.174663	-1.025003	-1.123351
C	3.244764	-1.296276	-0.258579

C	1.441134	-2.101194	-1.641296
C	3.584316	-2.607182	0.057992
H	3.806097	-0.487729	0.197166
C	1.771563	-3.411451	-1.312756
H	0.583845	-1.911632	-2.280724
C	2.846369	-3.664792	-0.465192
H	4.408783	-2.800363	0.733507
H	1.185076	-4.231962	-1.708858
H	3.099065	-4.685111	-0.200606
C	4.386629	1.379107	-1.650311
C	4.539830	2.127392	-0.488710
C	5.358132	0.474076	-2.055922
C	5.685194	1.951333	0.280077
H	3.777879	2.828455	-0.171684
C	6.498599	0.305935	-1.279525
H	5.218092	-0.111819	-2.957793
C	6.661492	1.039750	-0.109305
H	5.808148	2.526945	1.188686
H	7.254984	-0.403875	-1.589513
H	7.548109	0.903914	0.496999
N	3.150714	1.483554	-2.420479
C	2.835057	2.879343	-2.852293
H	1.984689	2.846190	-3.530416
H	2.572796	3.472638	-1.978490
H	3.701693	3.312028	-3.348772
H	3.274811	0.925802	-3.267515

N2-3

Symbol	X	Y	Z
Si	-3.487836	-0.091549	-2.159516
H	-0.443665	0.204866	0.005103
H	-4.842805	0.480847	-2.200008
F	3.389500	-3.964741	-1.355379
F	4.493629	2.930177	-1.693953
F	2.907713	-2.019664	0.401890
C	2.435310	-3.068742	-1.607728
C	2.474754	4.001945	-1.153470
C	3.248332	2.854825	-1.223405
C	2.157883	-2.051974	-0.706611
C	1.714604	-3.155370	-2.787276
F	0.422305	5.012534	-0.593387
C	1.187485	3.920219	-0.655831
F	3.518988	0.577826	-0.894086
C	2.716036	1.645607	-0.806463
C	0.704253	2.689809	-0.240160
C	1.426310	1.509746	-0.309617
C	1.172909	-1.097228	-0.917081
C	0.738021	-2.212259	-3.051430
F	-0.559422	2.682971	0.236818
C	0.502292	-1.215316	-2.121221
B	0.754931	0.087789	0.118842
F	0.011909	-2.290460	-4.170398
F	-0.473979	-0.327831	-2.440955
C	0.993661	-0.316276	1.676319
F	3.115264	0.712572	1.885458
F	-1.075405	-1.468973	1.701268
C	2.102389	0.028231	2.435764
C	0.033727	-1.061627	2.346172
C	2.248546	-0.317439	3.769909
C	0.135186	-1.427667	3.678671
F	3.336022	0.039673	4.455548
F	-0.827395	-2.137171	4.272360
C	1.255033	-1.049287	4.397044
F	1.961951	-4.130781	-3.657938
F	1.378734	-1.389994	5.678628
F	2.967131	5.170097	-1.558887
H	-2.856057	-0.195633	-3.481955
C	-3.294039	-1.601323	-1.116293
C	-4.020843	-1.778448	0.070585
C	-2.385543	-2.593363	-1.508914
C	-3.815754	-2.903736	0.858214
H	-4.746684	-1.034824	0.383971
C	-2.178275	-3.716957	-0.717640
H	-1.848980	-2.495424	-2.446120
C	-2.888327	-3.866475	0.468843
H	-4.374192	-3.028591	1.777768
H	-1.466605	-4.472598	-1.028159
H	-2.722930	-4.737894	1.090948
C	-3.299892	1.780829	-0.132537
C	-4.274663	2.758358	-0.279716
C	-3.038521	1.192926	1.098569
C	-4.996518	3.159416	0.839326
H	-4.475365	3.210116	-1.243082
C	-3.770126	1.601911	2.207467
H	-2.280401	0.423216	1.188396
C	-4.747080	2.583736	2.080503
H	-5.754648	3.925189	0.735262
H	-3.568596	1.151505	3.171308

H	-5.311511	2.902284	2.947864
N	-2.543370	1.304271	-1.303482
C	-2.154834	2.378735	-2.273985
H	-1.362407	1.981805	-2.905365
H	-3.011619	2.648398	-2.889369
H	-1.805234	3.245739	-1.717449
H	-1.666063	0.900910	-0.936651

N2-4

Symbol	X	Y	Z
F	0.899576	5.355081	-1.446118
F	5.004204	0.115203	-1.957669
F	0.533149	2.747133	-2.036212
C	0.841416	4.451588	-0.473242
C	3.659611	-1.803008	-1.815708
C	3.832906	-0.441044	-1.660878
C	0.645446	3.112756	-0.753956
C	0.983009	4.848922	0.847707
F	2.275009	-3.693114	-1.621324
C	2.440544	-2.380135	-1.498869
F	3.035497	1.641960	-1.052361
C	2.787793	0.335111	-1.178688
C	1.431404	-1.576212	-1.013783
C	1.552904	-0.202053	-0.841822
C	0.588994	2.135998	0.229567
C	0.931118	3.906919	1.859626
F	0.273123	-2.169577	-0.685980
C	0.739013	2.574155	1.532778
B	0.319746	0.625635	-0.246723
F	1.065136	4.284197	3.127324
F	0.690639	1.695693	2.550375
C	-1.139322	0.445408	-0.898116
F	-0.397081	-0.591854	-2.896593
F	-2.092288	1.447047	1.009759
C	-1.390910	-0.110441	-2.145655
C	-2.251113	0.930734	-0.218315
C	-2.667710	-0.195014	-2.676939
C	-3.536278	0.882658	-0.722305
F	-2.871248	-0.762080	-3.863277
F	-4.562451	1.369255	-0.033682
C	-3.742551	0.302817	-1.961945
F	1.170429	6.129549	1.139715
F	-4.970002	0.203694	-2.452200
F	4.662065	-2.562939	-2.235355
H	-0.227302	-0.271956	0.927873
Si	-0.443879	-3.003386	2.499441
H	0.441695	0.076497	1.140288
H	-0.877253	-4.116876	1.636440
C	1.366918	-2.658077	2.141137
C	2.121041	-3.664684	1.523079
C	2.017327	-1.458853	2.466143
C	3.470282	-3.480091	1.233739
H	1.649173	-4.602918	1.248394
C	3.357630	-1.256620	2.149665
H	1.486691	-0.662363	2.977836
C	4.086682	-2.269368	1.532539
H	4.031872	-4.270964	0.749730
H	3.833508	-0.313827	2.393645
H	5.131055	-2.115131	1.286191
H	-0.612729	-3.354121	3.931040
C	-2.693537	-1.725938	1.494939
C	-3.886361	-1.148842	1.946818
C	-2.717306	-2.428481	0.277611
C	-5.063532	-1.290955	1.213603
H	-3.913638	-0.585398	2.868982
C	-3.895216	-2.568032	-0.438910
H	-1.802063	-2.851275	-0.114939
C	-5.083294	-2.001159	0.022424
H	-5.970455	-0.828971	1.585472
H	-3.880146	-3.110196	-1.378043
H	-5.999579	-2.103367	-0.545144
N	-1.472676	-1.587227	2.205674
C	-1.505276	-0.675213	3.359480
H	-0.501603	-0.576541	3.775388
H	-1.836577	0.317041	3.051675
H	-2.159966	-1.040101	4.158911

N2-5

Symbol	X	Y	Z
Si	-1.329743	-4.720186	5.224329
H	-1.693618	-6.095049	4.806914
C	0.029254	-4.098209	4.091561
C	1.089792	-4.930087	3.713022
C	0.011952	-2.781837	3.616952
C	2.109821	-4.458576	2.893029
H	1.122533	-5.960159	4.056613
C	1.028462	-2.306961	2.793578
H	-0.811159	-2.126177	3.886212

C	2.079311	-3.144964	2.433170
H	2.924529	-5.114391	2.609151
H	0.999512	-1.286210	2.430705
H	2.871054	-2.777011	1.791151
H	-0.888146	-4.730451	6.634501
C	-3.522908	-3.509160	4.046443
C	-4.681453	-2.713867	4.059274
C	-3.196638	-4.165104	2.842864
C	-5.473591	-2.591508	2.922005
H	-4.976182	-2.187871	4.956263
C	-3.996885	-4.035520	1.718508
H	-2.304093	-4.776047	2.773785
C	-5.145033	-3.247582	1.743185
H	-6.361219	-1.970794	2.968108
H	-3.713047	-4.554830	0.810455
H	-5.765675	-3.147459	0.861804
N	-2.713894	-3.649124	5.178708
C	-3.086269	-2.882678	6.365130
H	-2.358024	-3.057163	7.154517
H	-3.104514	-1.809625	6.150198
H	-4.071622	-3.177398	6.740131

N2-6

Symbol	X	Y	Z
Si	-1.684602	-0.534588	-1.507963
H	1.077787	-0.245068	-1.188746
H	-0.953405	-1.471910	-2.387893
F	4.230766	4.413532	-1.554118
F	0.960646	0.167181	4.599186
F	4.262869	1.783332	-1.394345
C	3.166502	3.781136	-1.054596
C	0.626100	-1.846435	3.435113
C	1.011933	-0.520423	3.452860
C	3.152894	2.394753	-0.951126
C	2.068615	4.512232	-0.646960
F	0.305910	-3.833590	2.219579
C	0.669922	-2.548674	2.243972
F	1.829417	1.366763	2.393011
C	1.442212	0.084578	2.279756
C	1.073572	-1.895348	1.093899
C	1.471301	-0.562873	1.054909
C	2.082928	1.670889	-0.442549
C	0.974085	3.836982	-0.135556
F	1.050995	-2.616648	-0.039900
C	1.009862	2.459235	-0.044345
B	1.941399	0.041933	-0.392197
F	-0.108476	4.524649	0.257198
F	-0.090062	1.877428	0.471487
C	3.299921	-0.761551	-0.799267
F	4.450761	0.172359	1.030181
F	2.342627	-1.819832	-2.695283
C	4.443332	-0.666270	-0.019465
C	3.384016	-1.636548	-1.868543
C	5.604646	-1.372817	-0.270060
C	4.527704	-2.370019	-2.157640
F	6.683183	-1.236865	0.506371
F	4.562459	-3.203157	-3.202407
C	5.643495	-2.236741	-1.353420
F	2.058585	5.840995	-0.747293
F	6.750944	-2.931542	-1.617164
F	0.182291	-2.436796	4.546479
H	-1.114144	0.824576	-1.513114
C	-1.951556	-1.251259	0.185298
C	-2.179225	-2.627878	0.312568
C	-2.043814	-0.446091	1.327061
C	-2.519352	-3.181468	1.541476
H	-2.099656	-3.274451	-0.556097
C	-2.380744	-0.999708	2.558614
H	-1.841018	0.616254	1.261278
C	-2.626615	-2.366023	2.664795
H	-2.689198	-4.247911	1.626561
H	-2.439432	-0.364678	3.436068
H	-2.881819	-2.798440	3.625259
C	-4.355823	-1.195626	-2.113293
C	-4.877979	-1.966418	-3.153907
C	-4.925388	-1.308752	-0.837868
C	-5.946272	-2.829558	-2.919257
H	-4.458852	-1.905636	-4.149388
C	-5.986266	-2.174084	-0.612074
H	-4.518594	-0.729601	-0.016402
C	-6.505886	-2.939378	-1.653519
H	-6.336685	-3.420214	-3.739343
H	-6.406399	-2.248843	0.383953
H	-7.335676	-3.612406	-1.477770
N	-3.291583	-0.253005	-2.312008
C	-3.064827	0.135903	-3.718864
H	-2.367496	0.976389	-3.743046
H	-2.634852	-0.673707	-4.315777

H	-4.004890	0.447121	-4.180705
H	-3.902448	1.131821	-1.713807
C	-4.082152	2.276767	0.041684
C	-4.914822	1.754483	1.022639
C	-2.926249	2.973829	0.364062
C	-4.569250	1.937082	2.357220
H	-5.818030	1.215312	0.767265
C	-2.595517	3.155155	1.702458
H	-2.281717	3.369216	-0.413307
C	-3.414899	2.635120	2.698195
H	-5.208424	1.529833	3.130308
H	-1.689783	3.691346	1.953379
H	-3.154332	2.773251	3.740106
N	-4.380699	2.066340	-1.381085
C	-5.822200	2.021284	-1.762812
H	-5.880882	2.030065	-2.849025
H	-6.259345	1.100006	-1.386718
H	-6.329991	2.889154	-1.347686
H	-3.928783	2.816549	-1.906874

N2-7

Symbol	X	Y	Z
H	0.046699	-0.029298	1.446185
F	5.140806	-2.144429	0.201396
F	-2.146645	-2.192693	-3.384466
F	3.611540	-0.003862	0.041870
C	3.818989	-2.282443	0.320697
C	-3.022054	-0.275961	-2.351559
C	-2.014593	-1.212112	-2.489915
C	2.983791	-1.174876	0.232787
C	3.279672	-3.533771	0.541182
F	-3.824271	1.667980	-1.291378
C	-2.867229	0.749232	-1.434989
F	0.057974	-2.060433	-1.926969
C	-0.873300	-1.123014	-1.702867
C	-1.719366	0.785192	-0.666497
C	-0.689857	-0.145135	-0.735651
C	1.602535	-1.247679	0.343614
C	1.906875	-3.659813	0.674343
F	-1.646438	1.789548	0.240686
C	1.118255	-2.529772	0.579321
B	0.546913	-0.000873	0.334234
F	1.367719	-4.860702	0.893704
F	-0.208625	-2.719067	0.721134
C	1.212875	1.465498	0.108384
F	2.005500	0.841786	-2.017074
F	0.442448	2.323098	2.185253
C	1.861599	1.785219	-1.075480
C	1.098374	2.503162	1.014145
C	2.391309	3.034458	-1.342131
C	1.606536	3.774466	0.793656
F	3.019554	3.289134	-2.490314
F	1.463714	4.740167	1.703940
C	2.261390	4.038648	-0.394813
F	4.066359	-4.604348	0.630831
F	2.759187	5.250691	-0.631531
F	-4.129280	-0.359141	-3.086035
H	-1.150855	0.917774	2.263223
C	-3.019320	0.101574	2.072550
C	-4.225751	0.668956	1.701107
C	-2.665861	-1.192080	1.721006
C	-5.108914	-0.090015	0.939523
H	-4.467176	1.690434	1.972817
C	-3.550751	-1.933796	0.948758
H	-1.703460	-1.602545	1.997882
C	-4.768826	-1.383459	0.557554
H	-6.051224	0.342066	0.628603
H	-3.276431	-2.935903	0.643935
H	-5.450326	-1.960934	-0.054877
N	-2.034419	0.919187	2.802295
C	-1.756127	0.468516	4.200633
H	-1.026498	1.145902	4.637746
H	-2.689054	0.484429	4.757657
H	-1.355839	-0.540793	4.158139
H	-2.346857	1.893383	2.809855

N2-8

Symbol	X	Y	Z
H	-0.758827	0.182080	2.314631
F	-4.090041	3.656614	-1.138729
F	3.400219	0.430172	-2.820865
F	-3.448964	1.074153	-1.181691
C	-2.881620	3.271453	-0.744775
C	3.145399	-1.429868	-1.412523
C	2.661823	-0.250087	-1.950254
C	-2.523580	1.935530	-0.758777
C	-1.966493	4.214916	-0.307251
F	2.882019	-3.227818	0.073911

C	2.395948	-2.118242	-0.472393
F	0.983286	1.325940	-2.154662
C	1.410453	0.202429	-1.574288
C	1.150381	-1.639377	-0.118609
C	0.598318	-0.480679	-0.667908
C	-1.263088	1.485336	-0.366615
C	-0.703599	3.815611	0.098975
F	0.471128	-2.345493	0.787658
C	-0.373272	2.475169	0.047605
B	-0.868319	-0.031982	-0.358642
F	0.173297	4.717120	0.526698
F	0.857976	2.148514	0.444668
C	-1.970548	-1.117058	-0.079808
F	-1.032907	-2.665539	-1.607430
F	-3.093901	0.258051	1.486207
C	-1.974938	-2.357846	-0.714879
C	-3.009865	-0.898509	0.823437
C	-2.940598	-3.319368	-0.478205
C	-3.978801	-1.844003	1.101454
F	-2.912730	-4.483639	-1.117051
F	-4.939934	-1.597494	1.984407
C	-3.944977	-3.060412	0.439169
F	-2.298016	5.494618	-0.277159
F	-4.871053	-3.973080	0.683005
F	4.318055	-1.903292	-1.798001
H	-0.036723	0.004424	2.251811
N	2.391715	0.108758	2.824984
C	1.966428	1.367487	3.423233
H	1.448319	1.985611	2.689829
H	1.264722	1.148845	4.226840
H	2.808584	1.939880	3.830434
C	3.465677	0.111069	1.938570
C	3.757749	1.223884	1.139027
C	4.269478	-1.034503	1.813060
C	4.816984	1.179267	0.236189
H	3.156547	2.120344	1.214975
C	5.325352	-1.062998	0.916360
H	4.050727	-1.903835	2.425162
C	5.609173	0.044207	0.116220
H	5.019885	2.048452	-0.379277
H	5.928049	-1.960317	0.835583
H	6.428690	0.015646	-0.590563
H	2.452994	-0.642410	3.498124

N2-9

Symbol	X	Y	Z
Si	-1.935904	0.433496	-2.035989
H	0.614067	0.105511	-1.279795
H	-1.295148	-0.479820	-2.997612
F	4.455249	4.179177	-0.301731
F	-0.355337	-0.233078	4.425184
F	4.085275	1.590761	-0.656878
C	3.237381	3.665241	-0.112512
C	-0.811004	-1.979233	2.920542
C	-0.226879	-0.759845	3.202996
C	3.010262	2.304783	-0.285694
C	2.196361	4.495376	0.252833
F	-1.231475	-3.698277	1.371593
C	-0.677612	-2.515264	1.652180
F	1.038070	1.073338	2.581922
C	0.482699	-0.092959	2.213222
C	0.012286	-1.800866	0.689834
C	0.612228	-0.567272	0.917272
C	1.772120	1.706359	-0.102846
C	0.936975	3.950210	0.438938
F	0.063649	-2.348814	-0.537167
C	0.763983	2.592043	0.257981
B	1.384765	0.136245	-0.343937
F	-0.085527	4.737207	0.786188
F	-0.482435	2.117758	0.450728
C	2.648999	-0.827470	-0.698832
F	3.655472	-0.336121	1.371971
F	1.810346	-1.478340	-2.819819
C	3.670594	-1.024546	0.218877
C	2.745247	-1.572434	-1.861405
C	4.732466	-1.885275	0.012297
C	3.790516	-2.452135	-2.111453
F	5.697260	-2.031649	0.924233
F	3.841354	-3.151368	-3.249559
C	4.789143	-2.608190	-1.168912
F	2.397223	5.802592	0.418782
F	5.800273	-3.448203	-1.393389
F	-1.520567	-2.622403	3.849600
H	-1.222312	1.696978	-1.818203
C	-2.615296	-0.432106	-0.536276
C	-3.025665	-1.767070	-0.657913
C	-2.867895	0.236938	0.670523
C	-3.687122	-2.410786	0.382351

H	-2.829427	-2.313238	-1.575371
C	-3.511138	-0.413002	1.719603
H	-2.557347	1.267526	0.801553
C	-3.932509	-1.732357	1.572411
H	-3.989723	-3.445017	0.272422
H	-3.679766	0.109908	2.654364
H	-4.431794	-2.236272	2.391851
C	-5.692841	0.271539	-1.361257
C	-5.174384	1.605826	-1.266674
C	-5.328775	-0.539206	-2.489211
C	-4.184206	1.994999	-2.107804
H	-5.555854	2.280974	-0.513246
C	-4.335945	-0.135737	-3.314914
H	-5.834154	-1.486122	-2.636848
C	-3.587898	1.077683	-3.055056
H	-3.769503	2.993336	-2.023156
H	-4.034079	-0.763419	-4.145980
H	-3.096409	1.531753	-3.915528
N	-6.507770	-0.214818	-0.442286
C	-6.933430	0.496252	0.755695
H	-7.480502	-0.198814	1.386575
H	-6.062361	0.860384	1.302628
H	-7.585180	1.333753	0.500373
H	-6.828757	-1.166021	-0.560187

N2-10

Symbol	X	Y	Z
Si	-1.462472	-1.716186	0.270896
H	-1.582434	-2.787096	-0.751517
H	-1.721880	-2.332830	1.597877
C	-2.771906	-0.389417	0.024488
C	-2.570492	0.664381	-0.876933
C	-3.983925	-0.436723	0.723858
C	-3.549052	1.632437	-1.079003
H	-1.633957	0.734037	-1.422404
C	-4.966923	0.528509	0.524895
H	-4.163874	-1.234737	1.437773
C	-4.750018	1.564294	-0.378342
H	-3.374951	2.440657	-1.779825
H	-5.898331	0.475000	1.076504
H	-5.512521	2.318757	-0.533056
C	2.866699	0.098387	-0.034123
C	2.566438	-1.148467	-0.606762
C	1.830686	0.801676	0.618361
C	1.276949	-1.663441	-0.521145
H	3.337633	-1.705841	-1.122792
C	0.561075	0.268072	0.696148
H	2.052849	1.771428	1.051168
C	0.246091	-0.983143	0.131325
H	1.076676	-2.626980	-0.980820
H	-0.213752	0.837624	1.201493
N	4.118840	0.650887	-0.124289
C	5.260556	-0.112162	-0.576062
H	6.156258	0.492512	-0.450871
H	5.388386	-1.046769	-0.015100
H	5.167776	-0.364247	-1.636404
H	4.309281	1.418240	0.499529

N2-11

Symbol	X	Y	Z
F	3.048818	4.687678	-1.167159
F	5.448048	-1.319154	-1.131566
F	2.040168	2.246878	-1.687555
C	2.573811	3.931154	-0.174419
C	3.599603	-2.736083	-1.433616
C	4.130930	-1.522394	-1.044052
C	2.044472	2.676929	-0.414866
C	2.610945	4.404769	1.127717
F	1.704101	-4.101248	-1.706188
C	2.232736	-2.930640	-1.339378
F	3.911335	0.614864	-0.209908
C	3.292329	-0.523080	-0.565305
C	1.443816	-1.914346	-0.836287
C	1.916567	-0.667928	-0.444878
C	1.548250	1.847138	0.579990
C	2.122629	3.617124	2.153111
F	0.129414	-2.182731	-0.731090
C	1.602744	2.362427	1.863097
B	0.876281	0.409604	0.209262
F	2.153657	4.073374	3.408723
F	1.140033	1.658427	2.908456
C	-0.441144	0.758505	-0.712180
F	0.236936	-0.275553	-2.739294
F	-1.345983	1.898190	1.154726
C	-0.667743	0.423227	-2.040081
C	-1.450526	1.523999	-0.133046
C	-1.817814	0.776126	-2.735596
C	-2.597385	1.923761	-0.796207

F -1.992042 0.397746 -4.003462
F -3.543777 2.644316 -0.181930
C -2.790594 1.531699 -2.109077
F 3.114387 5.611501 1.387968
F -3.915133 1.862842 -2.748479
F 4.389303 -3.707995 -1.890937
H -1.395680 -0.673823 1.670057
Si -2.307286 -2.555201 2.758776
H 0.469912 -0.098810 1.230526
H -3.532033 -3.366748 2.609316
C -0.775243 -3.459305 2.215600
C -0.847882 -4.638724 1.465870
C 0.483853 -2.899518 2.473465
C 0.308918 -5.245216 0.986941
H -1.812592 -5.086450 1.248199
C 1.640244 -3.506769 1.998471
H 0.567815 -1.978818 3.043552
C 1.551260 -4.681048 1.256095
H 0.241958 -6.152960 0.399576
H 2.607634 -3.059062 2.195574
H 2.451206 -5.149278 0.873698
H -2.185246 -1.965864 4.106651
C -4.684983 -0.240331 -0.016529
C -4.507428 0.283101 1.309898
C -3.764609 -1.222946 -0.519014
C -3.410133 -0.061388 2.021265
H -5.211297 1.004943 1.699682
C -2.668462 -1.549462 0.197512
H -3.927209 -1.630161 -1.510408
C -2.431316 -1.026031 1.540024
H -3.242547 0.394845 2.991041
H -1.940688 -2.237594 -0.215418
N -5.650243 0.200136 -0.798822
C -6.594760 1.258524 -0.457600
H -7.230534 1.433648 -1.320408
H -7.216181 0.960605 0.387818
H -6.056501 2.176227 -0.218124
H -5.707631 -0.188713 -1.731062

N2-12

Symbol X Y Z
F 1.774201 5.006691 -1.695451
F 5.174004 -0.533696 -1.753416
F 1.209697 2.412228 -2.186866
C 1.458323 4.189779 -0.695442
C 3.633346 -2.303016 -1.714029
C 3.934835 -0.965769 -1.536709
C 1.163277 2.860111 -0.925852
C 1.423413 4.669518 0.605115
F 2.064592 -4.048126 -1.569327
C 2.350818 -2.754780 -1.455543
F 3.338826 1.172859 -0.912433
C 2.952170 -0.091237 -1.098368
C 1.396061 -1.851649 -1.034461
C 1.644702 -0.495313 -0.850217
C 0.842465 1.969088 0.087560
C 1.094058 3.817947 1.644180
F 0.175221 -2.333228 -0.775437
C 0.805917 2.490087 1.369037
B 0.468712 0.472318 -0.347714
F 1.049012 4.278151 2.891012
F 0.466187 1.713593 2.409630
C -0.982178 0.380577 -1.034554
F -0.283729 -0.792287 -2.973645
F -1.895210 1.553685 0.795794
C -1.253761 -0.220948 -2.255709
C -2.068775 0.981355 -0.402599
C -2.528014 -0.253025 -2.803221
C -3.344192 0.989866 -0.929966
F -2.746661 -0.855261 -3.969641
F -4.351974 1.556617 -0.273814
C -3.577541 0.352262 -2.135658
F 1.702158 5.943523 0.850648
F -4.800532 0.316639 -2.644337
F 4.577401 -3.155577 -2.087913
H -0.217118 -0.149318 0.908091
Si -0.439320 -1.613317 3.722882
H 0.547261 -0.216674 1.039124
H -0.676702 -2.724792 4.676572
C 1.203441 -1.946317 2.858982
C 1.486144 -3.210787 2.319579
C 2.187425 -0.952114 2.751096
C 2.702873 -3.473214 1.698297
H 0.756905 -4.011336 2.401691
C 3.404047 -1.207457 2.120196
H 2.008123 0.031849 3.172425
C 3.664823 -2.470597 1.599639
H 2.902120 -4.458664 1.292721

H	4.149525	-0.423526	2.045570
H	4.616211	-2.676168	1.121470
H	-0.338517	-0.337645	4.470371
C	-4.087017	-1.457451	0.739937
C	-4.050605	-0.638451	1.880404
C	-2.982171	-2.291163	0.484268
C	-2.947930	-0.662141	2.725234
H	-4.876081	0.026811	2.098271
C	-1.902878	-2.313761	1.348781
H	-2.985380	-2.921757	-0.399499
C	-1.848596	-1.498556	2.493397
H	-2.951337	-0.003578	3.588672
H	-1.077335	-2.978036	1.117097
N	-5.130287	-1.409825	-0.156930
C	-6.408370	-0.814790	0.187937
H	-7.096584	-0.978857	-0.639084
H	-6.838623	-1.246040	1.099591
H	-6.302387	0.262089	0.325843
H	-5.176547	-2.191334	-0.793286

N2-TS1

Symbol	X	Y	Z
Si	0.995291	-0.093402	-1.552077
H	-0.317239	0.027497	-0.628392
H	0.874216	1.218004	-2.208426
F	-5.507919	-2.418830	-0.944187
F	0.462305	-2.139427	4.453062
F	-4.267037	-0.203564	-0.170029
C	-4.191556	-2.441852	-0.749759
C	1.331978	-0.047838	3.834787
C	0.455315	-1.095261	3.625544
C	-3.516747	-1.296064	-0.351452
C	-3.490300	-3.617198	-0.943813
F	2.152192	2.054216	3.176898
C	1.315807	1.038747	2.976417
F	-1.241708	-2.102579	2.422728
C	-0.421159	-1.052944	2.550406
C	0.434724	1.037380	1.913425
C	-0.454809	0.000293	1.646281
C	-2.146314	-1.269156	-0.130977
C	-2.121125	-3.639143	-0.730406
F	0.487251	2.089349	1.083189
C	-1.492581	-2.478248	-0.326791
B	-1.324105	0.044692	0.299774
F	-1.437194	-4.766871	-0.907602
F	-0.167664	-2.546006	-0.107756
C	-2.017426	1.433593	-0.111331
F	-2.244951	2.233639	2.102939
F	-1.913919	0.812357	-2.385447
C	-2.425319	2.405941	0.788555
C	-2.265756	1.710275	-1.448528
C	-3.034716	3.586004	0.392236
C	-2.863927	2.877165	-1.887677
F	-3.411282	4.492860	1.291131
F	-3.071526	3.097692	-3.184084
C	-3.254562	3.822065	-0.953584
F	-4.123082	-4.719463	-1.327197
F	-3.835814	4.949581	-1.347849
F	2.193474	-0.084656	4.844797
H	0.544074	-1.274010	-2.290886
C	2.318263	-0.167690	-0.261405
C	3.057757	0.997802	-0.014288
C	2.573929	-1.307295	0.510897
C	4.022993	1.025032	0.983574
H	2.875937	1.892305	-0.600020
C	3.536977	-1.276223	1.515316
H	2.019401	-2.222109	0.339891
C	4.255631	-0.109391	1.756348
H	4.586345	1.932287	1.164263
H	3.719278	-2.160961	2.114236
H	4.996168	-0.083389	2.547231
C	4.164544	-0.131084	-2.776456
C	4.478510	1.204486	-3.045994
C	5.029425	-0.892056	-1.989547
C	5.637770	1.768477	-2.533677
H	3.802737	1.797745	-3.653980
C	6.186334	-0.314528	-1.475522
H	4.795759	-1.922252	-1.753560
C	6.499331	1.012774	-1.741851
H	5.868051	2.804289	-2.753542
H	6.842997	-0.913244	-0.855151
H	7.402229	1.454697	-1.339299
N	2.937418	-0.653185	-3.253457
C	2.811815	-2.111368	-3.336048
H	3.714612	-2.577904	-3.740499
H	2.612422	-2.527602	-2.343368
H	1.967879	-2.349923	-3.982637
H	2.672669	-0.209810	-4.124902

N2-TS2

Symbol	X	Y	Z
F	0.083016	5.409005	-1.462699
F	4.886603	0.757772	-1.971192
F	0.022706	2.774692	-2.029367
C	0.196654	4.513412	-0.486469
C	3.808977	-1.320974	-1.818386
C	3.798306	0.052539	-1.672625
C	0.157092	3.158025	-0.753915
C	0.364841	4.937401	0.822835
F	2.679822	-3.375466	-1.635270
C	2.675048	-2.052628	-1.503619
F	2.731843	2.015702	-1.081924
C	2.657350	0.686138	-1.198412
C	1.568540	-1.385144	-1.024232
C	1.505364	-0.007575	-0.853743
C	0.281150	2.189134	0.231415
C	0.495601	4.004485	1.835725
F	0.499132	-2.127256	-0.689253
C	0.455231	2.655146	1.521675
B	0.186080	0.646075	-0.218882
F	0.658170	4.406241	3.093344
F	0.584550	1.788780	2.542737
C	-1.250913	0.302856	-0.853176
F	-0.431771	-0.645854	-2.865166
F	-2.281886	1.194798	1.069242
C	-1.460756	-0.280433	-2.095234
C	-2.399307	0.660907	-0.156642
C	-2.728748	-0.518101	-2.602063
C	-3.679904	0.455979	-0.632815
F	-2.886486	-1.110118	-3.783744
F	-4.743358	0.810468	0.080988
C	-3.841111	-0.150797	-1.866130
F	0.403920	6.234567	1.102121
F	-5.058579	-0.405449	-2.326745
F	4.904655	-1.943578	-2.232696
H	-0.236362	-0.425755	1.045158
Si	0.047245	-3.009558	2.315492
H	0.350785	0.140865	1.095506
H	-0.282624	-4.110394	1.395843
C	1.825537	-2.504777	2.002682
C	2.661762	-3.483513	1.446786
C	2.380201	-1.245408	2.272296
C	3.998895	-3.217972	1.166760
H	2.263540	-4.465287	1.209678
C	3.709327	-0.966931	1.968425
H	1.785489	-0.455024	2.718380
C	4.521725	-1.953795	1.417016
H	4.622725	-3.989250	0.730713
H	4.111106	0.019679	2.167733
H	5.556595	-1.735691	1.179465
H	-0.146769	-3.431711	3.722947
C	-2.373442	-1.971928	1.491566
C	-3.572282	-1.567025	2.084796
C	-2.431178	-2.680142	0.281351
C	-4.793100	-1.869670	1.483144
H	-3.574587	-1.006792	3.008873
C	-3.651115	-2.981831	-0.303275
H	-1.516116	-2.975023	-0.214360
C	-4.845393	-2.577684	0.291959
H	-5.707775	-1.535115	1.957644
H	-3.666818	-3.521422	-1.243629
H	-5.796109	-2.803333	-0.174305
N	-1.096072	-1.648669	2.058194
C	-1.151361	-0.807101	3.272576
H	-0.136093	-0.596325	3.611194
H	-1.634903	0.144749	3.053328
H	-1.680408	-1.304351	4.090982

N2-TS3

Symbol	X	Y	Z
Si	-1.422792	-0.299362	-1.568570
H	0.919451	-0.206452	-0.703483
H	-0.597545	-1.077374	-2.512107
F	4.180112	4.439514	-1.023638
F	0.703411	0.159394	4.947871
F	4.255323	1.811777	-0.794154
C	3.094167	3.794242	-0.592057
C	0.563834	-1.890589	3.807858
C	0.861866	-0.543081	3.820703
C	3.098464	2.409061	-0.466340
C	1.958874	4.510528	-0.269584
F	0.450371	-3.913374	2.615800
C	0.733021	-2.607588	2.636119
F	1.612138	1.379109	2.776041
C	1.321489	0.070349	2.661950
C	1.165357	-1.949955	1.500711

C	1.471930	-0.593284	1.455688
C	2.004677	1.676467	-0.027659
C	0.844694	3.825555	0.185805
F	1.286028	-2.694432	0.388119
C	0.897690	2.450660	0.298752
B	1.864448	0.046282	0.010060
F	-0.268071	4.501821	0.505085
F	-0.211080	1.851137	0.769246
C	3.133047	-0.693572	-0.693182
F	4.217148	-1.344810	1.311400
F	2.276827	-0.104631	-2.817428
C	4.172783	-1.325077	-0.031502
C	3.215236	-0.723313	-2.077771
C	5.220217	-1.957928	-0.685235
C	4.240980	-1.339445	-2.773394
F	6.197479	-2.554912	0.002558
F	4.271466	-1.334056	-4.109453
C	5.253764	-1.966143	-2.067421
F	1.934230	5.837347	-0.389182
F	6.253041	-2.567006	-2.714872
F	0.094737	-2.494029	4.902256
H	-0.961449	1.093059	-1.433955
C	-1.736681	-1.207919	0.017428
C	-1.779737	-2.607886	-0.003088
C	-2.053850	-0.542919	1.208334
C	-2.147217	-3.324473	1.130147
H	-1.528407	-3.146351	-0.911791
C	-2.421895	-1.258705	2.343335
H	-2.012484	0.538276	1.257095
C	-2.474440	-2.649225	2.302860
H	-2.168534	-4.407036	1.102626
H	-2.659219	-0.728623	3.259300
H	-2.750259	-3.207988	3.190030
C	-3.951127	-1.179126	-2.370368
C	-4.194273	-2.017579	-3.456961
C	-4.628762	-1.400483	-1.167794
C	-5.109549	-3.061119	-3.337872
H	-3.679406	-1.869134	-4.396933
C	-5.538308	-2.442445	-1.057270
H	-4.415899	-0.774088	-0.308755
C	-5.784591	-3.277959	-2.143934
H	-5.288993	-3.706403	-4.189203
H	-6.049957	-2.604143	-0.116192
H	-6.494112	-4.091334	-2.057561
N	-3.023903	-0.071495	-2.440488
C	-2.762364	0.424596	-3.812381
H	-2.199074	1.358244	-3.743499
H	-2.173474	-0.280284	-4.403007
H	-3.706060	0.616284	-4.327246
H	-3.693626	1.047672	-1.865217
C	-4.132989	2.156182	-0.041745
C	-5.005308	1.545983	0.852221
C	-3.041085	2.888796	0.409222
C	-4.766626	1.671022	2.217146
H	-5.862490	0.982739	0.505650
C	-2.819586	3.017054	1.775264
H	-2.360990	3.355205	-0.295712
C	-3.679308	2.404597	2.680855
H	-5.439801	1.193098	2.917730
H	-1.963639	3.582694	2.119463
H	-3.503194	2.499443	3.744978
N	-4.303592	2.005431	-1.482932
C	-5.699589	1.897196	-1.982161
H	-5.670688	1.934483	-3.069300
H	-6.118393	0.942361	-1.673906
H	-6.299431	2.718553	-1.593383
H	-3.860299	2.808700	-1.928930

N2-TS4

Symbol	X	Y	Z
F	3.247385	-3.101206	2.429892
F	4.771153	2.866890	0.313368
F	1.922164	-0.789979	2.008160
C	2.737246	-2.831741	1.231687
C	2.765045	3.946085	-0.247148
C	3.475341	2.792337	0.023973
C	2.051513	-1.655970	0.995806
C	2.892733	-3.739800	0.195008
F	0.722223	4.963643	-0.820735
C	1.415862	3.861252	-0.553370
F	3.599499	0.498610	0.247910
C	2.833891	1.561222	-0.014114
C	0.817781	2.617962	-0.575239
C	1.482432	1.428123	-0.303871
C	1.513085	-1.329049	-0.240746
C	2.363826	-3.457742	-1.051296
F	-0.492980	2.575415	-0.874558
C	1.686628	-2.263492	-1.245685

B	0.668365	0.041482	-0.346753
F	2.505061	-4.329477	-2.046402
F	1.179990	-2.040336	-2.469098
C	-0.679430	-0.085966	0.521673
F	-0.300039	1.812388	1.889786
F	-1.273396	-1.970387	-0.755663
C	-1.048111	0.753673	1.562665
C	-1.530879	-1.156484	0.280539
C	-2.200710	0.554316	2.305987
C	-2.676697	-1.402961	1.012048
F	-2.539726	1.403885	3.273239
F	-3.458846	-2.440509	0.732838
C	-3.017637	-0.526862	2.027862
F	3.546899	-4.876634	0.400280
F	-4.139924	-0.706720	2.711960
F	3.369027	5.126214	-0.219630
H	-0.293743	0.198095	-1.655879
H	0.462844	-0.110211	-1.725065
C	-3.092382	0.413730	-1.733322
C	-3.939704	-0.684604	-1.879210
C	-3.437694	1.434455	-0.839556
C	-5.109114	-0.763435	-1.125094
H	-3.691311	-1.487366	-2.560725
C	-4.605431	1.346016	-0.097557
H	-2.774676	2.284426	-0.719958
C	-5.446808	0.241068	-0.228655
H	-5.751467	-1.628309	-1.238975
H	-4.853310	2.138562	0.599431
H	-6.350947	0.168748	0.362367
N	-1.855050	0.503594	-2.426961
C	-1.672123	-0.370266	-3.590038
H	-0.769183	-0.060153	-4.115156
H	-1.534095	-1.398682	-3.252846
H	-2.518200	-0.327844	-4.281385
H	-1.680754	1.472802	-2.675511

N2-TS5

Symbol	X	Y	Z
Si	-1.289771	-0.753294	-1.712479
H	0.242523	-0.148994	-0.641496
H	-0.787316	-2.109847	-1.443691
F	5.705625	1.927449	-0.960662
F	-0.298329	3.701738	3.280477
F	4.147654	0.227984	0.370653
C	4.384051	1.972237	-1.134836
C	-1.217228	1.558796	3.562983
C	-0.346598	2.416575	2.921273
C	3.548452	1.090189	-0.464679
C	3.837178	2.912222	-1.988388
F	-2.101863	-0.607901	3.791551
C	-1.259019	0.228266	3.182392
F	1.265448	2.848461	1.325056
C	0.460317	1.938311	1.896577
C	-0.445059	-0.202208	2.153427
C	0.439332	0.620359	1.463847
C	2.169183	1.090226	-0.609680
C	2.462982	2.964038	-2.150462
F	-0.558843	-1.493540	1.798903
C	1.669964	2.065461	-1.460193
B	1.185444	0.041742	0.145875
F	1.924558	3.879558	-2.958337
F	0.339933	2.195120	-1.621354
C	1.882234	-1.417399	0.283041
F	2.150974	-1.387621	2.634392
F	1.768208	-1.649822	-2.066895
C	2.297640	-2.018688	1.460781
C	2.116343	-2.155408	-0.868162
C	2.885990	-3.272527	1.505588
C	2.698477	-3.412111	-0.872150
F	3.266019	-3.809188	2.666492
F	2.889419	-4.079252	-2.012050
C	3.087753	-3.974918	0.330137
F	4.622307	3.767233	-2.640354
F	3.652181	-5.181033	0.356629
F	-2.022155	2.005229	4.526223
H	-0.562389	0.099522	-2.659356
C	-2.374949	0.002967	-0.406878
C	-3.110673	-0.816442	0.458249
C	-2.482785	1.392456	-0.268972
C	-3.947070	-0.264344	1.423818
H	-3.023454	-1.895604	0.391374
C	-3.309094	1.945893	0.703348
H	-1.908009	2.047604	-0.914457
C	-4.047312	1.117964	1.546052
H	-4.497854	-0.911655	2.095843
H	-3.371796	3.022677	0.808675
H	-4.684322	1.550332	2.309075
C	-5.221125	-0.509893	-2.113828

C	-4.484862	0.367160	-2.966228
C	-4.791287	-1.862173	-1.979756
C	-3.313507	-0.046173	-3.522352
H	-4.861305	1.368395	-3.139145
C	-3.607551	-2.254593	-2.536985
H	-5.387362	-2.565283	-1.414331
C	-2.761001	-1.332896	-3.221122
H	-2.751124	0.631903	-4.154495
H	-3.275216	-3.279782	-2.415465
H	-2.007929	-1.736967	-3.893711
N	-6.295798	-0.056966	-1.467821
C	-7.093003	-0.847641	-0.545765
H	-7.849799	-0.202638	-0.107438
H	-7.590031	-1.672336	-1.061391
H	-6.464856	-1.247069	0.254269
H	-6.546461	0.911933	-1.597143

N2-TS6

Symbol	X	Y	Z
Si	1.655499	0.385962	1.242302
H	-0.872918	0.116840	0.820800
H	1.350330	-0.580529	0.165817
F	-6.552365	-1.476065	0.351585
F	-3.776628	4.539980	-0.961516
F	-4.382354	-0.728701	-1.048129
C	-5.427664	-1.138271	0.987317
C	-1.702490	4.001376	-1.923180
C	-2.799732	3.650005	-1.164447
C	-4.294086	-0.759308	0.291456
C	-5.389489	-1.162281	2.372371
F	0.382171	3.408267	-2.838221
C	-0.691853	3.074840	-2.116971
F	-3.965354	2.141879	0.141981
C	-2.868460	2.382208	-0.598969
C	-0.803147	1.828414	-1.534119
C	-1.877091	1.425586	-0.747088
C	-3.103993	-0.392646	0.902363
C	-4.226539	-0.803317	3.027707
F	0.222509	0.981419	-1.739207
C	-3.113909	-0.428269	2.286665
B	-1.782975	0.000492	0.036996
F	-4.187836	-0.822717	4.363364
F	-2.023401	-0.084300	2.994149
C	-1.414643	-1.269357	-0.925535
F	-2.153458	-0.320944	-2.969247
F	-0.618272	-2.451320	0.969568
C	-1.621486	-1.350088	-2.295498
C	-0.870362	-2.412016	-0.354129
C	-1.296014	-2.464318	-3.055972
C	-0.560475	-3.557058	-1.068667
F	-1.499221	-2.476869	-4.374999
F	-0.054335	-4.639276	-0.462247
C	-0.759850	-3.577857	-2.436888
F	-6.468034	-1.525655	3.066834
F	-0.429967	-4.658925	-3.144769
F	-1.598360	5.226998	-2.440367
H	1.080380	-0.036574	2.538064
C	1.211188	2.175022	0.918363
C	2.008101	2.976781	0.092465
C	0.073024	2.743623	1.504625
C	1.671657	4.304371	-0.153997
H	2.898465	2.563374	-0.372669
C	-0.264629	4.070999	1.261779
H	-0.567751	2.135973	2.136843
C	0.533312	4.851967	0.429954
H	2.290558	4.907654	-0.807447
H	-1.155489	4.493044	1.712842
H	0.263233	5.882794	0.230339
C	6.042819	1.638889	0.979933
C	5.370198	1.714265	2.237300
C	5.458946	0.875321	-0.065271
C	4.184875	1.070663	2.422482
H	5.817078	2.298402	3.033966
C	4.262714	0.239077	0.143547
H	5.951115	0.803773	-1.025720
C	3.564196	0.281406	1.394064
H	3.691922	1.150050	3.387416
H	3.826891	-0.327291	-0.675544
H	3.509856	-0.971569	1.819301
N	7.202572	2.282038	0.811684
C	7.964052	2.295318	-0.425138
H	8.853665	2.900670	-0.273014
H	8.271638	1.285182	-0.705140
H	7.380020	2.728919	-1.240287
C	3.766945	-2.952792	0.829516
C	2.704330	-3.470377	0.094743
C	5.046837	-2.901043	0.276800
C	2.929901	-3.938340	-1.196731

H	1.711294	-3.523041	0.520522
C	5.260196	-3.370880	-1.012195
H	5.868115	-2.487046	0.853079
C	4.201732	-3.886973	-1.755264
H	2.103068	-4.352951	-1.761387
H	6.256161	-3.332690	-1.436084
H	4.369327	-4.252621	-2.760395
N	3.558943	-2.356519	2.116385
C	2.374815	-2.776629	2.887287
H	2.409133	-2.282437	3.856215
H	1.467934	-2.465302	2.371837
H	2.360519	-3.859630	3.022538
H	7.555412	2.821287	1.587392
H	4.397944	-2.454623	2.683939

N2-TS7

Symbol	X	Y	Z
F	3.247385	-3.101206	2.429892
F	4.771153	2.866890	0.313368
F	1.922164	-0.789979	2.008160
C	2.737246	-2.831741	1.231687
C	2.765045	3.946085	-0.247148
C	3.475341	2.792337	0.023973
C	2.051513	-1.655970	0.995806
C	2.892733	-3.739800	0.195008
F	0.722223	4.963643	-0.820735
C	1.415862	3.861252	-0.553370
F	3.599499	0.498610	0.247910
C	2.833891	1.561222	-0.014114
C	0.817781	2.617962	-0.575239
C	1.482432	1.428123	-0.303871
C	1.513085	-1.329049	-0.240746
C	2.363826	-3.457742	-1.051296
F	-0.492980	2.575415	-0.874558
C	1.686628	-2.263492	-1.245685
B	0.668365	0.041482	-0.346753
F	2.505061	-4.329477	-2.046402
F	1.179990	-2.040336	-2.469098
C	-0.679430	-0.085966	0.521673
F	-0.300039	1.812388	1.889786
F	-1.273396	-1.970387	-0.755663
C	-1.048111	0.753673	1.562665
C	-1.530879	-1.156484	0.280539
C	-2.200710	0.554316	2.305987
C	-2.676697	-1.402961	1.012048
F	-2.539726	1.403885	3.273239
F	-3.458846	-2.440509	0.732838
C	-3.017637	-0.526862	2.027862
F	3.546899	-4.876634	0.400280
F	-4.139924	-0.706720	2.711960
F	3.369027	5.126214	-0.219630
H	-0.293743	0.198095	-1.655879
H	0.462844	-0.110211	-1.725065
C	-3.092382	0.413730	-1.733322
C	-3.939704	-0.684604	-1.879210
C	-3.437694	1.434455	-0.839556
C	-5.109114	-0.763435	-1.125094
H	-3.691311	-1.487366	-2.560725
C	-4.605431	1.346016	-0.097557
H	-2.774676	2.284426	-0.719958
C	-5.446808	0.241068	-0.228655
H	-5.751467	-1.628309	-1.238975
H	-4.853310	2.138562	0.599431
H	-6.350947	0.168748	0.362367
N	-1.855050	0.503594	-2.426961
C	-1.672123	-0.370266	-3.590038
H	-0.769183	-0.060153	-4.115156
H	-1.534095	-1.398682	-3.252846
H	-2.518200	-0.327844	-4.281385
H	-1.680754	1.472802	-2.675511

Ph_2NH-add

Symbol	X	Y	Z
B	-0.103942	-0.023401	0.075175
C	1.135002	-0.408729	-0.929369
C	2.308519	0.348045	-0.942326
C	3.417517	0.047306	-1.711672
C	3.383389	-1.043212	-2.561366
C	2.232565	-1.803191	-2.625266
C	1.146880	-1.469387	-1.829542
C	-0.408306	1.599733	0.120501
C	-1.181581	2.163587	1.129358
C	-1.578789	3.486615	1.179243
C	-1.229777	4.327382	0.138586
C	-0.498833	3.816224	-0.916543
C	-0.112638	2.483290	-0.915805
C	-1.574036	-0.646940	-0.300271
C	-2.024246	-0.512977	-1.611356

C	-3.280480	-0.897605	-2.039453
C	-4.177095	-1.421697	-1.123174
C	-3.797829	-1.535892	0.199443
C	-2.525413	-1.137654	0.584525
F	2.416820	1.456169	-0.198975
F	4.511690	0.803039	-1.644122
F	4.435708	-1.347759	-3.310605
F	2.168533	-2.845678	-3.450803
F	0.074373	-2.264385	-1.984671
F	-1.596665	1.394218	2.163046
F	-2.293560	3.946471	2.202427
F	-1.599827	5.601930	0.149114
F	-0.171777	4.607929	-1.934836
F	0.563885	2.093639	-2.000976
F	-2.265383	-1.243046	1.900426
F	-4.655952	-2.012985	1.098386
F	-5.391724	-1.794276	-1.510378
F	-3.640191	-0.757773	-3.313142
F	-1.217734	0.030903	-2.532537
N	0.339309	-0.527464	1.622719
H	-0.389761	-0.120320	2.213521
C	1.611903	0.071171	2.075155
C	1.587157	1.283921	2.746894
C	2.807963	-0.583562	1.807773
C	2.790242	1.875235	3.119239
H	0.646262	1.762337	2.991218
C	4.002741	0.021118	2.174800
H	2.805736	-1.548194	1.313999
C	3.996774	1.253594	2.822134
H	2.777332	2.822294	3.643683
H	4.939709	-0.475555	1.955956
H	4.931345	1.720283	3.107149
C	0.316976	-1.972583	1.977282
C	0.431395	-2.274612	3.332034
C	0.179914	-2.974463	1.035876
C	0.412606	-3.597968	3.743465
H	0.540077	-1.476413	4.058151
C	0.168838	-4.304385	1.458694
H	0.050822	-2.752968	-0.009197
C	0.286377	-4.619571	2.803946
H	0.496828	-3.830929	4.797496
H	0.060749	-5.089655	0.721004
H	0.275276	-5.653927	3.124121

Ph_2NH

Symbol	X	Y	Z
C	0.672438	0.135914	0.390087
C	-0.146940	1.256551	0.203017
C	0.187200	-1.124472	0.015399
C	-1.406632	1.111647	-0.366041
H	0.192489	2.233623	0.521677
C	-1.079446	-1.257935	-0.535219
H	0.816392	-1.996174	0.158589
C	-1.884047	-0.140295	-0.740858
H	-2.025685	1.990693	-0.502897
H	-1.434643	-2.242897	-0.814812
H	-2.867844	-0.244538	-1.180694
N	1.931889	0.216610	0.984841
H	2.256478	-0.621157	1.443427
C	2.797458	1.309023	1.029412
C	2.787599	2.321005	0.060207
C	3.751341	1.367754	2.054742
C	3.696313	3.369390	0.137845
H	2.085551	2.274863	-0.762218
C	4.664637	2.410950	2.110847
H	3.765157	0.587366	2.807849
C	4.639421	3.427024	1.159383
H	3.672476	4.142446	-0.621464
H	5.394451	2.433391	2.911726
H	5.345560	4.246095	1.210473

Ph_2NH-product

Symbol	X	Y	Z
Si	-0.459579	-0.328055	0.575539
C	-0.163418	1.494082	0.248991
C	-0.527705	2.465064	1.189385
C	0.380155	1.921127	-0.969528
C	-0.359099	3.820930	0.921553
H	-0.947193	2.165734	2.145253
C	0.549902	3.274465	-1.242320
H	0.684149	1.183832	-1.707467
C	0.179266	4.226120	-0.295724
H	-0.645517	4.559247	1.661367
H	0.974704	3.587672	-2.188863
H	0.313004	5.280891	-0.505627
C	2.098926	-1.073344	-0.358827
C	2.940154	-1.775987	-1.237683
C	2.695822	-0.162320	0.529574

C	4.314570	-1.572863	-1.222048
H	2.513930	-2.482474	-1.937978
C	4.070348	0.035072	0.530812
H	2.090163	0.406243	1.223663
C	4.895933	-0.667461	-0.341128
H	4.934474	-2.131646	-1.913963
H	4.496653	0.745850	1.229371
H	5.967515	-0.513061	-0.332709
N	0.704551	-1.259686	-0.384718
C	-0.333295	-0.735892	2.398195
H	-1.126547	-0.235435	2.959393
H	-0.452179	-1.812905	2.544942
H	0.627684	-0.445096	2.826477
C	-2.163483	-0.805857	-0.050542
C	-2.752460	-2.022001	0.323166
C	-2.858324	0.017774	-0.943374
C	-3.981657	-2.412756	-0.194904
H	-2.238628	-2.686042	1.011867
C	-4.091772	-0.367306	-1.463255
H	-2.429695	0.969253	-1.243409
C	-4.651604	-1.585380	-1.093424
H	-4.416965	-3.360952	0.098346
H	-4.613810	0.282068	-2.156401
H	-5.608912	-1.889351	-1.500603
C	0.205824	-2.407183	-1.086878
C	0.316504	-3.674320	-0.515971
C	-0.429581	-2.257142	-2.317068
C	-0.220384	-4.781210	-1.164383
H	0.818731	-3.777834	0.440116
C	-0.974258	-3.363561	-2.960567
H	-0.511946	-1.265479	-2.747975
C	-0.872778	-4.626805	-2.384846
H	-0.137753	-5.762572	-0.712458
H	-1.480176	-3.238063	-3.910451
H	-1.299639	-5.487727	-2.885055

Ph_2NH-TS4

Symbol	X	Y	Z
F	-4.097989	-3.074926	-1.844724
F	-4.820059	2.977724	0.367922
F	-2.586564	-0.839540	-1.920420
C	-3.303249	-2.769708	-0.822925
C	-2.757788	4.016679	-0.046137
C	-3.508888	2.877378	0.170574
C	-2.516990	-1.634412	-0.844353
C	-3.258016	-3.597859	0.288636
F	-0.655455	4.990666	-0.450738
C	-1.391970	3.902937	-0.246072
F	-3.692415	0.592380	0.413966
C	-2.889583	1.635526	0.186541
C	-0.816654	2.647889	-0.227094
C	-1.524113	1.468973	-0.018857
C	-1.681963	-1.271361	0.202854
C	-2.434891	-3.276213	1.352123
F	0.512250	2.596610	-0.423850
C	-1.665830	-2.123913	1.291133
B	-0.774299	0.045182	-0.008407
F	-2.386136	-4.069665	2.418877
F	-0.880237	-1.856948	2.346066
C	0.343097	-0.266181	-1.127722
F	-0.374856	1.364445	-2.688365
F	1.192615	-1.961959	0.265794
C	0.449055	0.365548	-2.357839
C	1.224927	-1.318478	-0.912873
C	1.398991	0.002324	-3.300291
C	2.172693	-1.724202	-1.832021
F	1.489808	0.654931	-4.456890
F	2.995091	-2.735217	-1.572892
C	2.265182	-1.042959	-3.033576
F	-4.003320	-4.695882	0.330104
F	3.196460	-1.378212	-3.917135
F	-3.337117	5.208907	-0.058588
H	0.446104	0.288746	1.005687
H	-0.255799	-0.000469	1.308502
C	3.171335	0.555898	0.518724
C	3.957360	-0.562585	0.791800
C	3.397789	1.289963	-0.649065
C	4.946833	-0.946306	-0.108758
H	3.800813	-1.136184	1.695571
C	4.386237	0.895270	-1.540366
H	2.791860	2.163522	-0.861100
C	5.163093	-0.231113	-1.280742
H	5.544841	-1.822741	0.109896
H	4.542932	1.469018	-2.446683
H	5.926244	-0.544154	-1.981929
N	2.089092	0.952641	1.368240
H	1.888705	1.936171	1.210927
C	2.177262	0.687185	2.778785

C	1.844486	-0.576455	3.262688
C	2.541900	1.700378	3.662602
C	1.892854	-0.826722	4.630095
H	1.542285	-1.354789	2.571893
C	2.574319	1.447602	5.029326
H	2.799973	2.681065	3.277823
C	2.255360	0.183202	5.515504
H	1.634926	-1.810917	5.001624
H	2.854480	2.239206	5.713571
H	2.284766	-0.012401	6.580276

PhNH$_2$-add

Symbol	X	Y	Z
B	-0.123292	-0.039794	0.386252
C	1.138745	-0.930677	-0.135552
C	2.320774	-0.341711	-0.579361
C	3.443579	-1.059330	-0.948541
C	3.418308	-2.441739	-0.881189
C	2.274001	-3.076172	-0.438054
C	1.170625	-2.318819	-0.070887
C	-0.431491	1.323611	-0.465350
C	-1.232560	2.318288	0.081968
C	-1.627174	3.464105	-0.583584
C	-1.238265	3.636292	-1.899795
C	-0.472748	2.659825	-2.509852
C	-0.092414	1.531155	-1.798684
C	-1.543962	-0.851904	0.418966
C	-2.016222	-1.370573	-0.782050
C	-3.223548	-2.029548	-0.911815
C	-4.032252	-2.185614	0.202882
C	-3.613946	-1.679902	1.417979
C	-2.391502	-1.028856	1.499304
F	2.431133	0.990792	-0.642971
F	4.549232	-0.435773	-1.350431
F	4.486526	-3.149438	-1.228848
F	2.240929	-4.405027	-0.357478
F	0.117698	-3.014702	0.381077
F	-1.674364	2.192855	1.354708
F	-2.373297	4.384809	0.021044
F	-1.603507	4.721795	-2.571415
F	-0.109904	2.808366	-3.781715
F	0.619403	0.632811	-2.485099
F	-2.055001	-0.565675	2.722015
F	-4.380820	-1.818828	2.496967
F	-5.198081	-2.814067	0.101733
F	-3.617276	-2.512299	-2.087676
F	-1.268239	-1.246419	-1.886696
N	0.278666	0.359812	1.937663
H	-0.480412	0.898924	2.356163
C	1.538156	1.077936	2.120689
C	1.551353	2.462009	2.022549
C	2.701412	0.356246	2.348682
C	2.764134	3.132355	2.130044
H	0.630332	3.012886	1.874883
C	3.909576	1.036447	2.448731
H	2.667271	-0.724190	2.437877
C	3.943031	2.422031	2.331169
H	2.783841	4.212200	2.055325
H	4.822706	0.480300	2.619440
H	4.885948	2.948772	2.407024
H	0.313196	-0.508907	2.474822

PhNH$_2$

Symbol	X	Y	Z
C	0.437802	-0.298880	-0.074158
C	0.016194	1.037632	-0.068264
C	-0.532622	-1.307687	-0.019867
C	-1.335558	1.349567	-0.009542
H	0.758103	1.827974	-0.111845
C	-1.882219	-0.984350	0.038440
H	-0.219728	-2.346452	-0.025328
C	-2.297713	0.344247	0.043876
H	-1.638292	2.390470	-0.004076
H	-2.615227	-1.781858	0.081726
H	-3.350674	0.591770	0.090312
N	1.788947	-0.616997	-0.195603
H	2.039582	-1.525560	0.168277
H	2.420268	0.097613	0.138155

PhNH$_2$-product

Symbol	X	Y	Z
Si	0.648600	1.569617	0.950323
C	1.884405	0.395064	0.165760
C	3.137507	0.171223	0.751733
C	1.596137	-0.247374	-1.044701
C	4.073869	-0.662052	0.147636
H	3.393174	0.654782	1.690229
C	2.527682	-1.085039	-1.651559

H	0.628139	-0.094071	-1.512636
C	3.768199	-1.291913	-1.055768
H	5.038871	-0.821792	0.614276
H	2.286084	-1.576944	-2.586565
H	4.494899	-1.943585	-1.526697
C	-1.772493	0.258670	0.250169
C	-3.087755	0.359547	-0.228945
C	-1.345642	-0.976920	0.761184
C	-3.942738	-0.732647	-0.192222
H	-3.432063	1.307515	-0.629002
C	-2.213174	-2.062253	0.798541
H	-0.327876	-1.096974	1.115196
C	-3.516674	-1.953715	0.325048
H	-4.953291	-0.625831	-0.569754
H	-1.858400	-3.005264	1.198420
H	-4.186565	-2.803698	0.354675
N	-0.921467	1.360622	0.205969
H	-1.321041	2.168426	-0.252844
C	0.570814	1.300413	2.800015
H	1.560678	1.415625	3.248452
H	-0.094182	2.035074	3.260606
H	0.199032	0.304220	3.048631
C	1.126890	3.343043	0.566489
C	0.435906	4.407017	1.164664
C	2.168770	3.646359	-0.318362
C	0.770121	5.727700	0.884763
H	-0.375396	4.205787	1.858637
C	2.508827	4.967000	-0.599722
H	2.722021	2.841776	-0.792875
C	1.809398	6.008322	0.001428
H	0.224419	6.536815	1.355948
H	3.318396	5.182807	-1.287138
H	2.073333	7.036695	-0.216089

$PhNH_2$-TS

Symbol	X	Y	Z
F	2.866113	-3.993051	1.470151
F	3.774030	3.418946	2.102904
F	0.884403	-2.221449	1.580763
C	2.866635	-2.956801	0.636220
C	1.925952	4.297871	0.954764
C	2.679226	3.217485	1.375363
C	1.840650	-2.025429	0.667159
C	3.903439	-2.803178	-0.266764
F	0.045617	5.117447	-0.197527
C	0.779288	4.084692	0.205955
F	3.051270	0.936482	1.495627
C	2.284810	1.930849	1.037319
C	0.419218	2.788451	-0.103707
C	1.147559	1.672172	0.284813
C	1.802055	-0.927832	-0.180957
C	3.908463	-1.718185	-1.127599
F	-0.706276	2.615428	-0.817307
C	2.870429	-0.808256	-1.059277
B	0.658046	0.200117	-0.127532
F	4.903769	-1.560829	-1.995203
F	2.913667	0.242372	-1.894550
C	-0.779867	-0.280628	0.389677
F	-1.044486	1.446881	1.997835
F	-0.748998	-2.051483	-1.160680
C	-1.499985	0.339488	1.402283
C	-1.362084	-1.421292	-0.149384
C	-2.720280	-0.141225	1.850116
C	-2.568580	-1.938919	0.278583
F	-3.394696	0.502690	2.800595
F	-3.077667	-3.035971	-0.273383
C	-3.255368	-1.284875	1.287015
F	4.890136	-3.689179	-0.306429
F	-4.433667	-1.737424	1.693910
F	2.294926	5.532320	1.269291
H	-0.079804	0.353677	-1.614098
H	0.711364	0.434061	-1.575829
C	-2.932405	0.251392	-2.220301
C	-3.549328	-0.933450	-2.623746
C	-3.571446	1.079044	-1.293770
C	-4.790307	-1.284744	-2.103726
H	-3.048372	-1.584133	-3.332578
C	-4.811656	0.720295	-0.781281
H	-3.086172	1.994317	-0.972381
C	-5.427597	-0.464741	-1.178284
H	-5.254945	-2.210936	-2.419879
H	-5.293033	1.365859	-0.055357
H	-6.389748	-0.745032	-0.768791
N	-1.632479	0.575822	-2.677695
H	-1.510501	1.575785	-2.801127
H	-1.406763	0.109927	-3.550185

Al(C_6F_5)$_3$

Symbol	X	Y	Z
F	2.568594	4.470436	1.159348
F	-5.155255	-0.003892	1.151702
F	2.263359	1.789733	1.169786
C	1.503417	3.914462	0.591688
C	-4.354505	-1.889139	0.001242
C	-4.140845	-0.650642	0.587223
C	1.326412	2.542259	0.578952
C	0.538640	4.717986	0.003130
F	-3.524219	-3.762349	-1.148087
C	-3.303916	-2.579220	-0.584497
F	-2.680571	1.068635	1.166586
C	-2.863258	-0.119482	0.576535
C	-2.043320	-2.009110	-0.573410
C	-1.784594	-0.774238	0.001644
C	0.222610	1.934830	0.000113
C	-0.581285	4.152457	-0.587567
F	-1.050247	-2.687891	-1.161932
C	-0.716371	2.775465	-0.577867
F	-1.494327	4.934339	-1.154161
F	-1.797408	2.253727	-1.171537
C	1.564841	-1.159442	-0.002615
F	0.416573	-2.852758	1.167329
F	2.854155	0.429134	-1.172867
C	1.537001	-2.419321	0.575685
C	2.762923	-0.768536	-0.580743
C	2.634657	-3.261612	0.585917
C	3.885654	-1.577092	-0.593131
F	2.581264	-4.462499	1.152517
F	5.019356	-1.179606	-1.161159
C	3.813084	-2.830419	-0.004168
F	0.687626	6.034155	0.005259
F	4.875984	-3.620812	-0.005654
F	-5.569536	-2.416632	0.001380
Al	0.001442	0.000671	-0.000394

Al(C_6F_5)$_3$-TS-A

Symbol	X	Y	Z
Si	3.341334	1.154931	-1.972771
H	-0.479710	-1.760992	0.211334
F	-6.698259	0.222906	-0.800347
F	-3.991809	0.355238	4.960557
F	-4.250954	0.895260	0.113246
C	-5.686711	-0.641985	-0.885302
C	-1.880931	1.336377	4.646787
C	-2.924511	0.552020	4.185021
C	-4.419673	-0.326040	-0.427281
C	-5.897914	-1.891565	-1.446894
F	0.243639	2.290285	4.284069
C	-0.769447	1.540036	3.845123
F	-3.879085	-0.773958	2.537138
C	-2.836128	-0.018362	2.923279
C	-0.741187	0.948923	2.595370
C	-1.751387	0.156799	2.084055
C	-3.341395	-1.190989	-0.490324
C	-4.851238	-2.792780	-1.536624
F	0.362180	1.179616	1.845723
C	-3.603137	-2.422542	-1.058190
F	-5.059195	-3.995166	-2.077584
F	-2.625244	-3.344727	-1.168500
C	-0.958334	0.930462	-0.997221
F	-1.807765	2.656597	0.383608
F	-0.005354	-0.604571	-2.526023
C	-1.253885	2.265831	-0.776096
C	-0.363904	0.666147	-2.218553
C	-1.017189	3.269574	-1.703179
C	-0.101581	1.626086	-3.181060
F	-1.341825	4.536866	-1.447374
F	0.480006	1.314203	-4.342561
C	-0.436095	2.942874	-2.915450
F	-7.104570	-2.223710	-1.899157
F	-0.188236	3.887394	-3.818814
F	-1.943249	1.887623	5.856253
H	2.492360	0.547235	-3.025583
C	2.616581	2.775969	-1.377449
C	2.093052	2.906894	-0.087475
C	2.548694	3.880410	-2.238855
C	1.507685	4.098984	0.332040
H	2.134194	2.074780	0.605826
C	1.970980	5.074039	-1.822218
H	2.938225	3.808218	-3.250492
C	1.449520	5.183966	-0.534741
H	1.094048	4.170458	1.331595
H	1.917057	5.914964	-2.503253
H	0.987902	6.110527	-0.215239
C	4.075600	-0.668128	1.585173

C	4.883841	-0.961536	2.680236
C	4.581603	-0.179024	0.368969
C	6.244739	-0.749507	2.526703
H	4.471573	-1.341949	3.606705
C	5.958332	0.023493	0.237611
C	6.775453	-0.263332	1.320293
H	6.912324	-0.961746	3.352571
H	6.377583	0.394975	-0.690319
H	7.844376	-0.109074	1.238806
C	3.458742	-0.075572	-0.565379
C	2.342010	-0.477956	0.200434
H	3.630923	-1.268830	-1.408140
N	2.702705	-0.838408	1.438292
C	1.824724	-1.319914	2.495897
H	2.239929	-2.236834	2.913883
H	1.739452	-0.559978	3.274571
H	0.845686	-1.529021	2.068585
H	1.305745	-0.547748	-0.104739
C	3.693109	-3.278004	-0.799694
C	2.336769	-3.636940	-0.767031
C	1.753063	-4.311018	0.302095
C	2.591356	-4.641672	1.355661
C	3.952495	-4.293793	1.345085
C	4.512689	-3.599660	0.283351
C	3.880288	-2.478241	-2.010045
C	2.648388	-2.574042	-2.702487
H	0.698805	-4.559230	0.311165
H	2.186323	-5.173201	2.208097
H	5.555927	-3.304740	0.296730
H	4.824554	-2.303116	-2.508913
N	1.740796	-3.189610	-1.953074
C	0.350758	-3.457082	-2.308249
H	0.142717	-2.998511	-3.271153
H	0.202631	-4.535799	-2.366859
H	-0.303942	-3.029260	-1.551216
H	4.573194	-4.564847	2.190516
H	2.380390	-2.166962	-3.667208
Al	-1.562087	-0.577751	0.216589
H	4.704212	1.338661	-2.523074

Al(C_6F_5)$_3$-TS-B

Symbol	X	Y	Z
H	-0.232983	-0.272542	-1.754516
F	-4.916403	-2.346517	2.171220
F	3.348986	-2.903697	1.049472
F	-3.000841	-0.447376	1.915564
C	-4.063394	-2.382601	1.150728
C	3.253405	-0.857854	2.213056
C	2.702307	-1.775806	1.334692
C	-3.079085	-1.423736	0.993590
C	-4.146486	-3.404394	0.217117
F	3.140897	1.205655	3.324985
C	2.584933	0.323376	2.496943
F	1.000147	-2.344044	-0.160616
C	1.492126	-1.468494	0.741747
C	1.368713	0.575696	1.885981
C	0.794066	-0.300639	0.981702
C	-2.175390	-1.424632	-0.054013
C	-3.257293	-3.451897	-0.844266
F	0.756435	1.730106	2.186329
C	-2.291098	-2.465049	-0.957836
F	-3.344786	-4.438362	-1.734204
F	-1.451363	-2.550644	-2.001890
C	-1.443480	1.876585	-0.231649
F	-3.742632	1.348114	-0.100841
F	0.792744	2.596629	-0.416574
C	-2.771817	2.267050	-0.195925
C	-0.519494	2.900548	-0.350138
C	-3.168598	3.593525	-0.262507
C	-0.861184	4.238082	-0.424041
F	-4.454869	3.931065	-0.223804
F	0.063625	5.186656	-0.545696
C	-2.203643	4.580955	-0.376170
F	-5.078998	-4.340938	0.341987
F	-2.564591	5.855592	-0.446417
F	4.432056	-1.100091	2.772077
H	0.611519	-0.209501	-1.709561
H	2.634681	-1.043172	-4.111343
H	3.897771	-2.731203	-3.031567
C	3.962466	-1.163922	-1.519257
C	3.238803	0.026523	-1.507123
C	3.647823	1.120911	-0.757829
C	4.825328	0.999993	-0.018506
C	5.564128	-0.180532	-0.031463
C	5.131589	-1.273591	-0.783348
C	3.237167	-2.184820	-2.358662
C	2.231687	-1.313971	-3.130323
H	3.077405	2.040998	-0.739339

H	5.163655	1.839591	0.577240
H	5.696999	-2.198232	-0.787788
H	2.730294	-2.909656	-1.717941
N	2.067860	-0.069899	-2.324989
C	1.780702	1.109107	-3.151270
H	2.608859	1.315936	-3.835665
H	0.875303	0.917676	-3.730593
H	1.607839	1.973847	-2.512745
H	6.475059	-0.251490	0.549662
H	1.258105	-1.788325	-3.269532
Al	-0.823760	0.009504	-0.092545

$Al(C_6F_5)_3$-TS-C

Symbol	X	Y	Z
F	2.647345	4.331718	-2.980729
F	5.480158	-1.503689	-1.040162
F	1.895461	1.747082	-2.745521
C	1.995426	3.916431	-1.896482
C	3.842650	-3.141276	-0.642366
C	4.197016	-1.821449	-0.863504
C	1.602704	2.597702	-1.746729
C	1.688825	4.819779	-0.890640
F	2.183439	-4.739657	-0.168502
C	2.517682	-3.471841	-0.408201
F	3.618201	0.422176	-1.007436
C	3.212478	-0.847027	-0.844825
C	1.572983	-2.461225	-0.405861
C	1.876575	-1.129387	-0.628020
C	0.927195	2.125592	-0.636035
C	1.000927	4.397422	0.235131
F	0.299546	-2.814233	-0.148334
C	0.631650	3.065659	0.333835
F	0.703361	5.272370	1.195543
F	-0.046722	2.709325	1.444446
C	-1.231054	-0.265191	-1.508420
F	-0.314796	-2.026552	-2.788519
F	-2.325267	1.436908	-0.298860
C	-1.378221	-1.298214	-2.416488
C	-2.384605	0.430162	-1.199098
C	-2.598905	-1.638036	-2.979097
C	-3.622333	0.144399	-1.743648
F	-2.707444	-2.650123	-3.838911
F	-4.710599	0.838683	-1.402283
C	-3.726743	-0.910985	-2.633620
F	2.050235	6.092952	-1.008793
F	-4.911510	-1.235911	-3.141054
F	4.776157	-4.087673	-0.628336
C	-3.801794	-0.269519	1.693328
C	-4.816064	-0.901360	0.979878
C	-2.588421	-0.896078	2.017257
C	-4.559253	-2.193598	0.546066
H	-5.744678	-0.398572	0.740751
C	-2.346741	-2.193042	1.560787
C	-3.337245	-2.826919	0.823977
H	-5.311998	-2.719856	-0.028444
H	-1.402097	-2.686089	1.760532
H	-3.166908	-3.830453	0.453641
C	-1.746028	0.091636	2.699320
C	-2.559186	1.244472	2.749107
H	-0.814916	0.264175	1.629077
N	-3.742023	1.040476	2.168324
C	-4.769402	2.038824	1.913064
H	-4.868241	2.180867	0.836087
H	-5.718521	1.702663	2.330187
H	-4.475123	2.973997	2.382886
H	-2.320965	2.212140	3.169391
Si	-0.453902	-0.272373	4.030234
H	-0.073189	0.080746	1.070579
H	-0.877912	-1.482895	4.767527
C	1.254318	-0.557463	3.311691
C	1.794940	-1.842237	3.200652
C	2.030171	0.530065	2.879570
C	3.079660	-2.038043	2.694280
H	1.217938	-2.703434	3.523899
C	3.308460	0.339839	2.369914
H	1.632403	1.538639	2.945504
C	3.838799	-0.947155	2.287992
H	3.486939	-3.040653	2.626041
H	3.894037	1.190565	2.041348
H	4.841562	-1.094974	1.902193
Al	0.423050	0.210102	-0.519949
H	-0.426068	0.919702	4.906746

$B(2,6\text{-}Cl_2C_6H_3)(p\text{-}HC_6F_4)_2$-TS-A

Symbol	X	Y	Z
Si	2.892874	2.649879	-0.959240
H	-0.677002	-0.884470	-0.162120
H	4.214848	3.312513	-0.863152

F	-4.783910	-2.021773	3.719828
C	-4.156795	-2.472293	-2.811027
C	-2.792359	-0.886237	4.256766
C	-3.663764	-1.404243	3.317456
C	-3.619157	-1.479890	-1.997543
C	-3.713442	-3.778055	-2.677691
F	-0.770852	0.275203	4.641234
C	-1.661014	-0.249227	3.784320
F	-4.327326	-1.815674	1.128146
C	-3.406941	-1.296424	1.958324
C	-1.428202	-0.148898	2.424136
C	-2.268539	-0.680325	1.456483
C	-2.613443	-1.688017	-1.043624
C	-2.755219	-4.072745	-1.718894
F	-0.304437	0.503972	2.045807
C	-2.245569	-3.040859	-0.943544
B	-1.845851	-0.573781	-0.119010
C	-1.893635	0.977391	-0.627372
F	-3.836946	1.509602	0.613864
F	0.030760	0.696131	-2.004695
C	-2.837782	1.902727	-0.195065
C	-0.965847	1.484892	-1.523042
C	-2.853808	3.219070	-0.618353
C	-0.982514	2.791826	-1.986415
F	-3.790697	4.052288	-0.140752
F	-0.054066	3.184215	-2.872646
C	-1.927635	3.685922	-1.533065
H	2.582456	2.464927	-2.396994
C	1.570126	3.695899	-0.141057
C	0.816523	3.247136	0.947707
C	1.318322	4.982403	-0.638588
C	-0.168334	4.050062	1.517625
H	0.987029	2.261072	1.363303
C	0.348487	5.793820	-0.061237
H	1.881231	5.357436	-1.488468
C	-0.400163	5.325785	1.016569
H	-0.751777	3.673518	2.350094
H	0.169634	6.787345	-0.456014
H	-1.167522	5.951627	1.457430
C	3.800949	-0.434467	1.555752
C	4.646684	-1.084815	2.449895
C	4.235886	0.585784	0.692452
C	5.973213	-0.684482	2.459987
H	4.288312	-1.872460	3.101314
C	5.579683	0.969664	0.719575
C	6.433859	0.330472	1.605108
H	6.667860	-1.161902	3.140017
H	5.946577	1.748791	0.061620
H	7.477489	0.618016	1.642320
C	3.099609	0.962729	-0.154912
C	2.043059	0.149846	0.318888
H	3.402682	0.245170	-1.402131
N	2.455364	-0.668596	1.293027
C	1.636588	-1.652328	1.990242
H	2.152377	-2.612425	1.988143
H	1.460327	-1.322863	3.015326
H	0.687041	-1.748025	1.466036
H	1.012983	0.107597	-0.007372
C	3.942127	-1.784965	-1.665070
C	2.685962	-2.410312	-1.691387
C	2.387564	-3.549252	-0.948267
C	3.415871	-4.072861	-0.179255
C	4.686199	-3.473271	-0.150102
C	4.959470	-2.324456	-0.875880
C	3.808460	-0.561548	-2.454105
C	2.526021	-0.644582	-3.045959
H	1.400681	-3.995795	-0.962972
H	3.234879	-4.961801	0.412761
H	5.931118	-1.847422	-0.821240
H	4.624728	0.001694	-2.887027
N	1.856443	-1.680972	-2.551311
C	0.468890	-2.038872	-2.826301
H	0.422301	-3.094587	-3.094248
H	-0.134553	-1.848683	-1.940230
H	0.105358	-1.430572	-3.650616
H	5.462770	-3.911693	0.464670
H	2.048484	0.042087	-3.730287
H	-2.992571	-0.970324	5.316003
H	-1.935498	4.714058	-1.866979
H	-2.407624	-5.086253	-1.566963
H	-4.124513	-4.562459	-3.300501
H	-4.927196	-2.217033	-3.526770
Cl	-4.364087	0.105501	-2.194853
Cl	-1.055656	-3.543573	0.266570

$B(2,6\text{-}Cl_2C_6H_3)(p\text{-}HC_6F_4)_2$-TS-B

Symbol	X	Y	Z
H	-0.541714	-0.368848	-1.445403

F	3.320775	-2.180365	1.443063
C	-2.888788	-3.196065	1.591764
C	2.696400	-0.159589	2.487790
C	2.444790	-1.181238	1.594539
C	-2.260863	-1.993388	1.311307
C	-2.989573	-4.148860	0.587649
F	1.955221	1.853741	3.455483
C	1.756880	0.847270	2.597507
F	1.115440	-2.166004	-0.060674
C	1.292939	-1.171090	0.828548
C	0.604180	0.841409	1.828755
C	0.345476	-0.159186	0.902063
C	-1.701263	-1.668389	0.068361
C	-2.489150	-3.873795	-0.675065
F	-0.272461	1.833388	2.027726
C	-1.871354	-2.651685	-0.909070
B	-0.941053	-0.251734	-0.064290
C	-1.851726	1.055005	-0.299420
F	-3.942691	-0.064240	-0.103381
F	0.083827	2.370591	-0.568348
C	-3.242494	1.055772	-0.310855
C	-1.258030	2.287611	-0.546054
C	-3.977019	2.213639	-0.534805
C	-1.974341	3.444582	-0.781021
F	-5.310787	2.137212	-0.522036
F	-1.316487	4.583256	-1.016165
C	-3.356652	3.421895	-0.774682
H	0.225934	-0.249391	-1.241760
H	2.748612	-0.987618	-3.693744
H	4.354406	-2.139826	-2.405165
C	3.970129	-0.441495	-1.099571
C	2.986088	0.533521	-1.272550
C	3.107117	1.787133	-0.683591
C	4.237995	2.042426	0.092216
C	5.222295	1.074364	0.272734
C	5.089040	-0.178632	-0.327062
C	3.552941	-1.705487	-1.806853
C	2.387704	-1.215302	-2.683497
H	2.348763	2.547995	-0.811639
H	4.346951	3.013523	0.560784
H	5.849160	-0.939728	-0.190127
H	3.226911	-2.456316	-1.085030
N	1.904517	0.047426	-2.059597
C	1.364945	0.979433	-3.051469
H	1.024753	1.891897	-2.566871
H	2.117656	1.231436	-3.808231
H	0.510892	0.511941	-3.548036
H	6.093442	1.295931	0.877247
H	1.570386	-1.932347	-2.756966
H	3.600677	-0.145843	3.080852
H	-3.932692	4.319715	-0.953608
H	-2.578140	-4.591975	-1.479024
H	-3.472154	-5.097530	0.784749
H	-3.297319	-3.373839	2.577570
Cl	-2.244708	-0.791606	2.590549
Cl	-1.326140	-2.381914	-2.561372

B(2,6-$Cl_2C_6H_3$)(p-HC_6F_4)$_2$-TS-C

Symbol	X	Y	Z
F	5.144021	1.657829	-0.862653
C	0.175259	4.618504	1.289280
C	4.141026	-0.247440	-1.812430
C	4.056290	0.901006	-1.054841
C	0.262326	3.457298	0.538384
C	0.028162	4.518857	2.665694
F	3.029646	-2.158736	-2.635933
C	2.995854	-1.009544	-1.952045
F	2.901276	2.393770	0.293890
C	2.863535	1.283850	-0.456012
C	1.814376	-0.606815	-1.364299
C	1.691180	0.554940	-0.611650
C	0.211735	2.160749	1.074163
C	-0.025245	3.268726	3.259818
F	0.736782	-1.402937	-1.511158
C	0.065453	2.133506	2.462567
B	0.270923	0.908908	0.049765
C	-1.047232	0.879489	-0.875591
F	0.081402	0.925471	-2.961780
F	-2.370792	0.853842	1.078352
C	-1.061320	0.942768	-2.262079
C	-2.293460	0.942721	-0.263057
C	-2.237896	1.074304	-2.983924
C	-3.473127	1.095604	-0.968096
F	-2.173244	1.133741	-4.319878
F	-4.629820	1.179525	-0.300350
C	-3.459996	1.159012	-2.348211
C	-3.285505	-1.933124	0.776733
C	-4.549904	-1.757085	0.216458

C	-2.150153	-2.277156	0.020486
C	-4.656431	-1.936444	-1.151531
H	-5.406524	-1.483365	0.819849
C	-2.282810	-2.435037	-1.364812
C	-3.534037	-2.265208	-1.932753
H	-5.618929	-1.807321	-1.631613
H	-1.422207	-2.676956	-1.976753
H	-3.653755	-2.382596	-3.003190
C	-1.020554	-2.389208	0.944738
C	-1.590912	-2.132656	2.201239
H	-0.306000	-0.859470	0.827033
N	-2.895697	-1.833929	2.104663
C	-3.757962	-1.360711	3.173985
H	-4.112404	-0.357403	2.932423
H	-4.607717	-2.033317	3.293742
H	-3.185636	-1.329628	4.098246
H	-1.118151	-2.153966	3.173686
Si	0.379374	-3.611428	0.669445
H	0.227008	-0.182856	0.901232
H	0.258452	-4.139954	-0.706341
C	2.100591	-2.911860	0.902690
C	3.173597	-3.467134	0.194065
C	2.360664	-1.846014	1.771034
C	4.462712	-2.966151	0.340806
H	3.002021	-4.288884	-0.494257
C	3.645197	-1.328488	1.907647
H	1.554441	-1.398858	2.344370
C	4.697738	-1.888652	1.190133
H	5.279907	-3.402693	-0.221835
H	3.821216	-0.487040	2.568305
H	5.697889	-1.482457	1.288387
H	0.172699	-4.729942	1.622789
H	5.076635	-0.556187	-2.258750
H	-4.377238	1.264948	-2.911598
H	-0.137353	3.163868	4.330609
H	-0.042133	5.412678	3.272294
H	0.226650	5.581487	0.798923
Cl	-0.022206	0.603518	3.331665
Cl	0.486138	3.663817	-1.191501

B(2,6-$Cl_2C_6H_3$)(p-HC_6F_4)$_2$

Symbol	X	Y	Z
F	4.788279	-0.115108	-1.135797
C	-0.251770	3.868400	-1.182085
C	3.876850	-1.885743	0.119309
C	3.738066	-0.679205	-0.537559
C	-0.249449	2.482769	-1.156138
C	0.000290	4.554631	0.000690
F	2.858724	-3.598302	1.375041
C	2.762190	-2.439071	0.722209
F	2.449555	1.123273	-1.241957
C	2.511938	-0.035065	-0.583535
C	1.536667	-1.801514	0.657532
C	1.370203	-0.579657	0.004280
C	0.000386	1.748572	-0.002831
C	0.252412	3.865453	1.181732
F	0.502873	-2.380788	1.272152
C	0.250170	2.479889	1.152300
B	0.000292	0.174309	-0.004800
C	-1.369910	-0.579179	-0.008736
F	-0.507057	-2.383153	-1.275734
F	-2.444829	1.126676	1.237198
C	-1.538784	-1.802185	-0.659236
C	-2.509729	-0.032880	0.581158
C	-2.764844	-2.439161	-0.719398
C	-3.736337	-0.676402	0.539717
F	-2.863795	-3.599531	-1.369839
F	-4.784613	-0.110602	1.139743
C	-3.877563	-1.884088	-0.114517
H	4.835129	-2.386236	0.161975
H	-4.836231	-2.384125	-0.153664
Cl	0.534938	1.581316	2.629087
Cl	-0.533951	1.587948	-2.635258
H	0.444455	4.390570	2.107892
H	0.000268	5.637196	0.002048
H	-0.443793	4.395819	-2.106940

Ga(C_6F_5)$_3$

Symbol	X	Y	Z
F	-3.765707	3.543238	-1.145742
F	4.960017	1.471767	-1.168540
F	-2.690918	1.069029	-1.162934
C	-2.581923	3.323043	-0.583368
C	4.741195	-0.554813	0.000383
C	4.177564	0.563356	-0.595270
C	-2.012580	2.061948	-0.575532
C	-1.891820	4.373544	0.002180
F	4.488436	-2.578012	1.167849

C	3.935318	-1.513707	0.595458
F	2.279896	1.783806	-1.179688
C	2.800987	0.704177	-0.584430
C	2.563155	-1.334913	0.584037
C	1.962682	-0.231377	-0.000106
C	-0.777039	1.807926	-0.002422
C	-0.651664	4.160010	0.584437
F	1.808356	-2.266608	1.178849
C	-0.117763	2.883262	0.571050
F	-0.003223	5.174078	1.147463
F	1.072146	2.699021	1.155757
C	-1.177096	-1.579587	0.005033
F	0.412419	-2.872725	-1.159598
F	-2.865352	-0.418433	1.169720
C	-0.787118	-2.776626	-0.573562
C	-2.437220	-1.540852	0.579845
C	-1.602999	-3.894218	-0.588505
C	-3.284118	-2.634939	0.585772
F	-1.209307	-5.030287	-1.154469
F	-4.486287	-2.575399	1.148994
C	-2.857958	-3.815301	-0.003909
F	-2.421436	5.587761	0.005595
F	-3.654791	-4.873584	-0.008895
F	6.056987	-0.708249	0.000856
Ga	0.005137	-0.001749	0.001178

$Ga(C_6F_5)_3$-TS-A

Symbol	X	Y	Z
Si	3.446132	1.125720	-1.962757
H	-0.433573	-1.772680	0.187550
H	4.817381	1.285314	-2.499597
F	-6.656369	0.318566	-0.782407
F	-3.948116	0.418585	4.933213
F	-4.198834	0.949276	0.134420
C	-5.652911	-0.553324	-0.891410
C	-1.836611	1.401546	4.629102
C	-2.873833	0.609635	4.165721
C	-4.381231	-0.257214	-0.431916
C	-5.879096	-1.787992	-1.479642
F	0.288422	2.357393	4.277312
C	-0.717966	1.599384	3.836048
F	-3.808559	-0.736262	2.519431
C	-2.772796	0.026075	2.910961
C	-0.674383	0.995984	2.592304
C	-1.679430	0.197259	2.084151
C	-3.315383	-1.133437	-0.521251
C	-4.841590	-2.697034	-1.593955
F	0.434354	1.221517	1.850008
C	-3.587865	-2.349952	-1.112885
F	-5.063323	-3.885363	-2.160238
F	-2.616823	-3.276298	-1.245274
C	-0.862067	0.963204	-1.029100
F	-1.717220	2.679225	0.360546
F	0.098288	-0.568462	-2.556540
C	-1.152242	2.296469	-0.796251
C	-0.255828	0.701734	-2.243878
C	-0.894988	3.306335	-1.711298
C	0.024299	1.669472	-3.193785
F	-1.210556	4.574240	-1.446489
F	0.617510	1.364313	-4.351330
C	-0.303727	2.986277	-2.920444
F	-7.091244	-2.098377	-1.933588
F	-0.039434	3.936861	-3.813114
F	-1.911824	1.965621	5.832283
H	2.596893	0.531204	-3.022653
C	2.742827	2.760332	-1.378116
C	2.205257	2.904460	-0.095186
C	2.704498	3.863061	-2.243534
C	1.634608	4.107519	0.312846
H	2.223660	2.074802	0.602087
C	2.141549	5.067631	-1.838066
H	3.105175	3.781127	-3.250023
C	1.605225	5.190449	-0.558056
H	1.209287	4.189261	1.306667
H	2.110455	5.906982	-2.522439
H	1.154691	6.125391	-0.247287
C	4.123480	-0.702720	1.603639
C	4.919671	-1.012600	2.702951
C	4.646173	-0.230565	0.387873
C	6.286050	-0.834957	2.554164
H	4.494345	-1.379707	3.628887
C	6.028014	-0.063570	0.261043
C	6.833416	-0.366696	1.348130
H	6.944939	-1.060752	3.383405
H	6.460027	0.293555	-0.666686
H	7.906186	-0.239655	1.270352
C	3.530110	-0.104108	-0.552023
C	2.400958	-0.476099	0.210861

H	3.683885	-1.305611	-1.385399
N	2.747344	-0.840056	1.451615
C	1.855274	-1.299927	2.506867
H	2.242715	-2.230995	2.920470
H	1.792210	-0.541359	3.288927
H	0.870337	-1.477629	2.079400
H	1.364635	-0.516871	-0.097922
C	3.683069	-3.314025	-0.765770
C	2.318933	-3.644085	-0.769462
C	1.691707	-4.300327	0.286244
C	2.493188	-4.642851	1.364272
C	3.861338	-4.323993	1.390023
C	4.465543	-3.647871	0.340924
C	3.921202	-2.525516	-1.974401
C	2.707660	-2.599059	-2.700652
H	0.632643	-4.526530	0.267595
H	2.053310	-5.160857	2.207713
H	5.514288	-3.375621	0.381479
H	4.882605	-2.373596	-2.447514
N	1.766230	-3.191290	-1.974345
C	0.380890	-3.428327	-2.367588
H	0.218298	-2.992451	-3.349674
H	0.202799	-4.503467	-2.401329
H	-0.285833	-2.960209	-1.645979
H	4.452468	-4.604025	2.253561
H	2.476560	-2.193149	-3.675250
Ga	-1.492118	-0.567874	0.196469

$Ga(C_6F_5)_3$-TS-B

Symbol	X	Y	Z
H	-0.141737	-0.242766	-1.796810
F	-4.806945	-2.521463	2.149113
F	3.548423	-2.761181	1.118163
F	-2.977692	-0.540459	1.901557
C	-3.936245	-2.531160	1.142850
C	3.307491	-0.750927	2.317806
C	2.840835	-1.667402	1.390119
C	-2.994917	-1.528799	0.991099
C	-3.957394	-3.567250	0.222019
F	3.046763	1.272491	3.480066
C	2.575989	0.392333	2.598198
F	1.236985	-2.273660	-0.196635
C	1.647992	-1.400282	0.744362
C	1.377892	0.606202	1.937261
C	0.890867	-0.270359	0.985474
C	-2.076959	-1.505667	-0.042418
C	-3.049137	-3.586666	-0.823917
F	0.693224	1.717395	2.243670
C	-2.125348	-2.559143	-0.935411
F	-3.075956	-4.587124	-1.702223
F	-1.262049	-2.619225	-1.960676
C	-1.460322	1.869165	-0.230005
F	-3.729208	1.216669	-0.166179
F	0.744117	2.698896	-0.345528
C	-2.807107	2.186906	-0.224451
C	-0.581901	2.934390	-0.312052
C	-3.267646	3.492895	-0.286348
C	-0.992155	4.253302	-0.380718
F	-4.569991	3.764708	-0.277486
F	-0.114540	5.249568	-0.466494
C	-2.350857	4.528526	-0.363865
F	-4.847935	-4.544656	0.344454
F	-2.773059	5.784757	-0.428995
F	4.472410	-0.955471	2.920424
H	0.715148	-0.166306	-1.809565
H	2.757452	-0.771139	-4.251905
H	4.000963	-2.526114	-3.250114
C	4.001355	-1.067934	-1.631732
C	3.270045	0.115918	-1.564715
C	3.648261	1.164969	-0.738547
C	4.804778	1.003831	0.025243
C	5.553220	-0.169210	-0.040923
C	5.150892	-1.215268	-0.872200
C	3.312868	-2.032735	-2.564054
C	2.326281	-1.116873	-3.307067
H	3.070034	2.078739	-0.679358
H	5.120138	1.806010	0.682387
H	5.724797	-2.133510	-0.918970
H	2.789900	-2.802644	-1.992608
N	2.122773	0.061103	-2.417619
C	1.816681	1.293977	-3.151652
H	1.588736	2.093648	-2.448308
H	2.658504	1.588756	-3.784910
H	0.938438	1.118803	-3.775550
H	6.447718	-0.271056	0.560861
H	1.362004	-1.586407	-3.510970
Ga	-0.764683	0.001245	-0.094389

$Ga(C_6F_5)_3$-TS-C

Symbol	X	Y	Z
F	4.052835	-3.596273	1.643976
F	5.024586	3.468541	1.346048
F	2.479664	-1.441365	2.024911
C	3.515077	-2.966250	0.602469
C	3.303507	4.559349	0.172104
C	3.832672	3.418951	0.753934
C	2.695450	-1.862283	0.771889
C	3.779548	-3.415807	-0.682499
F	1.561555	5.612299	-1.001866
C	2.063025	4.511599	-0.443830
F	3.639521	1.157134	1.283297
C	3.100888	2.244199	0.705309
C	1.370029	3.312364	-0.469162
C	1.861547	2.151640	0.099048
C	2.116670	-1.185424	-0.287618
C	3.239675	-2.752336	-1.771920
F	0.177682	3.309949	-1.085859
C	2.435588	-1.645541	-1.549918
F	3.486420	-3.191255	-3.005802
F	1.948697	-1.016093	-2.638128
C	-0.531685	0.085219	1.412263
F	-1.178301	2.340446	1.181671
F	-0.058916	-2.212321	1.733625
C	-1.376437	1.130665	1.739108
C	-0.794159	-1.125936	2.027984
C	-2.440165	1.002306	2.615168
C	-1.819250	-1.292822	2.944136
F	-3.248936	2.026877	2.881092
F	-2.034665	-2.466159	3.536150
C	-2.655486	-0.224495	3.221689
F	4.553337	-4.478864	-0.868243
F	-3.677238	-0.385460	4.056236
F	3.985311	5.699166	0.202724
C	-1.949450	-2.874729	-0.546082
C	-2.461443	-3.647127	0.493075
C	-2.503063	-1.647780	-0.950188
C	-3.592281	-3.164591	1.128234
H	-1.999571	-4.579708	0.791955
C	-3.637295	-1.172399	-0.274972
C	-4.168880	-1.941862	0.748300
H	-4.030026	-3.731150	1.940300
H	-4.100499	-0.231841	-0.546294
H	-5.048492	-1.589553	1.274525
C	-1.700233	-1.160470	-2.083872
C	-0.757761	-2.191128	-2.297879
H	-0.701229	-0.217818	-1.600105
N	-0.867630	-3.153787	-1.381961
C	0.049699	-4.265746	-1.180472
H	0.619205	-4.097000	-0.262977
H	-0.513046	-5.195087	-1.098396
H	0.723751	-4.328345	-2.032121
H	0.023932	-2.236277	-3.044469
Si	-2.352236	-0.006913	-3.428296
H	-0.053345	0.510073	-1.403131
H	-3.196717	-0.747648	-4.390612
C	-3.388389	1.276834	-2.548984
C	-4.748504	1.421343	-2.842871
C	-2.853244	2.020455	-1.487026
C	-5.554639	2.274296	-2.092409
H	-5.191629	0.851894	-3.653634
C	-3.656383	2.864612	-0.729192
H	-1.801464	1.942609	-1.232952
C	-5.010885	2.989106	-1.030632
H	-6.607215	2.372051	-2.330715
H	-3.223846	3.417348	0.096217
H	-5.639657	3.643049	-0.437722
Ga	0.900862	0.388219	0.035964
H	-1.168587	0.559025	-4.114483

CH-a-TS

Symbol	X	Y	Z
F	-0.455144	4.114273	-3.872508
F	5.429142	2.120131	-1.231472
F	0.521435	1.677801	-3.281215
C	-0.412267	3.701562	-2.608284
C	5.084765	-0.000164	-0.287466
C	4.590441	1.154131	-0.863123
C	0.075622	2.449512	-2.281941
C	-0.856712	4.537091	-1.595474
F	4.673707	-2.113968	0.659450
C	4.207739	-1.001581	0.097224
F	2.827865	2.450532	-1.606354
C	3.220755	1.304552	-1.040488
C	2.855869	-0.801480	-0.082138
C	2.302818	0.336761	-0.654919
C	0.148021	1.979077	-0.978409

C	-0.800311	4.109653	-0.281251
F	2.032014	-1.776039	0.346717
C	-0.298598	2.848216	-0.000106
B	0.704500	0.489416	-0.726078
F	-1.225182	4.907613	0.696160
F	-0.275925	2.486891	1.295474
C	-0.165930	-0.630952	-1.483324
F	1.644946	-1.873608	-2.380008
F	-2.129491	0.414384	-0.681260
C	0.330518	-1.709679	-2.204729
C	-1.553871	-0.571926	-1.387305
C	-0.486939	-2.681439	-2.761226
C	-2.402538	-1.519825	-1.928800
F	0.037343	-3.723548	-3.400679
F	-3.719959	-1.445674	-1.742534
C	-1.860588	-2.590194	-2.618375
F	-1.330353	5.743801	-1.886268
F	-2.648912	-3.530515	-3.123128
F	6.388214	-0.143330	-0.077821
H	-0.053988	-0.318096	1.066816
Si	0.653330	-1.667367	3.251453
H	0.440349	0.310291	0.586679
H	0.999189	-3.053247	2.880319
C	2.165443	-0.573298	3.112702
C	3.434810	-1.131794	3.309606
C	2.079954	0.792916	2.818658
C	4.582530	-0.351425	3.213059
H	3.535921	-2.191714	3.521084
C	3.226607	1.572549	2.700452
H	1.115992	1.264301	2.659479
C	4.479514	1.000413	2.899480
H	5.556885	-0.803255	3.357327
H	3.139938	2.624216	2.452750
H	5.374440	1.604556	2.802819
H	0.109896	-1.644194	4.628292
C	-2.810936	-0.019957	2.072413
C	-1.584603	0.100991	2.619642
C	-0.777859	-1.036914	2.172553
C	-1.687534	-1.807104	1.425433
O	-2.867661	-1.188881	1.354530
H	-1.236954	0.931886	3.212632
C	-3.995843	0.830128	2.042156
C	-4.168193	1.814595	3.018968
C	-4.944635	0.684469	1.026842
C	-5.277804	2.645856	2.978205
H	-3.439930	1.918158	3.815187
C	-6.056003	1.517310	0.996378
H	-4.795745	-0.064233	0.258362
C	-6.225304	2.497617	1.968659
H	-5.407839	3.406248	3.738353
H	-6.788062	1.404387	0.206120
H	-7.092499	3.145990	1.940167
C	-1.626752	-3.098593	0.744598
C	-0.406918	-3.686089	0.399272
C	-2.825081	-3.732893	0.381901
C	-0.384402	-4.882818	-0.305755
H	0.522884	-3.193299	0.639505
C	-2.793754	-4.927236	-0.320384
H	-3.772124	-3.280639	0.646541
C	-1.574392	-5.502578	-0.671421
H	0.566297	-5.317641	-0.588314
H	-3.722412	-5.405021	-0.605865
H	-1.553239	-6.427067	-1.235073

CH-b-TS

Symbol	X	Y	Z
F	4.222937	-1.693139	-2.997759
F	-3.073895	-4.155986	-2.536394
F	1.966836	-0.268619	-3.067913
C	3.295671	-1.879712	-2.061825
C	-4.097371	-2.382523	-1.387270
C	-2.973056	-2.975418	-1.929330
C	2.119241	-1.149209	-2.074136
C	3.513638	-2.811215	-1.059698
F	-5.077103	-0.561977	-0.267927
C	-3.990247	-1.151168	-0.759274
F	-0.708216	-2.939200	-2.420882
C	-1.747408	-2.326957	-1.842589
C	-2.745991	-0.566754	-0.651082
C	-1.588672	-1.104084	-1.207456
C	1.138116	-1.274007	-1.098058
C	2.547467	-2.999797	-0.089155
F	-2.675395	0.596119	0.018757
C	1.395503	-2.233604	-0.131777
B	-0.179996	-0.350386	-1.042668
F	2.731697	-3.898008	0.877626
F	0.507764	-2.440067	0.863097
C	-0.040625	1.177455	-1.526221

F	-2.231977	1.382621	-2.435311
F	2.221995	1.259913	-0.828699
C	-1.076724	1.942485	-2.060167
C	1.130573	1.891797	-1.273700
C	-0.993762	3.314401	-2.233920
C	1.253392	3.260332	-1.433888
F	-2.036820	4.003151	-2.692293
F	2.386597	3.882932	-1.121384
C	0.172456	3.982030	-1.904673
F	4.638718	-3.516623	-1.033202
F	0.253119	5.298134	-2.049720
F	-5.276302	-2.990997	-1.455551
H	-0.334462	0.280717	1.091100
Si	-1.604786	0.501699	3.509552
H	-0.299101	-0.198444	0.333543
H	-2.362153	1.766839	3.504945
C	-2.646390	-0.894330	2.823373
C	-4.038990	-0.776656	2.751777
C	-2.064222	-2.104787	2.425743
C	-4.823327	-1.838312	2.309330
H	-4.521583	0.152179	3.038108
C	-2.840155	-3.158637	1.957942
H	-0.990926	-2.242576	2.475663
C	-4.225331	-3.027075	1.905731
H	-5.899531	-1.727930	2.257180
H	-2.365354	-4.080181	1.641955
H	-4.836299	-3.845896	1.543331
H	-1.136937	0.173112	4.875450
C	2.178177	0.480463	2.178186
C	-0.022089	0.751610	2.494038
C	0.610314	1.937017	2.027428
H	0.995522	-1.113347	2.822839
C	3.438299	-0.228967	1.942121
C	3.710031	-1.452576	2.560292
C	4.336046	0.295836	1.008216
C	4.858116	-2.157994	2.226839
H	3.037207	-1.857120	3.308938
C	5.479026	-0.418178	0.675348
H	4.109591	1.243777	0.538792
C	5.737328	-1.647530	1.276155
H	5.060452	-3.110369	2.700174
H	6.164207	-0.020052	-0.062921
H	6.622767	-2.208469	1.003618
C	-0.010534	3.214086	1.646893
C	-1.339479	3.281290	1.222193
C	0.777598	4.370510	1.625616
C	-1.878337	4.491200	0.797801
H	-1.944052	2.384086	1.175271
C	0.235643	5.574933	1.201346
H	1.815257	4.303816	1.927725
C	-1.093664	5.639076	0.788354
H	-2.903739	4.528470	0.450254
H	0.854046	6.463709	1.179004
H	-1.509343	6.577299	0.441846
N	1.931971	1.744663	1.844336
N	1.070925	-0.121230	2.646772

CH-c-TS

Symbol	X	Y	Z
F	-0.972462	3.913364	-3.969304
F	5.202694	2.595570	-1.212504
F	0.239794	1.590777	-3.367761
C	-0.825217	3.548793	-2.698095
C	5.058346	0.453879	-0.264705
C	4.459442	1.551877	-0.851365
C	-0.214266	2.353531	-2.364808
C	-1.284701	4.377096	-1.686390
F	4.850739	-1.692349	0.673623
C	4.281618	-0.630968	0.108788
F	2.586693	2.658211	-1.632680
C	3.084812	1.562703	-1.049979
C	2.918551	-0.568578	-0.088183
C	2.263144	0.508371	-0.672883
C	-0.032877	1.933833	-1.054522
C	-1.118611	4.000995	-0.365614
F	2.194328	-1.621145	0.329917
C	-0.494695	2.797142	-0.077275
B	0.662638	0.508814	-0.783464
F	-1.553727	4.794017	0.610647
F	-0.361872	2.487032	1.223518
C	-0.087535	-0.703013	-1.526044
F	1.835157	-1.779844	-2.398503
F	-2.150228	0.175013	-0.771692
C	0.511572	-1.745141	-2.223256
C	-1.472422	-0.780536	-1.427129
C	-0.206394	-2.809194	-2.748377
C	-2.219573	-1.836194	-1.915134
F	0.418270	-3.809087	-3.364502

F	-3.536386	-1.893709	-1.686236
C	-1.580102	-2.864468	-2.583155
F	-1.876372	5.528724	-1.984192
F	-2.268616	-3.902236	-3.038954
F	6.366253	0.443073	-0.036674
H	-0.058929	-0.299243	1.036477
Si	0.718996	-1.627082	3.269093
H	0.384966	0.314253	0.550384
H	1.166145	-2.983154	2.890092
C	2.158547	-0.433406	3.117432
C	3.463863	-0.894694	3.330514
C	1.979640	0.918494	2.801059
C	4.553375	-0.035079	3.231385
H	3.639014	-1.941259	3.559815
C	3.067357	1.778540	2.681512
H	0.985843	1.315424	2.627435
C	4.356504	1.302071	2.899273
H	5.556487	-0.413592	3.389178
H	2.905991	2.817837	2.418811
H	5.206530	1.968028	2.802396
H	0.224905	-1.655355	4.665659
C	-2.831607	-0.135082	2.072694
C	-1.590123	0.022050	2.615120
C	-0.751996	-1.094520	2.208630
C	-1.618615	-1.946142	1.468753
H	-1.266771	0.881913	3.182114
C	-3.998303	0.752567	2.049704
C	-4.272467	1.574984	3.145878
C	-4.831880	0.805744	0.928220
C	-5.361198	2.435596	3.119482
H	-3.637567	1.524260	4.022849
C	-5.926943	1.660867	0.911153
H	-4.603313	0.208778	0.051755
C	-6.193868	2.477639	2.004721
H	-5.565717	3.068806	3.974157
H	-6.563738	1.698652	0.035788
H	-7.045136	3.147051	1.987585
C	-1.422858	-3.258391	0.838610
C	-0.157733	-3.680076	0.424342
C	-2.527909	-4.082816	0.581076
C	-0.001525	-4.885335	-0.249593
H	0.700927	-3.047394	0.588779
C	-2.369861	-5.279379	-0.102774
H	-3.515045	-3.804471	0.932706
C	-1.106402	-5.682128	-0.527092
H	0.984987	-5.186146	-0.581137
H	-3.234784	-5.898923	-0.304202
H	-0.986032	-6.610373	-1.071511
N	-2.824139	-1.343093	1.399987
H	-3.599842	-1.684857	0.846806

CH-d-TS

Symbol	X	Y	Z
F	3.738085	-2.066621	-2.944882
F	-4.138271	-3.329535	-2.400909
F	1.761596	-0.292321	-2.912950
C	2.750643	-2.201142	-2.063779
C	-4.743226	-1.250894	-1.492621
C	-3.777020	-2.161864	-1.871872
C	1.708432	-1.287491	-2.020841
C	2.764045	-3.260447	-1.174583
F	-5.295051	0.831120	-0.552608
C	-4.364933	-0.044497	-0.924915
F	-1.557551	-2.772865	-2.106617
C	-2.434962	-1.852467	-1.691753
C	-3.023528	0.209599	-0.733643
C	-2.008026	-0.657598	-1.129813
C	0.663602	-1.365423	-1.106313
C	1.736523	-3.388966	-0.257586
F	-2.705596	1.374407	-0.145960
C	0.715922	-2.456628	-0.251768
B	-0.473810	-0.241279	-0.929219
F	1.767057	-4.377573	0.640086
F	-0.247923	-2.620087	0.671598
C	-0.038174	1.241709	-1.388813
F	-1.951207	1.749944	-2.704071
F	1.981028	0.964989	-0.213236
C	-0.775780	2.112498	-2.180213
C	1.191483	1.737992	-0.972786
C	-0.350024	3.399157	-2.485582
C	1.666154	2.998928	-1.269085
F	-1.110352	4.203998	-3.225344
F	2.855593	3.407328	-0.828331
C	0.872856	3.847708	-2.022396
F	3.760946	-4.137587	-1.185321
F	1.280091	5.083657	-2.286592
F	-6.032064	-1.541410	-1.633789
H	-0.238864	-0.073204	1.343509

Si	-1.554836	0.415807	3.648696
H	-0.513648	-0.131684	0.463922
H	-1.763220	1.865962	3.827842
C	-2.996078	-0.335022	2.717179
C	-4.234073	0.319003	2.723784
C	-2.927468	-1.617986	2.154780
C	-5.373060	-0.298524	2.214714
H	-4.316501	1.319676	3.136155
C	-4.062921	-2.237624	1.646317
H	-1.983572	-2.148539	2.107895
C	-5.290760	-1.581987	1.686513
H	-6.321356	0.224845	2.223897
H	-3.989730	-3.230755	1.217714
H	-6.177105	-2.063265	1.288929
H	-1.441368	-0.240668	4.973462
C	3.968647	-0.858701	1.394171
C	4.551520	-2.120603	1.224340
C	4.553168	0.247115	0.771122
C	5.668710	-2.276480	0.417228
H	4.134813	-2.989438	1.721098
C	5.674640	0.085573	-0.034001
H	4.110779	1.226485	0.890771
C	6.229955	-1.174335	-0.222334
H	6.097782	-3.261611	0.283327
H	6.107689	0.949394	-0.523463
H	7.097025	-1.298906	-0.859255
C	0.834473	2.347850	2.131296
C	-0.399530	2.727180	1.588359
C	1.867710	3.286425	2.213292
C	-0.597029	4.026818	1.144582
H	-1.192521	2.004306	1.443003
C	1.657851	4.590491	1.785379
H	2.830361	2.987771	2.608261
C	0.428778	4.962629	1.248883
H	-1.547174	4.299867	0.701472
H	2.461961	5.312333	1.852740
H	0.276291	5.975522	0.895763
C	1.083269	0.947737	2.520392
C	0.124966	0.016858	2.856389
C	1.912708	-1.685632	2.586133
C	2.725338	-0.717839	2.173875
H	2.145437	-2.720457	2.378610
O	2.377829	0.616696	2.397415
C	0.652862	-1.343468	3.315437
H	-0.093042	-2.123046	3.154353
H	0.826707	-1.307976	4.398559

CH-e-TS

Symbol	X	Y	Z
F	-1.471550	4.541636	-2.239331
F	4.207962	1.391556	-2.304302
F	-0.654108	1.975579	-2.358410
C	-1.280988	3.847395	-1.120974
C	3.731376	-0.868671	-1.898058
C	3.364559	0.462434	-1.859772
C	-0.860233	2.531167	-1.158421
C	-1.509232	4.445795	0.109143
F	3.249818	-3.103204	-1.328057
C	2.878716	-1.824877	-1.370837
F	1.894639	2.140465	-1.265378
C	2.144625	0.828443	-1.306746
C	1.675608	-1.414286	-0.837624
C	1.242789	-0.095634	-0.799063
C	-0.636305	1.770995	-0.018416
C	-1.316947	3.720080	1.270524
F	0.901465	-2.367663	-0.277197
C	-0.893232	2.401997	1.185722
B	-0.212573	0.225725	-0.198879
F	-1.545012	4.286790	2.454276
F	-0.749609	1.744174	2.350650
C	-1.443253	-0.630188	-0.786579
F	-0.409922	-1.291937	-2.814247
F	-2.660711	-0.083454	1.152566
C	-1.470669	-1.302947	-2.001225
C	-2.613747	-0.696588	-0.043396
C	-2.578746	-2.014726	-2.435119
C	-3.735671	-1.406062	-0.428823
F	-2.564161	-2.645470	-3.606452
F	-4.809683	-1.465084	0.351250
C	-3.714721	-2.069484	-1.643320
F	-1.915208	5.709093	0.170745
F	-4.775416	-2.756561	-2.048619
F	4.918552	-1.223403	-2.376893
H	-0.260725	-0.947742	1.452046
Si	1.434085	-2.342408	2.889915
H	-0.101831	-0.109043	1.087166
H	1.872598	-3.572838	2.204892
C	2.485306	-0.892673	2.368654

C	3.694739	-1.081803	1.690350
C	2.064238	0.416683	2.630364
C	4.452656	0.008285	1.272516
H	4.044256	-2.086458	1.470091
C	2.806895	1.508474	2.196423
H	1.131926	0.597273	3.156326
C	4.003424	1.302734	1.515059
H	5.384621	-0.151757	0.742003
H	2.452799	2.514909	2.386917
H	4.581542	2.150354	1.165824
H	1.429335	-2.518620	4.359479
C	-2.523927	-2.071187	3.126621
C	-0.400511	-2.055073	2.454761
C	-1.222171	-2.895931	1.672827
H	-1.109267	-0.934703	4.160682
N	-2.502920	-2.888727	2.080838
N	-1.318539	-1.569910	3.403608
H	-3.407474	-1.813815	3.691182
H	-0.902857	-3.470771	0.814900

CH-f-TS

Symbol	X	Y	Z
F	-1.453719	4.832370	-1.761872
F	4.431937	1.914247	-1.894494
F	-0.496258	2.352223	-2.197754
C	-1.287880	3.990739	-0.744714
C	4.088057	-0.389594	-1.606747
C	3.622593	0.910609	-1.558610
C	-0.794135	2.714386	-0.944431
C	-1.618232	4.392522	0.540859
F	3.722779	-2.674164	-1.181263
C	3.255141	-1.426546	-1.221836
F	1.968576	2.449955	-1.070506
C	2.327460	1.162044	-1.125781
C	1.973570	-1.132311	-0.805286
C	1.450861	0.153408	-0.753467
C	-0.594235	1.807051	0.086837
C	-1.455652	3.513959	1.596786
F	1.208682	-2.162785	-0.396715
C	-0.955265	2.245085	1.347549
B	-0.069627	0.319817	-0.251821
F	-1.786262	3.888678	2.831609
F	-0.851423	1.422610	2.412573
C	-1.180388	-0.478476	-1.114926
F	0.156414	-0.898773	-3.028101
F	-2.708362	-0.166676	0.655198
C	-1.009411	-1.012046	-2.386244
C	-2.449925	-0.637899	-0.580100
C	-2.018128	-1.682159	-3.063870
C	-3.479394	-1.310285	-1.211399
F	-1.804734	-2.178086	-4.279582
F	-4.654452	-1.486032	-0.601774
C	-3.259064	-1.840369	-2.470551
F	-2.096280	5.613771	0.755547
F	-4.226310	-2.497274	-3.100267
F	5.344276	-0.641728	-1.963028
H	-0.391088	-1.066438	1.469831
Si	0.795334	-2.551660	2.856222
H	-0.003157	-0.256233	0.897973
H	1.004320	-3.864521	2.227540
C	2.174332	-1.367950	2.488258
C	3.375178	-1.868185	1.971466
C	2.059928	0.008264	2.724588
C	4.434958	-1.011467	1.690454
H	3.481858	-2.928106	1.763127
C	3.108675	0.866751	2.420245
H	1.141470	0.424794	3.125689
C	4.296368	0.355952	1.901648
H	5.356758	-1.409078	1.281067
H	2.999893	1.932423	2.582764
H	5.112398	1.026442	1.657871
H	0.521283	-2.658286	4.303499
C	-0.985458	-1.980504	2.171146
C	-1.841122	-1.357971	3.109462
N	-3.138834	-1.628149	3.210375
C	-3.607226	-2.507496	2.335381
C	-2.792154	-3.137374	1.370929
N	-1.505272	-2.896212	1.287688
H	-1.445786	-0.613940	3.795270
H	-4.665797	-2.740124	2.387242
H	-3.220420	-3.848117	0.671665

NH-a-TS

Symbol	X	Y	Z
H	-0.034315	-0.127487	-1.678299
F	-1.968696	-4.197119	1.856832
F	4.107072	-2.512532	-0.122946
F	-0.206215	-2.199967	1.737918

C	-2.037570	-3.317654	0.861120
C	3.874285	-0.678141	1.330805
C	3.357753	-1.528575	0.367784
C	-1.128492	-2.273893	0.774331
C	-3.028950	-3.443120	-0.096727
F	3.601134	1.192022	2.723146
C	3.102434	0.369331	1.803742
F	1.619337	-2.136085	-1.051969
C	2.075869	-1.309180	-0.098742
C	1.829236	0.566950	1.293551
C	1.273871	-0.257818	0.324906
C	-1.166766	-1.333809	-0.245805
C	-3.106630	-2.522981	-1.129297
F	1.163871	1.627770	1.761981
C	-2.184137	-1.493390	-1.176846
B	-0.168103	-0.068910	-0.363290
F	-4.057972	-2.637512	-2.052511
F	-2.289802	-0.617164	-2.188989
C	-0.952076	1.313861	-0.093705
F	-1.476628	0.545999	2.064924
F	-0.525025	2.268782	-2.220693
C	-1.590413	1.492276	1.127053
C	-1.107297	2.342588	-1.003832
C	-2.331748	2.617444	1.435704
C	-1.840786	3.489427	-0.740490
F	-2.919431	2.748903	2.620388
F	-1.953443	4.451157	-1.653035
C	-2.457670	3.624415	0.489721
F	-3.903315	-4.439564	-0.025321
F	-3.164159	4.713421	0.766335
F	5.106851	-0.859356	1.789031
H	0.765611	0.290125	-1.892993
C	2.477352	1.833385	-1.775345
C	3.737397	1.406238	-1.558071
C	3.936688	0.193502	-2.325411
C	2.791797	-0.075948	-2.983474
N	1.820418	0.895044	-2.617760
H	1.255060	1.290505	-3.368754
H	1.933832	2.688365	-1.405727
H	4.462394	1.879639	-0.914319
H	4.834649	-0.404466	-2.351289
H	2.521235	-0.880325	-3.647576

NH-b-TS

Symbol	X	Y	Z
H	-0.098231	-0.112987	-1.678452
F	-2.801993	-3.736784	1.897736
F	3.527586	-2.835408	0.818503
F	-0.835223	-1.938442	1.894630
C	-2.662966	-2.917016	0.860472
C	3.473803	-0.716869	1.845194
C	2.894772	-1.685912	1.039542
C	-1.643889	-1.978773	0.831977
C	-3.547485	-2.997662	-0.201725
F	3.372523	1.414453	2.820099
C	2.822114	0.485785	2.044129
F	1.158371	-2.368752	-0.345400
C	1.673217	-1.422403	0.456437
C	1.603720	0.720569	1.419308
C	0.995306	-0.216804	0.597532
C	-1.464103	-1.100917	-0.228145
C	-3.406425	-2.139515	-1.279534
F	1.047062	1.913954	1.642438
C	-2.379766	-1.212099	-1.265619
B	-0.333611	0.041448	-0.257514
F	-4.250468	-2.212663	-2.303838
F	-2.272336	-0.395868	-2.324974
C	-0.902974	1.540299	-0.295237
F	-2.657945	1.029279	1.204617
F	0.820432	2.264095	-1.728909
C	-2.018173	1.918833	0.437978
C	-0.284810	2.547160	-1.020363
C	-2.505881	3.216315	0.440903
C	-0.740675	3.852072	-1.048960
F	-3.575778	3.538344	1.160671
F	-0.115295	4.781279	-1.764972
C	-1.864410	4.186026	-0.310403
F	-4.525396	-3.893460	-0.187661
F	-2.318714	5.432122	-0.318072
F	4.650171	-0.942029	2.412947
H	0.717745	-0.095373	-1.641157
C	4.313563	-1.372531	-1.988121
C	4.597912	-0.075023	-1.616136
C	3.366112	0.597035	-1.702285
N	2.996269	-1.412856	-2.266329
H	5.553749	0.327418	-1.328082
N	2.400686	-0.224613	-2.099639
H	2.447213	-2.213170	-2.542085

H	3.135236	1.629592	-1.493018
H	4.933285	-2.249783	-2.073736

NH-c-TS

Symbol	X	Y	Z
H	0.114297	0.288040	-1.420185
F	-1.311097	5.161936	0.296989
F	-4.239899	-1.656489	-2.741452
F	-2.199357	2.678822	0.093627
C	-0.435984	4.166198	0.210916
C	-4.654247	-0.871016	-0.566698
C	-3.791428	-1.093941	-1.623389
C	-0.878165	2.852420	0.114147
C	0.918986	4.440599	0.199469
F	-5.007593	-0.091923	1.624613
C	-4.180965	-0.300275	0.605955
F	-1.647985	-1.004024	-2.534595
C	-2.459165	-0.733881	-1.495665
C	-2.843319	0.040907	0.690392
C	-1.947380	-0.147941	-0.350731
C	-0.001410	1.777751	0.031515
C	1.827863	3.397895	0.100013
F	-2.402647	0.583844	1.831628
C	1.351054	2.107522	0.023755
B	-0.407747	0.223095	-0.101359
F	3.131886	3.651173	0.082801
F	2.256672	1.118456	-0.050661
C	0.299572	-0.806738	0.914844
F	-0.468123	-2.632228	-0.375878
F	1.103436	0.838828	2.427875
C	0.194422	-2.175103	0.696616
C	0.988156	-0.439679	2.067944
C	0.783339	-3.126073	1.509816
C	1.570552	-1.364476	2.921383
F	0.707020	-4.417546	1.200423
F	2.215010	-0.965684	4.010593
C	1.480598	-2.715907	2.634574
F	1.347789	5.691179	0.278495
F	2.045109	-3.607778	3.430354
F	-5.933563	-1.208495	-0.668754
H	0.286973	-0.513123	-1.351024
C	1.876011	-0.938905	-3.445901
C	2.940527	0.061688	-3.013445
C	3.478560	-1.753572	-1.047096
C	2.408255	-2.592613	-1.726878
H	3.308478	0.618825	-3.874947
H	2.286670	-1.599973	-4.219399
H	1.028340	-0.398000	-3.875762
H	4.219038	-2.380162	-0.548960
H	3.017761	-1.092347	-0.310519
H	2.867744	-3.224336	-2.500187
H	1.935373	-3.256609	-1.001993
H	2.535091	0.766917	-2.289071
H	0.596599	-2.281057	-2.603051
N	1.382441	-1.714379	-2.297978
S	4.390023	-0.750799	-2.267249

NH-d-TS

Symbol	X	Y	Z
H	0.191539	-0.258198	1.387094
F	0.940446	3.267836	-3.528234
F	4.579104	-1.303573	-1.057896
F	1.036049	0.769180	-2.598286
C	0.413168	2.987345	-2.341627
C	2.983283	-3.015847	-1.295567
C	3.314368	-1.704790	-0.989460
C	0.454903	1.697133	-1.836296
C	-0.187690	3.991772	-1.601926
F	1.344119	-4.680733	-1.506426
C	1.665376	-3.426079	-1.211214
F	2.672413	0.423542	-0.324475
C	2.311685	-0.833926	-0.610763
C	0.688097	-2.523564	-0.814787
C	0.974192	-1.199658	-0.500769
C	-0.084203	1.361334	-0.602719
C	-0.747559	3.697214	-0.369196
F	-0.558641	-2.996881	-0.754568
C	-0.695614	2.395126	0.094206
B	-0.124621	-0.135704	-0.021350
F	-1.326982	4.656006	0.346810
F	-1.260201	2.139868	1.284293
C	-1.636830	-0.656525	0.153154
F	-1.992959	-0.212880	-2.130187
F	-1.437355	-1.241509	2.433832
C	-2.471747	-0.638786	-0.954823
C	-2.184254	-1.164464	1.318504
C	-3.782384	-1.079334	-0.915425
C	-3.493380	-1.608883	1.404464

F	-4.547367	-1.049207	-2.001081
F	-3.979964	-2.077029	2.548946
C	-4.295885	-1.563732	0.277586
F	-0.231626	5.230187	-2.072538
F	-5.551400	-1.986575	0.337896
F	3.925869	-3.870343	-1.663606
H	0.398771	0.509428	1.363727
C	0.892336	1.694904	3.713465
C	1.211896	0.276410	4.198612
C	3.168735	0.221983	2.905537
C	2.920412	1.651348	2.419946
H	0.836912	0.169156	5.217919
H	1.306845	2.417066	4.430997
H	-0.187358	1.837875	3.653812
H	4.245608	0.047163	2.940587
H	2.772523	-0.468938	2.148564
H	3.410499	2.349366	3.114134
H	3.333352	1.798579	1.424984
H	0.643454	-0.442112	3.595979
H	1.287749	2.824174	2.052035
N	1.470876	1.879069	2.374598
O	2.633763	-0.027929	4.209877

NH-e-TS

Symbol	X	Y	Z
H	-0.166796	-0.081686	-1.679505
F	-2.769499	-3.889768	1.798851
F	3.423624	-2.926642	0.719759
F	-0.807374	-2.094230	1.863360
C	-2.681086	-2.980640	0.833128
C	3.497857	-0.800494	1.719722
C	2.841114	-1.756551	0.961112
C	-1.665507	-2.037459	0.841427
C	-3.611918	-2.972754	-0.190812
F	3.519195	1.332715	2.700350
C	2.893245	0.419594	1.962189
F	1.014509	-2.387307	-0.316786
C	1.594378	-1.458148	0.448426
C	1.642208	0.683093	1.422689
C	0.957552	-0.234563	0.636917
C	-1.535989	-1.067211	-0.143397
C	-3.520943	-2.022472	-1.195218
F	1.127918	1.886108	1.691707
C	-2.498517	-1.093815	-1.145426
B	-0.409482	0.068368	-0.127144
F	-4.411367	-2.010233	-2.181511
F	-2.445708	-0.183750	-2.127862
C	-0.927142	1.579724	-0.201470
F	-3.021294	1.117777	0.810235
F	1.103546	2.267011	-1.172263
C	-2.171735	1.988852	0.258423
C	-0.123091	2.580900	-0.731188
C	-2.597526	3.307611	0.190218
C	-0.510705	3.902865	-0.823471
F	-3.795903	3.655934	0.647139
F	0.298628	4.821441	-1.341104
C	-1.764327	4.266763	-0.356265
F	-4.586627	-3.870874	-0.211972
F	-2.158851	5.530053	-0.428384
F	4.705494	-1.054253	2.206945
H	0.623686	-0.066653	-1.651089
C	3.442223	0.449198	-1.588063
C	3.024662	-1.501010	-2.382727
C	4.347072	-1.507770	-2.042573
N	4.597138	-0.255320	-1.536851
H	3.360060	1.468765	-1.246797
H	2.439516	-2.302766	-2.802110
H	5.107843	-2.265966	-2.105639
N	2.470208	-0.275542	-2.094125
H	5.485109	0.085230	-1.201205

NH-f-TS

Symbol	X	Y	Z
H	-0.125609	-0.172012	1.612634
F	-3.612697	-2.706350	-2.212884
F	-3.683550	2.877140	2.403568
F	-2.085559	-0.505250	-1.918639
C	-2.801652	-2.592122	-1.167396
C	-2.856618	3.632725	0.335340
C	-2.877437	2.702081	1.361780
C	-2.021473	-1.464920	-0.989845
C	-2.725078	-3.619501	-0.238367
F	-2.003996	4.333986	-1.735664
C	-2.016256	3.444219	-0.748927
F	-2.095354	0.706177	2.279127
C	-2.051049	1.596768	1.275022
C	-1.197499	2.326457	-0.793833
C	-1.183974	1.373201	0.214387

C	-1.152277	-1.312252	0.081567
C	-1.870482	-3.507977	0.844047
F	-0.419300	2.190532	-1.870077
C	-1.103443	-2.361920	0.982442
B	-0.290012	0.041757	0.173802
F	-1.795538	-4.489000	1.737361
F	-0.297562	-2.288335	2.055659
C	1.071909	0.110891	-0.659190
F	1.721554	1.970841	0.620331
F	0.581873	-1.710477	-2.099707
C	1.995391	1.106506	-0.365526
C	1.401309	-0.731296	-1.712164
C	3.195713	1.252018	-1.032424
C	2.586499	-0.601629	-2.423390
F	4.064233	2.196017	-0.683456
F	2.865262	-1.426082	-3.426961
C	3.490549	0.386427	-2.075129
F	-3.466913	-4.707932	-0.390712
F	4.632730	0.507723	-2.735086
F	-3.642437	4.699117	0.390399
H	1.844313	-0.382165	3.441093
C	3.429783	0.649203	2.721578
C	2.926137	-1.751640	2.408182
H	3.919377	0.773223	1.752499
H	2.913570	1.589309	2.924693
H	2.073104	-2.429009	2.358709
H	0.600506	0.129233	1.533946
N	2.394417	-0.389056	2.584334
C	3.761667	-1.871514	1.140596
H	3.982532	-2.918919	0.930956
H	3.218720	-1.458233	0.289618
H	4.713092	-1.342146	1.230247
C	4.481383	0.392106	3.799569
H	4.009661	0.210582	4.769267
H	5.110612	-0.467597	3.560430
H	5.132918	1.262238	3.898284
H	3.533017	-2.059824	3.269045
